# Supplementary material for: 4D single-cell spatial transcriptomics reveals dynamic morphogenetic gradients and regenerative domains in planarians
Source: Gigascience. 2026 May 22;15:giag064. doi: 10.1093/gigascience/giag064 (PMC13273413; doi:10.1093/gigascience/giag064)
Supplement: giag064_Supplemental_Files [file giag064_Supplemental_Files.zip › SupplementaryMaterial-20260515.docx]

**
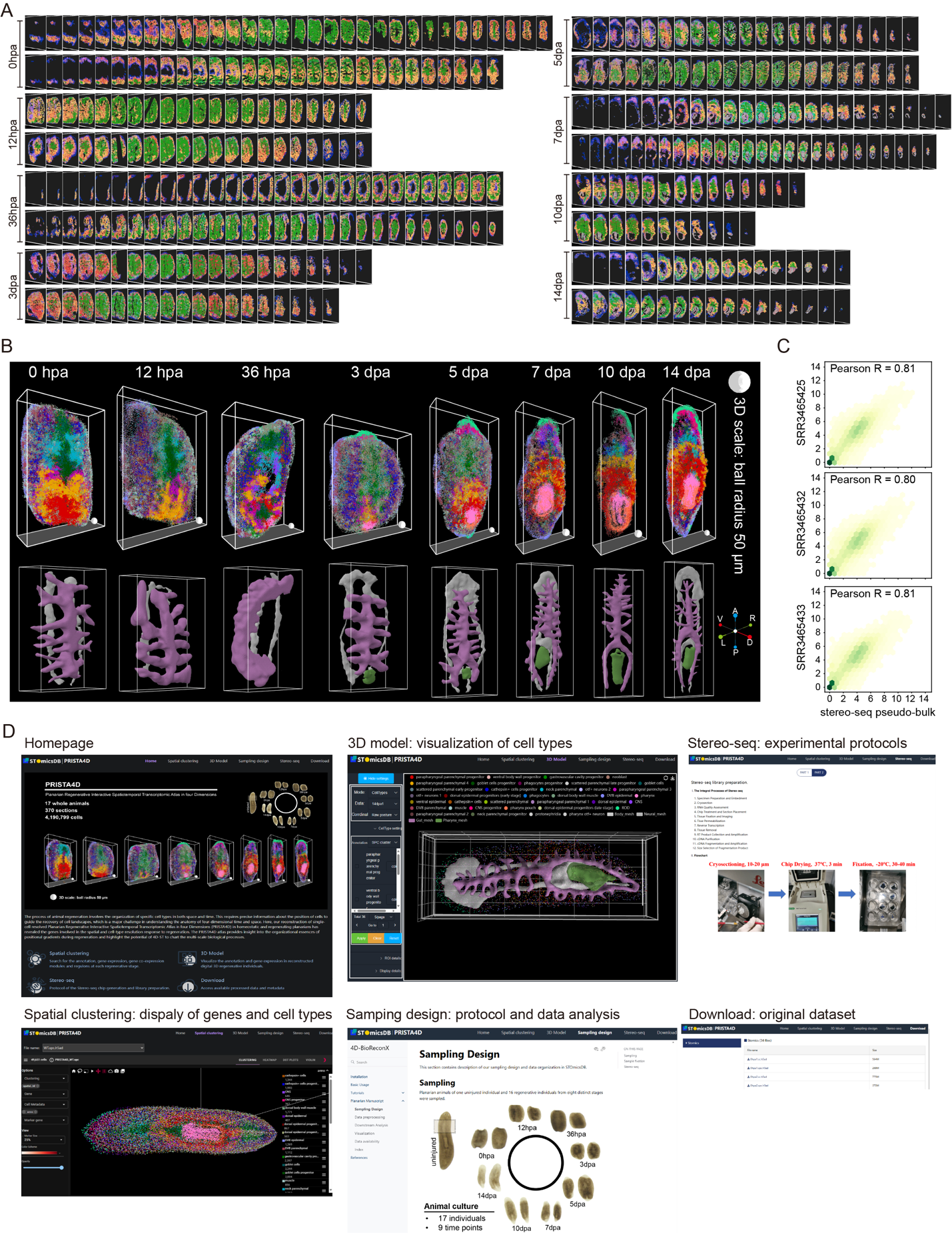
**

**Supplementary Figure S1: High-resolution four-dimensional spatial and molecular characterization of planarian regeneration. (A)** Consecutive Stereo-seq sections utilized for three-dimensional reconstruction across eight regenerative time points. Two animals were sampled per time point, with each row representing an individual animal. Sections display the spatial distribution of cell types along the ventral-to-dorsal axis from left to right. Cells are color-coded according to lineage annotations as detailed in Figure 1B. hpa, hours post-amputation; dpa, days post-amputation. **(B)** Three-dimensional spatial visualization of 36 spatial transcriptomic clusters (top row) and tissue meshes (bottom row) across identical regenerative time points. Cells are color-coded based on cluster annotations. Organisms are oriented with the anterior upwards in a dorsal view. **(C)** Correlation analysis between Stereo-seq pseudo-bulk data during homeostasis and three independent bulk RNA-seq replicates (Roberts-Galbraith et al., 2016). Pearson correlation coefficients are indicated for each replicate. **(D)** Overview of the interactive Planarian Regenerative Interactive Spatiotemporal Transcriptomic Atlas in Four Dimensions (PRISTA4D) database (https://db.cngb.org/stomics/prista4d/). The homepage and five principal functional modules are highlighted. The three-dimensional model module visualizes cell types, while the spatial clustering module illustrates gene and cell type distributions. The Stereo-seq module details experimental protocols, the sampling design module provides information on experimental design and analytical pipelines, and the download module ensures access to the complete original dataset.


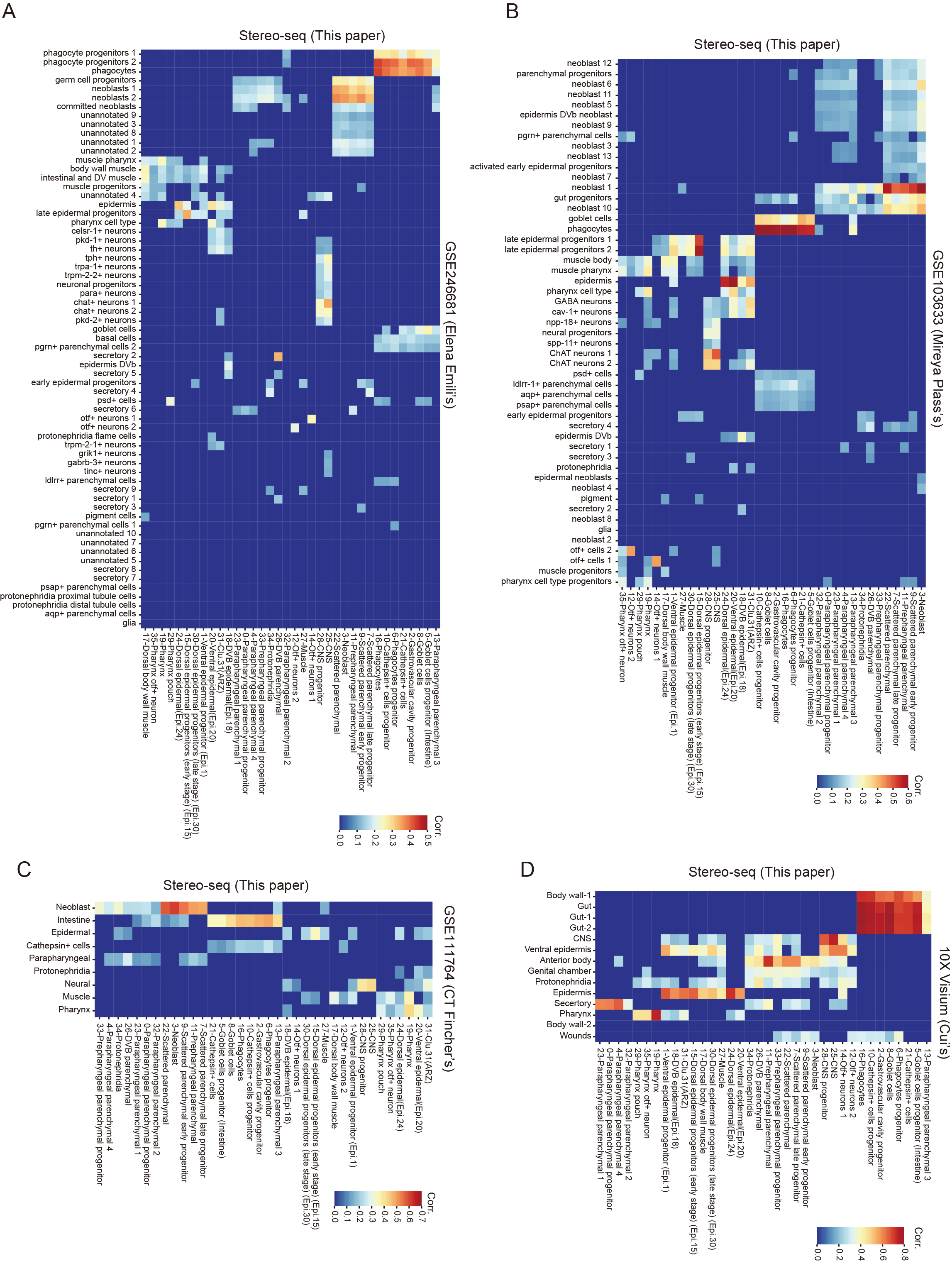


**Supplementary Figure S2: Consistency assessment of cell type annotations across independent datasets.** Heatmaps illustrate the correlation-based correspondence between cell type clusters identified in the current study using Stereo-seq (x-axis) and four previously published reference datasets (y-axis). Comparisons are detailed between the present dataset and those generated by **(A)** Emili et al. (GSE246681), **(B)** Plass et al. (GSE103633)**, (C)** Fincher et al. (GSE111764), and **(D)** Cui et al. (10x Visium). The color gradient reflects the correlation coefficient, with red denoting high correlation and blue denoting low correlation. Robust diagonal signals validate the reliability of the cell type nomenclature and functional annotations applied in this study against established planarian spatial atlases.


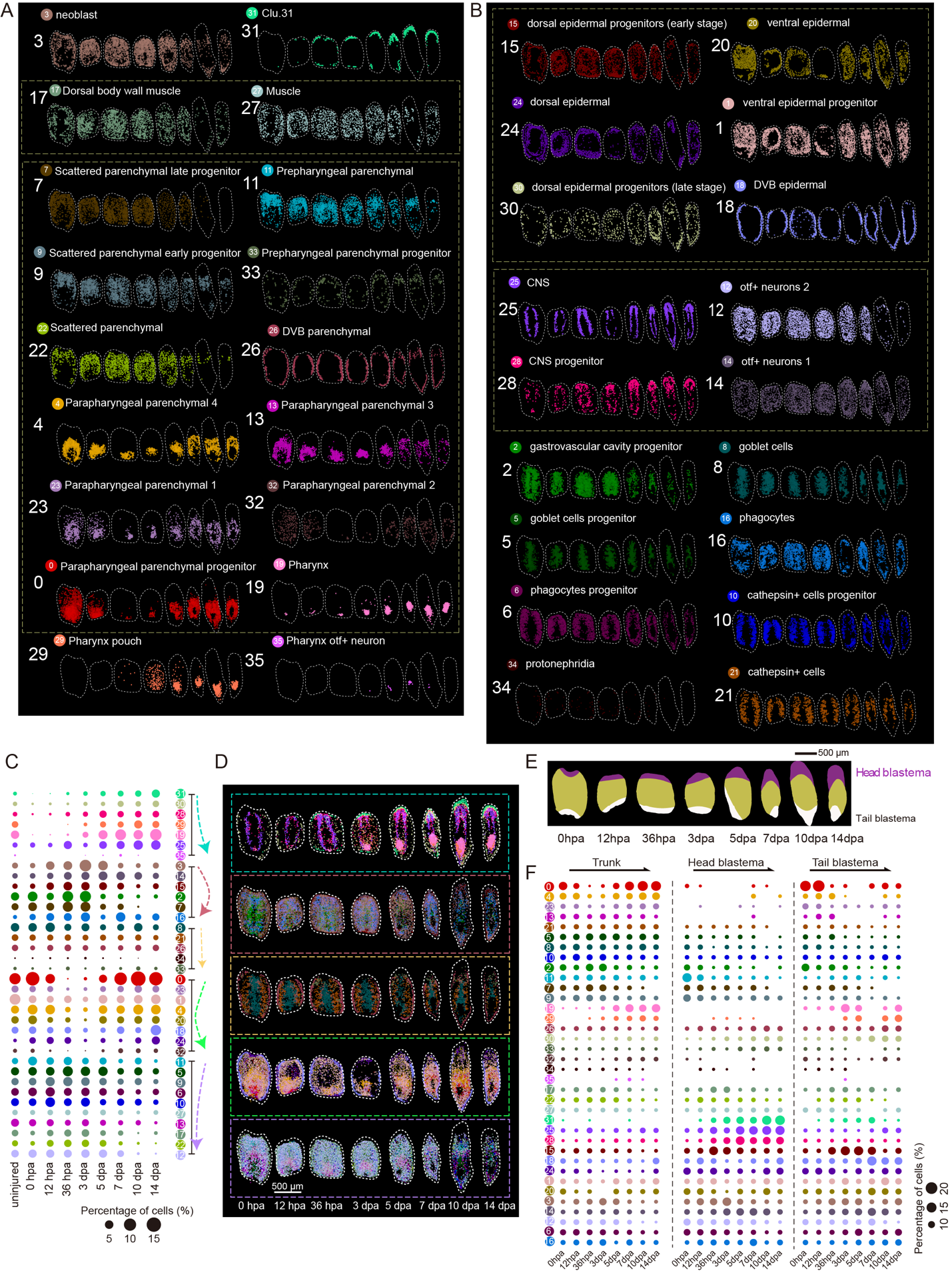


**Supplementary Figure S3: Characterization and spatial visualization of cellular populations across regenerative time points. (A)** Spatial visualization of 18 spatial transcriptomic clusters across eight regenerative time points, encompassing neoblasts, the Clu.31 regenerative domain, muscle lineages (two subtypes), parenchymal cells (11 subtypes), and pharyngeal cells (three subtypes). **(B)** Spatial visualization of 18 spatial transcriptomic clusters across eight regenerative time points, comprising epidermal cells (six subtypes), neural lineages (four subtypes), intestinal cells (five subtypes), cathepsin-expressing cells (two subtypes), and protonephridial lineages. **(C)** Bubble plots delineating proportional changes in cluster cell ratios between uninjured states and the eight regenerative time points. Clusters are categorized by population dynamics, with overall trends denoted by arrows. Corresponding cluster identifiers are provided on the right. **(D)** Three-dimensional projections of spatially categorized clusters across the eight regenerative time points. Cluster groupings correspond to the respective bubble plots. Scale bar, 500 µm. **(E)** Schematic representation of the head blastema (purple), trunk (brown), and tail blastema (white) domains in regenerating specimens. Blastema boundaries are defined based on the absence of pigmentation. Scale bar, 500 µm. **(F)** Bubble plot quantifying the temporal shifts in cluster proportions across eight regenerative time points, specifically within the trunk, head blastema, and tail blastema compartments.

**
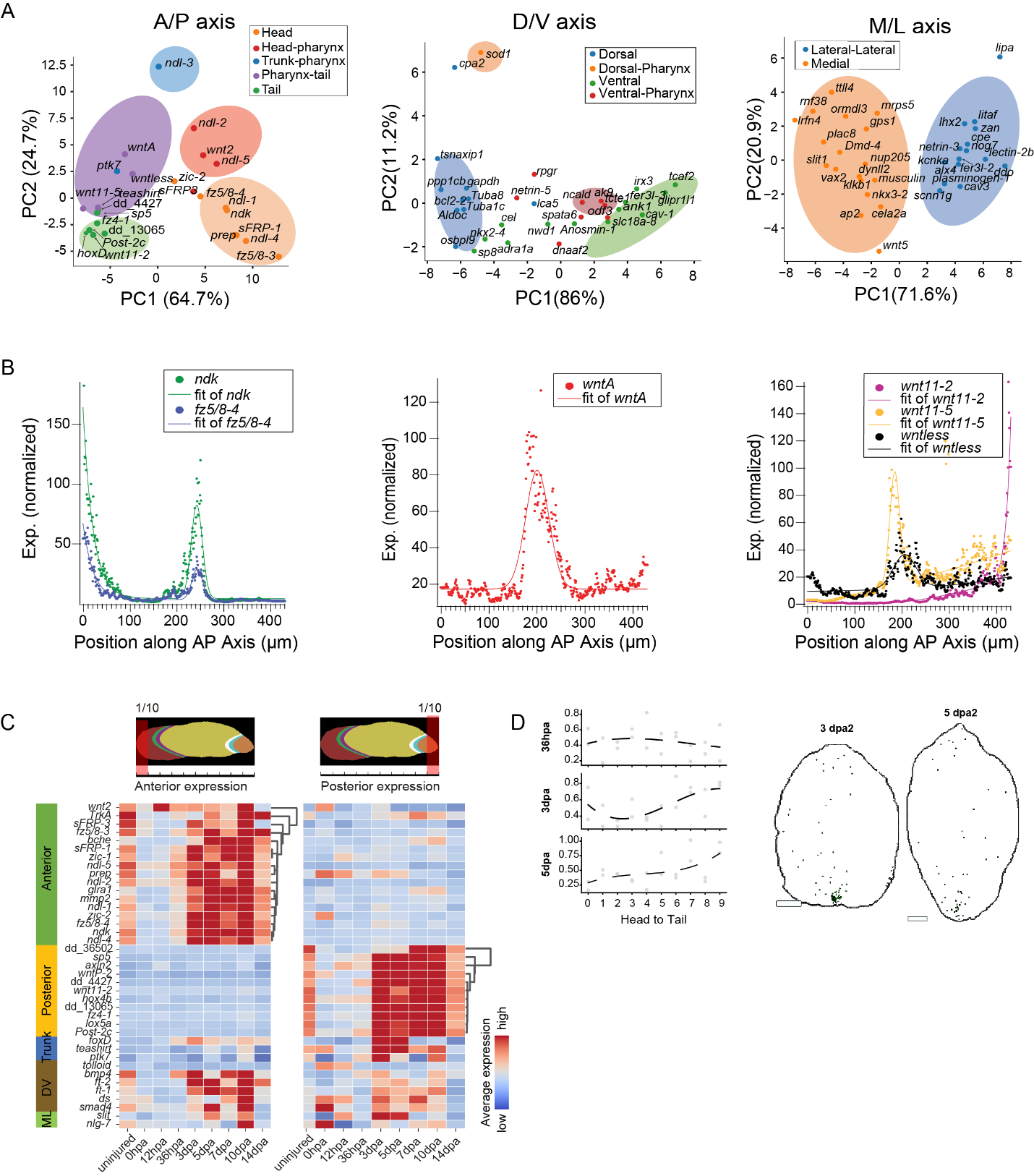
**

**Supplementary Figure S4: Self-organized morphogenetic gradient formation during polarity regeneration. (A)** Principal component analysis of gene expression patterns for anterior-posterior, medio-lateral, and dorso-ventral position control genes or candidates in intact animals. The proportion of variance explained by the principal components is detailed for each respective anatomical axis. **(B)** Exponential function fitting plots of spatial expression patterns for anterior-enriched, pharynx-enriched (incorporating an optional Gaussian fit), and posterior-enriched position control genes in intact planarians. **(C)** Heatmaps detailing the dynamic expression levels of specified genes localized to the anterior or posterior body extremities (defined as one-tenth of the total body length) throughout regeneration. Regionally enriched genes exhibiting analogous temporal patterns are organized via hierarchical clustering. **(D)** Spatiotemporal expression dynamics of the posterior position control gene wnt1 in regenerating planarians. The left panel demonstrates the relative expression distribution of wnt1 along the anterior-posterior axis at 36 hours post-amputation, 3 dpa, and 5dpa. Biological replicates were partitioned into ten longitudinal bins, with average expression levels calculated per bin. The emerging peaks at the posterior terminus confirm the correct polarization of wnt1 expression during tail regeneration. The right panel provides spatial visualization of wnt1 expression mapped onto the morphological contour of representative individuals at 3 and 5 dpa. The expression remains strictly confined to the posterior midline and tail tip, corroborating the established regulatory role of wnt1 in posterior polarity determination.


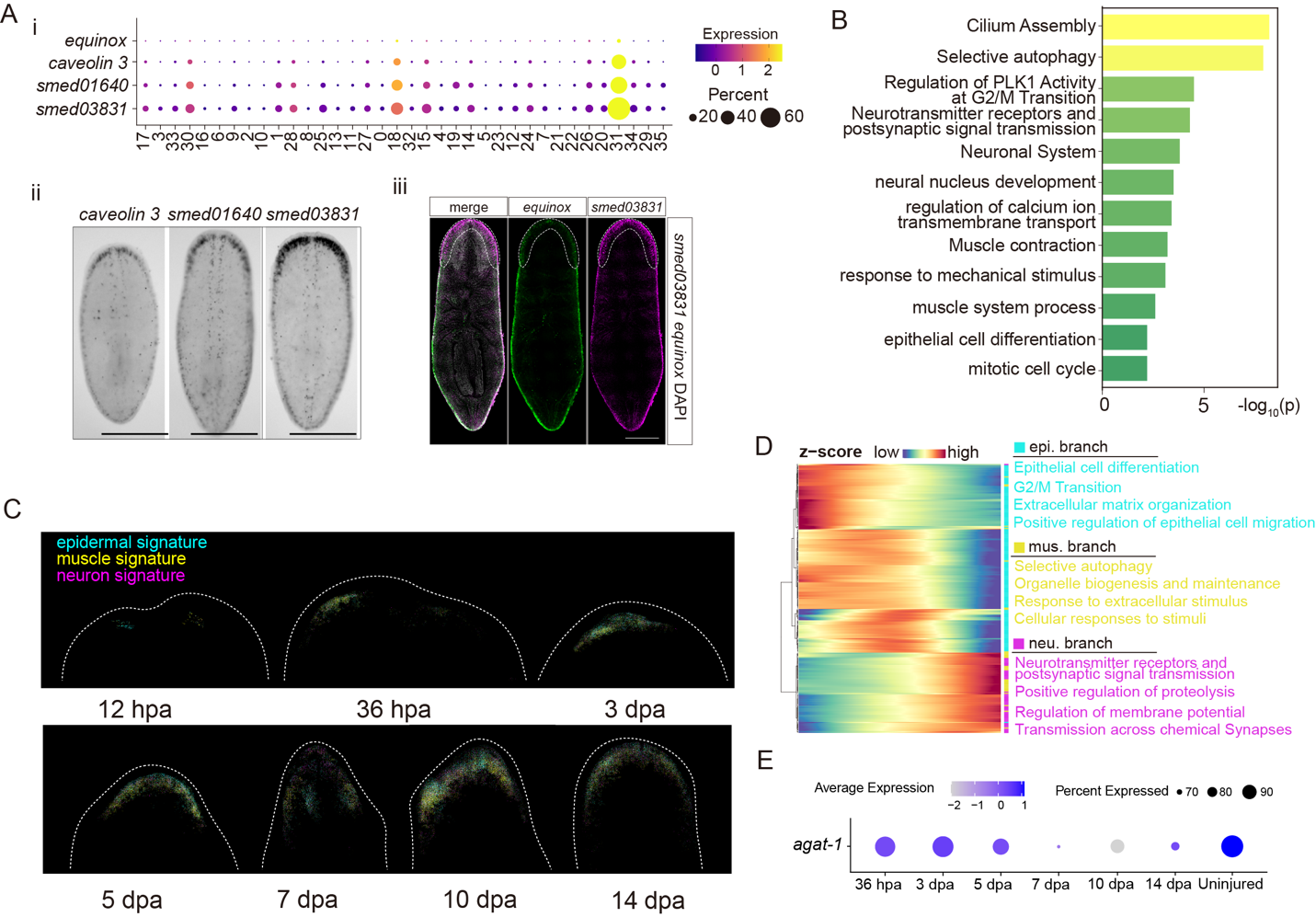


**Supplementary Figure S5: Composition and molecular signatures of the Clu.31 domain. (A)** Molecular characterization of the Clu.31 regenerative domain. The panel features a bubble plot illustrating the expression of the established wound epidermis marker equinox alongside the top three Clu.31-enriched marker genes across all transcriptomic clusters. Additionally, whole-mount in situ hybridization images localize three Clu.31 markers in homeostatic animals, accompanied by double fluorescence in situ hybridization of a representative Clu.31 marker (smed03831) mapped against equinox. Scale bars, 500 μm. n ≥ 3. **(B)** Functional enrichment analysis of Clu.31-enriched transcripts in homeostatic animals. **(C)** Spatial visualization of three constituent lineages residing within the Clu.31 domain at the head blastema across distinct regenerative time points. **(D)** Heatmap profiling enriched gene expression along the inferred Clu.31 developmental trajectory. Functional enrichment terms corresponding to genes associated with each pseudotime branch are detailed on the right. **(E)** Dot plot tracking the temporal expression profile of agat-1 throughout the regenerative process.


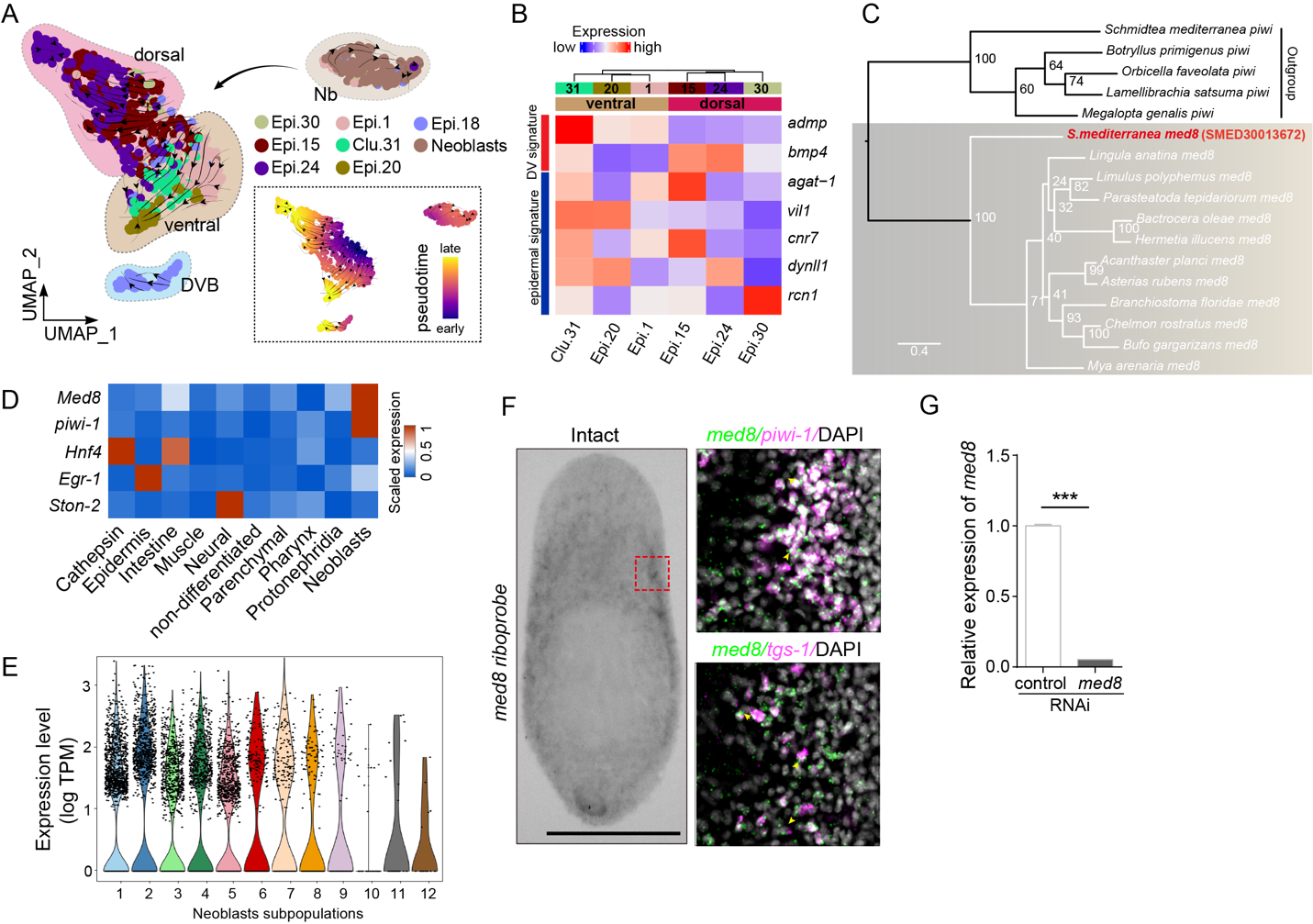


**Supplementary Figure S6: Transcriptomic characterization of the Clu.31 domain and expression profiling of med8. (A)** RNA velocity streamline plot depicting the predicted cellular transition trajectories at 36 hours post-amputation, coinciding with the initial emergence of the Clu.31 domain. Directional arrows indicate that Clu.31 predominantly originates from the ventral epidermal progenitor population (Cluster 1). The inset displays the inferred pseudotime progression, represented by a sequential color scale. **(B)** Heatmap comparing the relative expression of epidermal signature genes alongside dorsal (bmp4) and ventral (admp) positional markers within Clu.31 and selected epidermal clusters. **(C)** Phylogenetic tree of Mediator 8 family proteins, utilizing PIWI-1 as the defined outgroup. **(D)** Heatmap quantifying med8 expression across distinct cell populations, utilizing reference data from Benham-Pyle et al. **(E)** Violin plot detailing med8 expression variance across diverse neoblast subpopulations, referencing data from Zeng et al. **(F)** Whole-mount in situ hybridization demonstrating broad med8 expression and its spatial co-expression with the neoblast markers piwi-1 and tgs-1. Consistent expression patterns were observed across all biological replicates (n ≥ 6). Scale bar, 500 µm. **(G)** Quantitative real-time PCR assessment demonstrating med8 knockdown efficiency following RNA interference.


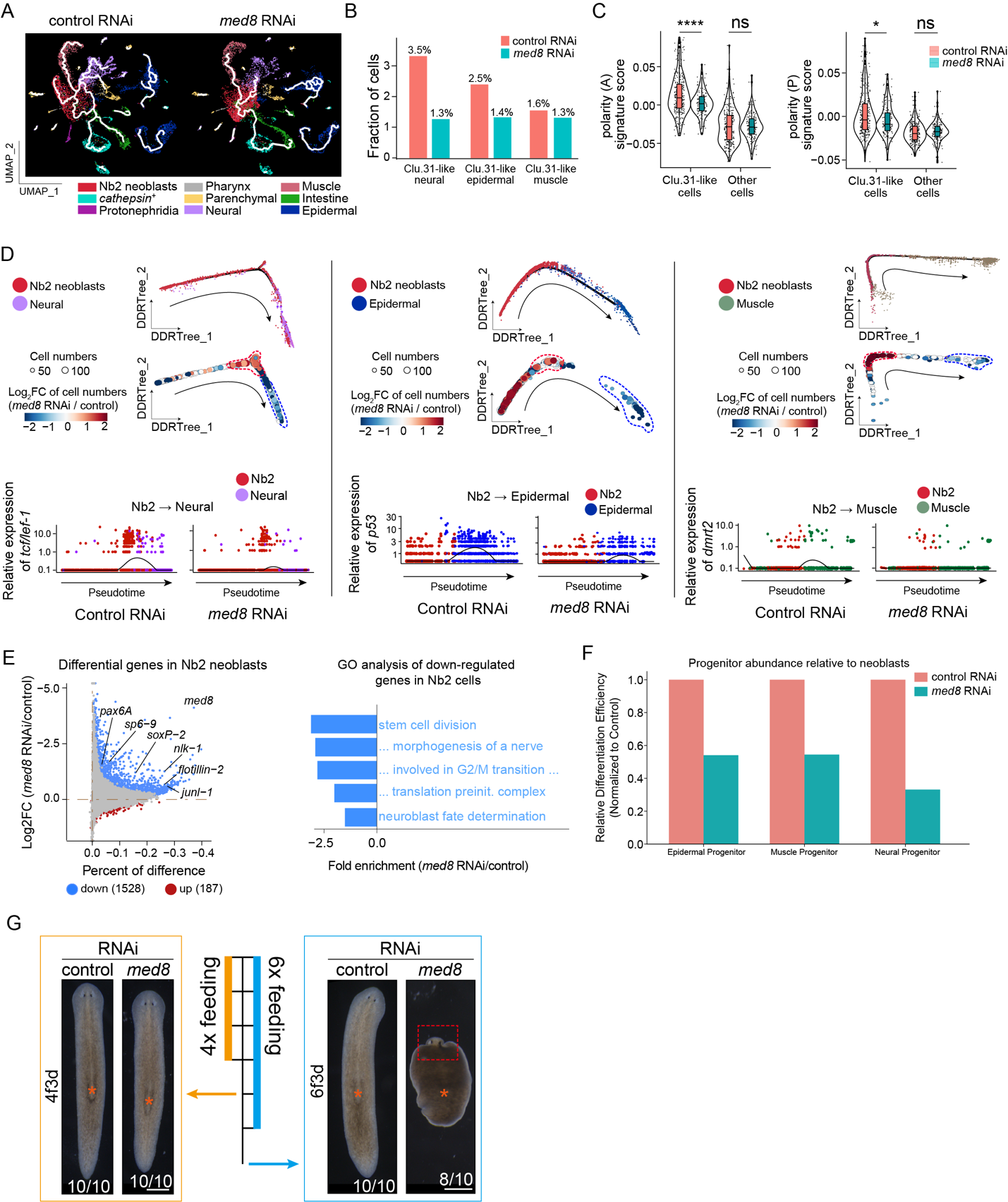


**Supplementary Figure S7: The *med8*-regulated Clu.31 domain modulates cellular production during blastema formation. (A)** Pseudotime trajectory analysis of single-cell RNA sequencing data revealing impaired differentiation of Nb2 neoblasts into subsequent cell lineages in med8 RNAi animals. Altered lineage trajectories are highlighted by dashed lines. **(B)** Bar plot comparing the proportional composition of lineage components within the Clu.31 domain between control and med8 RNAi specimens. **(C)** Violin plots quantifying the signature expression scores of anterior and posterior polarity genes. Statistical significance was determined using the Wilcoxon rank-sum test (ns, not significant; *, p < 0.05; ****, p < 0.0001). **(D)** Comprehensive differentiation analysis incorporating Monocle2 pseudotime trajectories and miloR differential cell abundance mapping for predicted transitions from Nb2 neoblasts toward neural, epidermal, and muscle lineages. Corresponding scatter plots depict the relative expression dynamics of *tcf/lef-1*, *p53*, and *dmrt2* along these inferred developmental paths. **(E)** Volcano plot identifying differentially expressed genes within the Nb2 neoblast population following med8 knockdown, accompanied by representative Gene Ontology enrichment terms for the significantly downregulated gene set. **(F)** Bar plot quantifying the reduction in differentiation efficiency across epidermal, muscle, and neural progenitor lineages. **(G)** Representative morphological phenotypes of med8 RNAi animals following four or six consecutive feedings, assessed three days post-final feeding. Scale bars, 500 µm.


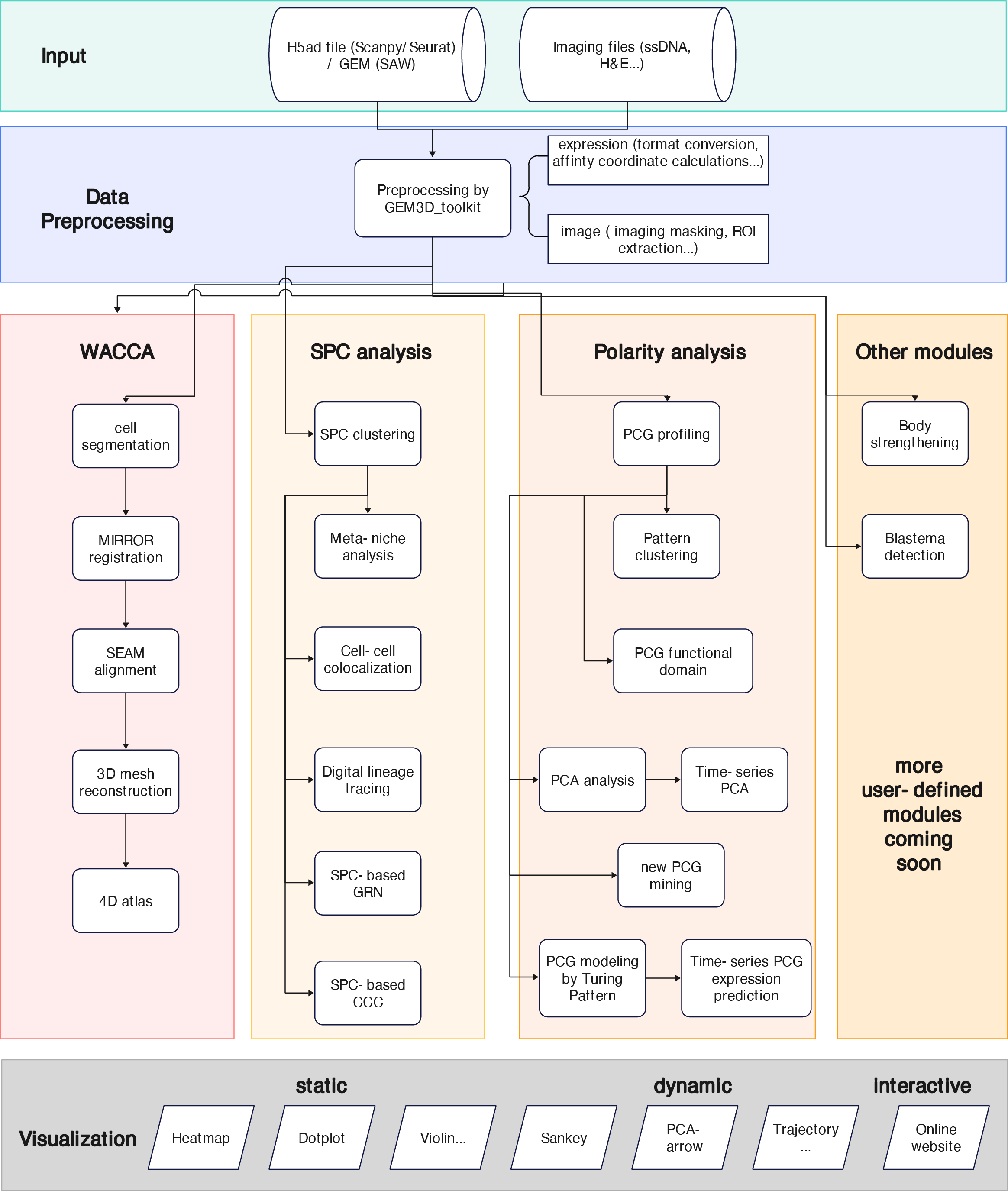


**Supplementary Figure S8:** **Schematic overview of the computational workflow.** The diagram delineates the sequential data processing pipeline, commencing with raw data input from h5ad and imaging files. This is followed by spatial preprocessing via the GEM3D toolkit and branches into parallel analytical modules. These subsequent modules include the WACCA algorithm for robust three-dimensional reconstruction, spatial transcriptomic analysis for cellular clustering, and positional analysis for modeling dynamic morphogenetic gradients.

**FISH probe sequences**

**Collagen(SMED30017945):**

TGGCCGACCTTTCCAACAGGACCTTGAGGACCTACAATACCAGCCTCACCTCTTACACCCGGCCTTCCATCAACTCCTGGATATCCCGGAGGTCCCAAAGAACCAGATGGGCCAGTCTTTCCTGCTAGACCTGGATTTCCAGAGTCTCCTTTAGCACCTTTGATTCCACTCTCACCCCTGATTCCTTTGTTTCCGATTTCACCGGGGATTCCCGATTGCCCCGGAGGTCCAATGGCACCTTGTGGTCCAAGTGATCCAGGTACCCCATCAGTGCCTTGTTTTCCCCGCTCACCGGTCGGGCCTTGTATACCTGCAGGACCAACTCGACCAGCAGGTCCTCTTAAACCTTGTGGACCCTGTAAACCAGGTTCCCCATTTGATCCTTTCAATCCAGCTAATCCAATGTCCCCTTTATCTCCGGTTAAACCTCGATTACCTTGAGGTCCAGTTCCTCCTTCTGGTCCAATGGAACCAGCTAATCCTGGTTCACCGCGAGGACCGATAGGTCCAACAACGCCATGACTACCAGGAGTACCATCTTTTCCTGGAATACCTGATGCTCCTGGTTTTCCTGATTTTCCTCTGGCACCATCTCTTCCTGGAGGCCCAATAGGTCCTAATGCTCCCGGATCTCCAGGAGGTCCAGGTTTTCCCATAGGACCAGGATCACCTCTAGGACCTTGGCTTCCTTTCGCTTGATTCGGAGATGTTGCTTGCCTTTTCAATCTGTTTAACTTTTCTTCTGCTCTGGCATTAATCGAGACGCATAGAACAAACAATAGAGAGAAAAAGATAGAATTCCACATTTCCCAAATAGTAATTTATCCTACGAACTGGAATGTTTAAAATTCCAAATTTTATCAACGTCACTAAGAAGTTATCCTTTGAACAGTCTATGATTTTCAATATCGAATTTTATCAAAATCGACGAGAAAATTCGATTGGAATAAATGAATTTTATTGGAATAGAAGCAATTTTACAACTACAATTTAAATTCGATACATACAGTTTCGCTTTATCCCATGCCATCCGCAG

**Agat1(SMED30018353):**

ATTGACACGATGTAAGCAGTGTATTTTATTACAAATAATTAATATACGTTGGAAAAGTTATGTTCTTTATATTTTCAATTGTGATATTCAGTGAGTTTCAAATTTCTCCAAGATTTCTTGAGGCCAGTCAAAATAGTTTTGAAGGTCTCCTCGCCTTCTAACATCCGATGTCCAGCAATGAATACCACCTCCTATGGAAAAGCAGTGTTTGAATGGAACTTTTACAGGAGTAATGCCACACTTTTCAAACAGTTTTTGAATTCCAATTTCATCTTTCTCTACGAGAACTCGGTTTTCATCCAACATGACAACATTAAGAGACAACCATTGAGAGCTGATATAGTATGGGAAATTCTTTGCAATATCTGGATAAGGAGCAACGACAACATCCCAGCCGGCTTTTTTAAGCATACCAATTTGTAAGCATGGTCTTTCTGGGCATGTTATCGCGAGGCCTGGCCGAGGTGTGAAAAGTGTCGTGTCAATATGCATTGGTTTAGGGTCAACGAAATTCATTGAATGAACTCTAATTCCTTTCGGTGCCAAATGTCTACGTACCCATTCAATTCCTGCATTGTTGCTAACCTGACTGACATGCGCAAATATATCCCTACCACATCGAATGAAATCTGCGGCGTCGAAAACTGGTTCCTCTTCCGTTAGAATCGAAGTCTTTTCATCGAAAAATGCTGTTGAATCTTCGTCAGCACGATAATTTTCTTTAAAAAGTGTTTCTTTACACATCGGCTTTGGTGCCGCTGTCCATTTAGCGCCCCGGCTCCAGTAATCTTTAATTAATTTTTTATAGCACATAAATTCAAAATATCTCGATCTCCAAGTCATGGTGGACTCAATCATTTCATTTCCGACAATTAATAGAAAATCTCTCGGCATCGCACAGTCGACTCCATGGGCAGTAAAGTTTGGAGTACAGAGCGTTTTAGCGAAATCAATCGGTTCTGGATGGACGACTTTAACTCCTTCTCCTTGTAGAATCTTTATGAAGTTCTTGTGCTCATCTAGCATTATTTTGTAGTGTTCTGGTTCAATTACATCCTTCCAATATTTTCCTTTGTGTTTTAAAAGGAAATCAAATTTGTTTTCCGAAATGCAAGCTTTGGCTTCTGGTAAAATCTCTGGACAACATGCCATATCTGGAAGTCCCAAAACGATTTCTTCTAATGGGTCCCATTCATTCCAGGCCCAAACCGGGCTGTGTTTTTTGTTACCAGGTGTATACGAGCATAACCTAGCTGACAATTTGTTGACAACGTTTATGGAAATCTGATCGTGCAGAGATCTAGCAATTTTGGTAAGCATTTTTGTACAATTTTTATCACAGAAAACCGGTGGAAATCC

**Piwi-1(SMED30007406):**

ACACAATGGACAGTATATCTTCATGTCGATCCAATGAAGTTGAAGACGCTTTAACAGATTTTATTCGTGTAAGCAAGGTACATATGGCATTAGTGTTCATTCCTGATGATAAAGTTTACGCAAAAGTAAAGAATTTTACAATGTCTACAGGTCTATTGACTCAGTGTGTGACGCAGAGAAACGGAAGTAATAGAGATGATCGACGTCGTAAAACGGTTGCAGATAAATCAGTTATGCAAATGTTTTCTAAATTAGGATACGACCCATGGGGTATTAATCTTAAAATGGCACCGACTATGATTGTAGGATTGGATACTTTTCATAGCAAAACCGGTAAAAGATCTGTTCAAGCATCCGTATTTTCAATTAGTGCCAAATTTTCTCAGTATATCAGTTTTGTCAATTCATCTAAAGGTAAAAATGAATTTCACGAAAATTTGGGCAAAAACTTCCTAACGGCCCTTACAACATTCCAAAACAAATTTAACACTATGCCTCTTCGTTTGATTATTTATCGTGACGGTGTTGGAGATTCACAGTTAGCATTTACCAAAAAGTTCGAAACTGATGCTGTTATGAAAATGATAGAGAAAATTTACGAAAATCAAACTTTGCCCCAAATTATTTATGTTGTGGTTAAGAAGAGAATTAGTGTTAAATTTTTCAAAGATGGGGCTAATCCAAATCCTGGTACTGTGGTTGATGAGAAAATAGTGAAACCCAATTTCTATGAATTTTACTTGGTGTCTCAGAAAACAACTAAAGGTACAGCATCTCCTACGAATTATAATGTTCTTATGGACACTAAGTTTACAAACAAGAAAACAAATGAGGTTTCAGTTATGTCTCCGAGTGTATTGCAGCAAATAACTTATTCGTTAACACATTTGTATTTTAACTGGATGGGAACAATCCGAGTTCCTGTTCCAACGCATTATGCCCATCGTTTGGCTGAACTCGTTGGCAAGATTCATCGTGGTGTTACTCCTCCAGCGATAAATGACAGAATTCGCGAACGCTTATT

**Pc2(SMED30010096):**

AGAATCGGCAAATAAAGTAGCTCAGGAAACTGGTTTCTCGGTCTTACGAAACCATCTGAATTCTCCAAATGAATTTGTTTTCCATCACAAAACCGTTCCTCACGTTAGTTCAGTTCCAAGTTGGAGTCAACACAAAGTTCTATTGGCTCACCATCTTGTTAAAAACGCAAAACAAGTTGAAGGGTTTTTGAGACGAAAGAGAGGCTATAAGCCAATTAAAAAACAAGGTCGTGTCCCACTGCCAACGATTAATAAAAAAGGAATTCCCGGCCTGGGAGAGTCTGACAACATTAAAGTGGATTTGAAATTACCTACTGATCCTCTATTTTCTAAAGAATGGTACATCAGAAATACCGGTCAAGCTGATGGAGTGAAAGATCTTGATCTTAATGTATTATCAGCGTGGGCTCAAGGTATAACCGGCAAAGGGGTCACGACTGCAATCATGGATGATGGAATAGACTATCTTCACCCGGA

**Pds (SMED30027819):**

TTGAACAGTTCCTGAATCGACATCAATGACGCCTCATCACAGAGAATGAAACACTGCAGTAAAGCTTTCATTCGATTGCCCGAATCGAAATTATACGGAACGAGAAATGTGTTAAATAGCCGCTCTACGACAATTTTGTCTTCGACTTCAGTCCTAAGATAGAGCCGCAATATTTGAGTGAGTAATAACTCCAAGCGAGTTCGTTGTCCGGGTAGTAGATTTCCTAAGTCTAGAATCGTATCCACGTTTCTATTGGAATCTCCAGCACGTGATTCCGATTCCGAAAGGATTCTACGATACAATTTCCCTAAACTGGCATTTGCTTCCTTCCGAACGACATTACACTTATCTCGAGTTCTGTCGACGAGTATTTTAAAAAGCGTCTCATTAACGAAATCGAGTTCCTCATCGACAATAATGGATTTGCTTTTTTCAAATATTTGCGAAATAGAAAGAACCAATTGAAATCTAATACTTTCTTCTTGATCATGAGACCGTTTTTGGAGAGCTCTAACTAAATCCTCTTTCGATGTTGGAATGGATAATATTCCGGAAATATCATCTCCGGTGATAGCTCGTCTCAAAACGTCTCTAGCAACACGCACACAAATTTCACGTACTTCTACTTCAATGTCACAAAACCTACCAAGAAAACACTCCCACAATGATGAGTGATTGCTGTTATTACTTGGGATATAAGAGATAAATAGTTTTCCCAGCAATCGACACGCACGTTTACGATCTGTGACATTATTTGCTGACTTCAATTTCATTTCGATTGTCGATAAGAGAGGAATCACAATTGCGGCGCTAATTTTGAAAAGTTCATAGACCAATTGGAATGTGTGATCACAGGAGTATTCAATGCACGTCTCAATTGTATTCTTGTTCTTTTCTAAC

**Wnt-1 (SMED30008884):**

CTATCTTCTTTGACGACCTTTACGGCATTTTCATATTTTTCTTTAAGAATATTACCGATTTCGTCAATACTAGCAACCTTTCTGTGGCAAATTTTGGTCGTACAAGATCCACTTGTGCCCTGGCATACACATTTCACGTTCATACTTTTGACTGCAACTCTTCTACCCGTTCCCGTGTTGTGAAGGTTCATTAATGTCTTTTTATTCCAATCTGGAATGTCTGGATCAAACATTTTTCTCGCAAATCTTCGACCGAATTGTACGTTGTCGTCACATCCTTGCCAAATCCAGTTGGTTTGAGATATCCGTCCTTTATTATTGCACGGACAGTGTGATAACTTAAACGAACAAGCCTCAGCGACAGTTTGAGCGACACTCGCACTAAGCATCGCATAAATGAAAGCGGTTTCGGGAAATCCTTTGAGCATAATGTCACCAAAAAGTAAGGCGGATGGATTATTCAAATTCGGTGTCGGACAGTTCCAGCGATGATTGGCAAACAACTTTTGACATGTGTAAATACCCTTTCTGATTCCTTCAATAGCCACTTGAATTAGATCACTGTTAGCAGGCAAATCGGTAAAGTTTTTCCAATTGGGTCTGTACTCTTGAGAATAAAGCTTACGTCTAATTGGATCAAAACTGTAAAATTGATTCCATTGGTGTCTGGTGAAAGCATATTGAAGGCTGTTAGTTGCAATATTTGGATCACTTTTCATTTTATATAAATGCTGACGTGCTTGATAAGGGAAATAGGATCTGTCAGCTGGGAGAGACTGATATTTGATTATTGGCAGGCCTGGCCCAGCAAAATACGTTGATTAAGTCGGGTAGCTGTCTCAACTCAGTCATGTTGACTACACAGTGATTGACAGTTATTTCGACCTTGGCGATTTCAGG

**Wnt11-1(SMED30007710):** TACAGCTTCCACTAACTCCATGGCATTTGCATTTAGATTTCCAACTATTCATCATTATCATAATTCCGACTTCATAATTGTGTCCATTTAATCTCTTTATGATTTGACTTTTGTTTTGTTTATTTGGTTTTTTATTCTCAGATTTTGAATCAGAGTCTTTGGATTTTATTCCCATTTGATGGAATCCCAAGAAGTTTCTCGTGGACTCTATTCCAATATCAATGTTATCAAGACAATTGCTATAATAATATGTATAATTTTGATTGTTTGATCCAACATTCGCATCTGACTCATCTTTTCCACAAGAGCAATGTCTCAATTTGTTTTGTGAACATCTTCTAGAAATTTCAAACACCAACGCAGCGCTACTAAAAGCATGAACAACGGCCTGCTCTTGAGTTCCCAACTTCAAATCAGGAGGTAGTTTCGGTAATTTTTCCACAGATTTACAATTCCATCTCCTGTCAGCAAAGGTTTTCATACAATAATAAACCGTGGCCTTTGAAGCCATCAAAACGGCATGCATAGCAAACCGATGTTTATGATGCAAACAAAACCTAAAATGCCTTTTTCGAAGAACTTGAATTTTCCTTGCGTATTCACATTCAGATCTCGTAAATCCATCCAATTCCTGTTTGTGGCTTCGGTTTAATTTGGCAAGACCCAGCCATTTGATTCCTGAATTGTAATTGATAAGAATAACAAACATCAAAAGGTTTCCAATCAAATCGGGAAAAATTCTCATTCTGTTTTATTTTCTGTTTTTTTATTTTTCTTTTCTTCTTTATTTTTTTTCTCTCTCTCTGTCTGTCTATTGGAAACCAAAATGGTAAAAATTGAAAACCATGTAACCAAAAAAAAATTAAAAAAGGCAAAAAAATATATATCGGTTCGATAAAA

**Equinox(SMED30032931):** GAGCCAGAGAAAGATTGCGGACAATGGCATTTGTTTTTCCCAATACAGGTTCCTCCATTCATACATTGCGGATAGCATTCATATTTTTCACACCGATTTCCAACAGTGCCTTCAGGACAGGAACAAATATTTCCTTTTTGGCAAATCCCTCCCTTTTGACATGGGGGATTGCATATCCTTTTTTCACAGAATTTTCCTATATAACCAAATGCACAAGTGCATAAATTTGGACGCAAGCACTGTCCGCCATTTTCGCAAGTAATATCGCATTTCAATTTTTCACAAAATAAACCTTCAAAACCTTCTGAACATTGACAAATATTTGGCCCTATACATTTACCCCCATTTTTACATTTTTCATTGCAAATTGGGTTTTGACAAAACTTGCCTTGATATCCGGTTGGACAAAAACATTTCCATTTCACACATTGCCCATTATTTTGACAGGTTATATTGCAAGATTCGTTAGGACACCGTTTTTGAATGAAACAGCTTCTAATTTCGGATTTTATATTCTCTTTTTCTAAGCATTCATCATCTTGACAAGGCCGATAACGTTTTTGCCATCCAATCCCGCAAGTTACAGAACACGTTGACCAACTTGATCTCGGCGACACTGTTGACAATGGAATTGACAGCTGCTGGATTTTGCTGGCATTTAGCACATTTGATGAGTGATCATAGAAAACATTGGAACAGACTGTATTTTTAAAAGTTTCTAGTGAATCTGTGGACACTCGAATGATAACATTTTTCGACACTTTTAAATGTAACTTTTGAACTGAACCTGAAAAGAACTTTCTAATTTTTTCATTATAGATCGTAAACATTCTAAATATACAAAATGTTTCCACAAATATTCCAGAATCGTTGATGAAAACATTGAAAGAAAGATCTTCA

**Caveolin-3(SMED30000881):** TGGCAAACTAATGAGGGTAACAAGTTCACAAAAATATCTAATGAGCTTCTGCACATCAAGCAAAATCTAGTTTCCAGTTAAATGGCTAGTTCATCTCAACCAAATTATAAGTATCATTTTTGAGTTACTTGAATGTTGGAAAAGCAAGCTCCACAGGCTCTGAAATATGGCTCACAACATAGCCCAGTAATAGAATCCATTAGTGATTTGAATATTGCGCAGTGCAGCCCATAAACTTTACACATTGGAGTTAAACACCAAATGTACTCAAAGGCCACACATGCAAATACGCAACCCCAACAAAGACCAAGACAAAGACCGCAAAGTGTTGTCAAAAACTTATAACAACATCCGATTCCAAATTTATAACAACAATATGATGTACGCCAAACACAGTCAATTGAATGGGCTCCTTCTGGCTCTGCTAGAATGTCTTCAAATGTCACTTTCAAATGATTATTAATATTCCTAGGATCTCTATTTTCTAAATCTAAGTCTGCCATCGATAAATTCGCAATCAAAAAATAAACAATTGACGCTGAAATATCACATTATATAGTAG

**Med8(SMED30013672):**

GATGATCGAAACTTAATATTAACAATTTAAAACTAAATCAAACAGTTTCCTAAACATAATAACATTCACCAAATAGTTTGAAATAACCATATACAGGTAATAAAAATAACAAGTTAAAATATTATTTCGATAATTTTAATCCTTTCCCATTTGACATGCAATTAAATAGTTCCTGAATCTCAGTCATTGTATTTTGCTTAATTGCTTTTTCAGTATTATCTACATCAATTTGATTTTTGAGAGAGGATAATTCTTTCAACGCACTATCAACAACTTTGTTTGACGCGTTTTGTTTAGAAACAATCTCAGCAGACAAGTTACTAGCTTTTATCAACTGTTCTTTTCTAGCATGTTGAACTTTTGGATCCAGCAATGTGCGCAGATATTGCGGTGGTACTTCATAAGTAAACGCTTTGATTCTATCTTGAGTTAAATTAATAAGATTCTGGTCAGGGTTAGGATTTAATGCCACTGGGATCATAACTAAATTATCTAATTGCAAATTTTTATCTGTCAGCATAGTTTTAACAATGTTATTTAATTCGGTGGCCAATAATCCGAATATATTTAAATATTTGGACCATTCATGATTTGAAATTCTAAACAACTCCGTAATTGAACAACTTTTTCACGAAATTTAAACAGAGAAAGTATTAATGGATCTAAATCGCATTCTTTGTTCATTTCAATGTAAAAAAAT

**Rod1(smed03831):**

GAAATACAAATTGAATATATTATTATGACAAACAATAACAGAGAGAAACAAAAGGAAAATCGCAACGACAACAAAAGTACATCTAATAATAATAACTAAGTGCAATATCAATAGTGTTCAAGGATTTTCCCATTGAGAACTATTTTAGAAATTCGCAACAAATGAAATCGTTTGATTAGTATCATCTCCAGAACTCATGGACATTTGTTGATTAGCCGTTTCAGATTGTTGTTTTACTTGGTAATTCATTACGTAACTTGTTTCCACATGACGATTTTCACTATTATCAATTGTTTGGATTTCATTTTCGTGACTTTCCGATTTAGTTGTTGACTTTTCATTAGTACCAGAGCCAGCCATTTCGGAAGTTTTTTTGATATCTGACATCTTTTGAATTGAGTAGAAATTATAACATATAAACACAGCAACAATTTGAAATAATAGCAAAT

**smed01640:**

ACAACTGACATATCATCAATATGCACGTAATAAACTCGCCAAGACCGGCAATCCTATCACAACTGCTATTGGAACTTTGAATTCACTTCCGGTGTTGCAGAGATTTGTAGAGCAGCACGTATTGGATGTTCCAGCTATTCCTGAGATTGTAACACAAGTTCCAGTGATGCATGTTTTTGAGATGACAGTCAGAGCTGTCGTTGTCATGCATGATGAACAATTGGGGGAAGTCATAGCACCGGAAATGACAAACGGATTTGGGCATCCAATGCAAACATAACAATTAATCAATTGAGCATGACCAAGTTGAAGATCAGTTATTGCAATCACAAAAAACAACAGCAACTTTGAAATCATATTTATGTTAATAATATTTTGTGATTGTGTTTTATAAAGCTAAAAATTACAAACAAATAAAATATGTTTCTTCTCATTCAACTCTGACAAAGAACAATTCCATTAAATAAATATTTATCATTCAATGACTCATGCTTACAGTTTCCAATGGGTACGCTTCCCCATCGCATTTCCCAGCGTTTCCAGG

**RNAi data:**

**Med8(SMED30013672):**

Target regions:

ATTTTTTTACATTGAAATGAACAAAGAATGCGATTTAGATCCATTAATACTTTCTCTGTTTAAATTTCGTGAAAAAGTTGTTCAATTACGGAGTTGTTTAGAATTTCAAATCATGAATGGTCCAAATATTTAAATATATTCGGATTATTGGCCACCGAATTAAATAACATTGTTAAAACTATGCTGACAGATAAAAATTTGCAATTAGATAATTTAGTTATGATCCCAGTGGCATTAAATCCTAACCCTGACCAGAATCTTATTAATTTAACTCAAGATAGAATCAAAGCGTTTACTTATGAAGTACCACCGCAATATCTGCGCACATTGCTGGATCCAAAAGTTCAACATGCTAGAAAAGAACAGTTGATAAAAGCTAGTAACTTGTCTGCTGAGATTGTTTCTAAACAAAACGCGTCAAACAAAGTTGTTGATAGTGCGTTGAAAGAATTATCCTCTCTCAAAAATCAAATTGATGTAGATAATACTGAAAAAGCAATTAAGCAAAATACAATGACTGAGATTCAGGAACTATTTAATTGCATGTCAAATGGGAAAGGATTAAAATTATCGAAATAATATTTTAACTTGTTATTTTTATTACCTGTATATGGTTATTTCAAACTATTTGGTGAATGTTATTATGTTTAGGAAACTGTTTGATTTAGTTTTAAATTGTTAATATTAAGTTTCGATCATC

RNAi constructs:

GATGATCGAAACTTAATATTAACAATTTAAAACTAAATCAAACAGTTTCCTAAACATAATAACATTCACCAAATAGTTTGAAATAACCATATACAGGTAATAAAAATAACAAGTTAAAATATTATTTCGATAATTTTAATCCTTTCCCATTTGACATGCAATTAAATAGTTCCTGAATCTCAGTCATTGTATTTTGCTTAATTGCTTTTTCAGTATTATCTACATCAATTTGATTTTTGAGAGAGGATAATTCTTTCAACGCACTATCAACAACTTTGTTTGACGCGTTTTGTTTAGAAACAATCTCAGCAGACAAGTTACTAGCTTTTATCAACTGTTCTTTTCTAGCATGTTGAACTTTTGGATCCAGCAATGTGCGCAGATATTGCGGTGGTACTTCATAAGTAAACGCTTTGATTCTATCTTGAGTTAAATTAATAAGATTCTGGTCAGGGTTAGGATTTAATGCCACTGGGATCATAACTAAATTATCTAATTGCAAATTTTTATCTGTCAGCATAGTTTTAACAATGTTATTTAATTCGGTGGCCAATAATCCGAATATATTTAAATATTTGGACCATTCATGATTTGAAATTCTAAACAACTCCGTAATTGAACAACTTTTTCACGAAATTTAAACAGAGAAAGTATTAATGGATCTAAATCGCATTCTTTGTTCATTTCAATGTAAAAAAAT

**Rod1(SMED30003831)：**

Target regions:

ATTTGCTATTATTTCAAATTGTTGCTGTGTTTATATGTTATAATTTCTACTCAATTCAAAAGATGTCAGATATCAAAAAAACTTCCGAAATGGCTGGCTCTGGTACTAATGAAAAGTCAACAACTAAATCGGAAAGTCACGAAAATGAAATCCAAACAATTGATAATAGTGAAAATCGTCATGTGGAAACAAGTTACGTAATGAATTACCAAGTAAAACAACAATCTGAAACGGCTAATCAACAAATGTCCATGAGTTCTGGAGATGATACTAATCAAACGATTTCATTTGTTGCGAATTTCTAAAATAGTTCTCAATGGGAAAATCCTTGAACACTATTGATATTGCACTTAGTTATTATTATTAGATGTACTTTTGTTGTCGTTGCGATTTTCCTTTTGTTTCTCTCTGTTATTGTTTGTCATAATAATATATTCAATTTGTATTTC

RNAi constructs:

GAAATACAAATTGAATATATTATTATGACAAACAATAACAGAGAGAAACAAAAGGAAAATCGCAACGACAACAAAAGTACATCTAATAATAATAACTAAGTGCAATATCAATAGTGTTCAAGGATTTTCCCATTGAGAACTATTTTAGAAATTCGCAACAAATGAAATCGTTTGATTAGTATCATCTCCAGAACTCATGGACATTTGTTGATTAGCCGTTTCAGATTGTTGTTTTACTTGGTAATTCATTACGTAACTTGTTTCCACATGACGATTTTCACTATTATCAATTGTTTGGATTTCATTTTCGTGACTTTCCGATTTAGTTGTTGACTTTTCATTAGTACCAGAGCCAGCCATTTCGGAAGTTTTTTTGATATCTGACATCTTTTGAATTGAGTAGAAATTATAACATATAAACACAGCAACAATTTGAAATAATAGCAAAT

**EGFP:**

Target regions : ATGGTCTCCAAGGGCGAGGAGCTGTTCACCGGCGTCGTCCCGATCCTGGTCGAGCTGGACGGCGACGTCAACGGCCACAAGTTCTCCGTCTCCGGCGAGGGCGAGGGCGACGCCACCTACGGCAAGCTGACCCTGAAGTTCATCTGCACCACCGGCAAGCTGCCGGTCCCGTGGCCGACCCTGGTCACCACCCTGACCTACGGCGTCCAGTGCTTCTCCCGCTACCCGGACCACATGAAGCAGCACGACTTCTTCAAGTCCGCCATGCCGGAGGGCTACGTCCAGGAGCGCACCATCTTCTTCAAGGACGACGGCAACTACAAGACCCGCGCCGAGGTCAAGTTCGAGGGCGACACCCTGGTCAACCGCATCGAGCTGAAGGGCATCGACTTCAAGGAGGACGGCAACATCCTGGGCCACAAGCTGGAGTACAACTACAACTCCCACAACGTCTACATCATGGCCGACAAGCAGAAGAACGGCATCAAGGTCAACTTCAAGATCCGCCACAACATCGAGGACGGCTCCGTCCAGCTGGCCGACCACTACCAGCAGAACACCCCGATCGGCGACGGCCCGGTCCTGCTGCCGGACAACCACTACCTGTCCACCCAGTCCGCCCTGTCCAAGGACCCGAACGAGAAGCGCGACCACATGGTCCTGCTGGAGTTCGTCACCGCCGCCGGCATCACCCTGGGCATGGACGAGCTGTACAAGTAA

RNAi constructs:

TTACTTGTACAGCTCGTCCATGCCCAGGGTGATGCCGGCGGCGGTGACGAACTCCAGCAGGACCATGTGGTCGCGCTTCTCGTTCGGGTCCTTGGACAGGGCGGACTGGGTGGACAGGTAGTGGTTGTCCGGCAGCAGGACCGGGCCGTCGCCGATCGGGGTGTTCTGCTGGTAGTGGTCGGCCAGCTGGACGGAGCCGTCCTCGATGTTGTGGCGGATCTTGAAGTTGACCTTGATGCCGTTCTTCTGCTTGTCGGCCATGATGTAGACGTTGTGGGAGTTGTAGTTGTACTCCAGCTTGTGGCCCAGGATGTTGCCGTCCTCCTTGAAGTCGATGCCCTTCAGCTCGATGCGGTTGACCAGGGTGTCGCCCTCGAACTTGACCTCGGCGCGGGTCTTGTAGTTGCCGTCGTCCTTGAAGAAGATGGTGCGCTCCTGGACGTAGCCCTCCGGCATGGCGGACTTGAAGAAGTCGTGCTGCTTCATGTGGTCCGGGTAGCGGGAGAAGCACTGGACGCCGTAGGTCAGGGTGGTGACCAGGGTCGGCCACGGGACCGGCAGCTTGCCGGTGGTGCAGATGAACTTCAGGGTCAGCTTGCCGTAGGTGGCGTCGCCCTCGCCCTCGCCGGAGACGGAGAACTTGTGGCCGTTGACGTCGCCGTCCAGCTCGACCAGGATCGGGACGACGCCGGTGAACAGCTCCTCGCCCTTGGAGACCAT
