# Supplementary material for: 4D single-cell spatial transcriptomics reveals dynamic morphogenetic gradients and regenerative domains in planarians
Source: Gigascience. 2026 May 22;15:giag064. doi: 10.1093/gigascience/giag064 (PMC13273413; doi:10.1093/gigascience/giag064)
Supplement: giag064_GIGA-D-25-00451_Revision_1 [file giag064_GIGA-D-25-00451_Revision_1.pdf]

GigaScience

4D Single-Cell Spatial Transcriptomics Reveals Dynamic Morphogenetic Gradients and Regenerative Domains in Planarians

--Manuscript Draft--

**Manuscript Number:**	GIGA-D-25-00451R1
**Full Title:**	4D Single-Cell Spatial Transcriptomics Reveals Dynamic Morphogenetic Gradients and Regenerative Domains in Planarians
**Article Type:**	Research

Funding Information:

National Key R&D Program of China (2022YFC3400400)	Dr. Xun Xu
National Key R&D Program of China (2020YFA0112502)	Dr. An Zeng
National Key R&D Program of China (2021YFA1100202)	Dr. An Zeng
National Natural Science Foundation of China (32070828)	Dr. An Zeng
Shenzhen Science and Technology Program (JCYJ20250604191305008)	Dr. Mengyang Xu
Shenzhen Science and Technology Program (RCJC20221008092804002)	Dr. Ying Gu
Strategic Priority Research Program of the Chinese Academy of Sciences (XDA16021300)	Dr. An Zeng
CAS Pioneer Hundred Talents Program	Dr. An Zeng
Shanghai Pujiang Program (20PJ1414600)	Dr. An Zeng
Shanghai Science and Technology Committee (STCSM) (22ZR1468400)	Dr. An Zeng
Guangdong Genomics Data Center (2021B1212100001)	Dr. Tao Yang
Feng Foundation of Biomedical Research	Dr. An Zeng

Abstract:

Background: Understanding how organisms reconstruct complex tissue architectures following injury requires precise mapping of gene expression and cellular responses across space and time. Although planarians serve as a classic model for whole-body regeneration, capturing the continuous spatiotemporal dynamics of positional information and cell fate decisions at the organismal scale remains a significant challenge.

Results: Using high-definition spatial transcriptomics, we generated a four-dimensional atlas encompassing over 3.5 million cells from whole animals across eight distinct regeneration timepoints. This comprehensive dataset enabled the definition of 36 spatial domains and the tracing of body axis restoration, revealing that positional control genes recover through self-organizing dynamics analogous to an underdamped control system. We identified a transient injury-induced spatial domain termed the Anterior Regenerative Zone. This unique region is characterized by the convergence of epidermal, muscular, and neural lineages enriched with positional signals. Furthermore, we demonstrated that the transcriptional co-factor Mediator 8 is a critical regulator of this zone. Depletion of Mediator 8 impairs the formation of the Anterior Regenerative Zone, disrupts polarity establishment, and prevents successful blastema formation.

Conclusions: Our study provides a holistic molecular and cellular reconstruction of whole-body regeneration, directly linking dynamic gene expression gradients to morphological restoration. The discovery of the Mediator 8-regulated Anterior Regenerative Zone highlights the importance of transient spatial domains in coordinating tissue repair. The resulting interactive atlas serves as a foundational resource for deciphering the logic of spatiotemporal patterning in regeneration.

Powered by Editorial Manager® and ProduXion Manager® from Aries Systems Corporation

Corresponding Author:	Mengyang Xu<br>BGI Group<br>Qingdao, CHINA
Corresponding Author Secondary Information:	
Corresponding Author's Institution:	BGI Group
Corresponding Author's Secondary Institution:	
First Author:	Kai Han
First Author Secondary Information:	
Order of Authors:	Kai Han
	Yue Chen
	Yao Li
	Lidong Guo
	Yuxiaofei Wang
	Xiawei Liu
	Yaru Lin
	Zhi Huang
	Qun Liu
	Wenjie Guo
	Rui Zhang
	Wandong Zhao
	Langchao Liang
	Xiaoyu Wei
	Li Zhou
	Xuebin Mao
	Jiaqi Wang
	Weijian Wu
	Hongwei Pan
	Tao Yang
	He Zhang
	Xiaoshan Su
	Shanshan Liu
	Wenwei Zhang
	Longqi Liu
	Søren Tvorup Christensen
	Jifeng Fei
	Xin Liu
	Guangyi Fan
	Hanbo Li
	Ying Gu

Powered by Editorial Manager® and ProduXion Manager® from Aries Systems Corporation

	Jian Wang
	Huanming Yang
	Gang Pei
	Xun Xu
	An Zeng
	Mengyang Xu
**Order of Authors Secondary Information:**	
**Response to Reviewers:**	Please note that all line numbers referenced in our Response to Reviewers correspond to the line numbering in the revised version of the manuscript.<br>Please also note that due to technical constraints of the online submission system, only text can be submitted, while figure uploads are not supported. Accordingly, all aforementioned figures are included in the Supplementary Materials file titled "Point-by-Point Response-20260224.docx."<br><br>Response to Reviewer #1:<br>General Comments: The authors employs high-resolution Stereo-seq technology combined with multi-timepoint spatial transcriptomic data to construct a 4D spatiotemporal transcriptomic map of planarian regeneration. This work significantly advances the understanding of spatial gene expression dynamics during planarian regeneration, overcoming the limitations of traditional two-dimensional and planar spatial transcriptomics. Furthermore, the authors identify a novel injury-indced Anterior Regenerative Zone (ARZ) and, through functional validation of Mediator 8 (med8), deepen insights into the mechanisms underlying polarity remodeling in planarians. The study also provides an interactive online database, enriching spatial molecular and cellular data resources for the regenerative biology. The work is notably innovative, and the authors present convincing evidence supporting their conclusions. The manuscript overall is written well and data is presented clearly. The discussion and conclusions has done well to highlight the potential problems in this study. I have a few points that should be addressed before publishingI have a few points that should be addressed before publishing.<br><br>Response: We are deeply grateful to the reviewer for the highly positive assessment of our work and for recognizing the significance of our 4D spatiotemporal transcriptomic map in advancing the understanding of planarian regeneration. We particularly appreciate your acknowledgement of the technical innovation behind our high-resolution Stereo-seq approach, as well as the biological insights regarding the injury-induced ARZ and the regulatory role of med8 in polarity remodeling. We are also glad that the interactive online database was found to be a valuable resource for the community.<br>We sincerely thank you for your constructive comments and the specific points raised regarding the temporal dynamics of PCGs and the regulatory scope of med8. We have taken these suggestions very seriously and have performed additional analyses and revisions to address each point. We believe these changes have significantly strengthened the logical rigor and clarity of our manuscript. Please find our point-by-point responses below.<br><br><br>Comment 1: In lines 233-236, it is reported that the positional control gene (PCG) like ndk restores its spatial expression pattern as early as 12 hpa, whereas its expression level only significantly increases at 36 hpa. Given this pronounced temporal discordance between early recovery of spatial patterning and the later peak in mRNA levels, the authors should analyze and discuss possible molecular mechanisms that could account for this discrepancy, and consider the biological implications of this phenomenon for understanding how spatial information and gene-expression regulation are coordinated during regeneration.<br><br>Response: We thank the reviewer for highlighting this critical temporal feature. The observation that spatial patterning (e.g., ndk asymmetry at 12 hpa) is re-established well before the surge in transcriptional abundance (36 hpa) suggests a fundamental "Pattern-then-Amplify" regulatory logic. We have expanded our Discussion to interpret

this phenomenon through three synergistic mechanisms, supported by additional literature analysis:

1.Retention of Positional Memory in Existing Tissue

Planarian PCGs are predominantly expressed in body-wall muscle cells, which harbor positional information (Witchley, J. N., et al. (2013). Muscle cells provide instructions for planarian regeneration. Cell Reports, 4(4), 633–641.). Following amputation, the early recovery of spatial patterns at 12 hpa likely represents the rapid repolarization or maintenance of identity in pre-existing muscle cells at the wound boundary, rather than de novo pattern formation in new tissue. As previously demonstrated, planarians utilize landmarks in existing tissue to define the coordinates for regeneration immediately after injury (Oderberg, I. M., et al. (2017). Landmarks in existing tissue at wounds are utilized to generate pattern in regenerating tissue. Current Biology, 27(5), 733–742.). Thus, the organism re-establishes the map using the surviving tissue scaffold before constructing the blastema.

2.Early Signaling Precedes Transcriptional Bursts

The initial definition of head and tail spatial domains is driven by rapid signaling cascades (e.g., Wnt, MAPK) and post-translational modulators that operate prior to large-scale gene expression changes. For instance, it recently demonstrated that the phosphatase DjPtpn11 modulates early wound response genes and Wnt signaling dynamics shortly after injury, acting as an essential starter for the regenerative program (Pang, Q., et al. (2022). DjPtpn11 is an essential modulator of planarian (Dugesia japonica) regeneration. International Journal of Biological Macromolecules, 209, 1054–1064.). This signaling-primed state allows for the precise spatial restriction of PCG expression pattern at low basal levels before the system ramps up production.

3.Decoupling for Regenerative Fidelity

The lag between pattern establishment (12 hpa) and the transcriptional peak (36 hpa) serves as a fidelity checkpoint. The massive upregulation at 36 hpa coincides with the onset of blastema formation and neoblast proliferation. By decoupling these phases, the organism ensures that the proliferation machinery is only fully engaged after the spatial coordinate system has been correctly verified. This hierarchical strategy prevents maladaptive growth and ensures that the new tissue integrates seamlessly with the old.

We have revised the Discussion section to articulate this “Pattern-then-Amplify” model, integrating these citations to provide a robust molecular explanation for the observed spatiotemporal dynamics (Lines 496-508).

Comment 2: In lines 356-360, Med8 knockdown markedly reduces the ARZ cell lineages and the expression of anterior-posterior polarity markers (e.g., sfrp-1, wnt1, wnt11-1), producing a clear effect on regeneration polarity formation.However no gross disruption of the whole-body AP axis was observed. Please further analyze and discuss the possible regulatory scope and mechanisms of Med8. Specifically, do other redundant pathways or compensatory mechanisms exist in planarians that maintain global positional information despite loss of Med8? What is the hierarchical and cell-type specificity of Med8's role in polarity regulation?

Response: We thank the reviewer for identifying this apparent paradox. The observation that med8 RNAi significantly dampens polarity markers without causing a gross axis duplication (e.g., double-head or double-tail) provides critical insight into its hierarchical role. We have expanded our analysis and Discussion to clarify that med8 functions as a differentiation-enabler for polarity-organizing cells, rather than a direct instructor of the body axis gradient itself.

1.Lineage-Dependent Polarity Expression Hypothesis

Our scRNA-seq data demonstrate that med8 is essential for the differentiation of specific ARZ lineages, particularly epidermal, muscle, and neural cells. In planarians, positional information is generated and maintained by these specialized differentiated cells. Therefore, the observed reduction in polarity markers (wnt1, sfrp-1) is a secondary consequence of the failure to generate the signal-producing cells, rather than a direct transcriptional silencing of polarity genes in the pre-existing tissue. Unlike instructional genes (e.g., β-catenin), whose perturbation directly flips the binary switch of axial identity, med8 depletion leads to a loss of voice for the organizer, resulting in regenerative failure rather than ectopic patterning (Lines 395-405).

2.Symmetric Signal Attenuation Preserves the Gradient Profile

Crucially, our data show that med8 knockdown reduces both anterior (sfrp-1) and

posterior (wnt1, wnt11-1) markers simultaneously. This symmetric reduction maintains the relative antagonism between Wnt and anti-Wnt signals, preventing the dominance of one pole over the other. In our case, the global dampening of the gradient amplitude halts blastema formation but preserves the gross body plan. (Lines 406-416)
3.Temporal Dynamics
While gross axis disruption was absent in the short regeneration window likely due to the stability of the extracellular matrix and long-lived muscle cells in the stump, we observed that prolonged med8 RNAi leads to head regression in homeostatic animals. This confirms that med8 is eventually required for maintaining global positional information by ensuring the continuous turnover of polarity-specifying cells. (Lines 434-437)
4.Subunit Specificity and Functional Redundancy
The Mediator complex is modular. Previous studies indicate that different subunits govern distinct biological outputs; for instance, Smed-med14 is required for stem cell maintenance, whereas med8 specifically directs differentiation. This suggests that while med8 is critical for the differentiation-coupled polarity maintenance, other Mediator subunits or parallel signaling pathways may compensate to maintain basal transcript levels in surviving tissue, preventing immediate axis collapse. (Lines 532-538)
We have revised the Discussion to incorporate these points, clarifying the hierarchical distinction between med8 (differentiation of the organizer) and canonical PCGs (instruction of the axis) (Lines 544-557).

Response to Reviewer #2:
General Comments: In the manuscript '4D single-cell spatial transcriptomics reveals dynamic morphogenetic gradients and regenerative domains in planarians,' Han and colleagues generate a truly stunning spatial transcriptomics dataset of planarian regeneration from the species Schmidtea mediterranea. The authors' dataset includes whole 3D reconstructions of two regenerating planarian fragments at 8 different timepoints during regeneration, a fantastic accomplishment and resource of broad interest to the regenerative biology community. The authors analysis of the dataset includes characterization of spatially biased genes (SBGs) and exploration of an anterior regenerative zone (ARZ) and the role of the gene med8 in its' regulation. While the authors' dataset is remarkable and their analysis of spatially biased genes and med8 function is interesting, I'm not yet convinced that their conclusions are fully tested by the included experiments. In addition, I think that the authors have not included sufficient quality control metrics for their spatial dataset, which makes determining the limitations or caveats of their analysis and conclusions more difficult. However, my concerns could be addressed by additional analysis and minor experiments, or by softening the conclusions of the authors to include alternative models. I've detailed the areas of analysis/discussion that I believe require improvement below:

Response: We sincerely thank Reviewer #2 for their highly encouraging evaluation of our work and for recognizing our 4D spatial transcriptomics dataset as a stunning and fantastic accomplishment that will serve as a valuable resource for the regenerative biology community. We truly appreciate the time and effort dedicated to reviewing our manuscript.
We find your constructive criticism regarding the interpretation of our conclusions, particularly concerning med8 function and spatially biased genes, and the need for more rigorous quality control metrics to be extremely insightful. We have taken these comments very seriously and have performed additional analyses and text revisions to address each point raised. We believe these improvements have significantly strengthened the manuscript, and we provide a detailed point-by-point response to your specific concerns below.

Major Criticisms:
Comment 1: Stereo-seq resolution and capture efficiency: The authors assert that their spatial approach is high enough resolution to resolve cell types and they claim to have characterized 36 cell types in their abstract. However, the 'cell type' in their dataset that they choose to focus on - Clu.31 - has gene markers expressed in three different cell types that have been shown to be distinct in the literature and prior planarian atlases. The authors should analyze gene expression signatures of other stereo-seq 'cell types'

Powered by Editorial Manager® and ProduXion Manager® from Aries Systems Corporation

to determine if they also show mixed expression signatures. In addition, I am curious if stereo-seq is more likely to capture highly expressed genes (like those expressed in parenchymal cell types) than more lowly expressed genes (like the transcription factors expressed in stem cells). If it exists, this bias could influence annotation of cell types in highly heterogeneous regions of the worm like the parenchyma or parapharyngeal region. Finally, there is very little QC data in the supplementary materials (Size/volume of segmented cells, UMIs and features per cell, variability in features/UMIs per section, per replicate, and per cell type, etc.) I think this analysis would be highly valuable for the reader to interpret the data and the 36 identified 'cell types'.

Response: We thank the reviewer for this critical assessment of our spatial terminology and quality control metrics. We acknowledge that the term “cell type” was applied too broadly in our initial submission. While our high-resolution Stereo-seq approach (715 nm) is capable of resolving single cells, we agree that the clusters identified via Spatial Proximity Clustering (SPC) technically represent spatial proximity domains, collections of cells with similar gene expression profiles that may include both monophyletic cell types and heterogeneous cellular neighborhoods. We have revised the manuscript to adopt the term “domains” to more accurately reflect the nature of these spatial units. Regarding the mixed signature of the Anterior Regenerative Zone (ARZ/Clu.31), we maintain that this is a verified biological feature rather than a technical artifact. The blastema is a region of high cellular density and extensive intercellular communication. Consequently, Clu.31 captures a functional regenerative niche where epidermal, muscle, and neural lineages physically converge. This interpretation is supported by our high-resolution in situ hybridization data (Fig. 3B-D), which confirms that smed03831-positive cells are physically intermingled with these distinct lineages. This multi-lineage convergence zone is reminiscent of the specialized microenvironments observed in other regeneration models.
To address the reviewer’s concern regarding the purity of the other 35 clusters, we performed a comprehensive correlation analysis comparing our Stereo-seq gene signatures with three published single-cell RNA-seq atlases (Emili, E., Pérez-Posada, A., Vanni, V., Salamanca-Díaz, D., & Solana, J. (2025). Allometry of cell types in planarians by single-cell transcriptomics. Science Advances, 11(19), eadm7042. Plass, M., Solana, J., Wolf, F. A., Ayoub, S., Misios, A., Glažar, P., Obermayer, B., Theis, F. J., Kocks, C., & Rajewsky, N. (2018). Cell type atlas and lineage tree of a whole complex animal by single-cell transcriptomics. Science, 360(6391), eaaq1723. Fincher, C. T., Wurtzel, O., de Hoog, T., Kravarik, K. M., & Reddien, P. W. (2018). Cell type transcriptome atlas for the planarian Schmidtea mediterranea. Science, 360(6391), eaaq1736.) and a 10x Genomics Visium spatial dataset (Cui, G., Dong, K., Zhou, J.-Y., Li, S., Wu, Y., Han, Q., Yao, B., Shen, Q., Zhao, Y.-L., Yang, Y., Cai, J., Zhang, S., & Yang, Y.-G. (2023). Spatiotemporal transcriptomic atlas reveals the dynamic characteristics and key regulators of planarian regeneration. Nature Communications, 14(1), 3205.). This analysis, presented in the new Supplementary Fig. S2 (referred to as Fig. R1 here), reveals the cellular composition of our domains (Lines 149-153, 682-687):
1.High-Fidelity Cell Types
Clusters 12 and 14 exhibit high-quality, one-to-one mappings with specific single-cell subtypes, confirming they represent distinct cell populations.
2.Lineage-Restricted Domains
The majority of domains are lineage-specific. For example, clusters 2, 5, 6, 8, 10, 13, 16, and 21 map exclusively to the Gut lineage; clusters 19, 29, and 35 map to the Pharynx; clusters 25 and 28 map to the Neural lineage; and cluster 17 maps to Muscle. These domains show minimal cross-contamination from other germ layers.
3.Stem Cell and Parenchymal Niches
Cluster 3 maps cleanly to the neoblast subtype. However, clusters 7, 9, 11, 22, and to a lesser extent 0, 4, 23, 26, and 33, exhibit signatures of both neoblasts and parenchymal cells. This finding aligns with recent single-cell studies describing the heterogeneity of stem cell microenvironments and the presence of transient post-mitotic states within the parenchyma (Benham-Pyle, B. W., Brewster, C. E., Kent, A. M., Mann, F. G., Chen, S., Scott, A. R., ... & Sánchez Alvarado, A. (2021). Identification of rare, transient post-mitotic cell states that are induced by injury and required for whole-body regeneration in Schmidtea mediterranea. Nature Cell Biology, 23(9), 939–952.).
Figure R1: Correlation analysis of Stereo-seq gene expression signatures with published transcriptomic datasets. (A-C) Heatmaps displaying the Pearson correlation

coefficients between the gene expression profiles of Stereo-seq clusters (columns) and annotated cell types from published single-cell RNA-seq datasets (A: Elena Emili et al., Science Advances, 2025; B: Mireya Plass et al., Science, 2018; C: Christopher T. Fincher et al., Science, 2018). (D) Similarity comparison between Stereo-seq clusters and spatial domains defined in previously published 10x Genomics Visium datasets (Cui, G., Dong, K., Zhou, J.-Y., Li, S., Wu, Y., Han, Q., Yao, B., Shen, Q., Zhao, Y.-L., Yang, Y., Cai, J., Zhang, S., & Yang, Y.-G. (2023). Spatiotemporal transcriptomic atlas reveals the dynamic characteristics and key regulators of planarian regeneration. Nature Communications, 14(1), 3205.). The color scale represents the Pearson correlation coefficient, with red indicating higher similarity between the corresponding groups.

4.The Unique ARZ
Consistent with our hypothesis, Clu.31 remains unique in its distinct tri-lineage enrichment (epidermal, neural, muscle), distinguishing it as a complex signaling center. Regarding capture efficiency and bias, we acknowledge the inherent sparsity of nanoball-based spatial transcriptomics (random sparse capture). However, to test for systematic bias against lowly expressed genes (e.g., transcription factors), we calculated the correlation between our aggregated Stereo-seq “pseudo-bulk” data and published bulk RNA-seq datasets (Roberts-Galbraith, R. H., Brubacher, J. L., & Newmark, P. A. (2016). A functional genomics screen in planarians reveals regulators of whole-brain regeneration. eLife, 5, e17002.). We observed a high Pearson correlation ($R \geq 0.8$), suggesting that the sparsity is random and does not disproportionately exclude low-abundance regulatory genes essential for defining cell identity.
Finally, we have significantly expanded the Quality Control metrics in the Supplementary Materials as requested. We updated Data S1 to include median Gene counts (150–230), UMI counts (190–370), and segmented cell areas (70–120 pixels) per section and per cluster. These metrics demonstrate high consistency across replicates and sections, ensuring that biological interpretations are not driven by technical variability.

Comment 2: Dynamics of spatially biased genes: The authors analysis on the dynamics of spatially biased genes (SBGs) is very interesting, but the 'oscillations' the authors referred to were not clear to me in the data across all or even most of the pattern clusters in Figure 2A. In general, it seemed more like the pattern cluster was 'noisy' or more broad before stabilizing to its final location. In addition, the PCA analysis in Figure 2B seems to show that Intact and 14dpa transcriptomics is very similar, but 0h, 12h, and 36h timepoints are very distinct from 3, 5, 7, and 10 day fragments. This would suggest that early wound response gene expression is highly distinct (even opposing) the gene expression programs active during late in regeneration. More exploration of this idea, as well as clarified language on exactly what the author means by 'oscillations' and which gene groups follow this pattern would greatly improve this section and better support the author's conclusions.

Response: We thank the reviewer for this insightful observation, particularly regarding the interpretation of the PCA trajectories and the terminology used to describe gene expression dynamics. We agree that the term “oscillation” carries a connotation of periodic sinusoidal behavior, which does not accurately reflect the biological reality of a system returning to homeostasis following severe structural perturbation. To address this, we have revised our terminology from “oscillations” to “underdamped dynamic responses”. We have adopted a systems biology perspective, referencing control theory and physical mechanics to better describe this process (Inman, D. J. (2007). Engineering Vibration (3rd ed.). Pearson Education, Inc. MacArthur, B. D., Ma'ayan, A., & Lemischka, I. R. (2009). Systems biology of stem cell fate and cellular reprogramming. Nature Reviews Molecular Cell Biology, 10(10), 672–681.). In this framework, we model the regenerating planarian as a dynamic system where equilibrium represents the intact, homeostatic state; perturbation represents the amputation event, which displaces the system far from equilibrium; and restoring force represents the GRN that drives the system back toward the homeostatic attractor. Under this model, the noisy broad patterns observed in Fig. 2A represent the system’s transient response, an initial overshoot and subsequent convergence, as it searches for and stabilizes at the new equilibrium.

Furthermore, we agree with your interpretation of the PCA data in Fig. 2B. The distinct separation of time points indeed reflects a phase transition in the biological program:

1.Acute Injury Phase (0h–36h)

The system is in a high-energy, non-equilibrium state dominated by wound healing and immediate injury responses, distinct from both the regenerative and homeostatic states. This aligns with the identification of transient, injury-induced cell states described in recent literature.

2.Patterning & Morphogenesis Phase (3d–10d)

The system begins to converge, dominated by the re-establishment of positional information and tissue differentiation.

3.Homeostatic Return (14dpa)

The system returns to a state statistically similar to the intact control.

We have revised the manuscript to explicitly discuss these distinct phases and have removed the confusing references to "oscillations" in favor of this more rigorous physical systems model (Lines 247-264, 467-473).

Comment 3: The Cellular/Functional identity of Clu.31: The authors state throughout the manuscript that Clu.31 (the ARZ) is an injury-induced anterior state enriched for SBGs and regulating polarity establishment. However, it is also possible that this spatial state represents the anterior peripheral nervous system (numerous sensory neurons and surface epithelial cells that help sense mechanical and chemical cues). SBGs could be enriched because this combination of cell types is only present in the anterior of the animal. Indeed, the authors show that the ARZ is localized to the anterior in intact animals in the absence of an injury (Figure 3) and enriched genes (S4Aii) strongly indicate that Clu.31 contains gabrg+ mechanosensory neurons. If Clu.31 is regenerating nervous system, this would also explain its ventral bias and expression of tgs-1 and other nb2 genes, since nb2 neoblasts have been suggested to be both an amputation responsive neoblast subset (Zeng et al. Cell) and a neural progenitor state (Raz et al. Cell Stem Cell). Clarifying how the composition of the tri-lineage region changes during regeneration may help distinguish if Clu.31 is truly an injury induced region vs. the regenerating sensory nervous system. For example, it is known that agat-1+ cells transcriptionally responsive and enriched at the wound site a 2-4 days post amputation, but less so at later timepoints (Benham-Pyle et al Nature Cell Biology, Kent et al. Developmental Biology). This shift in composition should be observable in Clu.31 since it contains agat+ epidermal cells. Such a shift in composition or the identification of a regeneration-specific marker expressed in Clu.31 would add support to the author's conclusions. Regardless of the outcome of these experiments/analyses, the discussion and interpretation of the data could be modified to address the hypothesis that Clu.31 represents the cellular neighborhood created when the peripheral nervous system intercalates with the anterior DV boundary epithelium and body wall muscle, which needs to be regenerated in amputated worms. As is, the comparison to the apical epithelial cap considered in the discussion (Line 438) may be pre-mature.

Response: We thank the reviewer for this insightful alternative interpretation. We agree that the cellular composition of Clu.31 rich in sensory neurons and epithelial cells shares significant features with the anterior peripheral nervous system. We appreciate the suggestion to consider this structural identity and have carefully re-analyzed our data to address this point. While Clu.31 contains components of the regenerating nervous system, we respectfully propose that it functions as a dynamic regenerative organizing center rather than solely representing the regenerating anterior structures. This conclusion is supported by three key lines of evidence from our 4D dataset:

1.Transient Appearance in the Tail Blastema (Fig. 3E; Supplementary Fig. S2F)

Crucially, the Clu.31 signature emerges transiently in both anterior and posterior wound sites (head and tail blastemas) at 36 hpa. In tail fragments, which are regenerating a tail and not an anterior brain, this signal appears and then recedes. If Clu.31 were simply the anterior PNS, its specific molecular signature would not be expected to appear in the posterior blastema. This "appear-and-restrict" pattern suggests a role in global polarity assessment.

2.Dynamic Compositional Shifts (Fig. 3H-J)

As suggested, we examined the temporal changes in cell composition. The composition of Clu.31 is not static or linearly increasing (as might be expected for simple tissue growth) but is highly dynamic: initiating with a predominant epidermal

signature (12 hpa), recruiting muscle and neural lineages during blastema formation (3 dpa), and eventually reverting to a homeostatic state.
3.Distinct Expression Dynamics of agat-1 (New)
Following the reviewer's specific suggestion regarding agat-1+ cells, we plotted the temporal expression of agat-1 within the Clu.31 cluster (Fig. R2). The results strikingly confirm the reviewer's prediction of a compositional shift:
1)Injury Response (36 hpa – 5 dpa): agat-1 expression is widespread and high, consistent with the recruitment of transient, injury-responsive epidermal states (Benham-Pyle, B. W., Brewster, C. E., Kent, A. M., Mann, F. G., Chen, S., Scott, A. R., ... & Sánchez Alvarado, A. (2021). Identification of rare, transient post-mitotic cell states that are induced by injury and required for whole-body regeneration in Schmidtea mediterranea. Nature Cell Biology, 23(9), 939–952.).
2)Remodeling Dip (7 dpa – 10 dpa): Crucially, we observe a significant reduction in agat-1 expression and cell percentage during the mid-to-late regeneration phase, before recovering in homeostasis. This non-linear fluctuation demonstrates that the ARZ undergoes specific compositional remodeling distinct from monotonic tissue growth.

Figure R2: Temporal expression dynamics of agat-1 in the Clu.31 cluster. Note that the distinct high levels during early regeneration (36hpa-5dpa) followed by a reduction at 7-10dpa, indicating a dynamic state shift.

In light of these findings, we have revised the Discussion section: 1) acknowledge that Clu.31 physically represents a cellular neighborhood comprising mechanosensory neurons, epithelium, and muscle; 2) cite relevant literature on transient cell states (Benham-Pyle, B. W., Brewster, C. E., Kent, A. M., Mann, F. G., Chen, S., Scott, A. R., ... & Sánchez Alvarado, A. (2021). Identification of rare, transient post-mitotic cell states that are induced by injury and required for whole-body regeneration in Schmidtea mediterranea. Nature Cell Biology, 23(9), 939–952. Kent, A. M., Guerrero-Hernández, C., Brewster, C., McKinney, S., Morrison, J. A., McKinney, M. C., Ross, E. J., Mann, F. G., Jr, Benham-Pyle, B. W., & Sánchez Alvarado, A. (2026). Metabolites produced by agat+ cells support regeneration in the planarian Schmidtea mediterranea. Developmental Biology, 529, 106–120.) to contextualize the dynamic compositional shifts we observed; and 3) soften the comparison to the vertebrate Apical Epithelial Cap (AEC), describing it as functionally reminiscent rather than implying direct homology (Lines 509-528).

Comment 4: Med8 function: Med8 produces a clear phenotype in the authors' experiments, and their data indicates that it is required for ARZ formation. However, I am not sure that the authors data supports the claim that Med8 is directly regulating blastema and PCG expression, as opposed to regeneration of the nervous system (which is highly interconnected with formation of the anterior pole and the size of the anterior blastema) and stem cell function more broadly. The fact that Med8 RNAi also leads to head degeneration in intact worms (Figure S6F) strongly suggests a more fundamental defect in neural differentiation or stem cell function. The strongest evidence presented by the authors supporting a broader function in polarity establishment is the disruption of posterior Wnt expression, (Figure 5F and G), but these in situs are single representative images with no quantitation and could also be explained by a stem cell defect. Additional data could be provided (e.g. visualization of wound-induced gene expression, quantitation of anterior or posterior stem cell numbers and proliferation rates at 2dpa) to support regulation of PCGs or blastema formation. The authors could also leverage their single cell sequencing to determine if Med8 RNAi impacts neural progenitor abundance more than other progenitor cell types. Together, these experiments would determine if Med8 is important for amputation-induced blastema formation and polarity re-establishment vs. stem cell function and neural differentiation more broadly.

Response: We sincerely thank the reviewer for this insightful comment. We fully agree with the reviewer’s interpretation that med8 is likely a fundamental regulator of stem cell function, rather than a direct regulator of polarity signaling. We acknowledge that our original description may have implied a direct regulatory role, which was not our intention. We have revised the manuscript to clarify the hierarchical mechanism: med8 is required for the formation of the ARZ, and the ARZ subsequently acts as the

organizer to establish polarity. Thus, the polarity defect is a secondary consequence of the failure to form the ARZ.
The reviewer asked whether the phenotype is driven specifically by a failure in neural regeneration, which then affects the anterior pole. To test this, we followed the reviewer's suggestion to analyze whether med8 RNAi impacts neural progenitor abundance more than other progenitor types. Our scRNA-seq analysis (Fig. R3) reveals that the defect is not neural-specific, but rather a broad suppression of lineage commitment. When we normalized progenitor counts to the size of the neoblast pool (calculating the Differentiation Efficiency), we found a consistent collapse across all major lineages:
1.Neural Differentiation: Efficiency reduced by 66.9%.
2.Muscle Differentiation: Efficiency reduced by 45.5%.
3.Epidermal Differentiation: Efficiency reduced by 45.9%.
The comparable reduction in muscle and epidermal progenitors demonstrates that med8 is not a neural-specific regulator. Since muscle cells are the cellular source of positional information in planarians, the differentiation blockade in the muscle lineage provides a sufficient mechanistic explanation for the polarity defect, independent of the neural phenotype.

Figure R3: Med8 RNAi causes a broad reduction in differentiation efficiency across ARZ lineages.

We also agree with the reviewer that the head regression phenotype points to a fundamental defect in stem cell function. However, our data allows us to be more specific about which function is impaired. In med8 (RNAi) animals, the absolute number of undifferentiated neoblasts (piwi-1+/lineage-negative) actually increased (accumulated) compared to controls, indicating that stem cell maintenance and self-renewal capabilities are preserved. The combination of "neoblast accumulation" and "progenitor depletion" defines the defect as a Differentiation Blockade. Stem cells are maintained but functionally fail to exit the pluripotent state to generate differentiated progeny.
We have rewritten the relevant Results and Discussion sections to adopt this refined model. We no longer claim med8 directly regulates PCGs. Instead, we propose that med8 is required for the broad differentiation output of stem cells, including the muscle and neural lineages that constitute the ARZ. The loss of these signaling centers subsequently leads to the observed polarity collapse.

Minor Criticism/Feedback:
Comment 5: In Figure 1I, the authors show DEGs enriched in each cluster/region. In the blastema regions, I was surprised by the number of DEGs for each time point. It appears that there are ~10K upregulated and 10K downregulated DEGs by the later time points, which suggests that 2/3 of the transcriptome is differentially expressed… The authors should clarify in the text or methods what cutoff they used for the DEGs and how significant the DEGs are in this figure.

Response: We thank the reviewer for this careful observation. We apologize for the omission of specific methodological details regarding the algorithms and cutoffs used for differential expression analysis in the original manuscript. To clarify, the DEGs presented in Fig. 1I were identified using DEsingle, an R package specifically designed to handle the zero-inflation characteristic inherent to single-cell and spatial transcriptomics data. Our analysis strategy involved pairwise comparisons between adjacent time points (e.g., 0 hpa vs. 12 hpa, 12 hpa vs. 36 hpa) for each cluster and blastema region to capture dynamic temporal transitions. We applied a stringent statistical cutoff of False Discovery Rate (FDR) < 0.05 (Benjamini-Hochberg adjusted p-value) to define significance.
The relatively high number of DEGs observed is attributable to two main factors. First, DEsingle is more sensitive than traditional methods; it detects not only changes in mean expression (Type I) but also changes in the proportion of expressing cells (Type II) and composite changes (Type III). This allows us to capture subtle but biologically significant shifts in cell activation states that standard fold-change cutoffs might miss. Second, whole-body regeneration involves profound transcriptomic remodeling, particularly in the blastema, where cells undergo rapid dedifferentiation and re-

Powered by Editorial Manager® and ProduXion Manager® from Aries Systems Corporation

specification. We have expanded the Methods section to explicitly describe this methodology (Lines 699-709).

Comment 6: For readability, I really think that all figures should be on a white background.
Response: We appreciate the reviewer's suggestion regarding figure readability. We have adjusted the background to white for the majority of the figures to ensure clarity and standard presentation. However, for certain high-density spatial transcriptomic maps (specifically the in situ cell type visualizations), we retained a dark background. This is a standard practice in spatial transcriptomics visualization to ensure sufficient contrast for distinguishing multiple fluorophores or cluster colors, which would otherwise be indiscernible against a white background.

Comment 7: How do gene expression profiles from the stereo-seq compare to bulk rnaseq at similar timepoints?

Response: We thank the reviewer for suggesting this crucial technical validation. To rigorously assess the quantitative accuracy of our Stereo-seq data, we compared our spatial transcriptomic profiles with high-quality published bulk RNA-seq datasets derived from homeostatic planarians (Roberts-Galbraith, R. H., Brubacher, J. L., & Newmark, P. A. (2016). A functional genomics screen in planarians reveals regulators of whole-brain regeneration. eLife, 5, e17002.).
We retrieved raw sequencing data for three biological replicates of intact animals (Accession numbers: SRR3465425, SRR3465432, SRR3465433) from the Sequence Read Archive (SRA). Reads were aligned to the S. mediterranea genome (dd_Smes_G4 assembly) using HISAT2, and gene counts were quantified using featureCounts, ensuring the reference genome and pipeline matched our Stereo-seq analysis. From our Stereo-seq dataset, we aggregated all captured UMIs from the homeostatic individual to construct a pseudo-bulk expression profile. Both datasets were normalized to Counts Per Million (CPM) and log2-transformed to allow for direct comparison. As shown in Fig. R2 (and added to Supplementary Fig. S1C in the revised manuscript), the Stereo-seq pseudo-bulk profile exhibits a high correlation with all three bulk RNA-seq replicates, with Pearson correlation coefficients (R) ranging from 0.80 to 0.81. This strong concordance confirms that our Stereo-seq platform captures the global transcriptome with high sensitivity and quantitative reliability, comparable to standard bulk sequencing methods.

Figure R4: Correlation analysis between Stereo-seq pseudo-bulk data (Homeostasis) and three independent bulk RNA-seq replicates (Roberts-Galbraith et al., 2016). The Pearson correlation coefficient (R) is indicated for each replicate.

Furthermore, we have included this correlation analysis in Supplementary Fig. S1C and added a description of this validation step in the Methods section under "Data Quality Control and Validation".

Comment 8: It is very interesting that there are some cell types that appear to contract and then expand during regeneration (Cluster 0, 23) or that aggregate/become more targeted during regeneration (pharynx pouch, cluster 29). Molecular differences between early and late cells within these cell types would be particularly interesting for understanding different phases of regeneration, but this may be beyond the scope of the current study.

Response: We appreciate the reviewer's interest in the molecular dynamics of these specific cell populations. We fully agree that characterizing the molecular differences between early and late stages within these dynamic clusters (e.g., Clusters 0, 23, and 29) is critical for understanding their regenerative behaviors. In fact, we have already performed this systematic analysis in our original manuscript. As summarized in Fig. 1I, we calculated the DEGs for each cluster including Clusters 0, 23, and 29 and blastema regions between adjacent regeneration timepoints (e.g., 0hpa vs. 12hpa, 12hpa vs. 36hpa, etc.).

Powered by Editorial Manager® and ProduXion Manager® from Aries Systems Corporation

The detailed results of this analysis are provided in Supplementary Table S2. This supplementary dataset is organized to facilitate exactly the kind of inquiry the reviewer suggests:
1.Each sheet represents a comparison between adjacent time intervals.
2.Columns correspond to specific clusters including the dynamic ones highlighted by the reviewer or regions.
3.Rows list the genes, with values indicating the expression differences.
Thus, the molecular transitions underlying the contraction, expansion, or aggregation of these specific cell types are fully captured and accessible within our provided data. We believe this comprehensive resource allows the community to mine the specific molecular programs driving these fascinating cellular behaviors.
We have ensured that the reference to Supplementary Table S2 and Fig. 1I is clear in the Results section (Lines 209-211), emphasizing that time-resolved molecular differences for all clusters are available for detailed exploration.

Comment 9: The authors frequently reference Han et al. submitted, but this manuscript would need to be pre-printed or published in order for this work to reference it.

Response: We have updated the citation. The manuscript “Han et al.” has now been uploaded to bioRxiv (Maoqin Sun, Yuxiaofei Wang, Kai Han, et al. (2026). Cell Type Architecture and Positional Gene Gradients in an Adult Animal at Subcellular Resolution. bioRxiv, 2026.02.19.705280), and the full citation has been added to the reference list.

Comment 10: The Y axis of Figure 2E should be labeled

Response: We have added the missing label to the Y-axis of Fig. 2E as suggested.

Response to Reviewer #3:
General Comments: In the manuscript entitled "4D Single-Cell Spatial Transcriptomics Reveals Dynamic Morphogenetic Gradients and Regenerative Domains in Planarians", Han, Chen, Li et al. use high-resolution Stereo-seq on regenerating planarians to reconstruct a 4D spatiotemporal transcriptomic map of planarian regeneration. In their analysis, they recognize most of the cell types identified in planarians and are able to recover the gene expression dynamics during regeneration of the body axes at the cellular, tissue, and organismal scales. One of the main findings is the identification of injury-induced spatial domains, specifically the Anterior Regenerative Zone (ARZ). Interestingly, the ARZ is enriched in positional control gene expression in several cell types, not only in muscle. The authors identify Mediator 8 as a gene expressed in the ARZ and required for proper blastema formation. The study also provides an interactive web portal with the corresponding data.
The analysis of the regeneration process in planarians using Stereo-seq provides a new and very useful strategy to understand the dynamics of gene expression integrated with cell and tissue types. Through this strategy, the authors corroborate the expression patterns of several genes already described as essential for planarian regeneration, and they identify new blastema regions comprising different expression patterns, both anterior and posterior. Among them, the study focuses on the ARZ and performs trajectory analysis, which provides a very informative view of the cellular movements and changes that occur at early stages of regeneration. The finding that Med8 is required to initiate regeneration in both wounds validates the utility of the strategy followed.
The publication of an open and interactive web portal with the dataset will be a useful tool for the planarian community and for research on regenerative processes in general.
However, in its present form, the study presents several weaknesses and issues that should be addressed before publication.

Main concerns:

The 3 main concerns are 1) the presentation of the strategy of each analysis

Powered by Editorial Manager® and ProduXion Manager® from Aries Systems Corporation

performed is not detailed and not clear enough; 2) essential data for the present manuscript is supposedly found it Han et al. submitted, when it should be available in the present manuscript; and 3) the conclusions from the functional analysis of med8 are not accurate.

Response: We extend our sincere gratitude to Reviewer #3 for the comprehensive and insightful evaluation of our manuscript. We appreciate the recognition of our 4D spatiotemporal transcriptomic atlas as a valuable resource for the regeneration community. The reviewer's constructive criticism regarding the definition of spatially biased genes, the interpretation of med8 function, and the clarity of our data integration strategies has been instrumental in refining the logic and precision of this study. We have carefully addressed every point raised, with particular attention to clarifying the distinction between cell fate specification and polarity establishment, as well as correcting the presentation errors in Fig. 2. Below, we provide a detailed response to each concern and outline the corresponding revisions made to the manuscript.

Regarding concern 1:
Comment 1: - The authors explain that they analyzed 16 animals (2 per time point) processed into 10 μm thick sections, producing a total of 353 sections. However, they do not specify whether the same number of sections were analyzed per animal, nor do they indicate the total size (or thickness) and cell number of the animals analyzed.
In this regard, it may be that the number of sections analyzed per animal is shown in Figure S2A (this is not clear from the figure legend or the text). If so, why is there variation in the number of sections per animal? Is it due to differences in animal size? If so, how were these differences addressed in order to integrate the data from different animals?

Response: We apologize for not making the metadata describing individual animals sufficiently prominent in the main text. We clarify that we have indeed recorded detailed metrics for every analyzed animal and employed standard computational strategies to robustly integrate data across individuals despite their biological variations.
1.Animal-Specific Metrics
As listed in Table S1 (Data S1), we provided a comprehensive summary for each of the 16 individuals. This table explicitly documents the exact section number, 3D body dimensions (DV/ML/AP lengths), tissue volume, and total cell counts for every replicate. To ensure clarity, we have now explicitly referenced this table in the Results section (Line 136).
2.Source of Variation
We presume the reviewer intended to refer to Supplementary Fig. S1A (mentioned as Supplementary Fig. S2A in the comment), which illustrates the sections analyzed per animal. The reviewer correctly notes that there is variation in these section counts. This is primarily due to natural biological variation in body size. Although we selected animals of similar size for the experiment, slight differences in body thickness (DV axis) result in different total section numbers, as documented in the physical measurements in Table S1.
3.Data Integration Strategy
To address these size differences and integrate data from different animals, we applied distinct strategies for cellular identity and spatial analysis:
1)For Cell Type Identification: To define cell types consistently across individuals, we employed the standard Seurat integration workflow. This method identifies anchors between datasets to remove batch effects and biological variability inherent to different individuals, allowing us to cluster cells based on their shared transcriptomic profiles rather than their sample of origin.
2)For Spatial Pattern Analysis: We did not perform rigid physical alignment of coordinates between individuals. Instead, to compare spatial distributions across animals of different sizes, we normalized the spatial coordinates (AP, ML, and DV axes) to a relative range (e.g., 0–1 or percentage distance) for each individual. This allowed us to calculate and compare the distribution patterns of cell types and gene expression signals along the canonical body axes, independent of the absolute size of the animal.

Powered by Editorial Manager® and ProduXion Manager® from Aries Systems Corporation

	Comment 2: - In Figure 1F, the different parts of the blastema are divided according to the pigmented/unpigmented area. What were the criteria used to divide each blastema into three parts (proximal, middle, and distal)? Gene expression? Length of the region? This should be clarified.<br><br>Response: We thank the reviewer for this question, as it highlights the need to better explain our segmentation logic. We clarify that the subdivision of the blastema was strictly anatomical and based on pigmentation patterns, not gene expression. This approach was chosen to provide an unbiased spatial reference frame independent of the molecular profiles we intended to study.<br>The segmentation process involved the following steps:<br>1.Pigmentation-Based Boundary Definition<br>We first identified the boundary between the pigmented (old tissue) and unpigmented (new blastema) regions using the Threshold function in ImageJ on microscopy images of the specific animals used for sequencing.<br>2.Quadratic Regression Refinement<br>To create a smooth, continuous mathematical boundary from the pixel data, we fitted a quadratic polynomial regression line to the pigmentation threshold.<br>3.Defining Border Regions (HBOR/HBIR)<br>A simple hard line often creates noise due to the irregular nature of the regeneration interface. To address this, we defined a buffer zone of 20 μm width on either side of the regression line.<br>1)HBOR (Head Border Outer Region): The 20 μm strip immediately inside the unpigmented blastema.<br>2)HBIR (Head Border Inner Region): The 20 μm strip immediately inside the pigmented trunk.<br>3)HAR (Head Anterior Region): The remaining distal portion of the blastema beyond the HBOR.<br>4)The same logic applies to the Tail blastema: TBOR, TBIR, and TPR.<br>This resulted in the distinct regions (HAR, HBOR, ...
**Additional Information:**	
**Question**	**Response**
Are you submitting this manuscript to a special series or article collection?	No
**Experimental design and statistics**<br><br>Full details of the experimental design and statistical methods used should be given in the Methods section, as detailed in our Minimum Standards Reporting Checklist. Information essential to interpreting the data presented should be made available in the figure legends.<br><br>Have you included all the information requested in your manuscript?	Yes
**Resources**<br><br>A description of all resources used, including antibodies, cell lines, animals and software tools, with enough information to allow them to be uniquely	Yes

identified, should be included in the Methods section. Authors are strongly encouraged to cite Research Resource Identifiers (RRIDs) for antibodies, model organisms and tools, where possible.<br><br>Have you included the information requested as detailed in our Minimum Standards Reporting Checklist?	
**Availability of data and materials**<br><br>All datasets and code on which the conclusions of the paper rely must be either included in your submission or deposited in publicly available repositories (where available and ethically appropriate), referencing such data using a unique identifier in the references and in the "Availability of Data and Materials" section of your manuscript.<br><br>Have you have met the above requirement as detailed in our Minimum Standards Reporting Checklist?	Yes
GigaScience has policies and guidelines in place for the use of generative AI-writing tools such as ChatGPT. If you have used such writing tools to assist with writing the manuscript this must be declared and cited in the text. Authors should not list AI-writing tools and other AI-assisted technologies as an author or co-author and should acknowledge that they are fully responsible for text generated or refined by AI-writing tools.<p><br><br>A summary of use (particularly in the introduction or among methods) needs to be included at the end of the paper, and the outputs should also be included as a supplementary file hosted in GigaDB or other open repositories. Please read our guidelines for more information. <p><br><br>By submitting to GigaScience, you are aware of the journal's AI-writing tools policy, and if you have declared use of	No

Powered by Editorial Manager® and ProduXion Manager® from Aries Systems Corporation

such tools below, you have acknowledged this where appropriate in your manuscript and have made a summary of use and outputs available. <p><br><b>AI-assisted writing tools have been used in the preparation of this manuscript?	

Revised manuscript with tracked changes
Click here to access/download;Manuscript;Manuscript-tracked-20260224.docx

4D Single-Cell Spatial Transcriptomics Reveals Dynamic Morphogenetic Gradients and Regenerative Domains in Planarians

Formatted: Line spacing: Double

Kai Han[1,3,†], Yue Chen[2,†], Yao Li[1,†], Lidong Guo[1,4], Yuxiaofei Wang[2], Xiawei Liu[1], Yaru Lin[2], Zhi Huang[1,5], Qun Liu[1,3], Wenjie Guo[1], Rui Zhang[1,3], Wandong Zhao[1], Langchao Liang[1,4], Xiaoyu Wei[6], Li Zhou[1], Xuebin Mao[1], Jiaqi Wang[1], Weijian Wu[1], Hongwei Pan[1], Tao Yang[7], He Zhang[1], Xiaoshan Su[1,3], Shanshan Liu[1], Wenwei Zhang[6], Longqi Liu[6], Søren Tvorup Christensen[3], Jifeng Fei[8], Xin Liu[1,6,9], Guangyi Fan[1,6,9], Hanbo Li[1,6,10], Ying Gu[6], Jian Wang[6], Huanming Yang[6], Gang Pei[2], Xun Xu[6,9], An Zeng[2,*], Mengyang Xu[1,6,*]

[1]BGI Research, Qingdao 266555, China

[2]Key Laboratory of Multi-Cell Systems, Shanghai Institute of Biochemistry and Cell Biology, Center for Excellence in Molecular Cell Science, Chinese Academy of Sciences, University of Chinese Academy of Sciences, Shanghai 200031, China

[3]Department of Biology, University of Copenhagen, DK-2100 Copenhagen OE, Denmark

[4]College of Life Sciences, University of Chinese Academy of Sciences, Beijing 100049, China

[5]School of Biology and Biological Engineering, South China University of Technology, Guangzhou, 510006, China

[6]BGI Research, Shenzhen 518083, China

[7]China National GeneBank, BGI Research, Shenzhen 518083, China

[8]Department of Pathology, Guangdong Provincial People's Hospital (Guangdong Academy of Medical Sciences), Southern Medical University, Guangzhou, Guangdong 510080, China

[9]State Key Laboratory of Agricultural Genomics, BGI Research, Shenzhen 518083, China

[10]Lars Bolund Institute of Regenerative Medicine Qingdao-Europe Advanced Institute for LifeSciences, BGI Research, Qingdao 266555, China.

†These authors contributed equally to this work.

*Correspondence: azeng@sibcb.ac.cn (A. Z.); xumengyang@genomics.cn (M. X.)

Abstract

Background: Understanding how organisms reconstruct complex tissue architectures following injury requires precise mapping of gene expression and cellular responses across space and time. Although planarians serve as a classic model for whole-body regeneration, capturing the continuous spatiotemporal dynamics of positional information and cell fate decisions at the organismal scale remains a significant challenge.

Results: Using high-definition spatial transcriptomics, we generated a four-dimensional atlas encompassing over 3.5 million cells from whole animals across eight distinct regeneration timepoints. This comprehensive dataset enabled the definition of 36 spatial domains and the tracing of body axis restoration, revealing that positional control genes recover through self-organizing dynamics analogous to an underdamped control system. We identified a transient injury-induced spatial domain termed the Anterior Regenerative Zone. This unique region is characterized by the convergence of epidermal, muscular, and neural lineages enriched with positional signals. Furthermore, we demonstrated that the transcriptional co-factor Mediator 8 is a critical regulator of this zone. Depletion of Mediator 8 impairs the formation of the Anterior Regenerative Zone, disrupts polarity establishment, and prevents successful blastema formation.~~establishment~~

Conclusions: Our study provides a holistic molecular and cellular reconstruction of whole-body regeneration, directly linking dynamic gene expression gradients to morphological restoration. The discovery of the Mediator 8-regulated Anterior Regenerative Zone highlights the importance of transient spatial domains in coordinating tissue repair. The resulting interactive atlas serves as a foundational resource for deciphering the logic of spatiotemporal patterning in

Formatted: Font: Bold

Formatted: Heading 2, Space Before: 0 pt, After: 0 pt, Line spacing: Double

Formatted: Line spacing: Double

Formatted: Font: Not Bold

regeneration.~~**Abstract**~~

~~Regeneration relies on precise spatiotemporal gene expression and cellular responses to establish tissue identity and body patterning. Using high-resolution Stereo-seq (715 nm) on 353 sections from 16 whole animals at 8 regeneration timepoints, we constructed a 4D spatiotemporal transcriptomic map of planarian regeneration. Our analysis captured 36 refined cell types from 3,508,004 segmented cells, enabling genome-wide transcriptional imputation of gene expression dynamics across body axes at cellular, tissue, and organismal scales. We identified dynamic positional gradients and distinct spatially distributed cell types during regeneration, including an injury-induced Anterior Regenerative Zone (ARZ). The ARZ exhibited enriched positional signals in epidermal, muscle, and neural cells and was regulated by Mediator 8, which is crucial for polarity remodeling and blastema formation. This study provides a comprehensive spatial molecular and cellular map of regenerative processes, highlighting injury-induced spatial domains and key regulatory factors in planarian regeneration. We also provide an interactive web portal, offering a valuable resource for exploring and analyzing regeneration mechanisms in a spatiotemporal context.~~

Keywords: Planarian regeneration, Spatial transcriptomics~~Stereo sequencing~~, Positional gradients, Regenerative zone, Single-cell atlas, Pattern formation

Background~~**Introduction**~~

Understanding the mechanisms of tissue and organ regeneration following injury is a fundamental biological question with profound implications for regenerative medicine, wound ~~injury~~ repair,

Formatted: Font: Bold

Formatted: Heading 2, Space Before: 0 pt, After: 0 pt, Line spacing: Double

Formatted: Font:

Formatted: Line spacing: Double

and aging. Regeneration involves a complex series of events, including local and systemic responses to injury, the restoration of positional information, and the formation of new tissue structures [1, 2]. While progress has been made in understanding these processes, several critical questions remain [3, 4]. How do cells spatially respond to injury, and how do molecular gradients influence tissue patterning? What roles do specific spatial ~~cell types~~domains, morphogen gradients, and injury-responsive regions play in regeneration across complex organisms? Furthermore, how can we capture and quantify these processes at the molecular level across an entire organism?

To address these questions, profiling organisms, cells, and genes across multiple spatial and temporal scales is crucial [5]. However, capturing continuous positional signals at molecular, cellular, and organismal levels ~~— especially in three-dimensional space and time —~~ remains a significant challenge, especially in three-dimensional space and time [6]. The complexity of tissue heterogeneity, large body sizes, and the preservation of cellular and molecular organization in extensive tissue sections complicate spatial transcriptomic analyses. Moreover~~Additionally~~, the absence of quantitative assays and frameworks to capture continuous positional signals at the transcriptome level in three-dimensional space and time presents an additional technical obstacle. This challenge is further compounded by the limited availability of classical model organisms that can fully regenerate their bodies. To date, a comprehensive three-dimensional molecular reconstruction of cellular architecture and morphogen gradients across an entire organism has not been achieved, limiting our understanding of the dynamic changes in cellular and molecular identities during regeneration.

Planarians, renowned for their exceptional regenerative abilities, serve as an ideal model for studying the spatial and temporal dynamics of tissue regeneration [3]. These bilateral metazoans possess a complex anatomy [7], including a brain, nerve cords, peripheral nervous system,

epidermis, intestine, muscles, excretory system, and a centrally located pharynx. Composed of a variety of cell typesdomains derived from three germ layers, planarians rely on pluripotent stem cells, or neoblasts, for constant tissue turnover and regenerationto generate and maintain their tissues [8, 9]. They utilize precise positional cues to guide body axis establishment formation and tissue patterning [10, 11]. Numerous genes involved in signaling pathways for body plan patterning have been identified, expressed in a complex spatial map across the dorsoventral (D/V), mediolateral (M/L), and anteroposterior (A/P)anterior-posterior, medial-lateral, and dorsal-ventral axes [12-15]. These genes, known as position control genes (PCGs), are largely expressed in muscle tissue and play a critical role in regulating positional information during regeneration [16]. However, the precise phenotypic outcomes associated with many of these genes remain poorly understood, and it is still unclear whether positional information is confined exclusively to muscle tissue [16]. The regenerative process requires cells to establish, record, and interpret positional information in order to rebuild the body's complex structure. This highlights the importance of profiling genes and cell typesdomains across multiple spatial and temporal scales. Although advances in single-cell RNA sequencing (scRNA-seq) [17-23] and spatial transcriptomics (ST) [24, 25] have enabled the profiling of cell typesdomains and gene expression patterns, these technologies still face limitations in achieving high spatial resolution at both the cellular and organismal levels. Furthermore, the mechanisms by which injury-induced local signals guide stem cells to reconstruct the body axis and regenerate fully functional three-dimensional structures remain poorly understood [3, 26]. Thus, there is a need for comprehensive analytical frameworks approaches that can capture the dynamic molecular and cellular events of regeneration across entire organisms.

In this study, we used Stereo-seq [27] and our custom framework [28] (Han et al., submitted)

to create an extensive atlas of 3,508,004 segmented cells from 353 sections of 16 complete planarians, spanning eight time points of whole-body regeneration. With a resolution of 715 nm, we generated detailed transcriptional and anatomical maps of the regeneration process, constructing positional and transcriptional gradients along the body axis. Our 4D transcriptomic atlas, annotated with 36 refined ~~cell types~~domains, provides a comprehensive view of gene expression dynamics across cellular, tissue, and organismal scales. Our findings offer key insights into the regenerative process. First, we reveal complete spatial gene expression patterns that define positional gradients along the body axes, tracking their dynamic spatiotemporal fluctuations following amputation~~changes during regeneration~~. Additionally, we identify an injury-induced Anterior Regenerative Zone (ARZ), marked by *ROD1*, which exhibits enriched positional signals in epidermal, muscle, and neural cells. The ARZ is regulated by Mediator 8 (~~*Med8*~~*med8*), which is crucial for polarity establishment~~remodeling~~, blastema formation, and overall regeneration. These results provide a comprehensive molecular and spatial map of regenerative processes, highlighting dynamic changes in regeneration-responsive cells, spatial domains, and key regulatory factors.

Data Description

To comprehensively map the spatiotemporal dynamics of whole-body regeneration, we employed high-resolution Stereo-seq (715 nm) on 353 cryosections derived from 16 *Schmidtea mediterranea* individuals across eight regeneration timepoints (0 hours to 14 days). This 4D reconstruction yielded 3,508,004 segmented cells, from which we annotated 36 distinct cell clusters and identified the injury-induced Anterior Regenerative Zone. This dataset enables genome-wide transcriptional imputation across body axes and provides a foundational resource for modeling morphogenetic gradients and regenerative patterning. Raw sequencing data are

Formatted: Font: Italic

Formatted: Font: Italic

Formatted: Font: Italic

Formatted: Font: Bold

Formatted: Heading 2, Space Before: 0 pt, Line spacing: Double

Formatted: Font: Not Bold

Formatted: Heading 3, Space Before: 0 pt, After: 0 pt, Line spacing: Double

Formatted: Font: Not Bold, Italic

Formatted: Font: Not Bold

deposited in the CNGB Nucleotide Sequence Archive under accession STT0000028, while processed data, code, and 3D visualizations are accessible via the PRISTA4D interactive portal (https://db.cngb.org/stomics/prista4d) and GitHub for unrestricted community reuse.

~~**Results**~~

Analyses

Reconstruction of ~~Planarian~~ planarian 4D spatiotemporal transcriptomes at single-cell resolution

To investigate the cellular and molecular dynamics of regeneration, we generated a comprehensive 4D atlas of gene expression and cellular changes during the regeneration process (~~Materials and~~ Methods). We focused on pre-pharyngeal amputations, which regenerate the head, tail, and pharynx over a two-week period (Fig. 1A). Using the Stereo-seq technique [27], which integrates tissue cryo-sectioning with *in situ* RNA sequencing at 715 nm resolution, we profiled gene expression~~s~~ at multiple stages of regeneration. Animals were collected at eight distinct time points~~—~~: 0 ~~hours~~, 12 ~~hours~~, and 36 hours post-amputation (hpa), and 3 ~~days~~, 5 ~~days~~, 7 ~~days~~, 10 ~~days~~, and 14 days post-amputation (dpa). For each time point, two animals were sampled, resulting in a total of 16 regenerating animals. These animals were sectioned along the dorsal-ventral axis to capture the entire organism (~~f~~Supplementary Fig. S1A). The 16 animals were processed into 10-µm-~~-~~thick sections, producing a total of 353 slices for spatial transcriptomics analysis using the Stereo-seq platform (Fig. 1A and ~~and~~ Supplementary F~~f~~ig. S1A).

To facilitate the generation of a comprehensive 3D reconstruction, we aligned and stitched

Formatted: Font: (Default) Times New Roman, 12 pt, Font color: Text 1

Formatted: Font: (Default) +Body (Calibri), 10.5 pt, Not Bold, Font color: Auto

Formatted: Space After: 0 pt, Line spacing: Double

Formatted: Font: Bold

Formatted: Heading 2, Line spacing: Double

Formatted: Heading 3, Space After: 0 pt, Line spacing: Double

Formatted: Font: Not Bold

Formatted: Line spacing: Double

the individual tissue sections (~~Materials and~~ Methods). This resulted in 3,508,004 segmented cells across the 16 reconstructed animals, with cell counts ranging from 58,450 to 432,197 per time point after quality control (UMIs per cell >50) (Fig. 1B, Supplementary ~~and~~ F~~f~~ig. S1A, and Supplementary ~~) (~~Table~~data~~ S1). This spatial transcriptomics atlas spans all eight time points, providing a high-resolution, 4D view of regenerating planarians at subcellular resolution (Fig. 1B and Supplementary F~~f~~ig. S1B). The dataset allows for the tracking of the spatial dynamics of regeneration-responsive genes and cellular interactions at various stages of regeneration. To validate the quantitative accuracy of our spatial transcriptomic atlas, we performed a correlation analysis between the Stereo-seq data (aggregated as pseudo-bulk) and public bulk RNA-seq data [29]. The high correlation coefficient (R = 0.8, Supplementary Fig. S1C) demonstrates the robust reliability and technical reproducibility of our spatial dataset. The dataset is publicly available via our searchable browser: https://db.cngb.org/stomics/prista4d (Supplementary Fig. S1D~~C~~).

To enhance the identification of biologically relevant tissue domains, we combined gene expression data with spatial information using the spatial proximity-based clustering (SPC) method. This method groups cells based on both transcriptional similarity and spatial proximity (~~Materials and~~ Methods). Using SPC, we identified 36 distinct spatial domains ~~cell clusters~~ (Fig. 1B)~~, each characterized by unique transcriptomic profiles and spatial distributions (fig S2, A and B)~~. Correlation analysis with published single-cell datasets revealed that the majority of these domains represent lineage-restricted populations (Supplementary Fig. S2, A, B and C~~B~~). Furthermore, comparison with published 10x ~~10~~Genomics~~X~~ Visium datasets confirmed consistent spatial transcriptional signatures across platforms (Supplementary Fig. S2D). Together, these systematic cross-platform validations confirmed the accuracy of our cell identities while highlighting the superior resolution of Stereo-seq in resolving fine-grained spatial heterogeneity

within broader tissue domains. A systematic comparison with published scRNA-seq datasets confirmed the consistency of these cell identities while highlighting the advantage of Stereo-seq in resolving spatial heterogeneity[28]. For example, our analysis revealed significant spatial heterogeneity in the parenchyma, which was subdivided into 11 distinct subclusters (Supplementary Ffig. S32A), consistent with previously identified heterogeneity [17, 22]. We also identified well-known tissue sub-populations, including epidermal progenitors, dorsal and ventral epidermal populations, and sub-populations of goblet cells and phagocytes within the intestine (Supplementary Ffig. S32B). Furthermore, we discovered a new spatially localized domain, Clu.31, within the blastema. Initially emerging in both the head and tail blastemas at 36 hours post-injury, this domain eventually became restricted to the head by the end of regeneration (fig. SSupplementary Fig. S32A), which we designated as the Anterior Regenerative Zone (ARZ).

For better tissue contour characterization, we generated tissue meshes for the intestines, pharynx, and neuronal regions. The spatial distributions of these clusters and tissue meshes were reproducible across two animals at each time point (Fig. 1B and fig. SSupplementary Fig. S1B), confirming the robustness of our data at the organismal level. Together, this 4D atlas provides a valuable resource for studying the temporal and spatial dynamics of gene expression and cellular coordination during whole-body regeneration.

Capturing tissue and organ remodeling and identifying genes responsive to regeneration

Formatted: Heading 3, Space After: 0 pt, Line spacing: Double

The comprehensive 3D reconstruction enabled precise measurements of tissue volume changes through the 4D dataset. Analyzing the length ratios along the dorsal/ventral (D/V), medial/lateral (M/L), and anterior/posterior (A/P) axes revealed that the D/V and M/L axes shortened, while the

Formatted: Line spacing: Double

A/P axis elongated during regeneration (Fig. 1C, top). Although the cell ~~count~~counts decreased, the average cell volume remained largely unchanged, suggesting that the observed volume changes were primarily due to a reduction in cell numbers (Fig. 1C, bottom). This finding aligns with the body-wide plasticity previously observed in planarians [30].

Using the 4D dataset, we tracked the regeneration of the pharynx, as well as the remodeling of the ~~intestine and~~ nervous system (Fig. 1D), with validation through fluorescent in situ hybridization (FISH) for pharyngeal (*foxA*) and neural markers (*pc2*) (Fig. 1E). Both the pharynx and central nervous system, particularly the cephalic ganglia, exhibited increased volume over time, while the intestine showed a decrease in size but underwent remodeling. The pharynx began to form between 3 and 5 dpa, while the central nervous system matured by 5 dpa (Fig. 1, D and E), reflecting the gradual remodeling in these organs.

To investigate the dynamic cellular responses during regeneration, we analyzed the proportions of different ~~cell types~~domains over time. We classified cell cluster dynamics into five patterns: continuous increase, initial increase followed by decrease, continuous decrease, initial decrease followed by increase, and unchanged (~~fig. S~~Supplementary Fig. S2C). Notably, dorsal epidermal progenitors (Epi.30), neural progenitors (Neu.28), and pharyngeal lineages (Pha.19 and Pha.29) exhibited a gradual increase (~~fig. S~~Supplementary Fig. S3~~2~~, C–D), reflecting the expansion of these specific lineages required for tissue reconstruction~~indicating an active wound response or the generation of new tissue~~. These results suggest that planarians may balance cell and organ proportions during regeneration, dynamically rescaling body proportions and restoring axial polarity.

Due to the fragility of blastema tissue, conventional FISH methods are challenging for capturing internal gene expression and spatial distribution in this region [31]. To overcome this

limitation, we hypothesized that digital segmentation of the blastema region, based on pigmentation intensity, would provide a more effective means of analyzing gene expression and cellular composition. Using image recognition algorithms, we segmented the animals into head blastema, tail blastema, and pre-existing trunk regions (~~fig. S~~Supplementary Fig. S3~~2~~E). Statistical analysis revealed positional heterogeneity in cellular responses. For instance, the pharynx (Pha.19) was initially localized to the tail blastema and later to the trunk, suggesting its centripetal migration ~~movement~~ toward the body~~'s~~ center. Additionally, the ARZ (Clu.31) was induced in both blastemas but persisted only in the head region (~~fig. S~~Supplementary Fig. S3~~2~~F). This segmentation highlights the potential of our virtual 4D data in identifying distinct cell subtypes that emerge at various stages and locations during regeneration.

To further investigate the molecular responses in finer regions, we divided the regenerating head and tail blastemas into three subdomains, ~~—~~proximal, middle, and distal, ~~—~~along with the trunk, and identified region-specific gene expression patterns through clustering (Fig. 1, F and G). Both the head and tail blastemas exhibited similar wound-healing and remodeling gene expression, with enrichment in genes associated with Wnt and BMP signaling pathways. However, different subdomains within these regions showed distinct gene expression profiles (Fig. 1H, Supplementary Table ~~data~~ S2). Additionally, comparison of differentially expressed genes within the same clusters or regions across various regeneration time points revealed temporal variations in gene expression patterns. For example, we observed that parenchymal ~~cell types~~domains and epidermal progenitors responded to injury within the first 12 hours post-amputation (hpa), while goblet cells and *cathepsin*$^+$ cells were activated between 12 hpa and 3 dpa. Neuronal cells and the ARZ (Clu.31) domain showed a response after 3 dpa (Fig. 1I, ~~data S~~Supplementary Table S2), highlighting distinct cellular and regional responses at different stages of regeneration.

In summary, the 4D atlas offers an in-depth view of tissue remodeling, dynamic changes in cellular localization, and the spatial distribution of cell populations throughout planarian regeneration.

Formatted: Font: 12 pt

Spatiotemporal dynamics of positional gradients during whole-body regeneration

Formatted: Heading 3, Space After: 0 pt, Line spacing: Double

Formatted: Font: Not Bold

In an accompanying study, we characterized ~~summarized~~ gene expression ~~pattern~~ patterns along body axes and identified genes with regional expression patterns in 3D intact planarians, which we proposed as spatially biased genes (SBGs) [28] ~~(Han et al., submitted~~) (Supplementary Table S3). Some of these genes, involved in patterning processes, were classified as positional control genes (PCGs) [10] (Supplementary Table S4). To investigate the dynamics of these SBGs during regeneration, we employed our 4D dataset to map gene expression across the entire organism. We hypothesized that injury would disrupt SBG expression, with recovery occurring gradually as regeneration progressed.

Formatted: Line spacing: Double

Formatted: Highlight

To test this, we analyzed the spatiotemporal patterns of SBGs by mapping their expression dynamics onto 16 canonical spatial clusters defined by normalized body length (100 bins) along the A/P axis using logistic regression (Fig. 2A, Supplementary Table S3). At 0 hpa, following injury, the physical loss of head and tail territories led to the disruption of anterior and posterior-specific clusters, whereas medial patterns remained stable. By 5 dpa, the overall gradient patterns had largely been restored to resemble those of uninjured individuals (Fig. 2A), highlighting the dynamic process of positional remodeling during regeneration. As regeneration proceeded, these spatial domains were progressively reconstructed, with gene expression patterns returning to their homeostatic states by 14 dpa. The complete gene composition for each spatial cluster is

documented in Supplementary Table S3.

~~we analyzed the spatiotemporal patterns of SBGs by mapping representative samples from each time point onto clusters along the A/P axis using logistic regression (Fig. 2A). Our results showed that, following injury, most SBG clusters were initially disrupted and then fluctuated like waves along the body axis. Over time, these patterns gradually stabilized, resembling the uninjured state, though at different rates. By 5 dpa, the overall gradient patterns had largely been restored to resemble those of uninjured individuals (Fig. 2A), highlighting the dynamic process of positional remodeling during regeneration.~~

Formatted: Line spacing: Double

Leveraging the quantitative nature of spatial transcriptomics, we further explored the spatiotemporal dynamics of SBGs. Principal component analysis (PCA) on known PCGs along the body axes of intact planarians revealed that genes expressed in the same regions clustered together (Supplementary Table S4). For example, *ndl-4* and *sfrp1* were specifically expressed in the head (~~fig. S~~Supplementary Fig. S4~~3~~A), confirming their region-specific patterns [32, 33]. This analysis also allowed us to quantify relative expression patterns, such as *ndk* in both the head and pharynx, and *wntA* in the pharynx (~~Fig. S~~Supplementary Fig. S4~~3~~B), consistent with established spatial distributions [14, 34-36].

Next, we mapped SBGs across multiple time points in PCA space to track their dynamic recovery during regeneration. Notably, several known PCGs, including *ndk*, *fz5/8-4*, *fz4-1*, and *wnt11-2 [14, 34]*, exhibited a reciprocal recovery pattern along the A/P axis. These genes initially showed higher expression compared to uninjured individuals, before gradually returning to baseline levels (Fig. 2B, bottom). Our analysis revealed that following the disruption caused by amputation, SBG expression did not simply ramp up or down linearly. Instead, these genes exhibited transient dynamic fluctuations along the body axis. We modeled this recovery as a

perturbation in a dynamic system, analogous to an underdamped mass-spring system (Fig. 2B, top), where the system is displaced from equilibrium (homeostasis) by amputation and subsequently driven back by a restoring force representing the gene regulatory network (GRN) [37]. This dynamic trajectory eventually restored the gene expression profile to its homeostatic state by 14 dpa (Fig. 2A). The PCA (Fig. 2B) further delineates this process into distinct biological phases. The early time points (0–36 hpa) cluster separately from later stages, representing an “Acute Injury Phase” characterized by a distinct wound response program that drives the system far from equilibrium. This is followed by a “Patterning Phase” (3–10 dpa), where the trajectory shifts direction and converges toward the homeostatic attractor [38]. This observation is consistent with the distinct, transient injury states recently identified in single-cell studies [17], confirming that early wound responses are transcriptionally distinct from the later morphogenetic programs that restore body plan fidelity.~~Following amputation, the disruption of PCG expression resembled a perturbation in a dynamic system, akin to an underdamped mass-spring system~~ [37]~~, where the system is displaced from equilibrium, oscillates, and then stabilizes. In line with this, some SBGs displayed wave-like expression along the A/P axis. This dynamic recovery trajectory eventually restored the gene expression profile to its homeostatic state at 14dpa (Fig. 2A).~~ We hypothesize that injury disrupts regional expression, and that the gene regulatory network may act as a restoring force, guiding the recovery of disrupted PCG expression through feedback mechanisms (Fig. 2B, top). Consistent with this model, our analysis showed that PCG expression was elevated between 3 and 10 dpa, and returned to baseline by 14 dpa upon completion of regeneration (~~Fig. S~~Supplementary Fig. S4~~3~~C), further supporting this recovery model.

To visualize the dynamic recovery of spatial patterns, we color-coded the expression patterns of known PCGs at each time point (~~Materials and~~ Methods). These patterns were restored by 12

~~hours~~hpa, 36 hpa~~ours~~, or 3 dpa (Fig. 2C), suggesting a temporal progression ~~transformation~~ in their recovery. Notably, spatial patterns were restored earlier than corresponding detectable changes in gene expression. For instance, the spatial pattern of *ndk* was restored by 12 hpa (Fig. 2C), while its expression began to increase only at 36 hpa (~~Fig. S~~Supplementary Fig. S4~~3~~C~~,~~ and Supplementary ~~data~~ Table S3). ~~This~~ These temporal dynamics suggest~~s~~ that spatial patterning may influence the regulation of gene expression.

Motivated by the underdamped response of PCG trajectories (Fig. 2B) and the temporal hierarchy between spatial pattern recovery and gene expression (Fig. 2C), we hypothesized that SBG expression dynamics could be modeled mathematically. Extending the self-organizing model proposed for the Wnt pathway along the A/P axis [32], we applied the Gierer-Meinhardt model of a simple activator-inhibitor system [39] within the Turing system framework [40] to predict global changes in SBG expression during regeneration. While the model traditionally describes the diffusion and reaction kinetics of secreted morphogens, such as the *Wnt/Notum* pair, we applied it here to characterize the expression of downstream TFs, such as *hox4b* and *EGR1*. We propose a Readout hypothesis where these nuclear factors do not diffuse themselves but act as high-resolution spatial proxies that interpret the primary, diffusive morphogen gradients. By analyzing spatial gradients in exponential form and excluding the influence of the pharynx-enriched genes, we separated independent temporal and spatial components to simulate changes in activator and inhibitor expression at each time point during regeneration (Fig. 2D, top). ~~The predictions from this model were largely consistent with the expression values measured in the Stereo-seq data for genes such as *ARNT* [41], *Ndk* [14], *EGR1*, *hox4b*, *Nfya*, and *glra1* (Fig. 2D, bottom), suggesting that the model offers a reasonable representation of the observed dynamics.~~ Our findings demonstrate that the predictions of trajectories of these “readout” genes, such as *ARNT* [41], *Ndk*

Formatted: Font: Italic

Formatted: Font: Italic

Formatted: Font: Italic

Formatted: Font: Not Italic

Formatted: Font: Not Italic

Formatted: Font: Not Italic

[14], *EGR1*, *hox4b*, *Nfya*, and *glra1* (Fig. 2D, bottom, Supplementary Table S4) closely follow theoretical activator-inhibitor kinetics, reinforcing the conclusion that planarian regeneration is guided by scalable, self-organizing patterning systems.

Formatted: Font: Not Italic

We next explored whether SBGs were enriched in specific ~~cell types~~domains or regions. By quantifying the number of SBGs enriched in each SPC at different stages of regeneration, we observed that SBGs were expressed across multiple lineages, including muscle, epidermal, and neural lineages (Fig. 2E). Interestingly, the ARZ domain (Clu.31) displayed characteristics from several lineages and contained the highest number of SBGs (Fig. 2E). This observation suggests that the ARZ may play a role in remodeling and maintaining polarity. Overall, our 4D analysis provides a detailed view of the spatiotemporal dynamics of SBG expression during regeneration, offering support for a model based on self-organized reaction-diffusion patterns.

Characteristics of the injury-induced Anterior Regenerative Zone (ARZ) enriched in SBGs

Formatted: Font: Not Bold

Having demonstrated that the ARZ (Clu.31) exhibits injury-induced anterior localization and is enriched in SBGs, we hypothesized that the ARZ plays a crucial role in regulating PCGs and maintaining regenerative polarity during regeneration, similar to the proposed function of the organizer [42]. We further sought to characterize this region. At homeostasis, the ARZ is localized to the anterior side, with signatures of three distinct lineages (Fig. 3A and ~~data S~~Supplementary Table S5). Gene expression analysis within this domain revealed enriched expression of SMED30003831 (*smed03831 or Rod1*), *caveolin3*, and SMED30001640 (*smed01640)* (~~fig. S~~Supplementary Fig. S5~~4~~A and ~~data S~~Supplementary Table S2). Notably, the ARZ spans both the peripheral epidermal and subepidermal areas of the head, distinguishing it from the *Equinox*-

expressing wound epidermis [43] (~~fig. S~~Supplementary Fig. S5~~4~~A). Gene ontology (GO) analysis of ARZ-enriched genes identified processes related to epidermal differentiation, muscle contraction, and neural development (~~fig. S~~Supplementary Fig. S5~~4~~B). Co-FISH experiments with the ARZ marker *smed03831* and lineage markers confirmed that the ARZ encompasses epidermal (*agat-1*) (Fig. 3B), muscular (*collagen*) (Fig. 3C), and neural (*pds*) cells (Fig. 3D), solidifying its tri-lineage characteristics. These findings suggest that the ARZ is a co-regulated, regeneration-responsive region.

To track the temporal dynamics of the ARZ, we analyzed its spatial location throughout regeneration. At 36 hpa, the ARZ was present as scattered clusters near the ventral wound sites in both head and tail fragments. By 3–5 dpa, these cells converged towards the midline and expanded to cover the wound area, coinciding with wound closure and blastema formation. By 10 dpa, ARZ cells diminished in the tail but persisted in the head region (Fig. 3E, ~~fig. S~~Supplementary Fig. S5~~4~~C). Measurements of the distance from the wound surface revealed that the ARZ gradually approached the amputation site during the first five days, with an increase in cell number within the zone (Fig. 3F). This was further confirmed by staining for the ARZ marker *smed03831* in the regenerating head region (Fig. 3G).

To investigate the putative origin of ARZ cells, we traced their pseudotime trajectory during regeneration using Monocle [44]. This analysis revealed three distinct branches (Fig. 3H), each enriched for genes specific to epidermal, muscle, or neuronal lineages (Fig. 3I, ~~Fig. S~~Supplementary Fig. S5~~4~~D). The earliest reappearance of epidermal signatures at 12 hpa marked the emergence of the ARZ. To validate this injury-induced differentiation dynamics, we profiled the temporal expression of the late epidermal progenitor marker *agat-1* within the ARZ. We observed a progressive enrichment of *agat-1* starting from 36 hpa and peaking at 3 dpa

Formatted: Font: Italic

Formatted: Font: Italic

(Supplementary Fig. S5~~4~~E), mirroring the kinetics of active blastema differentiation rather than static tissue maintenance [17, 45]. This was~~,~~ followed by the emergence of muscle and neuronal markers at 3 dpa, coinciding with blastema formation (Fig. 3J). By 14 dpa, the ARZ cellular composition had largely reverted to epidermal cells, resembling the homeostatic state (Fig. 3J). The expression of ARZ-enriched genes aligns with these cellular dynamics (~~data S~~Supplementary Table S5), further supporting the coordinated and timely assembly of the ARZ domain during regeneration.

Cellular composition and regulation of the polarity-enriched ARZ domain

Having demonstrated the coordination between ~~the~~ ARZ formation and regeneration, we next sought to investigate the cellular components that control ~~the~~ARZ formation~~ of the ARZ~~. Our focus was on the epidermis, as it constitutes ~~is~~ the earliest cell type to emerge within the ARZ. To identify the regulatory factors involved in ARZ formation, we employed RNA velocity, a method that distinguishes between unspliced and spliced mRNAs [46], to predict the putative trajectory of the SPC clusters in epidermal lineages (~~fig. S~~Supplementary Fig. S6~~5~~A). The velocity vectors indicated that epidermal cells in the ARZ primarily originate from the ventral epidermal lineage (Epi.1) (~~fig. S~~Supplementary Fig. S6~~5~~, A and B). To visualize ARZ formation in a spatiotemporal context, we projected 3D spatial data onto 2D maps of the head blastema at various time points using ~~UV~~ via unwrapping (Fig. 4A), a dimensionality reduction technique~~ used in 3D modeling to~~ transforming a 3D model's surface into a 2D plane (see ~~Materials and~~ Methods). ~~Applying~~ ~~p~~Pseudotime trajectory analysis of~~n~~ the unwrapped 2D map ~~after UV unwrapping~~ revealed a cell-state transition from ventral epidermal cells toward the ARZ, while left and right dorsal-ventral

Formatted: Heading 3, Space After: 0 pt, Line spacing: Double

Formatted: Font: Not Bold

Formatted: Line spacing: Double

boundary cells on both sides of the head blastema moved toward the anterior pole (Fig. 4, B to D). These observations suggest that ARZ formation involves interactions between ventral and dorsal epidermal cells. Indeed, comparing spatial maps at 36 hpa and 3 dpa revealed~~s~~ the expansion of the ARZ domain from both ventral and dorsal sides, coinciding with wound closure (Fig. 4E).

To explore the role of the ARZ as a signaling-rich domain ~~in positional control~~ during regeneration, we conducted trajectory analysis to identify potential regulators involved in cell differentiation within the ARZ (Fig. 4F). While genes such as *smed03831* and *caveolin3* serve as definitive markers of the differentiated ARZ, we sought to identify the upstream drivers governing its formation. Notably, the mediator complex subunit 8 (*med8*) emerged as an ~~early expressed~~early-expressed gene along the pseudotime trajectory (Fig. 4F), preceding the expression of structural markers. Med8 is an essential component of the mediator complex, playing a critical role in transcription regulation [47]. The planarian ~~*med8*~~*med8* homolog is evolutionarily conserved and shares ~~close~~ high sequence identity ~~homology~~ with orthologs in other species (~~fig. S~~Supplementary Fig. S6~~5~~C). In the homeostatic state, ~~*med8*~~*med8* is highly expressed in neoblasts (~~fig. S~~Supplementary Fig. S6~~5~~D), with enrichment observed across multiple neoblast subpopulations (~~fig. S~~Supplementary Fig. S6~~5~~E-F). Following injury, ~~*med8*~~*med8* expression increased in the wound area at 12 hpa, prior to the emergence of *smed03831*$^{+}$ cells at 1.5 dpa (Fig. 4G). This suggests that ~~*med8*~~*med8* may regulate ARZ formation. The ~~C~~co-expression of ~~*med8*~~*med8* ~~with~~ and *smed03831* in a substantial ~~significant~~ portion of blastema cells after 1.5 dpa further supports its regulatory role in ARZ formation (Fig. 4, G and H). To assess the functional role of ~~*med8*~~*med8*, we performed RNA interference (RNAi) knockdown experiments (~~fig. S~~Supplementary Fig. S6~~5~~G) and measured the expression of the ARZ marker *smed03831*. Knockdown of *med8* resulted in impaired blastema regeneration by 5dpa (Fig. 4I) and a significant

Formatted: Font: Italic

Formatted: Font: Italic

reduction in the number of *smed03831*$^{+}$ cells at 3 and 5 dpa (Fig. 4J), suggesting that *med8* is required for ARZ formation.

Having shown that *med8*(RNAi) hinders ARZ reconstruction (Fig. 4J), we next investigated the specific stage at which *med8* influences ARZ formation by examining gene expression at different regeneration time points. Given that the ARZ is enriched with various PCGs (Fig. 2E), we hypothesized that the failure in ARZ reconstruction would prevent the re-establishment of anterior polarity~~*med8* knockdown might disrupt polarity remodeling~~. To test this, we examined the expression of the anterior pole marker *sfrp-1* [13, 48], which was significantly reduced upon *med8* knockdown (Fig. 4J), suggesting a failure to generate the anterior pole identity during regeneration~~defects in pattern formation during regeneration~~. In control animals, *smed03831*$^{+}$ cells were enriched at the wound site at 1.5 dpa and fully covered the wound, followed by the appearance of *sfrp-1*$^{+}$ cells at the anterior pole by 3 dpa (Fig. 4J). This temporal sequence suggests that ARZ formation precedes anterior pole formation. In contrast, *med8*(RNAi) animals exhibited impaired ARZ formation and a reduction in *sfrp-1*$^{+}$ cells (Fig. 4J), indicating an inability to re-establish anterior identity.~~a disruption in patterning.~~ By 7 dpa, ARZ formation was completely disrupted in *med8*(RNAi) animals, and regeneration failed (Fig. 4K), linking ARZ formation to successful regeneration. Together, these findings support the idea that *med8*-mediated ARZ formation is essential for providing the cellular basis for pole formation~~proper pole formation~~ during regeneration.

Med~~iator~~ 8 **is required for ARZ lineage specification to support blastema development~~regulates ARZ formation to control differentiation and blastema development~~**

Formatted: Font: Not Italic

Formatted: Font: Italic

Formatted: Heading 3, Space After: 0 pt, Line spacing: Double

Formatted: Font: Not Bold

To identify the transcriptional programs mediating changes in the ARZ, we conducted scRNA-seq on *med8* and control RNAi animals with amputated tails undergoing head regeneration. By integrating the data from both groups, we identified known cell lineages, including stem cell populations and eight distinct domains~~cell types~~ (Fig. 5A), consistent with previous findings [8, 22]. Notably, *med8* RNAi expanded~~led to an increase in~~ the neoblast population~~,~~ while reducing the proportions of ~~lineages~~ ARZ-associated lineages~~with the ARZ,~~ ~~—~~specifically neural, muscle, and epidermal cells ~~— were reduced~~ (Fig. 5B). This observation was further confirmed by pseudotime trajectory analysis using Monocle, which revealed similar reductions in the differentiation of these cell lineages (~~fig. S~~Supplementary Fig. S7~~6~~A). Label transfer analysis ~~,~~ ~~performed to~~ matching ARZ cells across~~in~~ the scRNA-seq dataset~~,~~ confirmed a decrease in ~~cells representing~~ each ARZ cellular component in *med8* RNAi animals (Fig. 5C and ~~fig. S~~Supplementary Fig. S7~~6~~B).

Formatted: Line spacing: Double

Given that the ARZ is enriched for genes involved in polarity formation, we next examined whether the differentiation blockade caused by *med8* RNAi led to a loss of cells expressing anterior and posterior pole markers~~*med8* RNAi caused global defects in the expression of anterior and posterior pole markers~~. Analysis of A/P axis marker gene expression in the scRNA-seq data revealed that *med8* RNAi led to a reduction in the polarity signature score within ARZ cells, but not in other ~~cell types~~domains (Fig. 5D and ~~fig. S~~Supplementary Fig. S7~~6~~C). Further analysis showed a decrease in both anterior and posterior markers (Fig. 5E). Whole-mount in situ hybridization (WISH) confirmed reduced expression of posterior markers, including *wnt1* and *Wnt11-1 [36]*, though body-wide polarity was not completely disrupted in *med8* ~~*med8*~~ RNAi animals (Fig. 5, F and G), suggesting that the observed changes are linked to regenerative growth. These results indicate that *med8* RNAi impairs ARZ formation, a prerequisite for establishing

Formatted: Font: Italic

positional landmarks within the blastema during regeneration~~ and alters global positional information within the blastema during regeneration~~.

To investigate how *m~~m~~ed8* affects ARZ formation, we examined the expression of ARZ markers within the blastema. The spatial extent of m~~M~~ajor ~~cell types~~domains in the ARZ, including epidermal (*agat-1*) and muscle (*collagen*) cells, was~~ere~~ reduced in *med8* ~~*med8*~~ RNAi animals (Fig. 5H). Additionally, the expression of transcription factors essential for the differentiation of neural (*tcf/lef-1*) [49], epidermal (*p53*) [50], and muscle (*dmrt2*) cells was diminished upon *med8* knockdown (~~Fig. S~~Supplementary Fig. S7~~6~~D), indicating that *med8* ~~*med8*~~ is crucial for maintaining the transcriptional programs associated with ARZ cell fate [51]. In line with this, gene expression analysis in neoblasts revealed downregulation of pathways related to stem cell division ~~and~~ as well as neural and epidermal fate determination (~~Fig. S~~Supplementary Fig. S7~~6~~E). To quantify this defect at the cellular level, we calculated the differentiation efficiency across lineages in our scRNA-seq dataset. We found that despite an accumulation of undifferentiated neoblasts, the efficiency of generating lineage-committed progenitors was severely compromised for the neural, muscle, and epidermal domains (Supplementary Fig. S7~~6~~F), confirming a broad differentiation blockade. Furthermore, FISH staining at 3 dpa and 5 dpa demonstrated reduced expression of markers for epidermal and muscle cells in the blastema region (Fig. 5, H and I), suggesting impaired cell fate decisions in ARZ cells. We also observed decreased expression of neural markers in ~~*med8*~~ *med8* RNAi animals (Fig. 5J), further confirming the disruption of ARZ lineage specification and impaired head regeneration.

Finally, we assessed whether ~~*Med8*~~*med8*-mediated ARZ formation is required for homeostasis. While short-term *med8* ~~*med8*~~ RNAi treatment caused minimal phenotypic changes in homeostatic animals, prolonged *med8*, ~~med8~~ knockdown ~~RNAi~~ led to head regression (~~Fig.~~

Formatted: Not Highlight

Formatted: Not Highlight

Formatted: Not Highlight

Formatted: Not Highlight

Formatted: Font: Not Italic

Formatted: Font: Not Italic

~~S~~Supplementary Fig. S7~~6~~G~~F~~), suggesting that sustained loss of ARZ function results in homeostatic defects. In summary, our data support the role of *med8* ~~*med8* regulated ARZ~~ in controlling blastema growth by enabling the specification of the ARZ-associated lineages~~regulating positional information~~, which is essential for proper tissue regeneration and the maintenance of homeostasis.

Discussion

~~Discussion~~

Understanding the full spectrum of spatial information and the principles governing pattern formation during regeneration in tissues and organs remains a significant challenge. This complexity is driven by the intricate tissue geometry, the large size of multicellular organisms, and the limited number of model organisms capable of regenerating entire tissues. Additionally, the absence of techniques capable of capturing high-resolution spatial transcriptomic data across an entire organism in 3D over time further complicates this challenge. In this study, we applied high-resolution Stereo-seq (715 nm) to planarians to reconstruct the 4D spatiotemporal landscape of whole-body regeneration. Our 4D dataset addresses several~~some of the~~ limitations of traditional techniques, ~~such as~~including ~~resolution,~~ low-throughput FISH assays~~,~~ and 2D single-slice-level spatial transcriptomics, providing a holistic and high-resolution view of genes~~, cell types,~~ and spatial domains before and during regeneration. While recent spatial transcriptomics methods provide spatial context [24, 25, 52], they often lack full 3D or single-cell resolution, limiting the ability to comprehensively profile morphogen gradients and ~~cell types~~domains across entire organisms over time. In contrast, our 4D regeneration atlas offers a high-resolution, time-resolved framework for analyzing regenerative dynamics. Mining ~~Exploring~~ our dataset allowed for the recovery of single-cell transcriptomes at the spatiotemporal ~~temporospatial~~ level, enabling the

Formatted: Not Highlight

Formatted: Font: Bold

Formatted: Heading 2, Space Before: 0 pt, Line spacing: Double

Formatted: Font: Not Bold

Formatted: Heading 3, Line spacing: Double

Formatted: Indent: First line: 0 ch, Line spacing: Double

visualization of gene expression patterns, positional signals, and cell type distributions across multiple scales. This analysis revealed morphogen gradient gene dynamics, identified regenerative domains, and highlighted key regulatory factors. The full dataset is available through our online resource: https://db.cngb.org/stomics/prista4d.

By leveraging the complete repertoire of SBGs across four dimensions, we examined gene expression across anatomical body regions and scales, particularly within the delicate blastema region, allowing for quantitative analyses across multiple body regions. We confirmed known PCGs and identified potential new PCG candidates, facilitating the modeling of morphogenetic gradients using the Turing reaction-diffusion model. While the Turing system has been proposed for fission and regeneration [32, 40, 53], its applicability to modeling planarian regeneration remains unclear [54]. The temporal dynamics of SBGs suggest that the restoration of positional identity functions as a self-organizing system. Rather than simple oscillations, the recovery follows an underdamped trajectory where the initial injury response (0–36 hpa) creates a significant displacement from the transcriptomic baseline, followed by a directed convergence during the patterning phase (3–10 dpa). This "overshoot and return" dynamic supports a model where the GRN acts as a restoring force, progressively dampening the perturbation introduced by amputation until the homeostatic equilibrium is regained. Our real dataset supports simulation predictions, with specific genes exhibiting consistency between modeled and observed datasome genes showing consistency with actual data. This rich dataset and quantitative approach provide a foundation for studying scalable, self-organized pattern formation in more detail, offering a framework for understanding how positional information is re-established during regeneration. Integrating high-resolution spatial transcriptomics with single-cell analysis, our study offers a valuable resource for investigating how positional information is maintained, disrupted, and

Formatted: Line spacing: Double

interpreted across different tissues and ~~cell types~~domains following injury. This dataset bridges the gap between molecular, cellular, and morphological aspects of regeneration, offering a comprehensive multimodal view of whole-body regeneration dynamics in organisms. This supports a hierarchical model where global self-organizing gradients are interpreted by local gene regulatory networks to restore tissue identity. These findings align with the perspective that regeneration relies on the re-establishment of organizers that coordinate tissue repair through these dynamic molecular gradients [42, 55].

Muscle cells have long been recognized as the primary conveyors of positional cues in adult planarians [16]. However, our 4D dataset expands this view, identifying non-muscular lineages, such as neuronal and epidermal cells, as contributors to the regenerative positional landscape. This suggests that pattern remodeling involves a multi-lineage process, with multiple cell types participating in encoding, reading, and interpreting positional information [56, 57]. We comprehensively profiled SBGs, which extend beyond PCGs, to define a systemic patterning system involving genome-wide spatial regulation. Since regenerative patterning involves both global polarity and local fate specification, most SBGs may not show defects in polarity but could alter cell fate [33, 58, 59]. The temporal dynamics of different SBG classes suggest that encoding and interpreting positional gradients is a distributed and hierarchical process, coordinated among various ~~cell types~~domains and exhibiting self-organizing properties within the organism.

The distinct temporal lag between the restoration of spatial patterning such as *ndk* asymmetry at 12 hpa and the subsequent transcriptional surge at 36 hpa implies a hierarchical “Pattern-then-Amplify” regulatory logic. We propose that the immediate post-injury phase involves unmasking of positional identity within pre-existing tissues. As muscle cells serve as the primary source of positional instructions, surviving tissue at the wound boundary utilizes stable landmarks to rapidly

Formatted: Line spacing: Double

Formatted: Font: Italic

reset the anatomical coordinate system [16]. This process is likely orchestrated by early signaling modulators, such as *Ptpn11* that fine-tune wound response pathways and *Wnt* dynamics prior to significant transcriptional activation [60]. Consequently, a precise spatial blueprint is established at low transcriptional levels by 12 hpa to spatially constrain the subsequent proliferative burst. The later peak in expression at 36 hpa thus reflects the amplification of this established pattern to support the massive cellular demands of blastema formation, effectively decoupling the definition of “where to regenerate” from the execution of “how much to grow” [61].

Analysis of SBG distribution revealed a specific spatiotemporal domain, Clu.31, which we define as the ARZ. While this domain contains mechanosensory neurons and epithelial cells characteristic of the anterior peripheral nervous system, our data suggest it represents a dynamic cellular neighborhood [17, 52] where the intercalating peripheral nervous system integrates with the dorsal-ventral boundary epithelium and body wall muscle to orchestrate regeneration. Specifically, the transient appearance of this domain in the tail blastema (Fig. 3E) and its dynamic compositional shifts, transitioning from an initial injury-induced epidermal state to a multi-lineage hub before resolving (Fig. 3H-J), distinguish it from a static anatomical structure. The temporal dynamics of *agat-1* expression within this domain further mirror transient regeneration-activated cell states (TRACS) described in other contexts [17, 45], supporting the hypothesis that the ARZ functions as a transient regenerative organizing center rather than solely representing the regenerating nervous system. From an evolutionary perspective, the presence of this spatiotemporally coordinated domain is functionally reminiscent of the apical epithelial cap (AEC) observed in vertebrate appendage regeneration [62]. Although anatomical differences exist, particularly the integral role of neural components in the planarian ARZ, the principle of a transient signaling niche established by the convergence of epithelial and sub-epithelial tissues [63, 64]

Formatted: Font: Italic

Formatted: Font: Italic

Formatted: Font: Italic

appears conserved. Similar to the AEC, the ARZ exhibits conserved molecular marker gene expression involving the Wnt/β-catenin and FGF pathways [13, 14, 48]. Further comparative analyses will be required to determine if the specific molecular circuits governing these cellular neighborhoods are homologous across metazoans [42].

Analysis of SBG distribution revealed a multi-lineage mixing domain, which we define as the Anterior Regenerative Zone (ARZ, Clu.31). This region is characterized by high expression of SBGs and dynamic changes in cellular proportions. Notably, we identified ROD1 (SMED30003831), a neuronal-enriched gene, as a marker of the ARZ, suggesting the presence of distinct cell types within this domain. The extent of the ARZ appears to surpass that of the anterior pole [65], with significant changes in intrinsic cell types, indicating that it may function as a co-regulatory domain. The relationship between the ARZ and the anterior pole remains unclear, and whether the ARZ possesses inductive activity warrants further investigation [66]. From an evolutionary perspective, the presence of this spatiotemporally conserved regenerative domain (ARZ) is reminiscent of the apical epithelial cap (AEC) observed in vertebrate appendage regeneration [62]. Our findings suggest potential structural and functional similarities between the planarian ARZ domain and the AEC of regenerating axolotls, including the conservation of molecular marker gene expression (e.g., Wnt/β-catenin and FGF pathways) [13, 14, 48]. In axolotls, the blastema forms beneath the thickened multilayered AEC, where cells differentiate and grow to replace lost structures [63, 64]. While we observed parallels in blastema formation and differentiation, planarians exhibit intricate temporospatial cellular dynamics during regeneration. Further comparative and functional analyses across metazoans could help clarify whether specific positional circuits and molecular programs are evolutionarily conserved across species [42].

Our findings using *mMed8*(RNAi) as a proxy suggest that epidermal, muscular, and neural

Formatted: Font: Not Italic

cells within the ARZ likely contribute to positional information for blastema induction. The persistence of this domain in adult planarians may help explain their homeostatic maintenance, providing insights into the regulation of regeneration [42, 61]. We further demonstrate explore that Med8med8, a subunit of the Mediator complex acting as a bridge between transcription factors and RNA polymerase II [47], modulates key genes related to epidermal, muscle, and neural specification within the regenerative domain. The Mediator complex is known for its role in maintaining stem cells, as well as in lineage-specific differentiation [51]. While the loss of Smed-*med14* specifically affects stem cell populations, the loss of *Med8* *med8* does not [65], suggesting that distinct Mediator components have different requirements for stem cell function in planarians. Our findings build upon previous studies and demonstrate that *Med8* *med8* is important for the differentiation of stem cells into neural, muscle, and epidermal lineages within the ARZ region, thereby ensuring the production of as well as for generating proper regenerative patterning signals. This suggests that Mediator, in conjunction with transcription factors [66-71], may be involved in establishing the epigenetic landscape necessary for lineage commitment and cell fate transitionsregenerative patterning.

The observation that *med8* knockdown markedly reduces the expression of polarity markers without causing gross disruption of the whole-body AP axis (e.g., double-head formation) warrants further discussion. Our single-cell and lineage analyses suggest that this discrepancy likely stems from the hierarchical role of *med8* in cell fate specification rather than direct gradient scaling. Since *med8* is required for the differentiation of specific ARZ lineages (epidermal, muscle, and neural), the observed reduction in polarity markers (e.g., *sfrp-1*, *wnt1*) reflects a loss of the signal-producing cells themselves, rather than a simple downregulation of gene expression within an intact tissue. Furthermore, *med8* knockdown leads to a symmetric reduction in both anterior (*sfrp-*

Formatted: Font: Not Italic

Formatted: Font: Italic

1) and posterior (*wnt1*, *wnt11-1*) signals. This balanced reduction likely preserves the relative antagonism between the anterior and posterior poles, preventing the dominance of one pole over the other that typically drives ectopic structure formation. Finally, while gross morphology is maintained in the short term, we noted that prolonged *med8* RNAi eventually leads to head regression, confirming that *med8*-mediated cellular turnover is indeed essential for the long-term maintenance of cell populations that harbor positional landmarks of the global body axes.

Potential implications

Despite the advantages of our 4D approach, specific ~~some~~ limitations remain. First, the sequencing depth is lower compared to scRNA-seq, which may impact the detection of rare cell populations. Second, biological replicates are limited~~there are limited biological replicates~~ due to the technical challenges of generating whole-organism 4D~~-ST~~ data. Additional replicates and validation will be required to further assess the robustness of morphogenetic gradients across individuals. Future efforts should focus on increasing sequencing depth, expanding the number of biological replicates, and incorporating complementary approaches to validate the dynamics of gene regulation. Nevertheless, integrating ~~our~~ these data with gene perturbation and longitudinal imaging studies will enable us to directly assess the functional contributions of specific positional signals in guiding pattern remodeling and regeneration.

Our study establishes a framework for a ~~four-dimensional~~4D~~,~~ high-resolution atlas of gene expression dynamics throughout whole-body regeneration. By combining spatial and temporal transcriptomic data, we provide a novel framework for understanding the principles governing regenerative patterning, advancing both regenerative biology and spatial transcriptomics methodologies. This comprehensive dataset serves as a valuable resource for future studies,

Formatted: Font: Italic

Formatted: Font: Italic

Formatted: Font:

Formatted: Heading 2, Indent: First line: 0 ch, Line spacing: Double

Formatted: Font: Bold

Formatted: Indent: First line: 0 ch, Line spacing: Double

Formatted: Line spacing: Double

enabling researchers to explore positional information, tissue remodeling, and the regulation of regeneration in biological systems.

~~**Materials and Methods**~~

Methods

Animal culture

Asexual *Schmidtea mediterranea* (strain CIW4) were maintained at 20 °C in a recirculating 1×~~X~~ Montjuic salts solution without antibiotics, following a previously described protocol [72]. The animals were routinely fed beef liver. For experimental procedures, the animals were transferred to static culture and starved ~~fasted~~ for at least 7 days.

Gene cloning and RNAi feeding

Genes of interest were cloned from a CIW4 cDNA library into the pPR-T4P vector as previously described [73]. The resulting plasmids were used to produce dsRNA for RNA interference (RNAi). RNAi was performed following established protocols for gene knockdown [74]. Briefly, bacterial pellets expressing the dsRNA were mixed with fresh beef liver paste in a 4:1 ratio. EGFP dsRNA was used as a control. Animals were fed every 3 days for a total of four or six RNAi feedings. After the final RNAi feeding, animals were amputated 3 days later to collect samples at various stages of regeneration.

In situ hybridizations

Whole-mount *in situ* hybridizations were conducted following previously established protocols

Formatted: Indent: First line: 2 ch, Line spacing: Double

Formatted: Indent: First line: 0.5", Line spacing: Double

Formatted: Font: Bold

Formatted: Heading 2, Space Before: 0 pt, Line spacing: Double

Formatted: Line spacing: Double

Formatted: Font: (Default) Times New Roman, 12 pt, Font color: Text 1

Formatted: English (United States)

Formatted: Line spacing: Double

Formatted: Font: Italic

[31, 75]. In short, the mucus from the animals was removed using 5% NAC in PBS, and then fixed for 1 hour in 4% formaldehyde (FA) in PBSTx (0.5%). The animals were bleached with formamide and incubated with proteinase K (2 μg/mL, AM2546, ThermoFisher) for 10 minutes. After a 2-hour pre-hybridization step, the hybridization was performed at 56 °C for over 16 hours. Following extensive washes, the antibody signal was amplified using the Tyramide Signal Amplification system. Tissue clearing was achieved using ScaleA2 to reduce background noise [31].

Sample fixation and section preparation for Stereo-seq

Sample fixation was carried out using a modified version of a previously described protocol [76]. In short, animals were relaxed in 0.66 M $MgCl_2$ for 1 minute, followed by fixation in Methacarn solution (6 mL methanol, 3 mL chloroform, 1 mL glacial acetic acid) for 10 minutes. After fixation, the animals were rinsed in methanol three times, rehydrated in 50% methanol in PBS for 5 minutes, and then cryoprotected dehydrated in 20% sucrose in PBS for two cycles. The cryoprotected dehydrated tissues were embedded in pre-cooled OCT, frozen with dry ice, and stored at -80 °C until cryosectioning. Following embedding, the specimens were photographed under a stereomicroscope to acquire the brightfield microscopy images. These images documented the morphological features and macroscopic pigmentation patterns of each specific animal, serving as the anatomical reference for downstream spatial region segmentation and data alignment. Tissues were equilibrated in a -20 °C freezing microtome for 30 minutes prior to sectioning. RNA quality from cryosections was assessed using an Agilent 2100 Bioanalyzer. The cryosections of *Schmidtea mediterranea* were cut serially at 10 μm intervals using a Leica CM1950 cryostat. Each section was placed onto a Stereo-seq chip, incubated for 3 minutes at 37 °C on a Thermocycler Adaptor, and then fixed in methanol at -20 °C for 40 minutes.

ssDNA staining and imaging of Stereo-seq slides

Prior to tissue permeabilization, sections on the Stereo-seq chip were stained with a nucleic acid dye (Thermo Fisher, Q10212) to visualize single-stranded DNA (ssDNA). The stained sections were then imaged using a Leica DM6M microscope. The images were stitched together and processed using the Leica Application Suite X software.

Library construction and sequencing of Stereo-seq data

The library construction and sequencing protocols for Stereo-seq have been previously described [27]. In summary, tissue sections were first washed with 100 μL of 0.1× saline-sodium citrate buffer (SSC, Thermo, AM9770) containing 0.05 U/μL RNase inhibitor (NEB, M0314L) to remove any remaining staining solution. Sections were then permeabilized using 0.1% pepsin (Sigma, P7000) in 0.01 M HCl buffer (pH 2.0) and incubated at 37 °C for 18 minutes. Released mRNAs were captured on the Stereo-seq chip and reverse transcribed overnight at 42 °C using SuperScript II reverse transcription mix (Invitrogen, 18064-014), containing 10 U/μL reverse transcriptase, 1 mM dNTPs, 1 M betaine solution, 7.5 mM $MgCl_2$, 5 mM DTT, 2 U/μL RNase inhibitor, 2.5 μM Stereo-seq template switch oligo, and 1× First-Strand buffer.

After *in situ* reverse transcription (RT), tissue sections were treated with a removal buffer (10 mM Tris-HCl, 25 mM EDTA, 100 mM NaCl, 0.5% SDS) at 37 °C for 30 minutes. The remaining RT products were then collected and amplified using KAPA HiFi Hotstart Ready-Mix (Roche, KK2602) and 0.8 μM cDNA-PCR primers. PCR products were used to prepare sequencing libraries, with the following steps: quantification of concentration using the Qubit™ dsDNA Assay Kit (Thermo, Q32854), DNA fragmentation with in-house Tn5 transposase at 55 °C for 10 minutes,

Formatted: Font: Italic

PCR amplification (KAPA HiFi Hotstart Ready-Mix, Roche, KK2602) with 0.8 μM cDNA-PCR primers, and purification using Vazyme (N411-03). The purified PCR products were used to construct DNB libraries and sequenced on an MGI DNBSEQ-T1 sequencer (35 bp for Read1, 100 bp for Read2). The sequencing data were processed to generate a quantified spatial gene expression matrix at the subcellular level.

Spatial transcriptomics data processing

Spatially resolved single-cell RNA-seq data obtained through Stereo-seq were pre-processed for further analysis. The first read (Read1) of the sequencing library contained coordinate identifiers (CIDs), molecular identifiers (MIDs), and poly-T sequences, while the second read (Read2) provided the captured cDNA sequences. Spatial The x-y coordinates of cDNA at (715 nm resolution) were determined based on the CID sequences with a 1-bp mismatch tolerance. cDNA sequences were aligned to the *S. mediterranea* genome (dd_Smes_G4), and only mapped reads were used to identify exon transcripts. The MID sequences served to provide unique molecular identifiers (UMIs) for transcript quantification, with PCR duplicates removed using handleBam (https://github.com/BGIResearch/handleBam). Read pairs with a MID quality score below 10 were excluded. Finally, gene expression matrices incorporating spatial information were generated using quality-controlled clean exonic data.

3D reconstruction, clustering and cell type annotation of regenerating regenerative animals

Regenerating planarians animals were reconstructed using methods outlined in an accompanying manuscript, where we developed a 3D spatial transcriptomics framework (Han et al., submitted) [28]. First, the MIRROR algorithm was applied to align the spatial gene expression heatmap with

the ssDNA staining image. Cell segmentation was then performed utilizingcarried out using CellProfiler and Fiji. Gene expression data were mapped to each individual cell, creating a spatial transcriptome map at single-cell resolution. Next, after performing dimensionality reduction and clustering, cell clusters were annotated based on known lineage markers. The SEAM algorithm was employed to align the sections along the z-axis, thus determining the x-y-z coordinates of each cell. Morphological distortions changes in the animals, causedinduced by experimental procedures, were corrected based on established polarity gene patterns, and the 3D reconstructions were created using a combination of 3DSlicer and MeshLab. Finally, SPC cells from different stages of regeneration were integrated using the FindIntegration and IntegrateData functions in Seurat (v4.0.2). Dimensionality reduction and clustering were then conducted in Seurat following standard procedures. To facilitate a comprehensive understanding of the analytical strategies employed in this study, we provide a schematic overview of the entire computational workflow (Supplementary Fig. S8)XX. This diagram illustrates the sequential processing pipeline, beginning with raw data input (h5ad and imaging files) and preprocessing via the GEM3D toolkit, followed by parallel analytical modules including WACCA for 3D reconstruction, SPC analysis for spatial clustering, and polarity analysis for modeling morphogenetic gradients.

Formatted: Not Highlight

Ddata quality control and validation

Formatted: Font: Bold

To ensure the technical reliability, sensitivity, and reproducibility of our spatial transcriptomic atlas, we systematically evaluated key quality control (QC) metrics across all biological samples, tissue sections, and annotated spatial domains (detailed in Supplementary Table S1). Following single-cell segmentation, we quantified the segmented cell area in (pixels), the number of effective spatial spots (nDNB), total unique molecular identifiers (UMIs), and the number of detected genes

per cell. Analysis of all 35 tissue sections demonstrated high technical consistency across the dataset. The median number of detected genes per cell across sections ranged consistently from approximately 150 to 230, with median UMI counts ranging from 190 to 370.

To rule out potential technical biases that might favor highly transcriptionally active cells over cells with lower RNA content, we statistically evaluated the capture efficiency across all 36 identified spatial domains. Our analysis revealed no meaningful systematic bias toward specific lineages. The distributions of cell area, detected genes, and UMI counts remained broadly consistent and biologically appropriate across the diverse cellular populations. Robust transcript detection was achieved globally, ranging from large differentiated lineages to smaller undifferentiated neoblasts and progenitor states. This uniform data quality confirms that our Stereo-seq approach provides sufficient resolution and sensitivity to capture transcription factors and resolve heterogeneous cell states without significant transcript dropout for low-abundance populations.

Formatted: Indent: First line: 0.5"

Furthermore, to validate the quantitative accuracy of our Stereo-seq dataset at the global tissue level, we assessed its concordance with traditional bulk RNA-seq data. Single-cell spatial expression profiles from Stereo-seq sections were aggregated to generate pseudo-bulk transcriptomes. We then calculated the Pearson correlation coefficients between these pseudo-bulk profiles and corresponding bulk RNA-seq datasets of regenerating planarians. The high correlation observed (Figure S1C) verified the technical reproducibility of our platform and confirmed the absence of significant transcript dropout or amplification bias during *in situ* capturing and library preparation.

Formatted: Font: Italic

For the visualization of spatial gene expression patterns in Fig. 2A, a representative sample was selected for each time point from the biological replicates (n=2). This selection was

determined by calculating the mean gene expression vector (centroid) for each time point and identifying the replicate with the highest Pearson correlation to this centroid. All quantitative analyses and statistical tests were performed using the full integrated dataset across all replicates.

Correlation analysis with single-cell and spatial atlases

Formatted: Font: Bold

To characterize the cellular composition of the 36 identified spatial domains, we performed a Pearson correlation analysis comparing the expression profiles of our clusters against annotated cell types from three independent single-cell RNA-seq atlases [22, 23, 77] and spatial domains from a 10x Genomics Visium dataset [24]. This analysis categorized domains into High Fidelity (1-to-1 mapping), Lineage Restricted (mapping to a single tissue class), or Mixed Domains (containing signatures from multiple cell types, such as the neoblast-parenchyma niche).

Formatted: Line spacing: Double

Blastema region detection in 3D spatial transcriptomics data

The blastema regions were identified based on pigmentation patterns. The boundary between the unpigmented blastema and pigmented trunk was defined using the Threshold function in ImageJ and refined via quadratic polynomial regression. To spatially resolve the interface between pre-existing and newly formed tissue, we defined a border zone extending 20 µm on either side of this regression line. This subdivision created distinct spatial domains at the anterior wound: the Head Anterior Region (HAR), Head Border Outer Region (HBOR), and Head Border Inner Region (HBIR). An analogous ~~identical~~ approach applied to~~at~~ the posterior wound defined the TPR, TBOR, and TBIR domains. For regional analyses, the three anterior (HAR, HBOR, HBIR) and posterior (TPR, TBOR, TBIR) compartments were collectively designated as the head and tail blastema regions, corresponding to their distal, middle, and proximal subdivisions, respectively.

Cells were assigned to these discrete regions based on their spatial coordinates subsequent to following the alignment of the transcriptomic data with the microscopy images utilizing using TrakEM2. This subdivision created distinct spatial domains: the Head Anterior Region (HAR, distal blastema), Head Border Outer Region (HBOR, proximal blastema), and Head Border Inner Region (HBIR, distal trunk). Cells were assigned to these regions based on their spatial coordinates following alignment of the 3D model with the microscopy images using TrakEM2The blastema region was identified and segmented in the spatial coordinates of the reconstructed 3D model based on the pigmentation patterns observed in planarian body images. The boundaries separating the blastema from the rest of the body were defined using differences in pigmentation, which were quantified using the Threshold function in ImageJ. A quadratic polynomial regression was applied to refine these boundaries. The microscopy image and the corresponding 2D projection of the 3D model along the A/P-M/L (x, y) axis were aligned using TrakEM2. Each cell was then labeled with its corresponding region based on its position within the defined boundaries.

Formatted: Line spacing: Double

Identification of temporally differentially expressed genes

Temporal alterations in gene expression were analyzed by comparing adjacent time points for each defined SPC cluster and anatomical region (e.g., blastema and trunk regions) using the DEsingle algorithm. Specifically, expression profiles corresponding to a specific cluster or region were extracted from the integrated dataset. For each pair of adjacent time points (e.g., 0 hpa vs. 12 hpa, 12 hpa vs. 36 hpa, etc.), we employed the DEsingle R package [78] to detect differentially expressed genes (DEGs). Following the primary analysis, we utilized the DEtype function within the DEsingle package to classify the identified DEGs into distinct categories based on variations in gene expression abundance and distribution. To ensure statistical rigor, raw p-values were

adjusted using the Benjamini-Hochberg procedure, and genes with a False Discovery Rate (FDR) < 0.05 were retained as significant DEGs (Fig. 1I, Supplementary Table S2).

Identification of SBGs

To quantitatively examine spatial gene expression patterns, we established a molecular coordinate system by dividing the straightened planarian body into bins along the A/P (100 bins), M/L (40 bins), and D/V (14 bins) axes. SCT-transformed expression values for highly variable genes (HVGs) and known polarity control genes (PCGs) were averaged per bin, normalized by cell density, scaled, and smoothed using a Gaussian filter (sigma=3). Genes expressed in fewer than five consecutive bins were excluded from downstream analysis. We then applied a hierarchical density-based clustering algorithm to the homeostatic dataset to aggregate genes with similar spatial profiles along the body axes. To refine the clustering, parameters were optimized for spatial distinctness, and unassigned genes were assigned to the most probable groups using linear regression. The resulting clusters and their constituent genes are listed in Supplementary Table S3.

Spatial pattern analysis of regenerating animals

To investigate the spatiotemporal dynamics of positional information during regeneration, we applied the aforementioned coordinate binning strategy to the regenerating samples at each time point. Specifically, the expression profiles of the identified SBGs were mapped onto the A/P, M/L, and D/V axes of the regenerating fragments. The regenerated animals were then divided into 100 bins along the A/P axis, in line with the homeostatic reference, and gene expression was categorized for each sample separately. These cluster IDs function as spatial coordinates rather than static gene lists, allowing us to quantify the physical restoration of morphogenetic gradients

over time. By tracking the spatial distribution of these gene clusters over time and comparing them to their homeostatic baselines, we visualized and quantified the restoration of axial polarity and regional patterning across the regeneration process.

Formatted: Line spacing: Double

~~**Spatial pattern clustering of regenerating animals**~~

~~To assess the expression dynamics within each cluster defined in homeostasis (Han et al., submitted), gene expression data from different stages of regeneration were mapped onto the appropriate clusters using linear regression. The regenerated animals were then divided into 100 bins along the A/P axis, in line with the homeostatic reference, and gene expression was categorized for each sample separately.~~

Application of Turing pattern models to SBGs

We hypothesize that the interactions between SBGs and their upstream regulators adhere to ~~follow~~ Turing patterns within an autoregulatory activator-inhibitor framework. Following the removal of~~After removing~~ the influence of spatial gradients, temporal gene expression data across eight regenerative time points were normalized, interpolated, and smoothed. Reaction, degradation, and diffusion parameters were fine-tuned using linear regression. These optimized parameters allowed us to predict gene expression levels at any post-amputation time point for both established and candidate~~known and potential~~ PCGs~~s candidates~~. Pearson's correlation coefficients were then calculated to assess the accuracy of these predictions.

~~principal component analysis (pca~~PCA~~)~~ of SBG~~sbg~~s and PCG~~pcg~~s

To integrate temporal changes in gene expression with spatial variations, PCA was applied to the

binned expression data of selected PCGs along the three axes in homeostatic animals~~-~~ [28]~~(Han et al., submitted)~~. For the A/P axis, the training set comprised ~~included~~ 25 known PCGs. The first principal component (PC1), which accounted for 64.7% of the variance, corresponded to the head-tail gradient, while the second principal component (PC2), which explained 24.7% of the variance, captured fluctuations in the pharyngeal region (convex and concave). Based on their locations in the reduced-dimensional space, genes were manually grouped into five categories: head, h~~H~~ead-pharynx, t~~T~~runk-pharynx, pharynx-tail, and tail domains.

Given the paucity of~~Because there were few~~ previously identified PCGs with clear spatial patterns along the M/L and D/V axes in our Stereo-seq data, we expanded the training sets to include 42 and 67 newly inferred PCGs, respectively. Potential M/L PCGs candidates were selected based on Spearman's rank correlation coefficients greater than 0.7 or less than -0.7 for binned SCT-transformed gene expression data, showing patterns similar to known PCGs. For potential D/V PCGs, a fold change greater than 1.5 between dorsal and ventral regions in binned SCT-transformed values served~~was used~~ as a selection criterion.

PCA was performed using the Scikit-learn package with default parameters. The eigenvectors derived from this analysis were used to map both established ~~known~~ and potential PCGs~~s candidates~~ back into the reduced PCA space, trained on the homeostatic data. The regenerative trajectories of PCGs were visualized, resembling the behavior of an underdamped mass-spring system: the initial amputation stretched the “~~"~~spring”,~~"~~ disrupting the PCG expression profile, while the ~~gene regulatory network~~GRN provided restorative feedback to re-establish homeostasis.~~provided feedback to restore the profile to its homeostatic state.~~

UV unwrapping for the head blastema epidermal ~~epidermis~~ region~~.~~

To convert the 3D planarian body shell~~ of the planarian ~~into a 2D plane, we employed the UV unwrapping technique, a common procedure in the field of computer graphics, using the open-source 3D creation suite Blender (https://www.blender.org/)~~, an open source 3D creation suite~~. UV unwrapping is a process in which the surface of a 3D model is mathematically "unwrapped" and mapped onto a 2D plane, enabling precise application of textures and structures onto a flat surface, which is essential for accurate visualization and analysis. The process of UV unwrapping for the head blastema epidermis involved segmenting the planarian body, marking seams for accurate texture alignment, unwrapping the mesh, and mapping the epidermal cells onto a 2D plane. This approach enabled a high-precision representation of the head blastema's epidermal region, which will be useful for further texture analysis and studies related to planarian regeneration.

Briefly, the planarian body mesh was first segmented into two sections along a defined plane located near the boundary of the head blastema. This segmentation was performed using the Bisect Tool within Blender, which allowed for a clean division of the mesh without distorting the geometry. The separation enabled us to isolate the head blastema area, which we intended to unwrap for detailed analysis.

Next, to prepare for the unwrapping process, seams were strategically marked to guide the unfolding of the 3D mesh. The edges connecting the blastema cutting plane to the anterior pole of the planarian were designated as a seam, specifically placed along the D/V boundary. This step ensure~~s~~d that the unwrapping process adheres to natural anatomical divisions, preventing distortion of the texture in the subsequent 2D plane. ~~The s~~Seam marking was carried out using the Blender's UV editor tool, where the mesh's geometry was manipulated to set boundaries for the unwrapping operation.

Once the seams were defined, the head blastema mesh was subjected to the UV unwrapping

operation. The mesh was unfolded into a 2D plane, with particular attention paid to the correct alignment of the marked seam. The seam was clipped and adjusted to create a smooth, curved incision, ~~which~~ representings the head blastema epidermal cells ~~of the head blastema accurately~~ in a flat space. This incision helped ensure that the texture mapping would preserve the anatomical integrity of the original 3D model.

Finally, we focused on the outer epidermal cells of the head blastema, which were part of the original 3D point cloud data. These epidermal cells were mapped onto the unwrapped 2D plane using a process that minimized the distance between the original 3D coordinates of each epidermal cell and the vertices of the subdivided mesh surface. This step ensured a precise mapping of cellular structures to the 2D plane, allowing for high fidelity in representing the epidermal region's texture and topology. The minimization of these distances ~~between the original 3D points and the 2D unwrapped model helped~~ preserved spatial relationships and accurately represented the cellular organization in a flattened format.

Monocle3 analysis

To investigate the state transitions of the ARZ (Clu.31 cluster) during regeneration, we analyzed the trajectory dynamics using raw transcriptome counts from 36 hours post-amputation (hpa) to 14 days post-amputation (dpa). The raw counts were first normalized using SCTransform in Seurat (v4.0.2). Dimensionality reduction and clustering were then performed in Monocle3 (v1.3.1) [44]. The trajectory graph was constructed by fitting the principal graph with the learn_graph function, and pseudotime was calculated with the neoblast cell type set as the root. Marker genes for the ARZ (Clu.31 cluster) were identified using Seurat, and their expression was projected onto the trajectory branches. Cells were clustered in a 15-NN graph, which allowed us to divide the

trajectory into distinct branches enriched for epidermal, muscle, and neuronal signatures.

RNA velocity analysis

RNA velocity analysis, based on Waddington's epigenetic landscape and differential geometry, was used to make continuous, time-resolved predictions of cell state transitions. Cellular genes were aligned to the reference genome to identify exon and intron sequences. The relative abundance of spliced (mature) and unspliced (nascent) mRNAs was calculated to estimate splicing and degradation rates using Velocyto [46]. ~~E~~ Each DNB was assigned to its corresponding cell based on its x and y coordinates. Spliced and unspliced count matrices for different ~~cell types~~domains were processed using the recipe_monocle function in Dynamo [79] to identify highly expressed genes. Following dimensionality reduction, the continuous velocity vector field was reconstructed in UMAP space to predict future cell fates.

Monocle2 analysis

Monocle2 (v2.18.0) was used to analyze the ventral epidermal trajectory across different regeneration time points, following the tutorial (http://cole-trapnell-lab.github.io/monocle-release) [80].We focused on extracting SPC clusters during the putative ventral epidermal transition from the blastema region. Differentially expressed genes for each cell type were identified and used to order the cells. Dimensionality reduction was carried out using the DDRTree method, and the plot_cell_trajectory function was used for visualization. Marker genes identified by Seurat's FindAllMarkers function were projected along the estimated pseudotime to assess their potential role in the transition.

Visual representation of the ~~website:~~ PRISTA4D interactive spatiotemporal transcriptomic atlas database~~Planarian Regenerative Interactive Spatiotemporal Transcriptomic Atlas in Four Dimensions (PRISTA4D)~~

To enhance the accessibility and utility of our PRISTA4D (Planarian Regenerative Interactive Spatiotemporal Transcriptomic Atlas in Four Dimensions)~~4D planarian cell atlas~~ for researchers in the field of regeneration, we developed an open-source, interactive database (available at https://www.bgiocean.com/planarian). This platform enables users to explore the spatial distribution and dynamic changes of various genes and cells at different stages of regeneration.

Formatted: Indent: First line: 0 ch, Line spacing: Double

The PRISTA4D database provides several functions, including browsing capabilities and access to experimental procedures, data analysis pipelines, and the ability to download the original dataset. It serves as a~~n invaluable~~ resource for studying cell differentiation and spatiotemporal cell interactions within the regeneration research community.

Formatted: Line spacing: Double

The website includes a homepage and five key functional modules:

3D Model: Visualizes different ~~cell types~~domains in three dimensions, allowing users to view cellular organization across regeneration stages.

Spatial Clustering Module: Illustrates the distribution of genes and ~~cell types~~domains, offering insights into spatial gene expression patterns.

Stereo-seq Module: Provides detailed experimental protocols used to generate the transcriptomic data, ensuring transparency and reproducibility.

Sampling Design Module: Offers information on sample design and the data analysis pipelines used, enabling users to understand how the data was processed and analyzed.

Download Module: Grants access to the complete original dataset, allowing users to download the raw data for further analysis and research.

Cell sorting and library construction for s~~ingle-cell~~ cRNA-seq ~~sequencing~~

To prepare the cell suspension for scRNA-seq, CMFB buffer (CMF + 1% FBS) was placed on a cold plate (4 °C), and the animals were incubated in this solution before their tissues were manually chopped to release cells as described previously [8]. After dissociation, the cells were pelleted by centrifugation at 290 × g for 5 minutes at 4 °C. The resulting suspension was passed ~~filtered~~ through a 40 μm filter and stained with DAPI (1:1000; Beyotime) and DRAQ5 (1:1000; BioLegend). The cells were washed and resuspended in CMFB buffer. Flow cytometry and sorting were performed using a Sony MA900 cell sorter, with the temperature maintained ~~kept~~ at 4 °C to preserve ~~maintain~~ cell integrity. Approximately ~~Around~~ 20,000 viable cells (DAPI-; DRAQ5+) were loaded onto the SeekGene platform using the Single Cell 3' Transcriptome kit to generate scRNA-seq libraries. Library preparation followed the manufacturer's guidelines to ensure optimal coverage, and sequencing was conducted on an Illumina NovaSeq platform with paired-end 150 base pair (150 PE) reads for detailed transcriptomic profiling.

Analysis of scRNA-seq~~single-cell RNA sequence~~ data after RNAi knockdown

Raw scRNA-seq data were processed and aligned with the *S. mediterranea* reference transcriptome (smed_20140614). Cells containing~~that had~~ fewer than 200 detected features or genes expressed in fewer than three cells were excluded. Cells exhibiting unusually high mitochondrial or ribosomal RNA content (where mitochondrial percentage was more than twice the median) were also filtered out based on the annotated reads (~~data S~~Supplementary Table S6). After quality control, the remaining Unique Molecular Identifiers (UMIs) were quantified and analyzed using the Seurat package in R, which enabled normalization, scaling, and dimensionality reduction of

the data. Principal component analysis (PCA) was performed, and the top 30 principal components were used for 2D UMAP generation and clustering within Seurat. Cell lineages were annotated based on the expression of known marker genes, while SPC clusters were identified using markers derived from 3D spatial transcriptomics. Batch effects due to technical variations across samples were corrected using Seurat's integration functions, ensuring that the observed differences were biologically relevant. For analyzing cellular differentiation trajectories and lineage relationships, Monocle 2 was applied. Additionally, MiloR was used to examine and compare the abundance of cells in specific neighborhoods or microenvironments between the control and RNAi-treated groups [81]. Briefly, PCA was used for dimensionality reduction, and a KNN graph was constructed with the buildGraph function (k=30, d=30) based on the top 30 PCA dimensions. Neighborhoods were defined using the makeNhoods function (prop=0.1, k=30, d=30), and differential abundance testing was conducted with default parameters using the distinct function. Differentially abundant cell populations between control and knockdown groups were identified, and differentially expressed genes (DEGs) were determined using DESingle (v1.9.2) with standard settings [78]. Genes with an adjusted p-value of less than 0.05 were considered significantly different. This comparative analysis revealed how RNAi interventions altered affect the cellular composition within the tissue.

Availability of source code and requirements

Project name: 4D-BioReconX

Project home page: https://github.com/BGI-Qingdao/4D-BioReconX

Operating system(s): Linux or MacOS

Programming language: Jupyter Notebook, Python, R, Shell

Other requirements: anndata>=0.7.5, matplotlib>=3.6.2, numpy>=1.22.4, opencv-

Formatted: Font:

Field Code Changed

Formatted: Hyperlink, Font: (Default) Times New Roman, 12 pt

python>=4.6.0.66, pandas>=1.4.3, scikit-image>=0.19.2, scipy>=1.9.0, seaborn>=0.11.2

License: MIT License

RRID: SCR_027919

bio.tools ID: 4d-bioreconx

WorkflowHub: 10.48546/workflowhub.workflow.2045.1

Data ~~and materials~~ ~~A~~availability~~:~~

All data generated in this study were deposited in the~~at~~ CNGB Nucleotide Sequence Archive (accession code: STT0000028). The accession number for *Rod1* (SMED30003831) is OR211556. Processed data and 3D models can be interactively explored via ~~from~~ our PRISTA4D database (https://db.cngb.org/stomics/prista4d). All original code ~~s~~ supporting the current study is~~are~~ hosted on GitHub [82] and WorkflowHub [83] ~~(https://github.com/BGI-Qingdao/4D-BioReconX)~~. Any additional information required to reanalyze the data reported in this paper is available from the lead contact upon request.

List of abbreviations

A/P: Anterior/Posterior; ARZ: Anterior Regenerative Zone; D/V: Dorsal/Ventral; DEG: Differentially Expressed Gene; dpa: days post-amputation; FISH: Fluorescent In Situ Hybridization; GRN: Gene Regulatory Network; hpa: hours post-amputation; HVG: Highly Variable Gene; M/L: Medial/Lateral; PCA: Principal Component Analysis; PCG: Positional Control Gene; RNAi: RNA interference; SBG: Spatially Biased Gene; scRNA-seq: single-cell RNA sequencing; SPC: Spatial Proximity-based Clustering; ST: Spatial Transcriptomics; TF: Transcription Factor; UMI: Unique Molecular Identifier; WISH: Whole-mount In Situ Hybridization.

Formatted: Left, Space Before: 0 pt, After: 0 pt, Line spacing: Double, Tab stops: 1.47", Left

Formatted: Font: Not Bold

Formatted: Font: Italic

Formatted: Line spacing: Double

Competing interests

Formatted: Heading 2, Space Before: 0 pt, After: 0 pt, Line spacing: Double

~~Authors~~ The authors declare that they have no competing interests.

Formatted: Line spacing: Double

Funding

Formatted: Heading 2, Space Before: 0 pt, After: 0 pt, Line spacing: Double

This research was supported by the National Key R&D Program of China (2022YFC3400400), the National Key R&D Program of China (2020YFA0112502 and 2021YFA1100202 to A.Z.), the National Natural Science Foundation of China (32070828 to A.Z.), Shenzhen Science and Technology Program (JCYJ20250604191305008 to M.X. and RCJC20221008092804002 to Y.G.), the Strategic Priority Research Program of the Chinese Academy of Sciences (XDA16021300), the CAS Pioneer Hundred Talents Program (A.Z.), Shanghai Pujiang Program (20PJ1414600 to A.Z.), the Shanghai Science and Technology Committee (STCSM) (22ZR1468400 to A.Z.), Guangdong Genomics Data Center (2021B1212100001) and the Feng Foundation of Biomedical Research (A.Z.).

Formatted: Line spacing: Double

Authors’ contributions

Formatted: Heading 2, Space Before: 0 pt, After: 0 pt, Line spacing: Double

A.Z., X.X., G.F., H.L., K.H. and M.X. conceived and directed the study. A.Z., X.X., M.X. and H.L. supervised the work. Y.C. and YR.L. performed animal experiments and RNAi. XW.L., W.G., JQ.W., W.W. and H.P. performed the Stereo-seq experiments. K.H., M.X., L.G., Y.L., Y.W. and Z.H. analyzed the data. L.G., Y.L. and T.Y. performed database construction. Q.L., L.Z. and X.M. assisted in the data analysis. R.Z., L.L., X.W., H.Z., X.S., S.L., W.Z., S.T.C, J.F., X.L., Y.G., J.W. G.~~-~~P., and H.Y. performed investigations. A.Z., G.F., H.L., M.X. and K.H. wrote the manuscript with input from all ~~the~~ authors.

Formatted: Line spacing: Double

Acknowledgments

Formatted: Heading 2, Space Before: 0 pt, After: 0 pt, Line spacing: Double

We thank L. Bolund, D. Little and all Zeng lab members for ~~the~~ critical reading of the manuscript.

Formatted: Line spacing: Double

References

1. Forsthoefel DJ and Newmark PA. Emerging patterns in planarian regeneration. Curr Opin Genet Dev. 2009;19 4:412-20. doi:10.1016/j.gde.2009.05.003.
2. Poss KD and Tanaka EM. Hallmarks of regeneration. Cell Stem Cell. 2024;31 9:1244-61. doi:10.1016/j.stem.2024.07.007.
3. Newmark PA and Sanchez Alvarado A. Not your father's planarian: a classic model enters the era of functional genomics. Nat Rev Genet. 2002;3 3:210-9. doi:10.1038/nrg759.
4. Poss KD. Advances in understanding tissue regenerative capacity and mechanisms in animals. Nat Rev Genet. 2010;11 10:710-22. doi:10.1038/nrg2879.
5. Wolpert L. Positional Information and Pattern Formation. Curr Top Dev Biol. 2016;117:597-608. doi:10.1016/bs.ctdb.2015.11.008.
6. Wang M, Hu Q, Lv T, Wang Y, Lan Q, Xiang R, et al. High-resolution 3D spatiotemporal transcriptomic maps of developing Drosophila embryos and larvae. Dev Cell. 2022;57 10:1271-83 e4. doi:10.1016/j.devcel.2022.04.006.
7. Reddien PW and Sanchez Alvarado A. Fundamentals of planarian regeneration. Annu Rev Cell Dev Biol. 2004;20:725-57. doi:10.1146/annurev.cellbio.20.010403.095114.
8. Zeng A, Li H, Guo L, Gao X, McKinney S, Wang Y, et al. Prospectively Isolated Tetraspanin(+) Neoblasts Are Adult Pluripotent Stem Cells Underlying Planaria Regeneration. Cell. 2018;173 7:1593-608 e20. doi:10.1016/j.cell.2018.05.006.
9. Wagner DE, Wang IE and Reddien PW. Clonogenic neoblasts are pluripotent adult stem cells that underlie planarian regeneration. Science. 2011;332 6031:811-6. doi:10.1126/science.1203983.
10. Reddien PW. The Cellular and Molecular Basis for Planarian Regeneration. Cell. 2018;175 2:327-45. doi:10.1016/j.cell.2018.09.021.
11. Adell T, Cebria F and Salo E. Gradients in planarian regeneration and homeostasis. Cold Spring Harb Perspect Biol. 2010;2 1:a000505. doi:10.1101/cshperspect.a000505.
12. Rink JC, Gurley KA, Elliott SA and Sanchez Alvarado A. Planarian Hh signaling regulates regeneration polarity and links Hh pathway evolution to cilia. Science. 2009;326 5958:1406-10. doi:10.1126/science.1178712.
13. Petersen CP and Reddien PW. Smed-betacatenin-1 is required for anteroposterior blastema polarity in planarian regeneration. Science. 2008;319 5861:327-30. doi:10.1126/science.1149943.
14. Cebria F, Kobayashi C, Umesono Y, Nakazawa M, Mineta K, Ikeo K, et al. FGFR-related gene nou-darake restricts brain tissues to the head region of planarians. Nature. 2002;419 6907:620-4. doi:10.1038/nature01042.
15. Umesono Y, Tasaki J, Nishimura Y, Hrouda M, Kawaguchi E, Yazawa S, et al. The molecular logic for planarian regeneration along the anterior-posterior axis. Nature. 2013;500 7460:73-6. doi:10.1038/nature12359.
16. Witchley JN, Mayer M, Wagner DE, Owen JH and Reddien PW. Muscle cells provide instructions for planarian regeneration. Cell Rep. 2013;4 4:633-41. doi:10.1016/j.celrep.2013.07.022.
17. Benham-Pyle BW, Brewster CE, Kent AM, Mann FG, Jr., Chen S, Scott AR, et al. Identification of rare, transient post-mitotic cell states that are induced by injury and required for whole-body regeneration in Schmidtea mediterranea. Nat Cell Biol. 2021;23 9:939-52. doi:10.1038/s41556-021-00734-6.
18. Garcia-Castro H and Solana J. Single-cell transcriptomics in planaria: new tools allow new insights into cellular and evolutionary features. Biochem Soc Trans. 2022;50 5:1237-46. doi:10.1042/BST20210825.
19. Wurtzel O, Cote LE, Poirier A, Satija R, Regev A and Reddien PW. A Generic and Cell-Type-Specific Wound Response Precedes Regeneration in Planarians. Dev Cell. 2015;35 5:632-45. doi:10.1016/j.devcel.2015.11.004.
20. Molinaro AM and Pearson BJ. In silico lineage tracing through single cell transcriptomics

Formatted: Heading 2, Space Before: 0 pt, After: 0 pt, Line spacing: Double

Formatted: Font: 11 pt

identifies a neural stem cell population in planarians. Genome Biol. 2016;17:87. doi:10.1186/s13059-016-0937-9.
21. van Wolfswinkel JC, Wagner DE and Reddien PW. Single-cell analysis reveals functionally distinct classes within the planarian stem cell compartment. Cell Stem Cell. 2014;15 3:326-39. doi:10.1016/j.stem.2014.06.007.
22. Fincher CT, Wurtzel O, de Hoog T, Kravarik KM and Reddien PW. Cell type transcriptome atlas for the planarian Schmidtea mediterranea. Science. 2018;360 6391 doi:10.1126/science.aaq1736.
23. Plass M, Solana J, Wolf FA, Ayoub S, Misios A, Glažar P, et al. Cell type atlas and lineage tree of a whole complex animal by single-cell transcriptomics. Science. 2018;360 6391 doi:10.1126/science.aaq1723.
24. Cui G, Dong K, Zhou JY, Li S, Wu Y, Han Q, et al. Spatiotemporal transcriptomic atlas reveals the dynamic characteristics and key regulators of planarian regeneration. Nat Commun. 2023;14 1:3205. doi:10.1038/s41467-023-39016-0.
25. Park C, Owusu-Boaitey KE, Valdes GM and Reddien PW. Fate specification is spatially intermingled across planarian stem cells. Nat Commun. 2023;14 1:7422. doi:10.1038/s41467-023-43267-2.
26. Kato K, Orii H, Watanabe K and Agata K. Dorsal and ventral positional cues required for the onset of planarian regeneration may reside in differentiated cells. Dev Biol. 2001;233 1:109-21. doi:10.1006/dbio.2001.0226.
27. Chen A, Liao S, Cheng M, Ma K, Wu L, Lai Y, et al. Spatiotemporal transcriptomic atlas of mouse organogenesis using DNA nanoball-patterned arrays. Cell. 2022;185 10:1777-92 e21. doi:10.1016/j.cell.2022.04.003.
28. Sun M, Wang Y, Han K, Guo L, Chen Y, Li Y, et al. Cell Type Architecture and Positional Gene Gradients in an Adult Animal at Subcellular Resolution. bioRxiv. 2026:2026.02.19.705280. doi:10.64898/2026.02.19.705280.
29. Roberts-Galbraith RH, Brubacher JL and Newmark PA. A functional genomics screen in planarians reveals regulators of whole-brain regeneration. Elife. 2016;5 doi:10.7554/eLife.17002.
30. Levin M, Pietak AM and Bischof J. Planarian regeneration as a model of anatomical homeostasis: Recent progress in biophysical and computational approaches. Semin Cell Dev Biol. 2019;87:125-44. doi:10.1016/j.semcdb.2018.04.003.
31. King RS and Newmark PA. In situ hybridization protocol for enhanced detection of gene expression in the planarian Schmidtea mediterranea. BMC Dev Biol. 2013;13:8. doi:10.1186/1471-213X-13-8.
32. Stuckemann T, Cleland JP, Werner S, Thi-Kim Vu H, Bayersdorf R, Liu SY, et al. Antagonistic Self-Organizing Patterning Systems Control Maintenance and Regeneration of the Anteroposterior Axis in Planarians. Dev Cell. 2017;40 3:248-63 e4. doi:10.1016/j.devcel.2016.12.024.
33. Lander R and Petersen CP. Wnt, Ptk7, and FGFRL expression gradients control trunk positional identity in planarian regeneration. Elife. 2016;5 doi:10.7554/eLife.12850.
34. Scimone ML, Cote LE, Rogers T and Reddien PW. Two FGFRL-Wnt circuits organize the planarian anteroposterior axis. Elife. 2016;5 doi:10.7554/eLife.12845.
35. Kobayashi C, Saito Y, Ogawa K and Agata K. Wnt signaling is required for antero-posterior patterning of the planarian brain. Dev Biol. 2007;306 2:714-24. doi:10.1016/j.ydbio.2007.04.010.
36. Gurley KA, Elliott SA, Simakov O, Schmidt HA, Holstein TW and Sanchez Alvarado A. Expression of secreted Wnt pathway components reveals unexpected complexity of the planarian amputation response. Dev Biol. 2010;347 1:24-39. doi:10.1016/j.ydbio.2010.08.007.
37. Inman DJ. Engineering Vibration (3rd Edition). Pearson Education, Inc; 2007.
38. Macarthur BD, Ma'ayan A and Lemischka IR. Systems biology of stem cell fate and cellular reprogramming. Nat Rev Mol Cell Biol. 2009;10 10:672-81. doi:10.1038/nrm2766.
39. Gierer A and Meinhardt H. A theory of biological pattern formation. Kybernetik. 1972;12 1:30-9. doi:10.1007/BF00289234.

40. Werner S, Stuckemann T, Beiran Amigo M, Rink JC, Julicher F and Friedrich BM. Scaling and regeneration of self-organized patterns. Phys Rev Lett. 2015;114 13:138101. doi:10.1103/PhysRevLett.114.138101.

41. Cowles MW, Brown DD, Nisperos SV, Stanley BN, Pearson BJ and Zayas RM. Genome-wide analysis of the bHLH gene family in planarians identifies factors required for adult neurogenesis and neuronal regeneration. Development. 2013;140 23:4691-702. doi:10.1242/dev.098616.

42. Sureda-Gomez M and Adell T. Planarian organizers. Semin Cell Dev Biol. 2019;87:95-104. doi:10.1016/j.semcdb.2018.05.021.

43. Scimone ML, Cloutier JK, Maybrun CL and Reddien PW. The planarian wound epidermis gene equinox is required for blastema formation in regeneration. Nat Commun. 2022;13 1:2726. doi:10.1038/s41467-022-30412-6.

44. Cao J, Spielmann M, Qiu X, Huang X, Ibrahim DM, Hill AJ, et al. The single-cell transcriptional landscape of mammalian organogenesis. Nature. 2019;566 7745:496-502. doi:10.1038/s41586-019-0969-x.

45. Kent AM, Guerrero-Hernández C, Brewster C, McKinney S, Morrison JA, McKinney MC, et al. Metabolites produced by agat+ cells support regeneration in the planarian Schmidtea mediterranea. Dev Biol. 2026;529:106-20. doi:10.1016/j.ydbio.2025.10.001.

46. La Manno G, Soldatov R, Zeisel A, Braun E, Hochgerner H, Petukhov V, et al. RNA velocity of single cells. Nature. 2018;560 7719:494-8. doi:10.1038/s41586-018-0414-6.

47. Brower CS, Sato S, Tomomori-Sato C, Kamura T, Pause A, Stearman R, et al. Mammalian mediator subunit mMED8 is an Elongin BC-interacting protein that can assemble with Cul2 and Rbx1 to reconstitute a ubiquitin ligase. Proc Natl Acad Sci U S A. 2002;99 16:10353-8. doi:10.1073/pnas.162424199.

48. Gurley KA, Rink JC and Sanchez Alvarado A. Beta-catenin defines head versus tail identity during planarian regeneration and homeostasis. Science. 2008;319 5861:323-7. doi:10.1126/science.1150029.

49. Brown DDR, Molinaro AM and Pearson BJ. The planarian TCF/LEF factor Smed-tcf1 is required for the regeneration of dorsal-lateral neuronal subtypes. Dev Biol. 2018;433 2:374-83. doi:10.1016/j.ydbio.2017.08.024.

50. Pearson BJ and Sanchez Alvarado A. A planarian p53 homolog regulates proliferation and self-renewal in adult stem cell lineages. Development. 2010;137 2:213-21. doi:10.1242/dev.044297.

51. Yin JW and Wang G. The Mediator complex: a master coordinator of transcription and cell lineage development. Development. 2014;141 5:977-87. doi:10.1242/dev.098392.

52. Benham-Pyle BW, Mann FG, Brewster CE, Dewars ER, Vuu DM, Nowotarski SH, et al. Planarians employ diverse and dynamic stem cell microenvironments to support whole-body regeneration. bioRxiv. 2023:2022.03.20.485025. doi:10.1101/2022.03.20.485025.

53. Herath S and Lobo D. Cross-inhibition of Turing patterns explains the self-organized regulatory mechanism of planarian fission. J Theor Biol. 2020;485:110042. doi:10.1016/j.jtbi.2019.110042.

54. Pietak A, Bischof J, LaPalme J, Morokuma J and Levin M. Neural control of body-plan axis in regenerating planaria. PLoS Comput Biol. 2019;15 4:e1006904. doi:10.1371/journal.pcbi.1006904.

55. Stückemann T, Cleland JP, Werner S, Thi-Kim Vu H, Bayersdorf R, Liu SY, et al. Antagonistic Self-Organizing Patterning Systems Control Maintenance and Regeneration of the Anteroposterior Axis in Planarians. Dev Cell. 2017;40 3:248-63.e4. doi:10.1016/j.devcel.2016.12.024.

56. Wurtzel O, Oderberg IM and Reddien PW. Planarian Epidermal Stem Cells Respond to Positional Cues to Promote Cell-Type Diversity. Dev Cell. 2017;40 5:491-504 e5. doi:10.1016/j.devcel.2017.02.008.

57. Currie KW, Molinaro AM and Pearson BJ. Neuronal sources of hedgehog modulate neurogenesis in the adult planarian brain. Elife. 2016;5 doi:10.7554/eLife.19735.

58. Chan A, Ma S, Pearson BJ and Chan D. Collagen IV differentially regulates planarian stem cell

potency and lineage progression. Proc Natl Acad Sci U S A. 2021;118 16 doi:10.1073/pnas.2021251118.

59. Hill EM and Petersen CP. Positional information specifies the site of organ regeneration and not tissue maintenance in planarians. Elife. 2018;7 doi:10.7554/eLife.33680.

60. Wu W, Liu S, Wu H, Chen M, Gao L, Zhao B, et al. DjPtpn11 is an essential modulator of planarian (Dugesia japonica) regeneration. Int J Biol Macromol. 2022;209 Pt A:1054-64. doi:10.1016/j.ijbiomac.2022.04.095.

61. Oderberg IM, Li DJ, Scimone ML, Gavino MA and Reddien PW. Landmarks in Existing Tissue at Wounds Are Utilized to Generate Pattern in Regenerating Tissue. Curr Biol. 2017;27 5:733-42. doi:10.1016/j.cub.2017.01.024.

62. Christensen RN and Tassava RA. Apical epithelial cap morphology and fibronectin gene expression in regenerating axolotl limbs. Dev Dyn. 2000;217 2:216-24. doi:10.1002/(SICI)1097-0177(200002)217:2<216::AID-DVDY8>3.0.CO;2-8.

63. McCusker C, Bryant SV and Gardiner DM. The axolotl limb blastema: cellular and molecular mechanisms driving blastema formation and limb regeneration in tetrapods. Regeneration (Oxf). 2015;2 2:54-71. doi:10.1002/reg2.32.

64. Zhong J, Aires R, Tsissios G, Skoufa E, Brandt K, Sandoval-Guzman T, et al. Multi-species atlas resolves an axolotl limb development and regeneration paradox. Nat Commun. 2023;14 1:6346. doi:10.1038/s41467-023-41944-w.

65. Burrows JT, Pearson BJ and Scott IC. An in vivo requirement for the mediator subunit med14 in the maintenance of stem cell populations. Stem Cell Reports. 2015;4 4:670-84. doi:10.1016/j.stemcr.2015.02.006.

66. Pascual-Carreras E, Marin-Barba M, Castillo-Lara S, Coronel-Cordoba P, Magri MS, Wheeler GN, et al. Wnt/beta-catenin signalling is required for pole-specific chromatin remodeling during planarian regeneration. Nat Commun. 2023;14 1:298. doi:10.1038/s41467-023-35937-y.

67. Blassberg RA, Felix DA, Tejada-Romero B and Aboobaker AA. PBX/extradenticle is required to re-establish axial structures and polarity during planarian regeneration. Development. 2013;140 4:730-9. doi:10.1242/dev.082982.

68. Chen CC, Wang IE and Reddien PW. pbx is required for pole and eye regeneration in planarians. Development. 2013;140 4:719-29. doi:10.1242/dev.083741.

69. Vogg MC, Owlarn S, Perez Rico YA, Xie J, Suzuki Y, Gentile L, et al. Stem cell-dependent formation of a functional anterior regeneration pole in planarians requires Zic and Forkhead transcription factors. Dev Biol. 2014;390 2:136-48. doi:10.1016/j.ydbio.2014.03.016.

70. Scimone ML, Lapan SW and Reddien PW. A forkhead transcription factor is wound-induced at the planarian midline and required for anterior pole regeneration. PLoS Genet. 2014;10 1:e1003999. doi:10.1371/journal.pgen.1003999.

71. Vasquez-Doorman C and Petersen CP. zic-1 Expression in Planarian neoblasts after injury controls anterior pole regeneration. PLoS Genet. 2014;10 7:e1004452. doi:10.1371/journal.pgen.1004452.

72. Newmark PA and Sanchez Alvarado A. Bromodeoxyuridine specifically labels the regenerative stem cells of planarians. Dev Biol. 2000;220 2:142-53. doi:10.1006/dbio.2000.9645.

73. Adler CE, Seidel CW, McKinney SA and Sanchez Alvarado A. Selective amputation of the pharynx identifies a FoxA-dependent regeneration program in planaria. Elife. 2014;3:e02238. doi:10.7554/eLife.02238.

74. Newmark PA, Reddien PW, Cebria F and Sanchez Alvarado A. Ingestion of bacterially expressed double-stranded RNA inhibits gene expression in planarians. Proc Natl Acad Sci U S A. 2003;100 Suppl 1 Suppl 1:11861-5. doi:10.1073/pnas.1834205100.

75. Pearson BJ, Eisenhoffer GT, Gurley KA, Rink JC, Miller DE and Sanchez Alvarado A. Formaldehyde-based whole-mount in situ hybridization method for planarians. Dev Dyn. 2009;238 2:443-50. doi:10.1002/dvdy.21849.

76. Forsthoefel DJ, Cejda NI, Khan UW and Newmark PA. Cell-type diversity and regionalized gene

expression in the planarian intestine. Elife. 2020;9 doi:10.7554/eLife.52613.

77. Emili E, Pérez-Posada A, Vanni V, Salamanca-Díaz D, Ródriguez-Fernández D, Christodoulou MD, et al. Allometry of cell types in planarians by single-cell transcriptomics. Sci Adv. 2025;11 19:eadm7042. doi:10.1126/sciadv.adm7042.

78. Miao Z, Deng K, Wang X and Zhang X. DEsingle for detecting three types of differential expression in single-cell RNA-seq data. Bioinformatics. 2018;34 18:3223-4. doi:10.1093/bioinformatics/bty332.

79. Qiu X, Zhang Y, Martin-Rufino JD, Weng C, Hosseinzadeh S, Yang D, et al. Mapping transcriptomic vector fields of single cells. Cell. 2022;185 4:690-711 e45. doi:10.1016/j.cell.2021.12.045.

80. Qiu X, Hill A, Packer J, Lin D, Ma YA and Trapnell C. Single-cell mRNA quantification and differential analysis with Census. Nat Methods. 2017;14 3:309-15. doi:10.1038/nmeth.4150.

81. Dann E, Henderson NC, Teichmann SA, Morgan MD and Marioni JC. Differential abundance testing on single-cell data using k-nearest neighbor graphs. Nat Biotechnol. 2022;40 2:245-53. doi:10.1038/s41587-021-01033-z.

82. BGI-Qingdao: 4D-BioReconX. https://github.com/BGI-Qingdao/4D-BioReconX (2025). Accessed 2026-02-24.

83. Lidong G. 4D-BioReconX: a bioinformatic framework for reconstructing 4D spatial transcriptomics atlas and spatiotemporal analyses+. WorkflowHub. 2026; doi:10.48546/workflowhub.workflow.2045.1.

Formatted: Line spacing: Double

Figures:

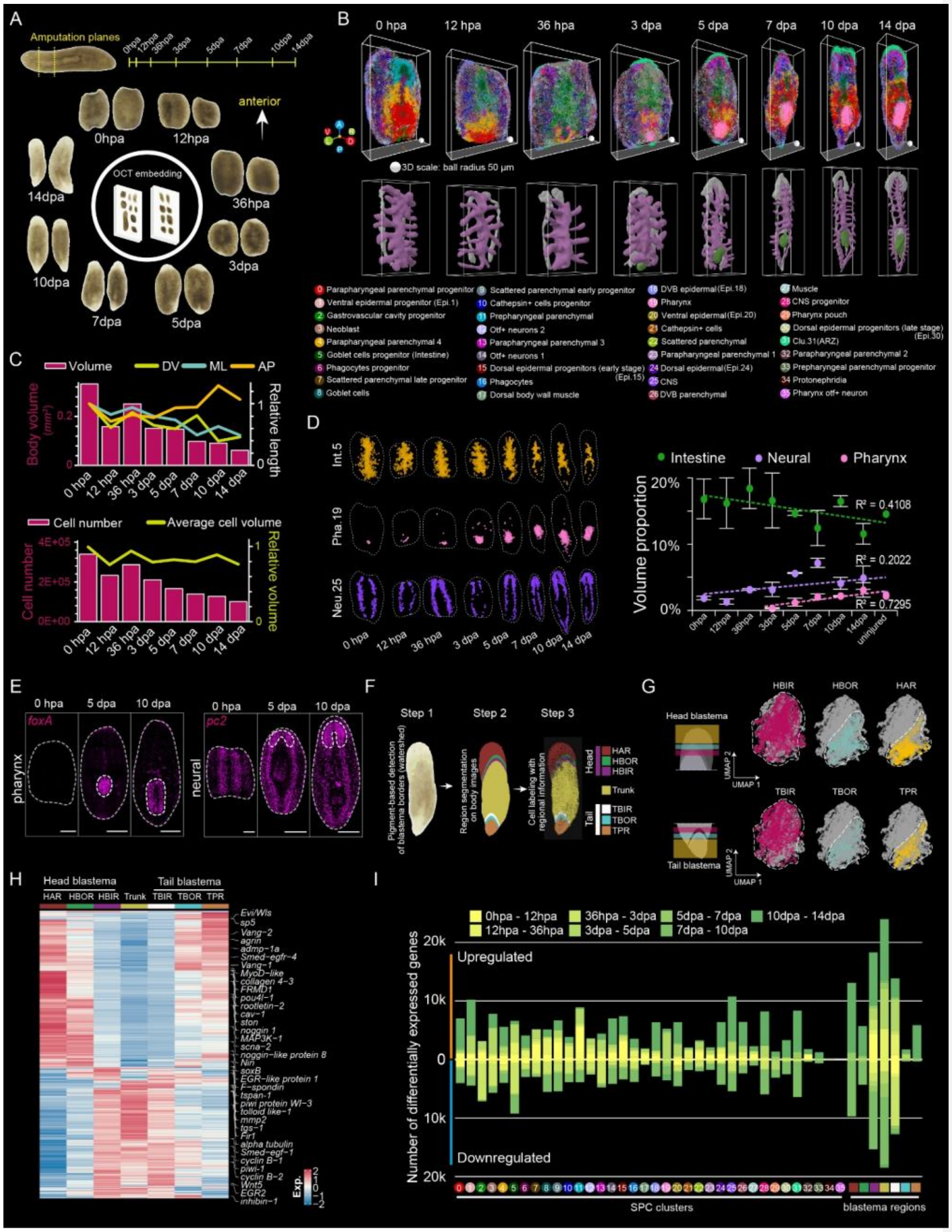

A
Amputation planes
0hpa
12hpa
36hpa
3dpa
5dpa
7dpa
10dpa
14dpa
anterior
OCT embedding
B
0 hpa
12 hpa
36 hpa
3 dpa
5 dpa
7 dpa
10 dpa
14 dpa
3D scale: ball radius 50 μm
0 Parapharyngeal parenchymal progenitor
1 Ventral epidermal progenitor (Epi.1)
2 Gastrovascular cavity progenitor
3 Neoblast
4 Parapharyngeal parenchymal 4
5 Goblet cells progenitor (Intestine)
6 Phagocytes progenitor
7 Scattered parenchymal late progenitor
8 Goblet cells
9 Scattered parenchymal early progenitor
10 Cathepsin+ cells progenitor
11 Prepharyngeal parenchymal
12 Otf+ neurons 2
13 Parapharyngeal parenchymal 3
14 Otf+ neurons 1
15 Dorsal epidermal progenitors (early stage) (Epi.15)
16 Phagocytes
17 Dorsal body wall muscle
18 DVB epidermal (Epi.18)
19 Pharynx
20 Ventral epidermal (Epi.20)
21 Cathepsin+ cells
22 Scattered parenchymal
23 Parapharyngeal parenchymal 1
24 Dorsal epidermal (Epi.24)
25 CNS
26 DVB parenchymal
27 Muscle
28 CNS progenitor
29 Pharynx pouch
30 Dorsal epidermal progenitors (late stage) (Epi.30)
31 Clu.31(ARZ)
32 Parapharyngeal parenchymal 2
33 Prepharyngeal parenchymal progenitor
34 Protonephridia
35 Pharynx otf+ neuron
C
Volume
DV
ML
AP
Body volume (mm³)
Relative length
Cell number
Average cell volume
Relative volume
0 hpa
12 hpa
36 hpa
3 dpa
5 dpa
7 dpa
10 dpa
14 dpa
D
Int.5
Pha.19
Neu.25
Intestine
Neural
Pharynx
Volume proportion
R² = 0.4108
R² = 0.2022
R² = 0.7295
uninjured
E
foxA
pc2
pharynx
neural
F
Step 1
Step 2
Step 3
Pigment-based detection of blastema borders (watershed)
Region segmentation on body images
Cell labeling with regional information
Head
HAR
HBOR
HBIR
Trunk
Tail
TBIR
TBOR
TPR
G
Head blastema
Tail blastema
UMAP 1
UMAP 2
H
Head blastema
Tail blastema
Evi/Wls
sp5
Vang−2
agrin
admp−1a
Smed−egfr−4
Vang−1
MyoD−like
collagen 4−3
FRMD1
pou4l−1
rootletin−2
cav−1
ston
noggin 1
MAP3K−1
scna−2
noggin−like protein 8
Nrl
soxB
EGR−like protein 1
F−spondin
tspan−1
piwi protein Wl−3
folloid like−1
mmp2
tgs−1
Pir1
alpha tubulin
Smed−egf−1
cyclin B−1
piwi−1
cyclin B−2
Wnt5
EGR2
inhibin−1
Exp.
I
0hpa - 12hpa
12hpa - 36hpa
36hpa - 3dpa
3dpa - 5dpa
5dpa - 7dpa
7dpa - 10dpa
10dpa - 14dpa
Number of differentially expressed genes
Upregulated
Downregulated
SPC clusters
blastema regions

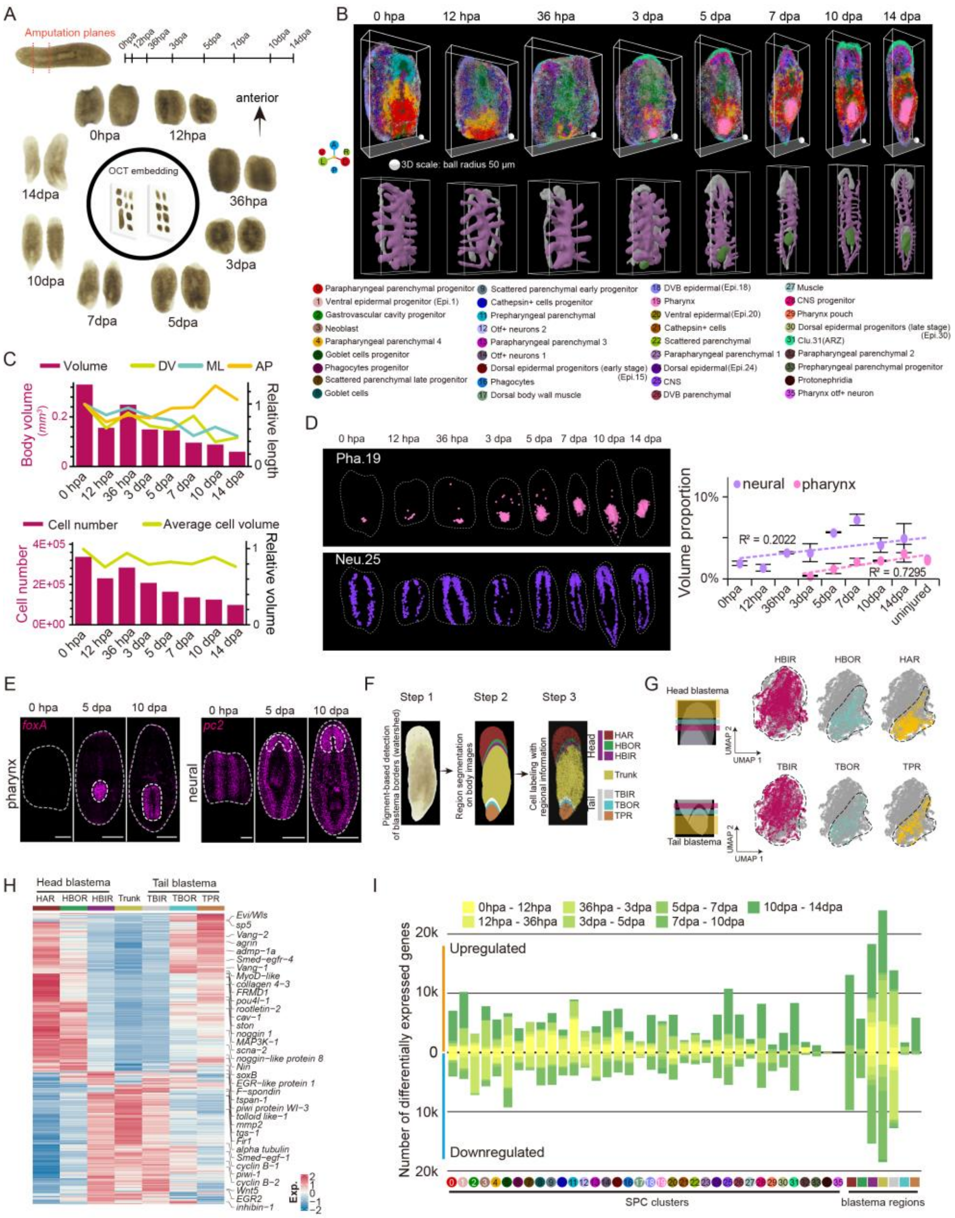

Figure. 1:. 3D molecular reconstruction of whole-body planarian regeneration using 4D

spatial transcriptomics.

(A) Schematic representation of the amputation strategy and sampling design for planarian whole-body regeneration (WBR). The dotted line indicates the amputated pre-pharyngeal fragments. The central diagram illustrates the arrangement of 17 embedded tissue samples per block, including one intact sample and two replicates at each of the eight time points post-amputation.

(B) 3D spatial visualization of 36 SPC clusters (top) and tissue meshes (bottom) in representative animals at eight time points during WBR. SPC clusters are labeled in the bottom panel. Tissue meshes highlight the intestine (purple), pharynx (green), and central nervous system (gray).

(C) Top: Bar plots showing changes in body volume size; line charts depicting variations in the length of the D/V, M/L, and A/P axes relative to the 0 hpa sample at eight time points of regeneration. Bottom: Bar plots showing SPC cell counts and line charts illustrating the average cell volume across the eight time points of regeneration.

(D) Left: Spatial patterns of the ~~intestine (Int.5),~~ pharynx (Pha.19) and CNS (Neu.25) clusters at eight regenerative time points. Right: Line plots showing the volume proportions of the reconstructed organs (~~Intestine,~~ neural and pharynx) during regeneration. Error bars represent the standard deviation from two replicates. Linear fitting lines show nearly linear time-dependent changes.

(E) FISH staining showing spatial patterns of pharynx (*foxA*) and neural (*pc2*) markers during regeneration. Scale bars: 500 µm. $n \geq 3$.

(F) Schematic diagrams illustrating the three-step process for identifying blastema subdomains based on the watershed algorithm for pigment variations. The head blastema region is divided into HAR (head anterior region), HBOR (head border outer region), and HBIR (head border inner region), while the tail blastema region includes TPR (tail posterior region), TBOR (tail border

outer region), and TBIR (tail border inner region).

(G) Left: Conceptual schematic illustrating the spatial definition of blastema sub-regions based on relative distance to the wound. Right: UMAP visualization of transcriptomic profiles from the corresponding cell populations. Colors indicate the distinct regions as defined in (F)~~UMAP representation of cell populations from the head and tail blastema shown in (F), with distinct regions color-coded to indicate separation~~.

(H) Heatmap depicting the relative expression of genes enriched in different blastema regions across all time points.

(I) Stacked bar plot illustrating the number of differentially expressed genes in each SPC cell type or blastema region under the indicated comparison conditions.

Formatted: Font: Bold

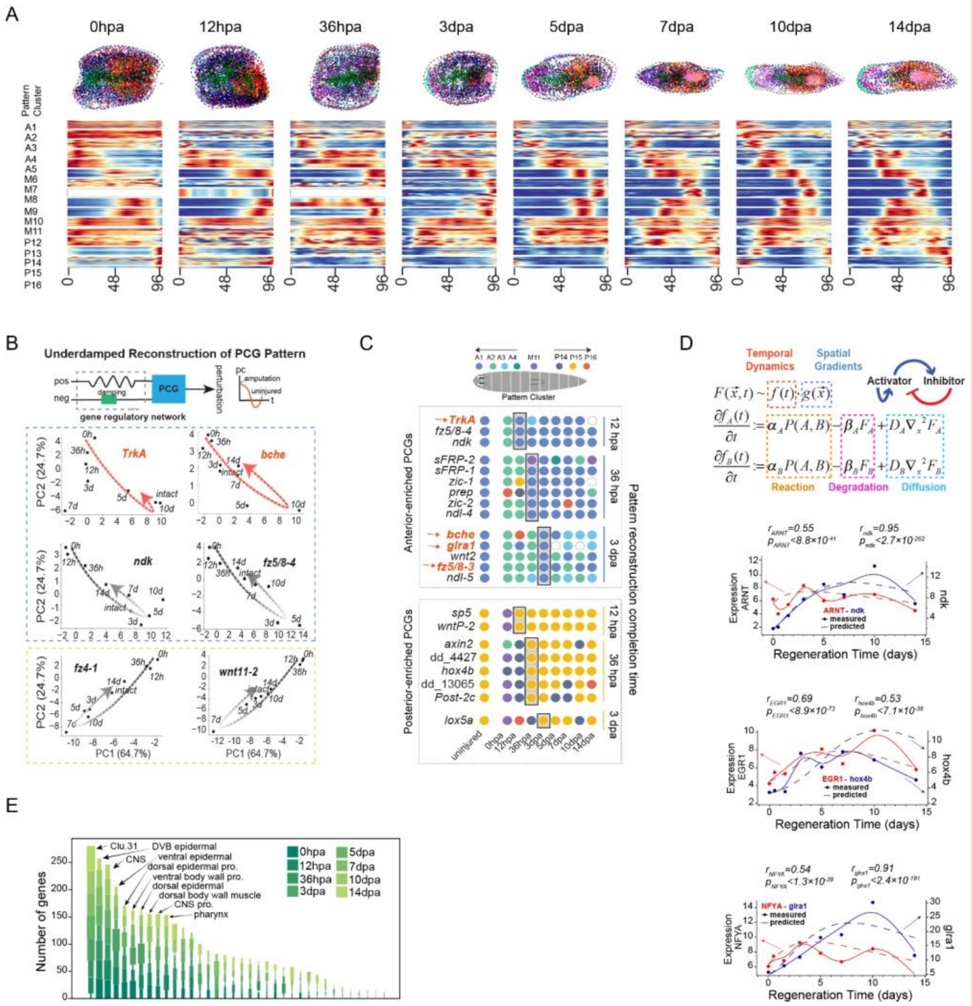
A
0hpa
12hpa
36hpa
3dpa
5dpa
7dpa
10dpa
14dpa
Pattern Cluster
A1
A2
A3
A4
A5
M6
M7
M8
M9
M10
M11
P12
P13
P14
P15
P16
0
48
96
B
Underdamped Reconstruction of PCG Pattern
pos
neg
damping
PCG
perturbation
amputation
uninjured
gene regulatory network
PC2 (24.7%)
PC1 (64.7%)
TrkA
bche
ndk
fz5/8-4
fz4-1
wnt11-2
intact
C
Pattern Cluster
Anterior-enriched PCGs
Posterior-enriched PCGs
TrkA
fz5/8-4
ndk
sFRP-2
sFRP-1
zic-1
prep
zic-2
ndl-4
bche
glra1
wnt2
fz5/8-3
ndl-5
sp5
wntP-2
axin2
dd_4427
hox4b
dd_13065
Post-2c
lox5a
12 hpa
36 hpa
3 dpa
Pattern reconstruction completion time
uninjured
0hpa
12hpa
36hpa
3dpa
5dpa
7dpa
10dpa
14dpa
D
Temporal Dynamics
Spatial Gradients
Activator
Inhibitor
Reaction
Degradation
Diffusion
Expression
Regeneration Time (days)
measured
predicted
ARNT - ndk
EGR1 - hox4b
NFYA - glra1
E
Number of genes
Clu.31
DVB epidermal
ventral epidermal
CNS
dorsal epidermal pro.
ventral body wall pro.
dorsal epidermal
dorsal body wall muscle
CNS pro.
pharynx
0hpa
12hpa
36hpa
3dpa
5dpa
7dpa
10dpa
14dpa

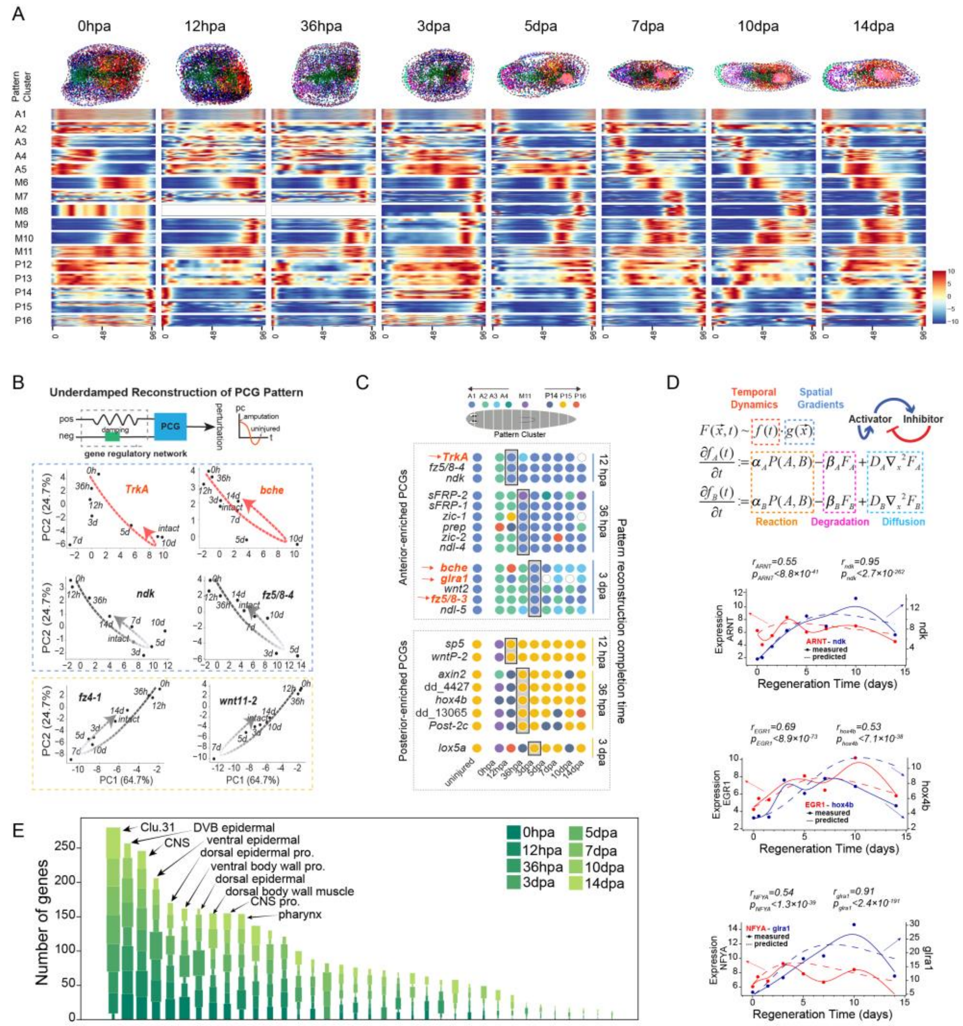
A
0hpa
12hpa
36hpa
3dpa
5dpa
7dpa
10dpa
14dpa
Pattern Cluster
A1
A2
A3
A4
A5
M6
M7
M8
M9
M10
M11
P12
P13
P14
P15
P16
B
Underdamped Reconstruction of PCG Pattern
pos
neg
damping
gene regulatory network
PCG
perturbation
amputation
uninjured
TrkA
bche
ndk
fz5/8-4
fz4-1
wnt11-2
PC2 (24.7%)
PC1 (64.7%)
C
Pattern Cluster
Anterior-enriched PCGs
Posterior-enriched PCGs
Pattern reconstruction completion time
TrkA
fz5/8-4
ndk
sFRP-2
sFRP-1
zic-1
prep
zic-2
ndl-4
bche
glra1
wnt2
fz5/8-3
ndl-5
sp5
wntP-2
axin2
dd_4427
hox4b
dd_13065
Post-2c
lox5a
12 hpa
36 hpa
3 dpa
uninjured
0hpa
12hpa
36hpa
3dpa
5dpa
7dpa
10dpa
14dpa
D
Temporal Dynamics
Spatial Gradients
Activator
Inhibitor
Reaction
Degradation
Diffusion
Regeneration Time (days)
measured
predicted
ARNT - ndk
EGR1 - hox4b
NFYA - glra1
E
Number of genes
Clu.31
DVB epidermal
CNS
ventral epidermal
dorsal epidermal pro.
ventral body wall pro.
dorsal epidermal
dorsal body wall muscle
CNS pro.
pharynx
0hpa
12hpa
36hpa
3dpa
5dpa
7dpa
10dpa
14dpa

Figure 2: Dynamics of spatially biased genes (SBGs) during whole-body regeneration.

(A) Heatmaps showing changes in spatial expression patterns along the A/P axis during regeneration for the 16 A/P pattern cluster genes. These genes exhibit spatially biased expression along the A/P axis in intact animals. Hollow rectangles indicate the absence of specific gene expression clusters at particular time points. Animals are virtually divided into 100 sections along the A/P axis. Left margin annotations indicate cluster numbers.

(B) PCA representation of pattern reconstruction for known and potential PCG candidates. PC1 corresponds to the head-tail gradient feature, while PC2 captures fluctuations in the pharynx region (convex and concave). Time-resolved trajectories in PCA space reveal universal self-organized dynamics during SBG pattern regeneration, resembling an underdamped mass-spring system (top).

(C) Schematic diagrams illustrating the hierarchical reconstruction of SBG patterns, colored by cluster ID. SBGs are arranged by the timing of repatterning completion, as shown on the right. Hollow circles indicate the absence of expression at specific time points. Red arrows highlight newly identified SBGs. The color gradient represents the recovery of SBGs at different stages post-amputation. The box highlights the time points when repatterning is complete.

(D) Spatiotemporal modeling of SBG patterns during regeneration. The interaction between SBGs and their upstream regulators generates Turing patterns in an autoregulatory activator-inhibitor system (top). Gene expression at any time point after amputation can be predicted. The lower line charts compare predicted repatterning (dashed line) with measured data (solid line) for both known and newly identified SBGs. r, Pearson’s correlation.

(E) Bar plot showing the number of SBGs highly enriched in each SPC cluster at different time points during regeneration.

1384
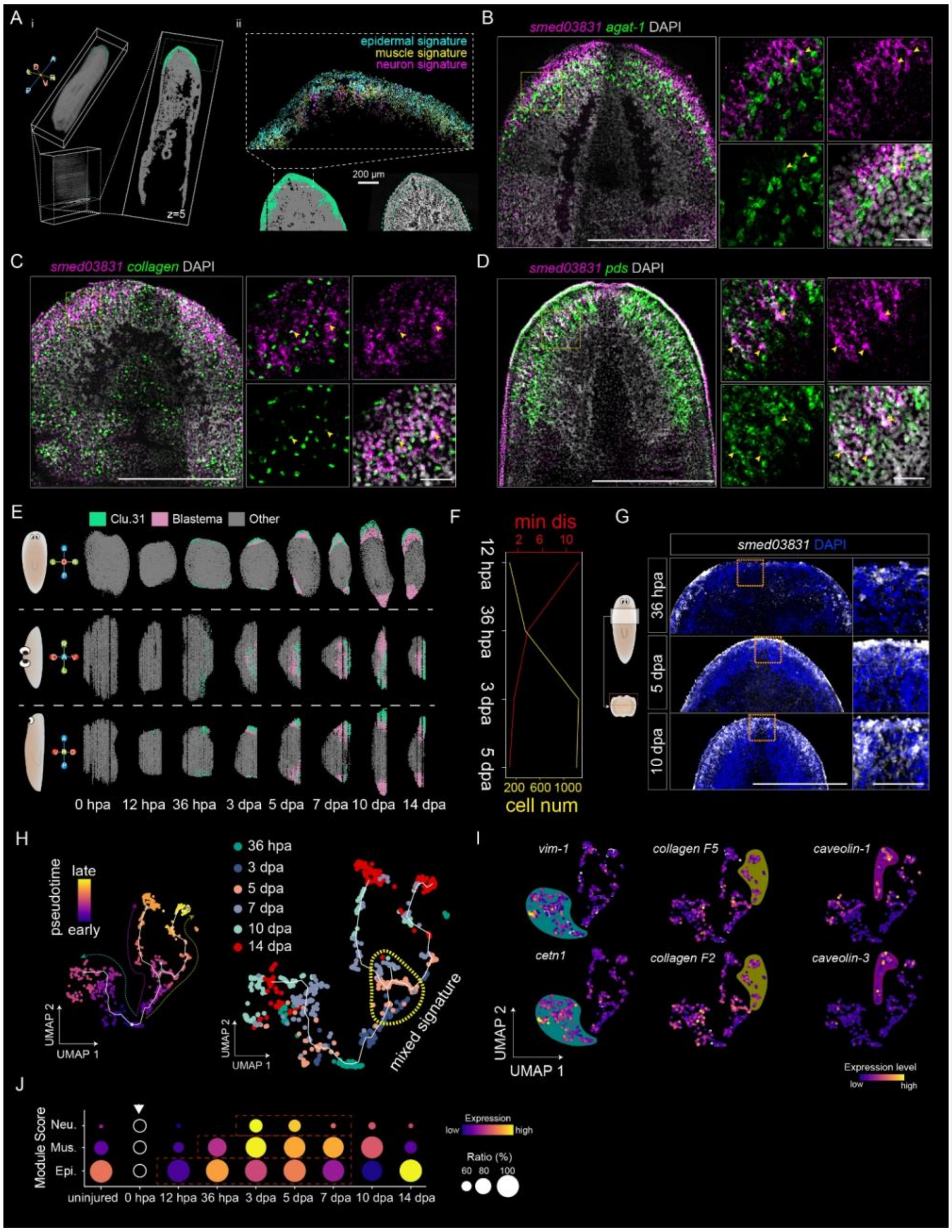

A
i
ii
epidermal signature
muscle signature
neuron signature
200 µm
z=5
B
smed03831 agat-1 DAPI
C
smed03831 collagen DAPI
D
smed03831 pds DAPI
E
Clu.31
Blastema
Other
0 hpa
12 hpa
36 hpa
3 dpa
5 dpa
7 dpa
10 dpa
14 dpa
F
min dis
2
6
10
12 hpa
36 hpa
3 dpa
5 dpa
200
600
1000
cell num
G
smed03831 DAPI
36 hpa
5 dpa
10 dpa
H
pseudotime
late
early
UMAP 2
UMAP 1
36 hpa
3 dpa
5 dpa
7 dpa
10 dpa
14 dpa
mixed signature
I
vim-1
collagen F5
caveolin-1
cetn1
collagen F2
caveolin-3
Expression level
low
high
J
Module Score
Neu.
Mus.
Epi.
uninjured
0 hpa
12 hpa
36 hpa
3 dpa
5 dpa
7 dpa
10 dpa
14 dpa
Expression
low
high
Ratio (%)
60
80
100

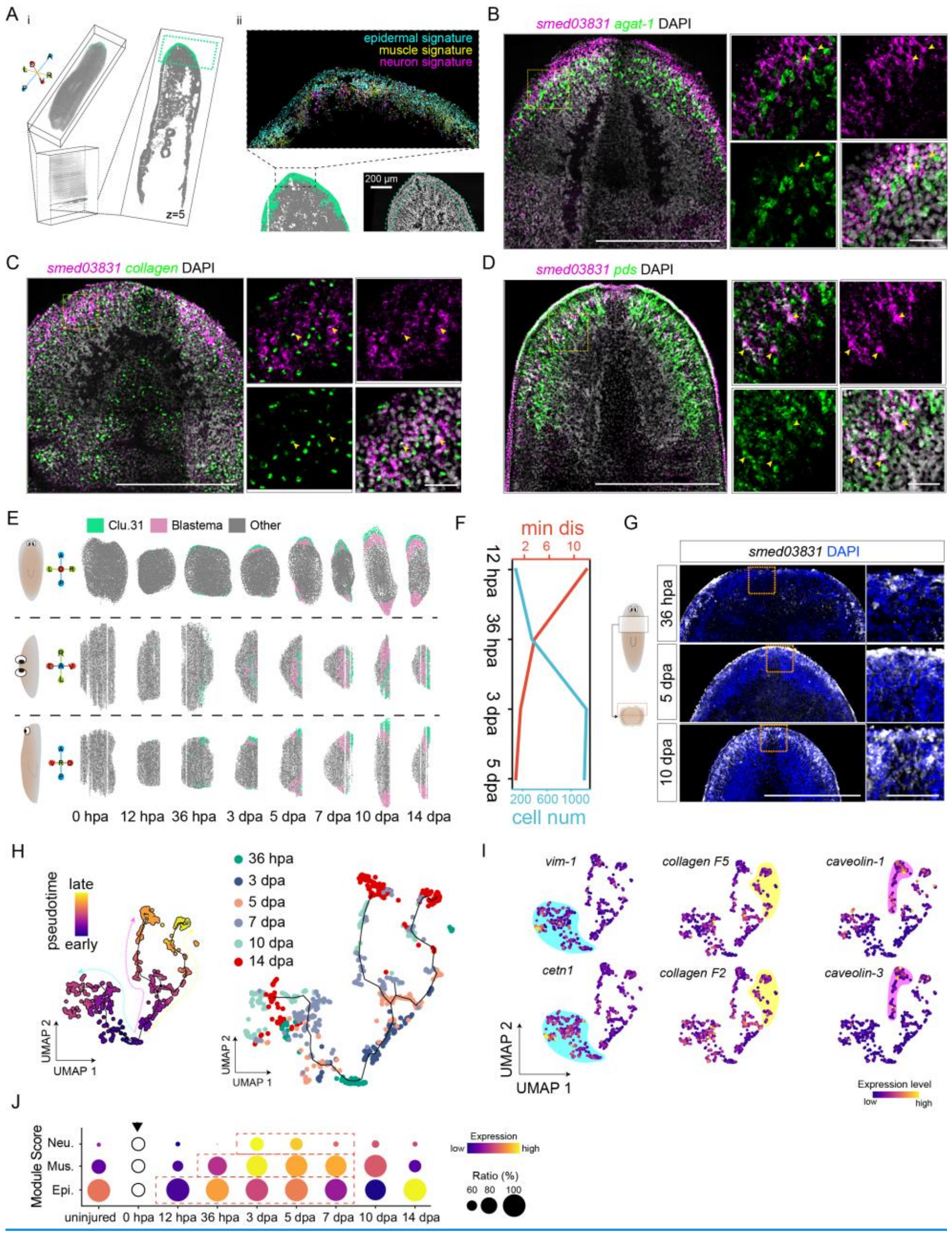
A i
ii
epidermal signature
muscle signature
neuron signature
z=5
200 μm
B
smed03831 agat-1 DAPI
C
smed03831 collagen DAPI
D
smed03831 pds DAPI
E
Clu.31
Blastema
Other
0 hpa
12 hpa
36 hpa
3 dpa
5 dpa
7 dpa
10 dpa
14 dpa
F
min dis
12 hpa
36 hpa
3 dpa
5 dpa
cell num
G
smed03831 DAPI
36 hpa
5 dpa
10 dpa
H
pseudotime
late
early
UMAP 1
UMAP 2
36 hpa
3 dpa
5 dpa
7 dpa
10 dpa
14 dpa
I
vim-1
collagen F5
caveolin-1
cetn1
collagen F2
caveolin-3
Expression level
low
high
J
Module Score
Neu.
Mus.
Epi.
uninjured
0 hpa
12 hpa
36 hpa
3 dpa
5 dpa
7 dpa
10 dpa
14 dpa
Expression
low
high
Ratio (%)
60 80 100

1385

Figure 3: Identification and characterization of the ARZ (Clu.31) domain in the blastema region.

(A) Spatial visualization of the Clu.31 domain in a homeostatic worm. (i) 3D spatial transcriptomics data showing Clu.31 (green) with the right panel displaying the fifth section from ventral to dorsal (z = 5). (ii) Top: Enlarged view of three lineage signatures within Clu.31 in a single slice. Bottom left: Enlarged view of the head region from (i). Bottom right: ssDNA staining highlights the presence of multiple cell layers coexisting in Clu.31, with the green dashed line indicating the location of Clu.31.

(B-D) FISH staining for the Clu.31 marker (*smed03831*, magenta) with the epidermal marker agat-1 (green) (B), muscle marker collagen (green) (C), and neuron marker pds (green) (D). Co-expressed cells are indicated by yellow arrowheads. Scale bars: 500 µm (left); 50 µm (right). n ≥ 3.

(E) Spatial distribution of the Clu.31 domain during regeneration, shown from top, front, and side views. Green dots represent SPC cells within Clu.31, pink dots represent SPC cells in the blastema region, and grey dots represent other SPCs.

(F) The position and cell number of Clu.31 during regeneration. Top: Line plot (red) showing the decreased minimal distance (min dis) of Clu.31 to the wound surface, accompanied by an increase in the cell number (Cell num) of Clu.31 during wound healing (yellow line). Min dis represents the minimal distance to the wound surface in UV spatial coordinates, while Cell num refers to the number of Clu.31 cells.

(G) FISH staining showing *smed03831* expression in the head blastema during regeneration. Enlarged areas are shown to the right. n ≥ 3. Scale bars: 500 µm (left); 50 µm (right).

(H) Pseudotime trajectory analysis of Clu.31 across six time points of whole-body regeneration

(WBR), from 36 hpa to 14 dpa. SPC cells exhibiting mixed neural, muscular, and epidermal markers are indicated by a yellow dashed circle.

(I) Feature plot showing the expression of representative cell-type marker genes—epidermis (left), muscle (middle), and neuronal (right) lineages—along pseudotime trajectories from (H).

(J) Bubble plot displaying the gene set module scores of markers for neural (Neu.), muscular (Mus.), and epidermal (Epi.) lineages within Clu.31 during regeneration.

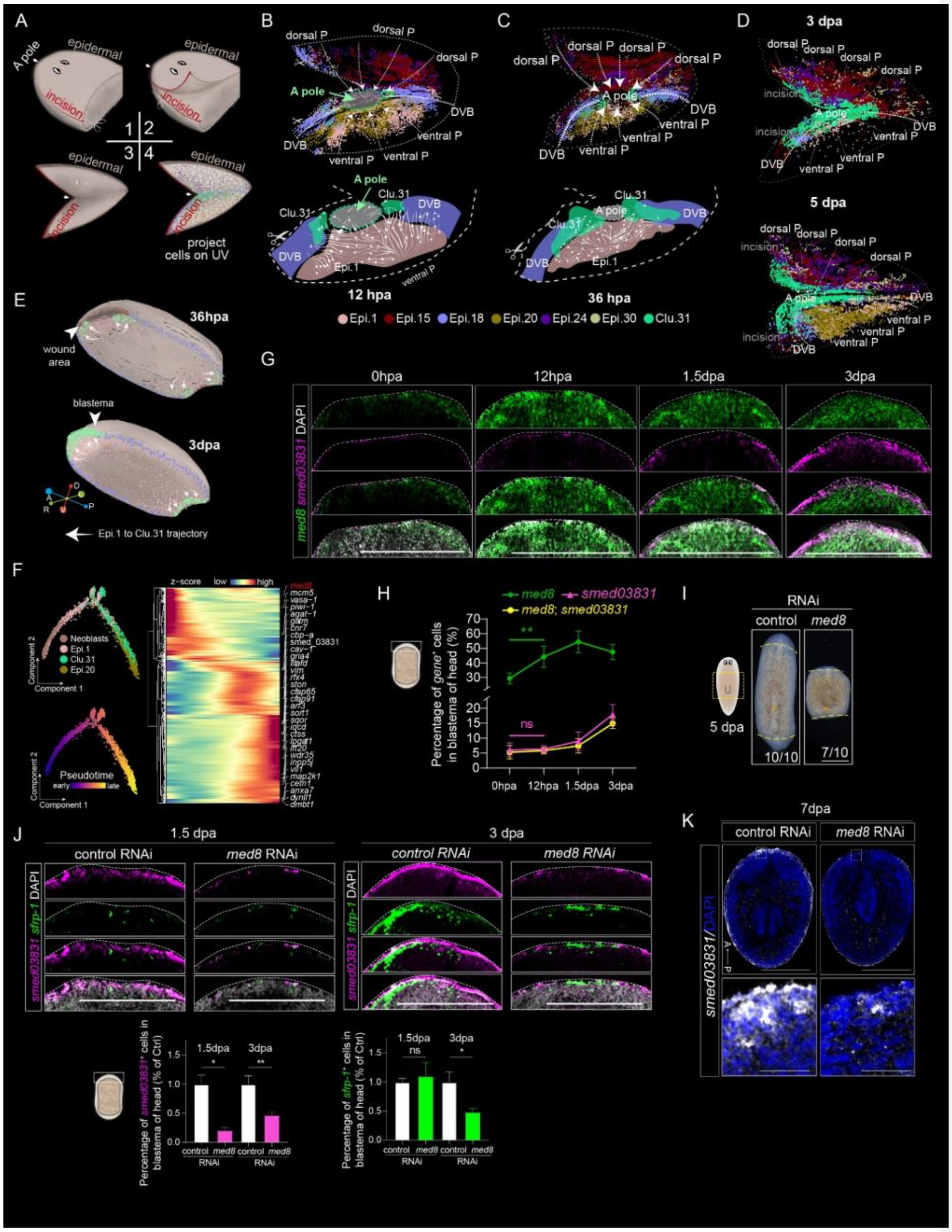
A
A pole
epidermal
incision
1 2
3 4
project
cells on UV
B
dorsal P
A pole
DVB
ventral P
Clu.31
Epi.1
12 hpa
C
36 hpa
D
3 dpa
5 dpa
incision
Epi.1 Epi.15 Epi.18 Epi.20 Epi.24 Epi.30 Clu.31
E
36hpa
wound
area
blastema
3dpa
Epi.1 to Clu.31 trajectory
G
0hpa
12hpa
1.5dpa
3dpa
med8 smed03831 DAPI
F
Neoblasts
Epi.1
Clu.31
Epi.20
Component 1
Component 2
Pseudotime
early
late
z-score low high
H
med8
smed03831
med8; smed03831
Percentage of gene+ cells in blastema of head (%)
ns
I
RNAi
control
med8
5 dpa
10/10
7/10
J
1.5 dpa
3 dpa
control RNAi
med8 RNAi
smed03831 sfrp-1 DAPI
Percentage of smed03831+ cells in blastema of head (% of Ctrl)
Percentage of sfrp-1+ cells in blastema of head (% of Ctrl)
K
7dpa
smed03831/DAPI

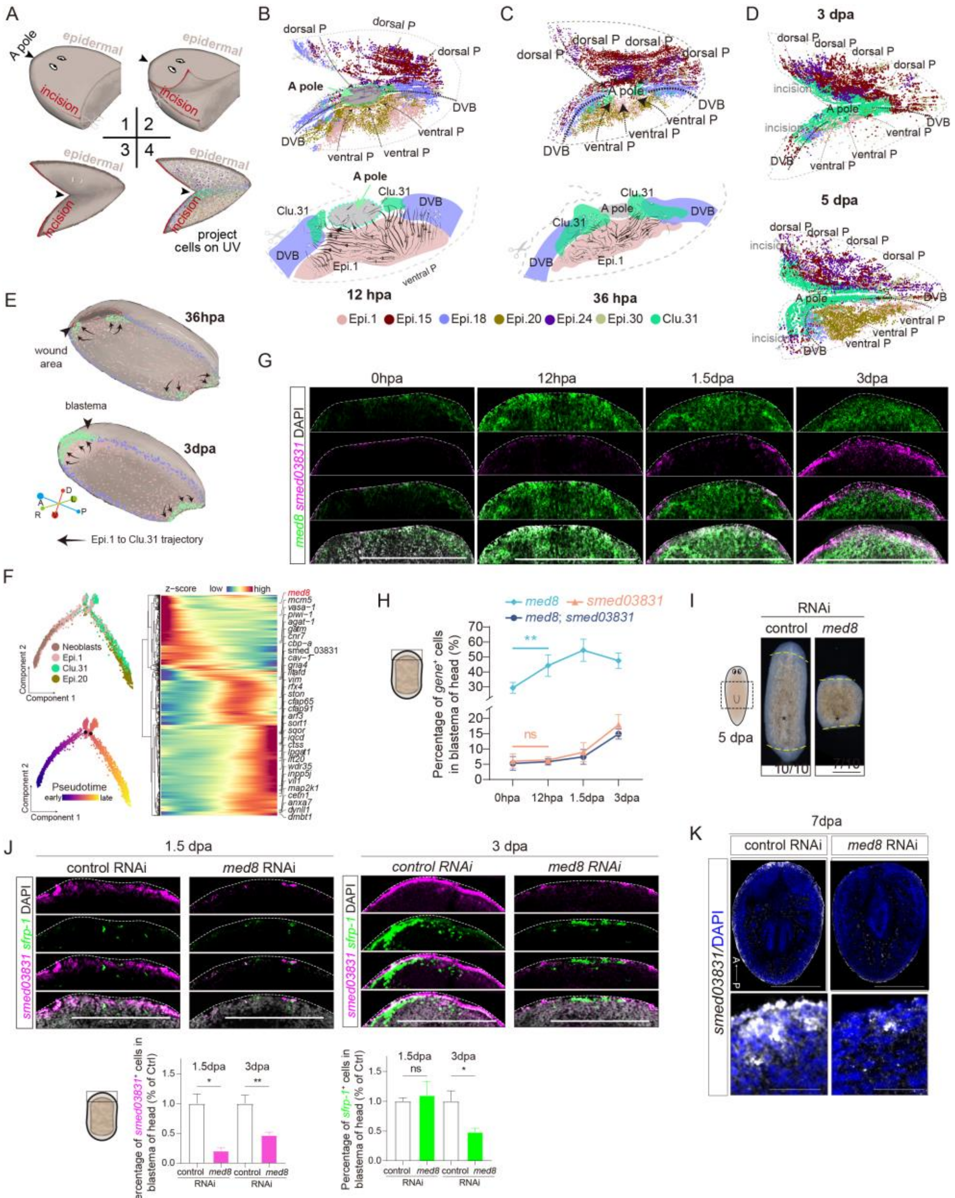
A
A pole
epidermal
incision
1
2
3
4
project cells on UV
B
dorsal P
A pole
DVB
ventral P
Clu.31
Epi.1
12 hpa
C
36 hpa
D
3 dpa
5 dpa
Epi.1
Epi.15
Epi.18
Epi.20
Epi.24
Epi.30
Clu.31
E
36hpa
wound area
blastema
3dpa
Epi.1 to Clu.31 trajectory
F
Neoblasts
Epi.1
Clu.31
Epi.20
Component 1
Component 2
Pseudotime
early
late
z-score
low
high
med8
G
0hpa
12hpa
1.5dpa
3dpa
med8 smed03831 DAPI
H
med8
smed03831
med8; smed03831
Percentage of gene+ cells in blastema of head (%)
ns
0hpa
12hpa
1.5dpa
3dpa
I
RNAi
control
med8
5 dpa
10/10
J
1.5 dpa
3 dpa
control RNAi
med8 RNAi
smed03831 sfrp-1 DAPI
Percentage of smed03831+ cells in blastema of head (% of Ctrl)
Percentage of sfrp-1+ cells in blastema of head (% of Ctrl)
1.5dpa
3dpa
ns
control
med8
RNAi
K
7dpa
control RNAi
med8 RNAi
smed03831/DAPI

Figure~~.~~ 4:~~.~~ Cellular composition and regulation of the ARZ domain.

(A) Schematic illustrating the workflow for unwrapping the 3D epidermal surface of the head blastema into 2D spatial coordinates. UV unwrapping of the 3D mesh was performed by manually marking a seam along the D/V boundary (Step 1) and unwrapping the surface by cutting along the seam (Steps 2, 3). Epidermal cells were projected onto the 2D coordinates by minimizing the distance between each 3D cell center and the nearest subdivision vertices (Step 4). See ~~Materials and~~ Methods for details.

(B) UV spatial mapping of the ventral epidermal transition forming the Clu.31 domain at 12 hours post-amputation (hpa). Top: Distribution of Clu.31 (green) and epidermal SPCs on the unwrapped UV map, with dot colors representing SPC types. Labels and dashed lines indicate key positions. Bottom: Predicted cell transition streams of the epidermis as modeled using Dynamo. DVB, Dorsal-Ventral Boundary; A pole, anterior pole; P, posterior.

(C) UV spatial map showing Clu.31 transition patterns at 36 hpa. Top: Distribution of Clu.31 and epidermal SPCs on the unwrapped UV spatial map. Bottom: Cell transition streams of the epidermis predicted using Dynamo.

(D) UV spatial map showing Clu.31 transition patterns at 3 days post-amputation (dpa, top) and 5 dpa (bottom). The distribution of Clu.31 (green) and epidermal SPCs on the unwrapped UV spatial map is shown.

(E) Visualization of Clu.31 movement during wound healing (top, 36 hpa) and blastema formation (bottom, 3 dpa). The white arrow indicates the predicted trajectory of the epidermal transition, delineated based on the spatial dynamo shown in (C) and (D). Arrowheads highlight the location of either the wound surface (top) or blastema (bottom).

(F) Pseudotime trajectory analysis of ventral epidermis and neoblast cells. Left: Distinct states of

SPCs identified by pseudotime analysis, with cells colored by SPC clusters (top left) and pseudotime (bottom left). Right: Heatmap showing significantly altered genes discovered by Monocle 2 along the trajectory.

(G) Expression and localization of *med8* and *smed03831* in the head blastema of regenerative fragments at the indicated time points. Scale bars, 500 µm.

(H) Percentage of *med8*$^+$, *smed03831*$^+$, or co-expressing cells in the blastema shown in (G). ns, $p > 0.05$; $^{**}p < 0.01$; two-tailed unpaired *t*-test.

(I) Representative phenotypes following *med8* RNAi at 5 dpa. n = 10 animals for each condition. Scale bars: 500 µm.

(J) Expression and localization of *smed03831* and *sfrp-1* in the blastema of control and *med8* RNAi animals at 1.5 (top left) and 3 dpa (top right). Scale bars: 500 µm. The ratio of *smed03831*- or *sfrp-1*-expressing cells in the blastema was quantified (bottom). ns, $p > 0.05$; $^{*}p < 0.05$; $^{**}p < 0.01$; two-tailed unpaired *t*-test.

(K) FISH staining of *smed03831* in control and *med8* RNAi animals. n ≥ 3 animals per condition. Scale bars: 500 µm (top); 50 µm (bottom).

1456

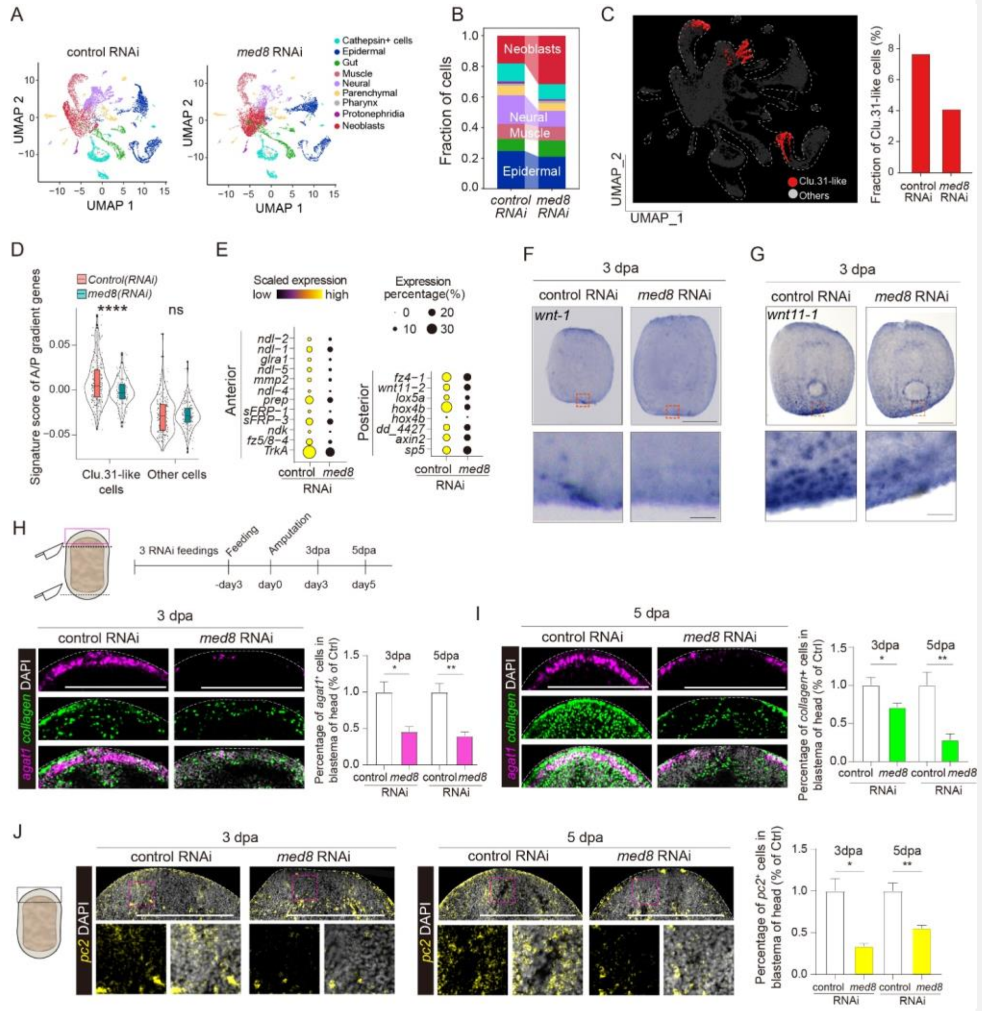
A
control RNAi
med8 RNAi
Cathepsin+ cells
Epidermal
Gut
Muscle
Neural
Parenchymal
Pharynx
Protonephridia
Neoblasts
UMAP 1
UMAP 2
B
Fraction of cells
Neoblasts
Neural
Muscle
Epidermal
control RNAi
med8 RNAi
C
Clu.31-like
Others
UMAP_1
UMAP_2
Fraction of Clu.31-like cells (%)
D
Control(RNAi)
med8(RNAi)
****
ns
Signature score of A/P gradient genes
Clu.31-like cells
Other cells
E
Scaled expression
low
high
Expression percentage(%)
Anterior
ndl-2
ndl-1
glra1
ndl-5
mmp2
ndl-4
prep
sFRP-1
sFRP-3
ndk
fz5/8-4
TrkA
Posterior
fz4-1
wnt11-2
lox5a
hox4b
hox4b
dd_4427
axin2
sp5
control
med8
RNAi
F
3 dpa
wnt-1
G
wnt11-1
H
3 RNAi feedings
Feeding
Amputation
-day3
day0
day3
day5
3dpa
5dpa
agat1 collagen DAPI
Percentage of agat1+ cells in blastema of head (% of Ctrl)
I
5 dpa
Percentage of collagen+ cells in blastema of head (% of Ctrl)
J
pc2 DAPI
Percentage of pc2+ cells in blastema of head (% of Ctrl)

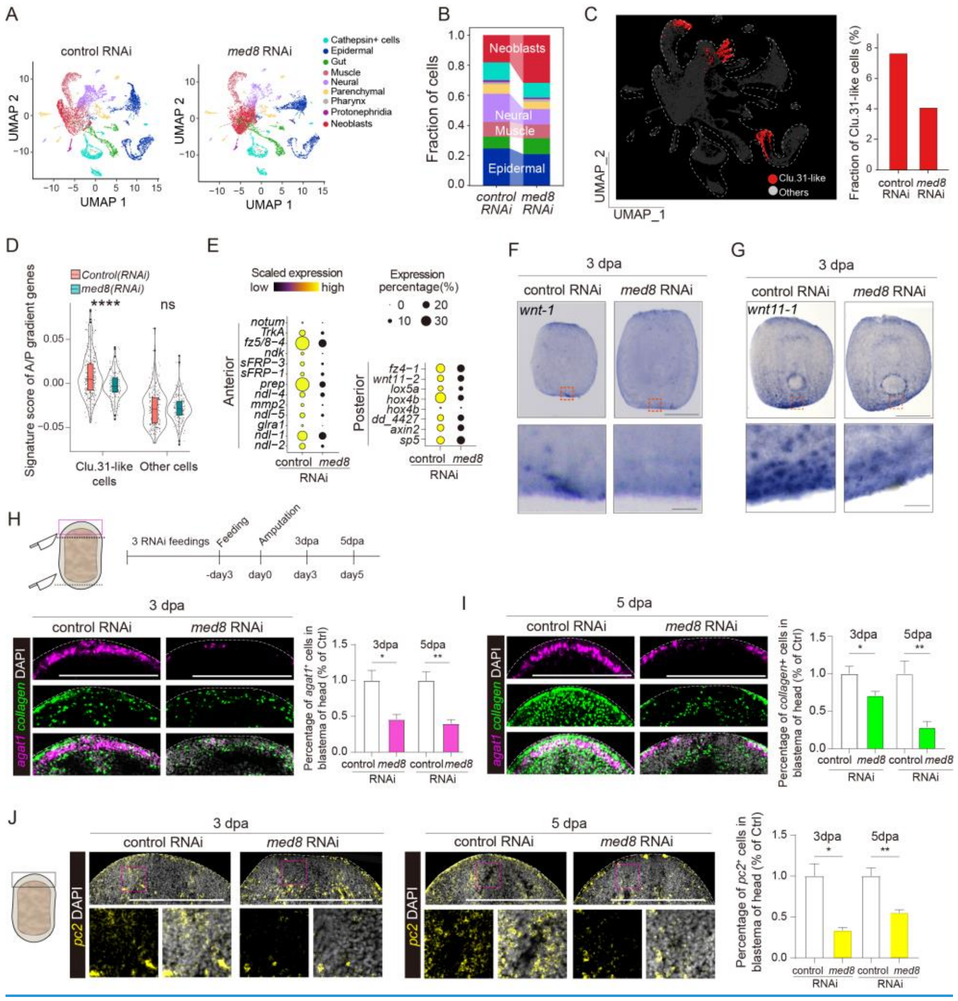
A
control RNAi
med8 RNAi
Cathepsin+ cells
Epidermal
Gut
Muscle
Neural
Parenchymal
Pharynx
Protonephridia
Neoblasts
UMAP 1
UMAP 2
B
Fraction of cells
Neoblasts
Neural
Muscle
Epidermal
control RNAi
med8 RNAi
C
Clu.31-like
Others
UMAP_1
UMAP_2
Fraction of Clu.31-like cells (%)
control RNAi
med8 RNAi
D
Control(RNAi)
med8(RNAi)
Signature score of A/P gradient genes
Clu.31-like cells
Other cells
****
ns
E
Scaled expression
low
high
Expression percentage(%)
Anterior
notum
TrkA
fz5/8-4
ndk
sFRP-3
sFRP-1
prep
ndl-4
mmp2
ndl-5
glra1
ndl-1
ndl-2
Posterior
fz4-1
wnt11-2
lox5a
hox4b
hox4b
dd_4427
axin2
sp5
control
med8
RNAi
F
3 dpa
control RNAi
med8 RNAi
wnt-1
G
3 dpa
control RNAi
med8 RNAi
wnt11-1
H
3 RNAi feedings
Feeding
Amputation
3dpa
5dpa
-day3
day0
day3
day5
3 dpa
control RNAi
med8 RNAi
DAPI
agat1
collagen
Percentage of agat1+ cells in blastema of head (% of Ctrl)
3dpa
5dpa
I
5 dpa
control RNAi
med8 RNAi
Percentage of collagen+ cells in blastema of head (% of Ctrl)
J
3 dpa
5 dpa
control RNAi
med8 RNAi
pc2
DAPI
Percentage of pc2+ cells in blastema of head (% of Ctrl)

Figure 5: *Med8*-dependent regulation of anterior regenerative zone and blastema formation.

(A) UMAP visualization of scRNA-seq analysis depicting cell lineages from control and *med8* RNAi-treated tail fragments.

(B) Bar plot showing the changes in cell populations following *med8* RNAi knockdown.

(C) Left: UMAP visualization of neural, muscular, and epidermal cells highlighting the ARZ signature (Clu.31-like) in scRNA-seq data. Right: Bar plot illustrating the decrease in the fraction of ARZ (Clu.31) cells in *med8* RNAi-treated animals compared to controls.

(D) Violin plot showing changes in the module score of A/P gradient genes in ARZ (Clu.31) cells. p-values are from the Wilcoxon test: ns, $p > 0.05$; ****, $p < 0.0001$.

(E) Dot plot illustrating the relative expression of representative PCGs in anterior or posterior regions from scRNA-seq data.

(F-G) WISH analysis showing the expression and localization of posterior markers *wnt-1* (F) and *wnt11-1* (G) in control and *med8* knockdown animals. Scale bars: 500 µm (top), 50 µm (bottom). n = 6 animals with consistent results.

(H-I) Expression and localization of muscle marker *collagen* and epidermal marker *agat-1* in the blastema of control and *med8* RNAi animals at 3 (H) and 5 days post-amputation (dpa) (I), respectively. Scale bars: 500 µm. n = 6 animals with consistent results. The percentage of positive cells in the blastema was quantified. ns, $p > 0.05$; *$p < 0.05$; **$p < 0.01$, two-tailed unpaired t-test.

(J) Expression and localization of the neural marker *PC2* in the blastema of control and *med8* RNAi animals at 3 and 5dpa. Scale bars: 500 µm. The percentage of positive cells in the blastema was quantified (right). *$p < 0.05$; **$p < 0.01$, two-tailed unpaired t-test.

All FISH images are maximum-intensity projections. Error bars represent SEM. n ≥ 3 biologically independent experiments.

Formatted: Font: Italic

Formatted: Font: Italic

Revised manuscript clean version

Click here to access/download;Manuscript;Manuscript-clean-20260224.docx

4D Single-Cell Spatial Transcriptomics Reveals Dynamic Morphogenetic Gradients and Regenerative Domains in Planarians

Kai Han[1,3,†], Yue Chen[2,†], Yao Li[1,†], Lidong Guo[1,4], Yuxiaofei Wang[2], Xiawei Liu[1], Yaru Lin[2], Zhi Huang[1,5], Qun Liu[1,3], Wenjie Guo[1], Rui Zhang[1,3], Wandong Zhao[1], Langchao Liang[1,4], Xiaoyu Wei[6], Li Zhou[1], Xuebin Mao[1], Jiaqi Wang[1], Weijian Wu[1], Hongwei Pan[1], Tao Yang[7], He Zhang[1], Xiaoshan Su[1,3], Shanshan Liu[1], Wenwei Zhang[6], Longqi Liu[6], Søren Tvorup Christensen[3], Jifeng Fei[8], Xin Liu[1,6,9], Guangyi Fan[1,6,9], Hanbo Li[1,6,10], Ying Gu[6], Jian Wang[6], Huanming Yang[6], Gang Pei[2], Xun Xu[6,9], An Zeng[2,*], Mengyang Xu[1,6,*]

[1]BGI Research, Qingdao 266555, China

[2]Key Laboratory of Multi-Cell Systems, Shanghai Institute of Biochemistry and Cell Biology, Center for Excellence in Molecular Cell Science, Chinese Academy of Sciences, University of Chinese Academy of Sciences, Shanghai 200031, China

[3]Department of Biology, University of Copenhagen, DK-2100 Copenhagen OE, Denmark

[4]College of Life Sciences, University of Chinese Academy of Sciences, Beijing 100049, China

[5]School of Biology and Biological Engineering, South China University of Technology, Guangzhou, 510006, China

[6]BGI Research, Shenzhen 518083, China

[7]China National GeneBank, BGI Research, Shenzhen 518083, China

[8]Department of Pathology, Guangdong Provincial People's Hospital (Guangdong Academy of Medical Sciences), Southern Medical University, Guangzhou, Guangdong 510080, China

[9]State Key Laboratory of Agricultural Genomics, BGI Research, Shenzhen 518083, China

[10]Lars Bolund Institute of Regenerative Medicine Qingdao-Europe Advanced Institute for LifeSciences, BGI Research, Qingdao 266555, China.

[†]These authors contributed equally to this work.

*Correspondence: azeng@sibcb.ac.cn (A. Z.); xumengyang@genomics.cn (M. X.)

Abstract

Background: Understanding how organisms reconstruct complex tissue architectures following injury requires precise mapping of gene expression and cellular responses across space and time. Although planarians serve as a classic model for whole-body regeneration, capturing the continuous spatiotemporal dynamics of positional information and cell fate decisions at the organismal scale remains a significant challenge.

Results: Using high-definition spatial transcriptomics, we generated a four-dimensional atlas encompassing over 3.5 million cells from whole animals across eight distinct regeneration timepoints. This comprehensive dataset enabled the definition of 36 spatial domains and the tracing of body axis restoration, revealing that positional control genes recover through self-organizing dynamics analogous to an underdamped control system. We identified a transient injury-induced spatial domain termed the Anterior Regenerative Zone. This unique region is characterized by the convergence of epidermal, muscular, and neural lineages enriched with positional signals. Furthermore, we demonstrated that the transcriptional co-factor Mediator 8 is a critical regulator of this zone. Depletion of Mediator 8 impairs the formation of the Anterior Regenerative Zone, disrupts polarity establishment, and prevents successful blastema formation.

Conclusions: Our study provides a holistic molecular and cellular reconstruction of whole-body regeneration, directly linking dynamic gene expression gradients to morphological restoration. The discovery of the Mediator 8-regulated Anterior Regenerative Zone highlights the importance of transient spatial domains in coordinating tissue repair. The resulting interactive atlas serves as a foundational resource for deciphering the logic of spatiotemporal patterning in regeneration.

Keywords: Planarian regeneration, Spatial transcriptomics, Positional gradients, Regenerative

zone, Single-cell atlas, Pattern formation

Background

Understanding the mechanisms of tissue and organ regeneration following injury is a fundamental biological question with profound implications for regenerative medicine, wound repair, and aging. Regeneration involves a complex series of events, including local and systemic responses to injury, the restoration of positional information, and the formation of new tissue structures [1, 2]. While progress has been made in understanding these processes, several critical questions remain [3, 4]. How do cells spatially respond to injury, and how do molecular gradients influence tissue patterning? What roles do specific spatial domains, morphogen gradients, and injury-responsive regions play in regeneration across complex organisms? Furthermore, how can we capture and quantify these processes at the molecular level across an entire organism?

To address these questions, profiling organisms, cells, and genes across multiple spatial and temporal scales is crucial [5]. However, capturing continuous positional signals at molecular, cellular, and organismal levels remains a significant challenge, especially in three-dimensional space and time [6]. The complexity of tissue heterogeneity, large body sizes, and the preservation of cellular and molecular organization in extensive tissue sections complicate spatial transcriptomic analyses. Moreover, the absence of quantitative assays and frameworks to capture continuous positional signals at the transcriptome level in three-dimensional space and time presents an additional technical obstacle. This challenge is further compounded by the limited availability of classical model organisms that can fully regenerate their bodies. To date, a comprehensive three-dimensional molecular reconstruction of cellular architecture and morphogen gradients across an entire organism has not been achieved, limiting our understanding of the dynamic changes in cellular and molecular identities during regeneration.

Planarians, renowned for their exceptional regenerative abilities, serve as an ideal model for studying the spatial and temporal dynamics of tissue regeneration [3]. These bilateral metazoans possess a complex anatomy [7], including a brain, nerve cords, peripheral nervous system, epidermis, intestine, muscles, excretory system, and a centrally located pharynx. Composed of a variety of domains derived from three germ layers, planarians rely on pluripotent stem cells, or neoblasts, for constant tissue turnover and regeneration [8, 9]. They utilize precise positional cues to guide body axis establishment and tissue patterning [10, 11]. Numerous genes involved in signaling pathways for body plan patterning have been identified, expressed in a complex spatial map across the dorsoventral (D/V), mediolateral (M/L), and anteroposterior (A/P) axes [12-15]. These genes, known as position control genes (PCGs), are largely expressed in muscle tissue and play a critical role in regulating positional information during regeneration [16]. However, the precise phenotypic outcomes associated with many of these genes remain poorly understood, and it is still unclear whether positional information is confined exclusively to muscle tissue [16]. The regenerative process requires cells to establish, record, and interpret positional information to rebuild the body's complex structure. This highlights the importance of profiling genes and domains across multiple spatial and temporal scales. Although advances in single-cell RNA sequencing (scRNA-seq) [17-23] and spatial transcriptomics (ST) [24, 25] have enabled the profiling of domains and gene expression patterns, these technologies still face limitations in achieving high spatial resolution at both the cellular and organismal levels. Furthermore, the mechanisms by which injury-induced local signals guide stem cells to reconstruct the body axis and regenerate fully functional three-dimensional structures remain poorly understood [3, 26]. Thus, there is a need for comprehensive analytical frameworks that can capture the dynamic molecular and cellular events of regeneration across entire organisms.

In this study, we used Stereo-seq [27] and our custom framework [28] to create an extensive atlas of 3,508,004 segmented cells from 353 sections of 16 complete planarians, spanning eight time points of whole-body regeneration. With a resolution of 715 nm, we generated detailed transcriptional and anatomical maps of the regeneration process, constructing positional and transcriptional gradients along the body axis. Our 4D transcriptomic atlas, annotated with 36 refined domains, provides a comprehensive view of gene expression dynamics across cellular, tissue, and organismal scales. Our findings offer key insights into the regenerative process. First, we reveal complete spatial gene expression patterns that define positional gradients along the body axes, tracking their dynamic spatiotemporal fluctuations following amputation. Additionally, we identify an injury-induced Anterior Regenerative Zone (ARZ), marked by *ROD1*, which exhibits enriched positional signals in epidermal, muscle, and neural cells. The ARZ is regulated by Mediator 8 (*med8*), which is crucial for polarity establishment, blastema formation, and overall regeneration. These results provide a comprehensive molecular and spatial map of regenerative processes, highlighting dynamic changes in regeneration-responsive cells, spatial domains, and key regulatory factors.

Data Description

To comprehensively map the spatiotemporal dynamics of whole-body regeneration, we employed high-resolution Stereo-seq (715 nm) on 353 cryosections derived from 16 *Schmidtea mediterranea* individuals across eight regeneration timepoints (0 hours to 14 days). This 4D reconstruction yielded 3,508,004 segmented cells, from which we annotated 36 distinct cell clusters and identified the injury-induced Anterior Regenerative Zone. This dataset enables genome-wide transcriptional imputation across body axes and provides a foundational resource for modeling morphogenetic gradients and regenerative patterning. Raw sequencing data are deposited in the CNGB Nucleotide

Sequence Archive under accession STT0000028, while processed data, code, and 3D visualizations are accessible via the PRISTA4D interactive portal (https://db.cngb.org/stomics/prista4d) and GitHub for unrestricted community reuse.

Analyses

Reconstruction of planarian 4D spatiotemporal transcriptomes at single-cell resolution

To investigate the cellular and molecular dynamics of regeneration, we generated a comprehensive 4D atlas of gene expression and cellular changes during the regeneration process (Methods). We focused on pre-pharyngeal amputations, which regenerate the head, tail, and pharynx over a two-week period (Fig. 1A). Using the Stereo-seq technique [27], which integrates tissue cryo-sectioning with *in situ* RNA sequencing at 715 nm resolution, we profiled gene expression at multiple stages of regeneration. Animals were collected at eight distinct time points: 0, 12, and 36 hours post-amputation (hpa), and 3, 5, 7, 10, and 14 days post-amputation (dpa). For each time point, two animals were sampled, resulting in a total of 16 regenerating animals. These animals were sectioned along the dorsal-ventral axis to capture the entire organism (Supplementary Fig. S1A). The 16 animals were processed into 10-μm-thick sections, producing a total of 353 slices for spatial transcriptomics analysis using the Stereo-seq platform (Fig. 1A and Supplementary Fig. S1A).

To facilitate the generation of a comprehensive 3D reconstruction, we aligned and stitched the individual tissue sections (Methods). This resulted in 3,508,004 segmented cells across the 16 reconstructed animals, with cell counts ranging from 58,450 to 432,197 per time point after quality control (UMIs per cell >50) (Fig. 1B, Supplementary Fig. S1A, and Supplementary Table S1). This spatial transcriptomics atlas spans all eight time points, providing a high-resolution, 4D view

of regenerating planarians at subcellular resolution (Fig. 1B and Supplementary Fig. S1B). The dataset allows for the tracking of the spatial dynamics of regeneration-responsive genes and cellular interactions at various stages of regeneration. To validate the quantitative accuracy of our spatial transcriptomic atlas, we performed a correlation analysis between the Stereo-seq data (aggregated as pseudo-bulk) and public bulk RNA-seq data [29]. The high correlation coefficient ($R = 0.8$, Supplementary Fig. S1C) demonstrates the robust reliability and technical reproducibility of our spatial dataset. The dataset is publicly available via our searchable browser: https://db.cngb.org/stomics/prista4d (Supplementary Fig. S1D).

To enhance the identification of biologically relevant tissue domains, we combined gene expression data with spatial information using the spatial proximity-based clustering (SPC) method. This method groups cells based on both transcriptional similarity and spatial proximity (Methods). Using SPC, we identified 36 distinct spatial domains (Fig. 1B). Correlation analysis with published single-cell datasets revealed that the majority of these domains represent lineage-restricted populations (Supplementary Fig. S2, A, B and C). Furthermore, comparison with published 10x Genomics Visium datasets confirmed consistent spatial transcriptional signatures across platforms (Supplementary Fig. S2D). Together, these systematic cross-platform validations confirmed the accuracy of our cell identities while highlighting the superior resolution of Stereo-seq in resolving fine-grained spatial heterogeneity within broader tissue domains [28]. For example, our analysis revealed significant spatial heterogeneity in the parenchyma, which was subdivided into 11 distinct subclusters (Supplementary Fig. S3A), consistent with previously identified heterogeneity [17, 22]. We also identified well-known tissue sub-populations, including epidermal progenitors, dorsal and ventral epidermal populations, and subpopulations of goblet cells and phagocytes within the intestine (Supplementary Fig. S3B). Furthermore, we discovered a new

spatially localized domain, Clu.31, within the blastema. Initially emerging in both the head and tail blastemas at 36 hours post-injury, this domain eventually became restricted to the head by the end of regeneration (Supplementary Fig. S3A), which we designated as the Anterior Regenerative Zone (ARZ).

For better tissue contour characterization, we generated tissue meshes for the intestines, pharynx, and neuronal regions. The spatial distributions of these clusters and tissue meshes were reproducible across two animals at each time point (Fig. 1B and Supplementary Fig. S1B), confirming the robustness of our data at the organismal level. Together, this 4D atlas provides a valuable resource for studying the temporal and spatial dynamics of gene expression and cellular coordination during whole-body regeneration.

Capturing tissue and organ remodeling and identifying genes responsive to regeneration

The comprehensive 3D reconstruction enabled precise measurements of tissue volume changes through the 4D dataset. Analyzing the length ratios along the D/V, M/L, and A/P axes revealed that the D/V and M/L axes shortened, while the A/P axis elongated during regeneration (Fig. 1C, top). Although the cell counts decreased, the average cell volume remained largely unchanged, suggesting that the observed volume changes were primarily due to a reduction in cell numbers (Fig. 1C, bottom). This finding aligns with the body-wide plasticity previously observed in planarians [30].

Using the 4D dataset, we tracked the regeneration of the pharynx, as well as the remodeling of the nervous system (Fig. 1D), with validation through fluorescent in situ hybridization (FISH) for pharyngeal (*foxA*) and neural markers (*pc2*) (Fig. 1E). Both the pharynx and central nervous system, particularly the cephalic ganglia, exhibited increased volume over time, while the intestine

showed a decrease in size but underwent remodeling. The pharynx began to form between 3 and 5 dpa, while the central nervous system matured by 5 dpa (Fig. 1, D and E), reflecting the gradual remodeling in these organs.

To investigate the dynamic cellular responses during regeneration, we analyzed the proportions of different domains over time. We classified cell cluster dynamics into five patterns: continuous increase, initial increase followed by decrease, continuous decrease, initial decrease followed by increase, and unchanged (Supplementary Fig. S2C). Notably, dorsal epidermal progenitors (Epi.30), neural progenitors (Neu.28), and pharyngeal lineages (Pha.19 and Pha.29) exhibited a gradual increase (Supplementary Fig. S3, C–D), reflecting the expansion of these specific lineages required for tissue reconstruction. These results suggest that planarians may balance cell and organ proportions during regeneration, dynamically rescaling body proportions and restoring axial polarity.

Due to the fragility of blastema tissue, conventional FISH methods are challenging for capturing internal gene expression and spatial distribution in this region [31]. To overcome this limitation, we hypothesized that digital segmentation of the blastema region, based on pigmentation intensity, would provide a more effective means of analyzing gene expression and cellular composition. Using image recognition algorithms, we segmented the animals into head blastema, tail blastema, and pre-existing trunk regions (Supplementary Fig. S3E). Statistical analysis revealed positional heterogeneity in cellular responses. For instance, the pharynx (Pha.19) was initially localized to the tail blastema and later to the trunk, suggesting its centripetal migration toward the body center. Additionally, the ARZ (Clu.31) was induced in both blastemas but persisted only in the head region (Supplementary Fig. S3F). This segmentation highlights the potential of our virtual 4D data in identifying distinct cell subtypes that emerge at various stages

and locations during regeneration.

To further investigate the molecular responses in finer regions, we divided the regenerating head and tail blastemas into three subdomains, proximal, middle, and distal, along with the trunk, and identified region-specific gene expression patterns through clustering (Fig. 1, F and G). Both the head and tail blastemas exhibited similar wound-healing and remodeling gene expression, with enrichment in genes associated with Wnt and BMP signaling pathways. However, different subdomains within these regions showed distinct gene expression profiles (Fig. 1H, Supplementary Table S2). Additionally, comparison of differentially expressed genes within the same clusters or regions across various regeneration time points revealed temporal variations in gene expression patterns. For example, we observed that parenchymal domains and epidermal progenitors responded to injury within the first 12 hours post-amputation (hpa), while goblet cells and *cathepsin*$^{+}$ cells were activated between 12 hpa and 3 dpa. Neuronal cells and the ARZ (Clu.31) domain showed a response after 3 dpa (Fig. 1I, Supplementary Table S2), highlighting distinct cellular and regional responses at different stages of regeneration.

In summary, the 4D atlas offers an in-depth view of tissue remodeling, dynamic changes in cellular localization, and the spatial distribution of cell populations throughout planarian regeneration.

Spatiotemporal dynamics of positional gradients during whole-body regeneration

In an accompanying study, we characterized gene expression patterns along body axes and identified genes with regional expression patterns in 3D intact planarians, which we proposed as spatially biased genes (SBGs) [28] (Supplementary Table S3). Some of these genes, involved in patterning processes, were classified as positional control genes (PCGs) [10] (Supplementary

Table S4). To investigate the dynamics of these SBGs during regeneration, we employed our 4D dataset to map gene expression across the entire organism. We hypothesized that injury would disrupt SBG expression, with recovery occurring gradually as regeneration progressed.

To test this, we analyzed the spatiotemporal patterns of SBGs by mapping their expression dynamics onto 16 canonical spatial clusters defined by normalized body length (100 bins) along the A/P axis using logistic regression (Fig. 2A, Supplementary Table S3). At 0 hpa, following injury, the physical loss of head and tail territories led to the disruption of anterior and posterior-specific clusters, whereas medial patterns remained stable. By 5 dpa, the overall gradient patterns had largely been restored to resemble those of uninjured individuals (Fig. 2A), highlighting the dynamic process of positional remodeling during regeneration. As regeneration proceeded, these spatial domains were progressively reconstructed, with gene expression patterns returning to their homeostatic states by 14 dpa. The complete gene composition for each spatial cluster is documented in Supplementary Table S3.

Leveraging the quantitative nature of spatial transcriptomics, we further explored the spatiotemporal dynamics of SBGs. Principal component analysis (PCA) on known PCGs along the body axes of intact planarians revealed that genes expressed in the same regions clustered together (Supplementary Table S4). For example, *ndl-4* and *sfrp1* were specifically expressed in the head (Supplementary Fig. S4A), confirming their region-specific patterns [32, 33]. This analysis also allowed us to quantify relative expression patterns, such as *ndk* in both the head and pharynx, and *wntA* in the pharynx (Supplementary Fig. S4B), consistent with established spatial distributions [14, 34-36].

Next, we mapped SBGs across multiple time points in PCA space to track their dynamic recovery during regeneration. Notably, several known PCGs, including *ndk*, *fz5/8-4*, *fz4-1*, and

wnt11-2 [14, 34], exhibited a reciprocal recovery pattern along the A/P axis. These genes initially showed higher expression compared to uninjured individuals, before gradually returning to baseline levels (Fig. 2B, bottom). Our analysis revealed that following the disruption caused by amputation, SBG expression did not simply ramp up or down linearly. Instead, these genes exhibited transient dynamic fluctuations along the body axis. We modeled this recovery as a perturbation in a dynamic system, analogous to an underdamped mass-spring system (Fig. 2B, top), where the system is displaced from equilibrium (homeostasis) by amputation and subsequently driven back by a restoring force representing the gene regulatory network (GRN) [37]. This dynamic trajectory eventually restored the gene expression profile to its homeostatic state by 14 dpa (Fig. 2A). The PCA (Fig. 2B) further delineates this process into distinct biological phases. The early time points (0–36 hpa) cluster separately from later stages, representing an "Acute Injury Phase" characterized by a distinct wound response program that drives the system far from equilibrium. This is followed by a "Patterning Phase" (3–10 dpa), where the trajectory shifts direction and converges toward the homeostatic attractor [38]. This observation is consistent with the distinct, transient injury states recently identified in single-cell studies [17], confirming that early wound responses are transcriptionally distinct from the later morphogenetic programs that restore body plan fidelity. We hypothesize that injury disrupts regional expression, and that the gene regulatory network may act as a restoring force, guiding the recovery of disrupted PCG expression through feedback mechanisms (Fig. 2B, top). Consistent with this model, our analysis showed that PCG expression was elevated between 3 and 10 dpa, and returned to baseline by 14 dpa upon completion of regeneration (Supplementary Fig. S4C), further supporting this recovery model.

To visualize the dynamic recovery of spatial patterns, we color-coded the expression patterns

of known PCGs at each time point (Methods). These patterns were restored by 12 hpa, 36 hpa, or 3 dpa (Fig. 2C), suggesting a temporal progression in their recovery. Notably, spatial patterns were restored earlier than corresponding detectable changes in gene expression. For instance, the spatial pattern of *ndk* was restored by 12 hpa (Fig. 2C), while its expression began to increase only at 36 hpa (Supplementary Fig. S4C and Supplementary Table S3). These temporal dynamics suggest that spatial patterning may influence the regulation of gene expression.

Motivated by the underdamped response of PCG trajectories (Fig. 2B) and the temporal hierarchy between spatial pattern recovery and gene expression (Fig. 2C), we hypothesized that SBG expression dynamics could be modeled mathematically. Extending the self-organizing model proposed for the Wnt pathway along the A/P axis [32], we applied the Gierer-Meinhardt model of a simple activator-inhibitor system [39] within the Turing system framework [40] to predict global changes in SBG expression during regeneration. While the model traditionally describes the diffusion and reaction kinetics of secreted morphogens, such as the *Wnt/Notum* pair, we applied it here to characterize the expression of downstream TFs, such as *hox4b* and *EGR1*. We propose a Readout hypothesis where these nuclear factors do not diffuse themselves but act as high-resolution spatial proxies that interpret the primary, diffusive morphogen gradients. By analyzing spatial gradients in exponential form and excluding the influence of the pharynx-enriched genes, we separated independent temporal and spatial components to simulate changes in activator and inhibitor expression at each time point during regeneration (Fig. 2D, top). Our findings demonstrate that the predictions of trajectories of these “readout” genes, such as *ARNT* [41], *Ndk* [14], *EGR1*, *hox4b*, *Nfya*, and *glra1* (Fig. 2D, bottom, Supplementary Table S4) closely follow theoretical activator-inhibitor kinetics, reinforcing the conclusion that planarian regeneration is guided by scalable, self-organizing patterning systems.

We next explored whether SBGs were enriched in specific domains or regions. By quantifying the number of SBGs enriched in each SPC at different stages of regeneration, we observed that SBGs were expressed across multiple lineages, including muscle, epidermal, and neural lineages (Fig. 2E). Interestingly, the ARZ domain (Clu.31) displayed characteristics from several lineages and contained the highest number of SBGs (Fig. 2E). This observation suggests that the ARZ may play a role in remodeling and maintaining polarity. Overall, our 4D analysis provides a detailed view of the spatiotemporal dynamics of SBG expression during regeneration, offering support for a model based on self-organized reaction-diffusion patterns.

Characteristics of the injury-induced Anterior Regenerative Zone (ARZ) enriched in SBGs

Having demonstrated that the ARZ (Clu.31) exhibits injury-induced anterior localization and is enriched in SBGs, we hypothesized that the ARZ plays a crucial role in regulating PCGs and maintaining regenerative polarity during regeneration, similar to the proposed function of the organizer [42]. We further sought to characterize this region. At homeostasis, the ARZ is localized to the anterior side, with signatures of three distinct lineages (Fig. 3A and Supplementary Table S5). Gene expression analysis within this domain revealed enriched expression of SMED30003831 (*smed03831 or Rod1*), *caveolin3*, and SMED30001640 (*smed01640)* (Supplementary Fig. S5A and Supplementary Table S2). Notably, the ARZ spans both the peripheral epidermal and subepidermal areas of the head, distinguishing it from the *Equinox*-expressing wound epidermis [43] (Supplementary Fig. S5A). Gene ontology (GO) analysis of ARZ-enriched genes identified processes related to epidermal differentiation, muscle contraction, and neural development (Supplementary Fig. S5B). Co-FISH experiments with the ARZ marker *smed03831* and lineage markers confirmed that the ARZ encompasses epidermal (*agat-1*) (Fig.

3B), muscular (*collagen*) (Fig. 3C), and neural (*pds*) cells (Fig. 3D), solidifying its tri-lineage characteristics. These findings suggest that the ARZ is a co-regulated, regeneration-responsive region.

To track the temporal dynamics of the ARZ, we analyzed its spatial location throughout regeneration. At 36 hpa, the ARZ was present as scattered clusters near the ventral wound sites in both head and tail fragments. By 3–5 dpa, these cells converged towards the midline and expanded to cover the wound area, coinciding with wound closure and blastema formation. By 10 dpa, ARZ cells diminished in the tail but persisted in the head region (Fig. 3E, Supplementary Fig. S5C). Measurements of the distance from the wound surface revealed that the ARZ gradually approached the amputation site during the first five days, with an increase in cell number within the zone (Fig. 3F). This was further confirmed by staining for the ARZ marker *smed03831* in the regenerating head region (Fig. 3G).

To investigate the putative origin of ARZ cells, we traced their pseudotime trajectory during regeneration using Monocle [44]. This analysis revealed three distinct branches (Fig. 3H), each enriched for genes specific to epidermal, muscle, or neuronal lineages (Fig. 3I, Supplementary Fig. S5D). The earliest reappearance of epidermal signatures at 12 hpa marked the emergence of the ARZ. To validate this injury-induced differentiation dynamics, we profiled the temporal expression of the late epidermal progenitor marker *agat-1* within the ARZ. We observed a progressive enrichment of *agat-1* starting from 36 hpa and peaking at 3 dpa (Supplementary Fig. S5E), mirroring the kinetics of active blastema differentiation rather than static tissue maintenance [17, 45]. This was followed by the emergence of muscle and neuronal markers at 3 dpa, coinciding with blastema formation (Fig. 3J). By 14 dpa, the ARZ cellular composition had largely reverted to epidermal cells, resembling the homeostatic state (Fig. 3J). The expression of ARZ-enriched

genes aligns with these cellular dynamics (Supplementary Table S5), further supporting the coordinated and timely assembly of the ARZ domain during regeneration.

Cellular composition and regulation of the polarity-enriched ARZ domain

Having demonstrated the coordination between ARZ formation and regeneration, we next sought to investigate the cellular components that control ARZ formation. Our focus was on the epidermis, as it constitutes the earliest cell type to emerge within the ARZ. To identify the regulatory factors involved in ARZ formation, we employed RNA velocity, a method that distinguishes between unspliced and spliced mRNAs [46], to predict the putative trajectory of the SPC clusters in epidermal lineages (Supplementary Fig. S6A). The velocity vectors indicated that epidermal cells in the ARZ primarily originate from the ventral epidermal lineage (Epi.1) (Supplementary Fig. S6, A and B). To visualize ARZ formation in a spatiotemporal context, we projected 3D spatial data onto 2D maps of the head blastema at various time points using via unwrapping (Fig. 4A), a dimensionality reduction technique transforming a 3D model's surface into a 2D plane (see Methods). Pseudotime trajectory analysis of the unwrapped 2D map revealed a cell-state transition from ventral epidermal cells toward the ARZ, while left and right dorsal-ventral boundary cells on both sides of the head blastema moved toward the anterior pole (Fig. 4, B to D). These observations suggest that ARZ formation involves interactions between ventral and dorsal epidermal cells. Indeed, comparing spatial maps at 36 hpa and 3 dpa revealed the expansion of the ARZ domain from both ventral and dorsal sides, coinciding with wound closure (Fig. 4E).

To explore the role of the ARZ as a signaling-rich domain during regeneration, we conducted trajectory analysis to identify potential regulators involved in cell differentiation within the ARZ (Fig. 4F). While genes such as *smed03831* and *caveolin3* serve as definitive markers of the

differentiated ARZ, we sought to identify the upstream drivers governing its formation. Notably, the mediator complex subunit 8 (*med8*) emerged as an early-expressed gene along the pseudotime trajectory (Fig. 4F), preceding the expression of structural markers. Med8 is an essential component of the mediator complex, playing a critical role in transcription regulation [47]. The planarian *med8* homolog is evolutionarily conserved and shares high sequence identity with orthologs in other species (Supplementary Fig. S6C). In the homeostatic state, *med8* is highly expressed in neoblasts (Supplementary Fig. S6D), with enrichment observed across multiple neoblast subpopulations (Supplementary Fig. S6E-F). Following injury, *med8* expression increased in the wound area at 12 hpa, prior to the emergence of *smed03831*$^{+}$ cells at 1.5 dpa (Fig. 4G). This suggests that *med8* may regulate ARZ formation. The co-expression of *med8* and *smed03831* in a substantial portion of blastema cells after 1.5 dpa further supports its regulatory role in ARZ formation (Fig. 4, G and H). To assess the functional role of *med8*, we performed RNA interference (RNAi) knockdown experiments (Supplementary Fig. S6G) and measured the expression of the ARZ marker *smed03831*. Knockdown of *med8* resulted in impaired blastema regeneration by 5dpa (Fig. 4I) and a significant reduction in the number of *smed03831*$^{+}$ cells at 3 and 5 dpa (Fig. 4J), suggesting that *med8* is required for ARZ formation.

Having shown that *med8*(RNAi) hinders ARZ reconstruction (Fig. 4J), we next investigated the specific stage at which *med8* influences ARZ formation by examining gene expression at different regeneration time points. Given that the ARZ is enriched with various PCGs (Fig. 2E), we hypothesized that the failure in ARZ reconstruction would prevent the re-establishment of anterior polarity. To test this, we examined the expression of the anterior pole marker *sfrp-1* [13, 48], which was significantly reduced upon *med8* knockdown (Fig. 4J), suggesting a failure to generate the anterior pole identity during regeneration. In control animals, *smed03831*$^{+}$ cells were

enriched at the wound site at 1.5 dpa and fully covered the wound, followed by the appearance of *sfrp-1*$^+$ cells at the anterior pole by 3 dpa (Fig. 4J). This temporal sequence suggests that ARZ formation precedes anterior pole formation. In contrast, *med8*(RNAi) animals exhibited impaired ARZ formation and a reduction in *sfrp-1*$^+$ cells (Fig. 4J), indicating an inability to re-establish anterior identity. By 7 dpa, ARZ formation was completely disrupted in *med8*(RNAi) animals, and regeneration failed (Fig. 4K), linking ARZ formation to successful regeneration. Together, these findings support the idea that *med8*-mediated ARZ formation is essential for providing the cellular basis for pole formation during regeneration.

***Med8* is required for ARZ lineage specification to support blastema development**

To identify the transcriptional programs mediating changes in the ARZ, we conducted scRNA-seq on *med8* and control RNAi animals with amputated tails undergoing head regeneration. By integrating the data from both groups, we identified known cell lineages, including stem cell populations and eight distinct domains (Fig. 5A), consistent with previous findings [8, 22]. Notably, *med8* RNAi expanded the neoblast population while reducing the proportions of ARZ-associated lineages, specifically neural, muscle, and epidermal cells (Fig. 5B). This observation was further confirmed by pseudotime trajectory analysis using Monocle, which revealed similar reductions in the differentiation of these cell lineages (Supplementary Fig. S7A). Label transfer analysis matching ARZ cells across the scRNA-seq dataset confirmed a decrease in each ARZ cellular component in *med8* RNAi animals (Fig. 5C and Supplementary Fig. S7B).

Given that the ARZ is enriched for genes involved in polarity formation, we next examined whether the differentiation blockade caused by *med8* RNAi led to a loss of cells expressing anterior and posterior pole markers. Analysis of A/P axis marker gene expression in the scRNA-seq data

revealed that *med8* RNAi led to a reduction in the polarity signature score within ARZ cells, but not in other domains (Fig. 5D and Supplementary Fig. S7C). Further analysis showed a decrease in both anterior and posterior markers (Fig. 5E). Whole-mount in situ hybridization (WISH) confirmed reduced expression of posterior markers, including *wnt1* and *Wnt11-1 [36]*, though body-wide polarity was not completely disrupted in *med8* RNAi animals (Fig. 5, F and G), suggesting that the observed changes are linked to regenerative growth. These results indicate that *med8* RNAi impairs ARZ formation, a prerequisite for establishing positional landmarks within the blastema during regeneration.

To investigate how *med8* affects ARZ formation, we examined the expression of ARZ markers within the blastema. The spatial extent of major domains in the ARZ, including epidermal (*agat-1)* and muscle (*collagen*) cells, was reduced in *med8* RNAi animals (Fig. 5H). Additionally, the expression of transcription factors essential for the differentiation of neural (*tcf/lef-1*) [49], epidermal (*p53*) [50], and muscle (*dmrt2*) cells was diminished upon *med8* knockdown (Supplementary Fig. S7D), indicating that *med8* is crucial for maintaining the transcriptional programs associated with ARZ cell fate [51]. In line with this, gene expression analysis in neoblasts revealed downregulation of pathways related to stem cell division as well as neural and epidermal fate determination (Supplementary Fig. S7E). To quantify this defect at the cellular level, we calculated the differentiation efficiency across lineages in our scRNA-seq dataset. We found that despite an accumulation of undifferentiated neoblasts, the efficiency of generating lineage-committed progenitors was severely compromised for the neural, muscle, and epidermal domains (Supplementary Fig. S7F), confirming a broad differentiation blockade. Furthermore, FISH staining at 3 dpa and 5 dpa demonstrated reduced expression of markers for epidermal and muscle cells in the blastema region (Fig. 5, H and I), suggesting impaired cell fate decisions in ARZ cells.

We also observed decreased expression of neural markers in *med8* RNAi animals (Fig. 5J), further confirming the disruption of ARZ lineage specification and impaired head regeneration.

Finally, we assessed whether *med8*-mediated ARZ formation is required for homeostasis. While short-term *med8* RNAi treatment caused minimal phenotypic changes in homeostatic animals, prolonged *med8* knockdown led to head regression (Supplementary Fig. S7G), suggesting that sustained loss of ARZ function results in homeostatic defects. In summary, our data support the role of *med8* in controlling blastema growth by enabling the specification of the ARZ-associated lineages, which is essential for proper tissue regeneration and the maintenance of homeostasis.

Discussion

Understanding the full spectrum of spatial information and the principles governing pattern formation during regeneration in tissues and organs remains a significant challenge. This complexity is driven by the intricate tissue geometry, the large size of multicellular organisms, and the limited number of model organisms capable of regenerating entire tissues. Additionally, the absence of techniques capable of capturing high-resolution spatial transcriptomic data across an entire organism in 3D over time further complicates this challenge. In this study, we applied high-resolution Stereo-seq (715 nm) to planarians to reconstruct the 4D spatiotemporal landscape of whole-body regeneration. Our 4D dataset addresses several limitations of traditional techniques, including low-throughput FISH assays and 2D single-slice-level spatial transcriptomics, providing a holistic and high-resolution view of genes and spatial domains before and during regeneration. While recent spatial transcriptomics methods provide spatial context [24, 25, 52], they often lack full 3D or single-cell resolution, limiting the ability to comprehensively profile morphogen gradients and domains across entire organisms over time. In contrast, our 4D regeneration atlas

offers a high-resolution, time-resolved framework for analyzing regenerative dynamics. Mining our dataset allowed for the recovery of single-cell transcriptomes at the spatiotemporal level, enabling the visualization of gene expression patterns, positional signals, and cell type distributions across multiple scales. This analysis revealed morphogen gradient gene dynamics, identified regenerative domains, and highlighted key regulatory factors. The full dataset is available through our online resource: https://db.cngb.org/stomics/prista4d.

By leveraging the complete repertoire of SBGs across four dimensions, we examined gene expression across anatomical regions and scales, particularly within the delicate blastema region, allowing for quantitative analyses across multiple body regions. We confirmed known PCGs and identified potential new PCG candidates, facilitating the modeling of morphogenetic gradients using the Turing reaction-diffusion model. While the Turing system has been proposed for fission and regeneration [32, 40, 53], its applicability to modeling planarian regeneration remains unclear [54]. The temporal dynamics of SBGs suggest that the restoration of positional identity functions as a self-organizing system. Rather than simple oscillations, the recovery follows an underdamped trajectory where the initial injury response (0–36 hpa) creates a significant displacement from the transcriptomic baseline, followed by a directed convergence during the patterning phase (3–10 dpa). This “overshoot and return” dynamic supports a model where the GRN acts as a restoring force, progressively dampening the perturbation introduced by amputation until the homeostatic equilibrium is regained. Our real dataset supports simulation predictions, with specific genes exhibiting consistency between modeled and observed data. This rich dataset and quantitative approach provide a foundation for studying scalable, self-organized pattern formation in more detail, offering a framework for understanding how positional information is re-established during regeneration. Integrating high-resolution spatial transcriptomics with single-cell analysis, our

study offers a valuable resource for investigating how positional information is maintained, disrupted, and interpreted across different tissues and domains following injury. This dataset bridges the gap between molecular, cellular, and morphological aspects of regeneration, offering a comprehensive multimodal view of whole-body regeneration dynamics in organisms. This supports a hierarchical model where global self-organizing gradients are interpreted by local gene regulatory networks to restore tissue identity. These findings align with the perspective that regeneration relies on the re-establishment of organizers that coordinate tissue repair through these dynamic molecular gradients [42, 55].

Muscle cells have long been recognized as the primary conveyors of positional cues in adult planarians [16]. However, our 4D dataset expands this view, identifying non-muscular lineages, such as neuronal and epidermal cells, as contributors to the regenerative positional landscape. This suggests that pattern remodeling involves a multi-lineage process, with multiple cell types participating in encoding, reading, and interpreting positional information [56, 57]. We comprehensively profiled SBGs, which extend beyond PCGs, to define a systemic patterning system involving genome-wide spatial regulation. Since regenerative patterning involves both global polarity and local fate specification, most SBGs may not show defects in polarity but could alter cell fate [33, 58, 59]. The temporal dynamics of different SBG classes suggest that encoding and interpreting positional gradients is a distributed and hierarchical process, coordinated among various domains and exhibiting self-organizing properties within the organism.

The distinct temporal lag between the restoration of spatial patterning such as *ndk* asymmetry at 12 hpa and the subsequent transcriptional surge at 36 hpa implies a hierarchical “Pattern-then-Amplify” regulatory logic. We propose that the immediate post-injury phase involves unmasking of positional identity within pre-existing tissues. As muscle cells serve as the primary source of

positional instructions, surviving tissue at the wound boundary utilizes stable landmarks to rapidly reset the anatomical coordinate system [16]. This process is likely orchestrated by early signaling modulators, such as *Ptpn11* that fine-tune wound response pathways and *Wnt* dynamics prior to significant transcriptional activation [60]. Consequently, a precise spatial blueprint is established at low transcriptional levels by 12 hpa to spatially constrain the subsequent proliferative burst. The later peak in expression at 36 hpa thus reflects the amplification of this established pattern to support the massive cellular demands of blastema formation, effectively decoupling the definition of “where to regenerate” from the execution of “how much to grow” [61].

Analysis of SBG distribution revealed a specific spatiotemporal domain, Clu.31, which we define as the ARZ. While this domain contains mechanosensory neurons and epithelial cells characteristic of the anterior peripheral nervous system, our data suggest it represents a dynamic cellular neighborhood [17, 52] where the intercalating peripheral nervous system integrates with the dorsal-ventral boundary epithelium and body wall muscle to orchestrate regeneration. Specifically, the transient appearance of this domain in the tail blastema (Fig. 3E) and its dynamic compositional shifts, transitioning from an initial injury-induced epidermal state to a multi-lineage hub before resolving (Fig. 3H-J), distinguish it from a static anatomical structure. The temporal dynamics of *agat-1* expression within this domain further mirror transient regeneration-activated cell states (TRACS) described in other contexts [17, 45], supporting the hypothesis that the ARZ functions as a transient regenerative organizing center rather than solely representing the regenerating nervous system. From an evolutionary perspective, the presence of this spatiotemporally coordinated domain is functionally reminiscent of the apical epithelial cap (AEC) observed in vertebrate appendage regeneration [62]. Although anatomical differences exist, particularly the integral role of neural components in the planarian ARZ, the principle of a transient

signaling niche established by the convergence of epithelial and sub-epithelial tissues [63, 64] appears conserved. Similar to the AEC, the ARZ exhibits conserved molecular marker gene expression involving the Wnt/β-catenin and FGF pathways [13, 14, 48]. Further comparative analyses will be required to determine if the specific molecular circuits governing these cellular neighborhoods are homologous across metazoans [42].

Our findings using *med8*(RNAi) as a proxy suggest that epidermal, muscular, and neural cells within the ARZ likely contribute to positional information for blastema induction. The persistence of this domain in adult planarians may help explain their homeostatic maintenance, providing insights into the regulation of regeneration [42, 61]. We further demonstrate that med8, a subunit of the Mediator complex acting as a bridge between transcription factors and RNA polymerase II [47], modulates key genes related to epidermal, muscle, and neural specification within the regenerative domain. The Mediator complex is known for its role in maintaining stem cells, as well as in lineage-specific differentiation [51]. While the loss of Smed-*med14* specifically affects stem cell populations, the loss of *med8* does not [65], suggesting that distinct Mediator components have different requirements for stem cell function in planarians. Our findings build upon previous studies and demonstrate that *med8* is important for the differentiation of stem cells into neural, muscle, and epidermal lineages within the ARZ region, thereby ensuring the production of proper regenerative patterning signals. This suggests that Mediator, in conjunction with transcription factors [66-71], may be involved in establishing the epigenetic landscape necessary for lineage commitment and cell fate transitions.

The observation that *med8* knockdown markedly reduces the expression of polarity markers without causing gross disruption of the whole-body AP axis (e.g., double-head formation) warrants further discussion. Our single-cell and lineage analyses suggest that this discrepancy likely stems

from the hierarchical role of *med8* in cell fate specification rather than direct gradient scaling. Since *med8* is required for the differentiation of specific ARZ lineages (epidermal, muscle, and neural), the observed reduction in polarity markers (e.g., *sfrp-1*, *wnt1*) reflects a loss of the signal-producing cells themselves, rather than a simple downregulation of gene expression within an intact tissue. Furthermore, *med8* knockdown leads to a symmetric reduction in both anterior (*sfrp-1*) and posterior (*wnt1*, *wnt11-1*) signals. This balanced reduction likely preserves the relative antagonism between the anterior and posterior poles, preventing the dominance of one pole over the other that typically drives ectopic structure formation. Finally, while gross morphology is maintained in the short term, we noted that prolonged *med8* RNAi eventually leads to head regression, confirming that *med8*-mediated cellular turnover is indeed essential for the long-term maintenance of cell populations that harbor positional landmarks of the global body axes.

Potential implications

Despite the advantages of our 4D approach, specific limitations remain. First, the sequencing depth is lower compared to scRNA-seq, which may impact the detection of rare cell populations. Second, biological replicates are limited due to the technical challenges of generating whole-organism 4D data. Additional replicates and validation will be required to further assess the robustness of morphogenetic gradients across individuals. Future efforts should focus on increasing sequencing depth, expanding the number of biological replicates, and incorporating complementary approaches to validate the dynamics of gene regulation. Nevertheless, integrating these data with gene perturbation and longitudinal imaging studies will enable us to directly assess the functional contributions of specific positional signals in guiding pattern remodeling and regeneration.

Our study establishes a framework for a 4D high-resolution atlas of gene expression dynamics throughout whole-body regeneration. By combining spatial and temporal transcriptomic

data, we provide a novel framework for understanding the principles governing regenerative patterning, advancing both regenerative biology and spatial transcriptomics methodologies. This comprehensive dataset serves as a valuable resource for future studies, enabling researchers to explore positional information, tissue remodeling, and the regulation of regeneration in biological systems.

Methods

Animal culture

Asexual *Schmidtea mediterranea* (strain CIW4) were maintained at 20 °C in a recirculating 1× Montjuic salts solution without antibiotics, following a previously described protocol [72]. The animals were routinely fed beef liver. For experimental procedures, the animals were transferred to static culture and starved for at least 7 days.

Gene cloning and RNAi feeding

Genes of interest were cloned from a CIW4 cDNA library into the pPR-T4P vector as previously described [73]. The resulting plasmids were used to produce dsRNA for RNA interference (RNAi). RNAi was performed following established protocols for gene knockdown [74]. Briefly, bacterial pellets expressing the dsRNA were mixed with fresh beef liver paste in a 4:1 ratio. EGFP dsRNA was used as a control. Animals were fed every 3 days for a total of four or six RNAi feedings. After the final RNAi feeding, animals were amputated 3 days later to collect samples at various stages of regeneration.

In situ hybridizations

Whole-mount *in situ* hybridizations were conducted following previously established protocols

[31, 75]. In short, the mucus from the animals was removed using 5% NAC in PBS, and then fixed for 1 hour in 4% formaldehyde (FA) in PBSTx (0.5%). The animals were bleached with formamide and incubated with proteinase K (2 μg/mL, AM2546, ThermoFisher) for 10 minutes. After a 2-hour pre-hybridization step, the hybridization was performed at 56 °C for over 16 hours. Following extensive washes, the antibody signal was amplified using the Tyramide Signal Amplification system. Tissue clearing was achieved using ScaleA2 to reduce background noise [31].

Sample fixation and section preparation for Stereo-seq

Sample fixation was carried out using a modified version of a previously described protocol [76]. In short, animals were relaxed in 0.66 M $MgCl_2$ for 1 minute, followed by fixation in Methacarn solution (6 mL methanol, 3 mL chloroform, 1 mL glacial acetic acid) for 10 minutes. After fixation, the animals were rinsed in methanol three times, rehydrated in 50% methanol in PBS for 5 minutes, and then cryoprotected in 20% sucrose in PBS for two cycles. The cryoprotected tissues were embedded in pre-cooled OCT, frozen with dry ice, and stored at -80 °C until cryosectioning. Following embedding, the specimens were photographed under a stereomicroscope to acquire brightfield microscopy images. These images documented the morphological features and macroscopic pigmentation patterns of each specific animal, serving as the anatomical reference for downstream spatial region segmentation and data alignment. Tissues were equilibrated in a -20 °C freezing microtome for 30 minutes prior to sectioning. RNA quality from cryosections was assessed using an Agilent 2100 Bioanalyzer. The cryosections of *Schmidtea mediterranea* were cut serially at 10 μm intervals using a Leica CM1950 cryostat. Each section was placed onto a Stereo-seq chip, incubated for 3 minutes at 37 °C on a Thermocycler Adaptor, and then fixed in methanol at -20 °C for 40 minutes.

ssDNA staining and imaging of Stereo-seq slides

Prior to tissue permeabilization, sections on the Stereo-seq chip were stained with a nucleic acid dye (Thermo Fisher, Q10212) to visualize single-stranded DNA (ssDNA). The stained sections were then imaged using a Leica DM6M microscope. The images were stitched together and processed using the Leica Application Suite X software.

Library construction and sequencing of Stereo-seq data

The library construction and sequencing protocols for Stereo-seq have been previously described [27]. In summary, tissue sections were first washed with 100 μL of 0.1× saline-sodium citrate buffer (SSC, Thermo, AM9770) containing 0.05 U/μL RNase inhibitor (NEB, M0314L) to remove any remaining staining solution. Sections were then permeabilized using 0.1% pepsin (Sigma, P7000) in 0.01 M HCl buffer (pH 2.0) and incubated at 37 °C for 18 minutes. Released mRNAs were captured on the Stereo-seq chip and reverse transcribed overnight at 42 °C using SuperScript II reverse transcription mix (Invitrogen, 18064-014), containing 10 U/μL reverse transcriptase, 1 mM dNTPs, 1 M betaine solution, 7.5 mM $MgCl_2$, 5 mM DTT, 2 U/μL RNase inhibitor, 2.5 μM Stereo-seq template switch oligo, and 1× First-Strand buffer.

After *in situ* reverse transcription (RT), tissue sections were treated with a removal buffer (10 mM Tris-HCl, 25 mM EDTA, 100 mM NaCl, 0.5% SDS) at 37 °C for 30 minutes. The remaining RT products were then collected and amplified using KAPA HiFi Hotstart ReadyMix (Roche, KK2602) and 0.8 μM cDNA-PCR primers. PCR products were used to prepare sequencing libraries, with the following steps: quantification of concentration using the Qubit™ dsDNA Assay Kit (Thermo, Q32854), DNA fragmentation with in-house Tn5 transposase at 55 °C for 10 minutes,

PCR amplification (KAPA HiFi Hotstart ReadyMix, Roche, KK2602) with 0.8 μM cDNA-PCR primers, and purification using Vazyme (N411-03). The purified PCR products were used to construct DNB libraries and sequenced on an MGI DNBSEQ-T1 sequencer (35 bp for Read1, 100 bp for Read2). The sequencing data were processed to generate a quantified spatial gene expression matrix at the subcellular level.

Spatial transcriptomics data processing

Spatially resolved single-cell RNA-seq data obtained through Stereo-seq were pre-processed for further analysis. The first read (Read1) of the sequencing library contained coordinate identifiers (CIDs), molecular identifiers (MIDs), and poly-T sequences, while the second read (Read2) provided the captured cDNA sequences. Spatial x-y coordinates of cDNA at 715 nm resolution were determined based on the CID sequences with a 1-bp mismatch tolerance. cDNA sequences were aligned to the *S. mediterranea* genome (dd_Smes_G4), and only mapped reads were used to identify exon transcripts. The MID sequences served to provide unique molecular identifiers (UMIs) for transcript quantification, with PCR duplicates removed using handleBam (https://github.com/BGIResearch/handleBam). Read pairs with a MID quality score below 10 were excluded. Finally, gene expression matrices incorporating spatial information were generated using quality-controlled exonic data.

3D reconstruction, clustering and cell type annotation of regenerating animals

Regenerating planarians were reconstructed using methods outlined in an accompanying manuscript, where we developed a 3D spatial transcriptomics framework [28]. First, the MIRROR algorithm was applied to align the spatial gene expression heatmap with the ssDNA staining image.

Cell segmentation was then performed utilizing using CellProfiler and Fiji. Gene expression data were mapped to each individual cell, creating a spatial transcriptome map at single-cell resolution. Next, after performing dimensionality reduction and clustering, cell clusters were annotated based on known lineage markers. The SEAM algorithm was employed to align the sections along the z-axis, thus determining the x-y-z coordinates of each cell. Morphological distortions induced by experimental procedures were corrected based on established polarity gene patterns, and the 3D reconstructions were created using a combination of 3DSlicer and MeshLab. Finally, SPC cells from different stages of regeneration were integrated using the FindIntegration and IntegrateData functions in Seurat (v4.0.2). Dimensionality reduction and clustering were then conducted in Seurat following standard procedures. To facilitate a comprehensive understanding of the analytical strategies employed in this study, we provide a schematic overview of the entire computational workflow (Supplementary Fig. S8). This diagram illustrates the sequential processing pipeline, beginning with raw data input (h5ad and imaging files) and preprocessing via the GEM3D toolkit, followed by parallel analytical modules including WACCA for 3D reconstruction, SPC analysis for spatial clustering, and polarity analysis for modeling morphogenetic gradients.

Data quality control and validation

To ensure the technical reliability, sensitivity, and reproducibility of our spatial transcriptomic atlas, we systematically evaluated key quality control (QC) metrics across all biological samples, tissue sections, and annotated spatial domains (Supplementary Table S1). Following single-cell segmentation, we quantified the segmented cell area in pixels, the number of effective spatial spots (nDNB), total unique molecular identifiers (UMIs), and the number of detected genes per cell.

Analysis of all 35 tissue sections demonstrated high technical consistency across the dataset. The median number of detected genes per cell across sections ranged from approximately 150 to 230, with median UMI counts ranging from 190 to 370.

To rule out potential technical biases that might favor highly transcriptionally active cells over cells with lower RNA content, we statistically evaluated the capture efficiency across all 36 identified spatial domains. Our analysis revealed no meaningful systematic bias toward specific lineages. The distributions of cell area, detected genes, and UMI counts remained broadly consistent and biologically appropriate across the diverse cellular populations. Robust transcript detection was achieved globally, ranging from large differentiated lineages to smaller undifferentiated neoblasts and progenitor states. This uniform data quality confirms that our Stereo-seq approach provides sufficient resolution and sensitivity to capture transcription factors and resolve heterogeneous cell states without significant transcript dropout for low-abundance populations.

Furthermore, to validate the quantitative accuracy of our Stereo-seq dataset at the global tissue level, we assessed its concordance with traditional bulk RNA-seq data. Single-cell spatial expression profiles from Stereo-seq sections were aggregated to generate pseudo-bulk transcriptomes. We then calculated the Pearson correlation coefficients between these pseudo-bulk profiles and corresponding bulk RNA-seq datasets of regenerating planarians. The high correlation observed (Figure S1C) verified the technical reproducibility of our platform and confirmed the absence of significant transcript dropout or amplification bias during *in situ* capturing and library preparation.

For the visualization of spatial gene expression patterns in Fig. 2A, a representative sample was selected for each time point from the biological replicates (n=2). This selection was

determined by calculating the mean gene expression vector (centroid) for each time point and identifying the replicate with the highest Pearson correlation to this centroid. All quantitative analyses and statistical tests were performed using the full integrated dataset across all replicates.

Correlation analysis with single-cell and spatial atlases

To characterize the cellular composition of the 36 identified spatial domains, we performed a Pearson correlation analysis comparing the expression profiles of our clusters against annotated cell types from three independent single-cell RNA-seq atlases [22, 23, 77] and spatial domains from a 10x Genomics Visium dataset [24]. This analysis categorized domains into High Fidelity (1-to-1 mapping), Lineage Restricted (mapping to a single tissue class), or Mixed Domains (containing signatures from multiple cell types, such as the neoblast-parenchyma niche).

Blastema region detection in 3D spatial transcriptomics data

The blastema regions were identified based on pigmentation patterns. The boundary between the unpigmented blastema and pigmented trunk was defined using the Threshold function in ImageJ and refined via quadratic polynomial regression. To spatially resolve the interface between pre-existing and newly formed tissue, we defined a border zone extending 20 μm on either side of this regression line. This subdivision created distinct spatial domains at the anterior wound: the Head Anterior Region (HAR), Head Border Outer Region (HBOR), and Head Border Inner Region (HBIR). An analogous approach applied to the posterior wound defined the TPR, TBOR, and TBIR domains. For regional analyses, the three anterior (HAR, HBOR, HBIR) and posterior (TPR, TBOR, TBIR) compartments were collectively designated as the head and tail blastema regions, corresponding to their distal, middle, and proximal subdivisions, respectively. Cells were assigned

to these discrete regions based on their spatial coordinates subsequent to the alignment of the transcriptomic data with the microscopy images utilizing TrakEM2.

Identification of temporally differentially expressed genes

Temporal alterations in gene expression were analyzed by comparing adjacent time points for each defined SPC cluster and anatomical region (e.g., blastema and trunk regions) using the DEsingle algorithm. Specifically, expression profiles corresponding to a specific cluster or region were extracted from the integrated dataset. For each pair of adjacent time points (e.g., 0 hpa vs. 12 hpa, 12 hpa vs. 36 hpa, etc.), we employed the DEsingle R package [78] to detect differentially expressed genes (DEGs). Following the primary analysis, we utilized the DEtype function within the DEsingle package to classify the identified DEGs into distinct categories based on variations in gene expression abundance and distribution. To ensure statistical rigor, raw p-values were adjusted using the Benjamini-Hochberg procedure, and genes with a False Discovery Rate (FDR) < 0.05 were retained as significant DEGs (Fig. 1I, Supplementary Table S2).

Identification of SBGs

To quantitatively examine spatial gene expression patterns, we established a molecular coordinate system by dividing the straightened planarian body into bins along the A/P (100 bins), M/L (40 bins), and D/V (14 bins) axes. SCT-transformed expression values for highly variable genes (HVGs) and known polarity control genes (PCGs) were averaged per bin, normalized by cell density, scaled, and smoothed using a Gaussian filter (sigma=3). Genes expressed in fewer than five consecutive bins were excluded from downstream analysis. We then applied a hierarchical density-based clustering algorithm to the homeostatic dataset to aggregate genes with similar

spatial profiles along the body axes. To refine the clustering, parameters were optimized for spatial distinctness, and unassigned genes were assigned to the most probable groups using linear regression. The resulting clusters and their constituent genes are listed in Supplementary Table S3.

Spatial pattern analysis of regenerating animals

To investigate the spatiotemporal dynamics of positional information during regeneration, we applied the aforementioned coordinate binning strategy to the regenerating samples at each time point. Specifically, the expression profiles of the identified SBGs were mapped onto the A/P, M/L, and D/V axes of the regenerating fragments. The regenerated animals were then divided into 100 bins along the A/P axis, in line with the homeostatic reference, and gene expression was categorized for each sample separately. These cluster IDs function as spatial coordinates rather than static gene lists, allowing us to quantify the physical restoration of morphogenetic gradients over time. By tracking the spatial distribution of these gene clusters over time and comparing them to their homeostatic baselines, we visualized and quantified the restoration of axial polarity and regional patterning across the regeneration process.

Application of Turing pattern models to SBGs

We hypothesize that the interactions between SBGs and their upstream regulators adhere to Turing patterns within an autoregulatory activator-inhibitor framework. Following the removal of the influence of spatial gradients, temporal gene expression data across eight regenerative time points were normalized, interpolated, and smoothed. Reaction, degradation, and diffusion parameters were fine-tuned using linear regression. These optimized parameters allowed us to predict gene expression levels at any post-amputation time point for both established and candidate PCGs.

Pearson’s correlation coefficients were then calculated to assess the accuracy of these predictions.

PCA of SBGs and PCGs

To integrate temporal changes in gene expression with spatial variations, PCA was applied to the binned expression data of selected PCGs along the three axes in homeostatic animals [28]. For the A/P axis, the training set comprised 25 known PCGs. The first principal component (PC1), which accounted for 64.7% of the variance, corresponded to the head-tail gradient, while the second principal component (PC2), which explained 24.7% of the variance, captured fluctuations in the pharyngeal region (convex and concave). Based on their locations in the reduced-dimensional space, genes were manually grouped into five categories: head, head-pharynx, trunk-pharynx, pharynx-tail, and tail domains.

Given the paucity of previously identified PCGs with clear spatial patterns along the M/L and D/V axes in our Stereo-seq data, we expanded the training sets to include 42 and 67 newly inferred PCGs, respectively. Potential M/L PCGs candidates were selected based on Spearman's rank correlation coefficients greater than 0.7 or less than -0.7 for binned SCT-transformed gene expression data, showing patterns similar to known PCGs. For potential D/V PCGs, a fold change greater than 1.5 between dorsal and ventral regions in binned SCT-transformed values served as a selection criterion.

PCA was performed using the Scikit-learn package with default parameters. The eigenvectors derived from this analysis were used to map both established and potential PCGs back into the reduced PCA space, trained on the homeostatic data. The regenerative trajectories of PCGs were visualized, resembling the behavior of an underdamped mass-spring system: the initial amputation stretched the “spring”, disrupting the PCG expression profile, while the GRN provided restorative

feedback to re-establish homeostasis.

UV unwrapping for the head blastema epidermal region

To convert the 3D planarian body shell into a 2D plane, we employed the UV unwrapping technique, a common procedure in the field of computer graphics, using the open-source 3D creation suite Blender (https://www.blender.org/). UV unwrapping is a process in which the surface of a 3D model is mathematically unwrapped and mapped onto a 2D plane, enabling precise application of textures and structures onto a flat surface, which is essential for accurate visualization and analysis. The process of UV unwrapping for the head blastema epidermis involved segmenting the planarian body, marking seams for accurate texture alignment, unwrapping the mesh, and mapping the epidermal cells onto a 2D plane. This approach enabled a high-precision representation of the head blastema's epidermal region, which will be useful for further texture analysis and studies related to planarian regeneration.

Briefly, the planarian body mesh was first segmented into two sections along a defined plane located near the boundary of the head blastema. This segmentation was performed using the Bisect Tool within Blender, which allowed for a clean division of the mesh without distorting the geometry. The separation enabled us to isolate the head blastema area, which we intended to unwrap for detailed analysis.

Next, to prepare for the unwrapping process, seams were strategically marked to guide the unfolding of the 3D mesh. The edges connecting the blastema cutting plane to the anterior pole of the planarian were designated as a seam, specifically placed along the D/V boundary. This step ensured that the unwrapping process adheres to natural anatomical divisions, preventing distortion of the texture in the subsequent 2D plane. Seam marking was carried out using the Blender's UV

editor tool, where the mesh's geometry was manipulated to set boundaries for the unwrapping operation.

Once the seams were defined, the head blastema mesh was subjected to the UV unwrapping operation. The mesh was unfolded into a 2D plane, with particular attention paid to the correct alignment of the marked seam. The seam was clipped and adjusted to create a smooth, curved incision, representing the head blastema epidermal cells in a flat space. This incision helped ensure that the texture mapping would preserve the anatomical integrity of the original 3D model.

Finally, we focused on the outer epidermal cells of the head blastema, which were part of the original 3D point cloud data. These epidermal cells were mapped onto the unwrapped 2D plane using a process that minimized the distance between the original 3D coordinates of each epidermal cell and the vertices of the subdivided mesh surface. This step ensured a precise mapping of cellular structures to the 2D plane, allowing for high fidelity in representing the epidermal region's texture and topology. The minimization of these distances preserved spatial relationships and accurately represented the cellular organization in a flattened format.

Monocle3 analysis

To investigate the state transitions of the ARZ (Clu.31 cluster) during regeneration, we analyzed the trajectory dynamics using raw transcriptome counts from 36 hours post-amputation (hpa) to 14 days post-amputation (dpa). The raw counts were first normalized using SCTransform in Seurat (v4.0.2). Dimensionality reduction and clustering were then performed in Monocle3 (v1.3.1) [44]. The trajectory graph was constructed by fitting the principal graph with the learn_graph function, and pseudotime was calculated with the neoblast cell type set as the root. Marker genes for the ARZ (Clu.31 cluster) were identified using Seurat, and their expression was projected onto the

trajectory branches. Cells were clustered in a 15-NN graph, which allowed us to divide the trajectory into distinct branches enriched for epidermal, muscle, and neuronal signatures.

RNA velocity analysis

RNA velocity analysis, based on Waddington's epigenetic landscape and differential geometry, was used to make continuous, time-resolved predictions of cell state transitions. Cellular genes were aligned to the reference genome to identify exon and intron sequences. The relative abundance of spliced (mature) and unspliced (nascent) mRNAs was calculated to estimate splicing and degradation rates using Velocyto [46]. Each DNB was assigned to its corresponding cell based on its x and y coordinates. Spliced and unspliced count matrices for different domains were processed using the recipe_monocle function in Dynamo [79] to identify highly expressed genes. Following dimensionality reduction, the continuous velocity vector field was reconstructed in UMAP space to predict future cell fates.

Monocle2 analysis

Monocle2 (v2.18.0) was used to analyze the ventral epidermal trajectory across different regeneration time points, following the tutorial (http://cole-trapnell-lab.github.io/monocle-release) [80].We focused on extracting SPC clusters during the putative ventral epidermal transition from the blastema region. Differentially expressed genes for each cell type were identified and used to order the cells. Dimensionality reduction was carried out using the DDRTree method, and the plot_cell_trajectory function was used for visualization. Marker genes identified by Seurat's FindAllMarkers function were projected along the estimated pseudotime to assess their potential role in the transition.

Visual representation of the PRISTA4D interactive spatiotemporal transcriptomic atlas database

To enhance the accessibility and utility of our PRISTA4D (Planarian Regenerative Interactive Spatiotemporal Transcriptomic Atlas in Four Dimensions) for researchers in the field of regeneration, we developed an open-source, interactive database (available at https://www.bgiocean.com/planarian). This platform enables users to explore the spatial distribution and dynamic changes of various genes and cells at different stages of regeneration.

The PRISTA4D database provides several functions, including browsing capabilities and access to experimental procedures, data analysis pipelines, and the ability to download the original dataset. It serves as a resource for studying cell differentiation and spatiotemporal cell interactions within the regeneration research community.

The website includes a homepage and five key functional modules:

3D Model: Visualizes different domains in three dimensions, allowing users to view cellular organization across regeneration stages.

Spatial Clustering Module: Illustrates the distribution of genes and domains, offering insights into spatial gene expression patterns.

Stereo-seq Module: Provides detailed experimental protocols used to generate the transcriptomic data, ensuring transparency and reproducibility.

Sampling Design Module: Offers information on sample design and the data analysis pipelines used, enabling users to understand how the data was processed and analyzed.

Download Module: Grants access to the complete original dataset, allowing users to download the raw data for further analysis and research.

Cell sorting and library construction for scRNA-seq

To prepare the cell suspension for scRNA-seq, CMFB buffer (CMF + 1% FBS) was placed on a cold plate (4 °C), and the animals were incubated in this solution before their tissues were manually chopped to release cells as described previously [8]. After dissociation, the cells were pelleted by centrifugation at 290 × g for 5 minutes at 4 °C. The resulting suspension was passed through a 40 μm filter and stained with DAPI (1:1000; Beyotime) and DRAQ5 (1:1000; BioLegend). The cells were washed and resuspended in CMFB buffer. Flow cytometry and sorting were performed using a Sony MA900 cell sorter, with the temperature maintained at 4 °C to preserve cell integrity. Approximately 20,000 viable cells (DAPI-; DRAQ5+) were loaded onto the SeekGene platform using the Single Cell 3' Transcriptome kit to generate scRNA-seq libraries. Library preparation followed the manufacturer's guidelines to ensure optimal coverage, and sequencing was conducted on an Illumina NovaSeq platform with paired-end 150 base pair (150 PE) reads for detailed transcriptomic profiling.

Analysis of scRNA-seq data after RNAi knockdown

Raw scRNA-seq data were processed and aligned with the *S. mediterranea* reference transcriptome (smed_20140614). Cells containing fewer than 200 detected features or genes expressed in fewer than three cells were excluded. Cells exhibiting unusually high mitochondrial or ribosomal RNA content (where mitochondrial percentage was more than twice the median) were also filtered out based on the annotated reads (Supplementary Table S6). After quality control, the remaining Unique Molecular Identifiers (UMIs) were quantified and analyzed using the Seurat package in R, which enabled normalization, scaling, and dimensionality reduction of the data. PCA was

performed, and the top 30 principal components were used for 2D UMAP generation and clustering within Seurat. Cell lineages were annotated based on the expression of known marker genes, while SPC clusters were identified using markers derived from 3D spatial transcriptomics. Batch effects due to technical variations across samples were corrected using Seurat's integration functions, ensuring that the observed differences were biologically relevant. For analyzing cellular differentiation trajectories and lineage relationships, Monocle 2 was applied. Additionally, MiloR was used to examine and compare the abundance of cells in specific neighborhoods or microenvironments between the control and RNAi-treated groups [81]. Briefly, PCA was used for dimensionality reduction, and a KNN graph was constructed with the buildGraph function (k=30, d=30) based on the top 30 PCA dimensions. Neighborhoods were defined using the makeNhoods function (prop=0.1, k=30, d=30), and differential abundance testing was conducted with default parameters using the distinct function. Differentially abundant cell populations between control and knockdown groups were identified, and differentially expressed genes (DEGs) were determined using DESingle (v1.9.2) with standard settings [78]. Genes with an adjusted p-value of less than 0.05 were considered significantly different. This comparative analysis revealed how RNAi interventions altered the cellular composition within the tissue.

Availability of source code and requirements

Project name: 4D-BioReconX

Project home page: https://github.com/BGI-Qingdao/4D-BioReconX

Operating system(s): Linux or MacOS

Programming language: Jupyter Notebook, Python, R, Shell

Other requirements: anndata>=0.7.5, matplotlib>=3.6.2, numpy>=1.22.4, opencv-python>=4.6.0.66, pandas>=1.4.3, scikit-image>=0.19.2, scipy>=1.9.0, seaborn>=0.11.2

License: MIT License

RRID: SCR_027919

bio.tools ID: 4d-bioreconx

WorkflowHub: 10.48546/workflowhub.workflow.2045.1

Data Availability

All data generated in this study were deposited in the CNGB Nucleotide Sequence Archive (accession code: STT0000028). The accession number for *Rod1* (SMED30003831) is OR211556. Processed data and 3D models can be interactively explored via our PRISTA4D database (https://db.cngb.org/stomics/prista4d). All original code supporting the current study is hosted on GitHub [82] and WorkflowHub [83]. Any additional information required to reanalyze the data reported in this paper is available from the lead contact upon request.

List of abbreviations

A/P: Anterior/Posterior; ARZ: Anterior Regenerative Zone; D/V: Dorsal/Ventral; DEG: Differentially Expressed Gene; dpa: days post-amputation; FISH: Fluorescent In Situ Hybridization; GRN: Gene Regulatory Network; hpa: hours post-amputation; HVG: Highly Variable Gene; M/L: Medial/Lateral; PCA: Principal Component Analysis; PCG: Positional Control Gene; RNAi: RNA interference; SBG: Spatially Biased Gene; scRNA-seq: single-cell RNA sequencing; SPC: Spatial Proximity-based Clustering; ST: Spatial Transcriptomics; TF: Transcription Factor; UMI: Unique Molecular Identifier; WISH: Whole-mount In Situ Hybridization.

Competing interests

The authors declare that they have no competing interests.

Funding

This research was supported by the National Key R&D Program of China (2022YFC3400400), the National Key R&D Program of China (2020YFA0112502 and 2021YFA1100202 to A.Z.), the National Natural Science Foundation of China (32070828 to A.Z.), Shenzhen Science and Technology Program (JCYJ20250604191305008 to M.X. and RCJC20221008092804002 to Y.G.), the Strategic Priority Research Program of the Chinese Academy of Sciences (XDA16021300), the CAS Pioneer Hundred Talents Program (A.Z.), Shanghai Pujiang Program (20PJ1414600 to A.Z.), the Shanghai Science and Technology Committee (STCSM) (22ZR1468400 to A.Z.), Guangdong Genomics Data Center (2021B1212100001) and the Feng Foundation of Biomedical Research (A.Z.).

Authors' contributions

A.Z., X.X., G.F., H.L., K.H. and M.X. conceived and directed the study. A.Z., X.X., M.X. and H.L. supervised the work. Y.C. and YR.L. performed animal experiments and RNAi. XW.L., W.G., JQ.W., W.W. and H.P. performed the Stereo-seq experiments. K.H., M.X., L.G., Y.L., Y.W. and Z.H. analyzed the data. L.G., Y.L. and T.Y. performed database construction. Q.L., L.Z. and X.M. assisted in the data analysis. R.Z., L.L., X.W., H.Z., X.S., S.L., W.Z., S.T.C, J.F., X.L., Y.G., J.W. G.P., and H.Y. performed investigations. A.Z., G.F., H.L., M.X. and K.H. wrote the manuscript with input from all authors.

Acknowledgments

We thank L. Bolund, D. Little and all Zeng lab members for critical reading of the manuscript.

References

1. Forsthoefel DJ and Newmark PA. Emerging patterns in planarian regeneration. Curr Opin Genet Dev. 2009;19 4:412-20. doi:10.1016/j.gde.2009.05.003.
2. Poss KD and Tanaka EM. Hallmarks of regeneration. Cell Stem Cell. 2024;31 9:1244-61. doi:10.1016/j.stem.2024.07.007.

3. Newmark PA and Sanchez Alvarado A. Not your father's planarian: a classic model enters the era of functional genomics. Nat Rev Genet. 2002;3 3:210-9. doi:10.1038/nrg759.
4. Poss KD. Advances in understanding tissue regenerative capacity and mechanisms in animals. Nat Rev Genet. 2010;11 10:710-22. doi:10.1038/nrg2879.
5. Wolpert L. Positional Information and Pattern Formation. Curr Top Dev Biol. 2016;117:597-608. doi:10.1016/bs.ctdb.2015.11.008.
6. Wang M, Hu Q, Lv T, Wang Y, Lan Q, Xiang R, et al. High-resolution 3D spatiotemporal transcriptomic maps of developing Drosophila embryos and larvae. Dev Cell. 2022;57 10:1271-83 e4. doi:10.1016/j.devcel.2022.04.006.
7. Reddien PW and Sanchez Alvarado A. Fundamentals of planarian regeneration. Annu Rev Cell Dev Biol. 2004;20:725-57. doi:10.1146/annurev.cellbio.20.010403.095114.
8. Zeng A, Li H, Guo L, Gao X, McKinney S, Wang Y, et al. Prospectively Isolated Tetraspanin(+) Neoblasts Are Adult Pluripotent Stem Cells Underlying Planaria Regeneration. Cell. 2018;173 7:1593-608 e20. doi:10.1016/j.cell.2018.05.006.
9. Wagner DE, Wang IE and Reddien PW. Clonogenic neoblasts are pluripotent adult stem cells that underlie planarian regeneration. Science. 2011;332 6031:811-6. doi:10.1126/science.1203983.
10. Reddien PW. The Cellular and Molecular Basis for Planarian Regeneration. Cell. 2018;175 2:327-45. doi:10.1016/j.cell.2018.09.021.
11. Adell T, Cebria F and Salo E. Gradients in planarian regeneration and homeostasis. Cold Spring Harb Perspect Biol. 2010;2 1:a000505. doi:10.1101/cshperspect.a000505.
12. Rink JC, Gurley KA, Elliott SA and Sanchez Alvarado A. Planarian Hh signaling regulates regeneration polarity and links Hh pathway evolution to cilia. Science. 2009;326 5958:1406-10. doi:10.1126/science.1178712.
13. Petersen CP and Reddien PW. Smed-betacatenin-1 is required for anteroposterior blastema polarity in planarian regeneration. Science. 2008;319 5861:327-30. doi:10.1126/science.1149943.
14. Cebria F, Kobayashi C, Umesono Y, Nakazawa M, Mineta K, Ikeo K, et al. FGFR-related gene nou-darake restricts brain tissues to the head region of planarians. Nature. 2002;419 6907:620-4. doi:10.1038/nature01042.
15. Umesono Y, Tasaki J, Nishimura Y, Hrouda M, Kawaguchi E, Yazawa S, et al. The molecular logic for planarian regeneration along the anterior-posterior axis. Nature. 2013;500 7460:73-6. doi:10.1038/nature12359.
16. Witchley JN, Mayer M, Wagner DE, Owen JH and Reddien PW. Muscle cells provide instructions for planarian regeneration. Cell Rep. 2013;4 4:633-41. doi:10.1016/j.celrep.2013.07.022.
17. Benham-Pyle BW, Brewster CE, Kent AM, Mann FG, Jr., Chen S, Scott AR, et al. Identification of rare, transient post-mitotic cell states that are induced by injury and required for whole-body regeneration in Schmidtea mediterranea. Nat Cell Biol. 2021;23 9:939-52. doi:10.1038/s41556-021-00734-6.
18. Garcia-Castro H and Solana J. Single-cell transcriptomics in planaria: new tools allow new insights into cellular and evolutionary features. Biochem Soc Trans. 2022;50 5:1237-46. doi:10.1042/BST20210825.
19. Wurtzel O, Cote LE, Poirier A, Satija R, Regev A and Reddien PW. A Generic and Cell-Type-Specific Wound Response Precedes Regeneration in Planarians. Dev Cell. 2015;35 5:632-45. doi:10.1016/j.devcel.2015.11.004.
20. Molinaro AM and Pearson BJ. In silico lineage tracing through single cell transcriptomics identifies a neural stem cell population in planarians. Genome Biol. 2016;17:87. doi:10.1186/s13059-016-0937-9.
21. van Wolfswinkel JC, Wagner DE and Reddien PW. Single-cell analysis reveals functionally distinct classes within the planarian stem cell compartment. Cell Stem Cell. 2014;15 3:326-39. doi:10.1016/j.stem.2014.06.007.
22. Fincher CT, Wurtzel O, de Hoog T, Kravarik KM and Reddien PW. Cell type transcriptome atlas

for the planarian Schmidtea mediterranea. Science. 2018;360 6391 doi:10.1126/science.aaq1736.

23. Plass M, Solana J, Wolf FA, Ayoub S, Misios A, Glažar P, et al. Cell type atlas and lineage tree of a whole complex animal by single-cell transcriptomics. Science. 2018;360 6391 doi:10.1126/science.aaq1723.

24. Cui G, Dong K, Zhou JY, Li S, Wu Y, Han Q, et al. Spatiotemporal transcriptomic atlas reveals the dynamic characteristics and key regulators of planarian regeneration. Nat Commun. 2023;14 1:3205. doi:10.1038/s41467-023-39016-0.

25. Park C, Owusu-Boaitey KE, Valdes GM and Reddien PW. Fate specification is spatially intermingled across planarian stem cells. Nat Commun. 2023;14 1:7422. doi:10.1038/s41467-023-43267-2.

26. Kato K, Orii H, Watanabe K and Agata K. Dorsal and ventral positional cues required for the onset of planarian regeneration may reside in differentiated cells. Dev Biol. 2001;233 1:109-21. doi:10.1006/dbio.2001.0226.

27. Chen A, Liao S, Cheng M, Ma K, Wu L, Lai Y, et al. Spatiotemporal transcriptomic atlas of mouse organogenesis using DNA nanoball-patterned arrays. Cell. 2022;185 10:1777-92 e21. doi:10.1016/j.cell.2022.04.003.

28. Sun M, Wang Y, Han K, Guo L, Chen Y, Li Y, et al. Cell Type Architecture and Positional Gene Gradients in an Adult Animal at Subcellular Resolution. bioRxiv. 2026:2026.02.19.705280. doi:10.64898/2026.02.19.705280.

29. Roberts-Galbraith RH, Brubacher JL and Newmark PA. A functional genomics screen in planarians reveals regulators of whole-brain regeneration. Elife. 2016;5 doi:10.7554/eLife.17002.

30. Levin M, Pietak AM and Bischof J. Planarian regeneration as a model of anatomical homeostasis: Recent progress in biophysical and computational approaches. Semin Cell Dev Biol. 2019;87:125-44. doi:10.1016/j.semcdb.2018.04.003.

31. King RS and Newmark PA. In situ hybridization protocol for enhanced detection of gene expression in the planarian Schmidtea mediterranea. BMC Dev Biol. 2013;13:8. doi:10.1186/1471-213X-13-8.

32. Stuckemann T, Cleland JP, Werner S, Thi-Kim Vu H, Bayersdorf R, Liu SY, et al. Antagonistic Self-Organizing Patterning Systems Control Maintenance and Regeneration of the Anteroposterior Axis in Planarians. Dev Cell. 2017;40 3:248-63 e4. doi:10.1016/j.devcel.2016.12.024.

33. Lander R and Petersen CP. Wnt, Ptk7, and FGFRL expression gradients control trunk positional identity in planarian regeneration. Elife. 2016;5 doi:10.7554/eLife.12850.

34. Scimone ML, Cote LE, Rogers T and Reddien PW. Two FGFRL-Wnt circuits organize the planarian anteroposterior axis. Elife. 2016;5 doi:10.7554/eLife.12845.

35. Kobayashi C, Saito Y, Ogawa K and Agata K. Wnt signaling is required for antero-posterior patterning of the planarian brain. Dev Biol. 2007;306 2:714-24. doi:10.1016/j.ydbio.2007.04.010.

36. Gurley KA, Elliott SA, Simakov O, Schmidt HA, Holstein TW and Sanchez Alvarado A. Expression of secreted Wnt pathway components reveals unexpected complexity of the planarian amputation response. Dev Biol. 2010;347 1:24-39. doi:10.1016/j.ydbio.2010.08.007.

37. Inman DJ. Engineering Vibration (3rd Edition). Pearson Education, Inc; 2007.

38. Macarthur BD, Ma'ayan A and Lemischka IR. Systems biology of stem cell fate and cellular reprogramming. Nat Rev Mol Cell Biol. 2009;10 10:672-81. doi:10.1038/nrm2766.

39. Gierer A and Meinhardt H. A theory of biological pattern formation. Kybernetik. 1972;12 1:30-9. doi:10.1007/BF00289234.

40. Werner S, Stuckemann T, Beiran Amigo M, Rink JC, Julicher F and Friedrich BM. Scaling and regeneration of self-organized patterns. Phys Rev Lett. 2015;114 13:138101. doi:10.1103/PhysRevLett.114.138101.

41. Cowles MW, Brown DD, Nisperos SV, Stanley BN, Pearson BJ and Zayas RM. Genome-wide analysis of the bHLH gene family in planarians identifies factors required for adult neurogenesis and neuronal regeneration. Development. 2013;140 23:4691-702. doi:10.1242/dev.098616.

42. Sureda-Gomez M and Adell T. Planarian organizers. Semin Cell Dev Biol. 2019;87:95-104. doi:10.1016/j.semcdb.2018.05.021.
43. Scimone ML, Cloutier JK, Maybrun CL and Reddien PW. The planarian wound epidermis gene equinox is required for blastema formation in regeneration. Nat Commun. 2022;13 1:2726. doi:10.1038/s41467-022-30412-6.
44. Cao J, Spielmann M, Qiu X, Huang X, Ibrahim DM, Hill AJ, et al. The single-cell transcriptional landscape of mammalian organogenesis. Nature. 2019;566 7745:496-502. doi:10.1038/s41586-019-0969-x.
45. Kent AM, Guerrero-Hernández C, Brewster C, McKinney S, Morrison JA, McKinney MC, et al. Metabolites produced by agat+ cells support regeneration in the planarian Schmidtea mediterranea. Dev Biol. 2026;529:106-20. doi:10.1016/j.ydbio.2025.10.001.
46. La Manno G, Soldatov R, Zeisel A, Braun E, Hochgerner H, Petukhov V, et al. RNA velocity of single cells. Nature. 2018;560 7719:494-8. doi:10.1038/s41586-018-0414-6.
47. Brower CS, Sato S, Tomomori-Sato C, Kamura T, Pause A, Stearman R, et al. Mammalian mediator subunit mMED8 is an Elongin BC-interacting protein that can assemble with Cul2 and Rbx1 to reconstitute a ubiquitin ligase. Proc Natl Acad Sci U S A. 2002;99 16:10353-8. doi:10.1073/pnas.162424199.
48. Gurley KA, Rink JC and Sanchez Alvarado A. Beta-catenin defines head versus tail identity during planarian regeneration and homeostasis. Science. 2008;319 5861:323-7. doi:10.1126/science.1150029.
49. Brown DDR, Molinaro AM and Pearson BJ. The planarian TCF/LEF factor Smed-tcf1 is required for the regeneration of dorsal-lateral neuronal subtypes. Dev Biol. 2018;433 2:374-83. doi:10.1016/j.ydbio.2017.08.024.
50. Pearson BJ and Sanchez Alvarado A. A planarian p53 homolog regulates proliferation and self-renewal in adult stem cell lineages. Development. 2010;137 2:213-21. doi:10.1242/dev.044297.
51. Yin JW and Wang G. The Mediator complex: a master coordinator of transcription and cell lineage development. Development. 2014;141 5:977-87. doi:10.1242/dev.098392.
52. Benham-Pyle BW, Mann FG, Brewster CE, Dewars ER, Vuu DM, Nowotarski SH, et al. Planarians employ diverse and dynamic stem cell microenvironments to support whole-body regeneration. bioRxiv. 2023:2022.03.20.485025. doi:10.1101/2022.03.20.485025.
53. Herath S and Lobo D. Cross-inhibition of Turing patterns explains the self-organized regulatory mechanism of planarian fission. J Theor Biol. 2020;485:110042. doi:10.1016/j.jtbi.2019.110042.
54. Pietak A, Bischof J, LaPalme J, Morokuma J and Levin M. Neural control of body-plan axis in regenerating planaria. PLoS Comput Biol. 2019;15 4:e1006904. doi:10.1371/journal.pcbi.1006904.
55. Stückemann T, Cleland JP, Werner S, Thi-Kim Vu H, Bayersdorf R, Liu SY, et al. Antagonistic Self-Organizing Patterning Systems Control Maintenance and Regeneration of the Anteroposterior Axis in Planarians. Dev Cell. 2017;40 3:248-63.e4. doi:10.1016/j.devcel.2016.12.024.
56. Wurtzel O, Oderberg IM and Reddien PW. Planarian Epidermal Stem Cells Respond to Positional Cues to Promote Cell-Type Diversity. Dev Cell. 2017;40 5:491-504 e5. doi:10.1016/j.devcel.2017.02.008.
57. Currie KW, Molinaro AM and Pearson BJ. Neuronal sources of hedgehog modulate neurogenesis in the adult planarian brain. Elife. 2016;5 doi:10.7554/eLife.19735.
58. Chan A, Ma S, Pearson BJ and Chan D. Collagen IV differentially regulates planarian stem cell potency and lineage progression. Proc Natl Acad Sci U S A. 2021;118 16 doi:10.1073/pnas.2021251118.
59. Hill EM and Petersen CP. Positional information specifies the site of organ regeneration and not tissue maintenance in planarians. Elife. 2018;7 doi:10.7554/eLife.33680.
60. Wu W, Liu S, Wu H, Chen M, Gao L, Zhao B, et al. DjPtpn11 is an essential modulator of planarian (Dugesia japonica) regeneration. Int J Biol Macromol. 2022;209 Pt A:1054-64.

doi:10.1016/j.ijbiomac.2022.04.095.
61. Oderberg IM, Li DJ, Scimone ML, Gavino MA and Reddien PW. Landmarks in Existing Tissue at Wounds Are Utilized to Generate Pattern in Regenerating Tissue. Curr Biol. 2017;27 5:733-42. doi:10.1016/j.cub.2017.01.024.
62. Christensen RN and Tassava RA. Apical epithelial cap morphology and fibronectin gene expression in regenerating axolotl limbs. Dev Dyn. 2000;217 2:216-24. doi:10.1002/(SICI)1097-0177(200002)217:2<216::AID-DVDY8>3.0.CO;2-8.
63. McCusker C, Bryant SV and Gardiner DM. The axolotl limb blastema: cellular and molecular mechanisms driving blastema formation and limb regeneration in tetrapods. Regeneration (Oxf). 2015;2 2:54-71. doi:10.1002/reg2.32.
64. Zhong J, Aires R, Tsissios G, Skoufa E, Brandt K, Sandoval-Guzman T, et al. Multi-species atlas resolves an axolotl limb development and regeneration paradox. Nat Commun. 2023;14 1:6346. doi:10.1038/s41467-023-41944-w.
65. Burrows JT, Pearson BJ and Scott IC. An in vivo requirement for the mediator subunit med14 in the maintenance of stem cell populations. Stem Cell Reports. 2015;4 4:670-84. doi:10.1016/j.stemcr.2015.02.006.
66. Pascual-Carreras E, Marin-Barba M, Castillo-Lara S, Coronel-Cordoba P, Magri MS, Wheeler GN, et al. Wnt/beta-catenin signalling is required for pole-specific chromatin remodeling during planarian regeneration. Nat Commun. 2023;14 1:298. doi:10.1038/s41467-023-35937-y.
67. Blassberg RA, Felix DA, Tejada-Romero B and Aboobaker AA. PBX/extradenticle is required to re-establish axial structures and polarity during planarian regeneration. Development. 2013;140 4:730-9. doi:10.1242/dev.082982.
68. Chen CC, Wang IE and Reddien PW. pbx is required for pole and eye regeneration in planarians. Development. 2013;140 4:719-29. doi:10.1242/dev.083741.
69. Vogg MC, Owlarn S, Perez Rico YA, Xie J, Suzuki Y, Gentile L, et al. Stem cell-dependent formation of a functional anterior regeneration pole in planarians requires Zic and Forkhead transcription factors. Dev Biol. 2014;390 2:136-48. doi:10.1016/j.ydbio.2014.03.016.
70. Scimone ML, Lapan SW and Reddien PW. A forkhead transcription factor is wound-induced at the planarian midline and required for anterior pole regeneration. PLoS Genet. 2014;10 1:e1003999. doi:10.1371/journal.pgen.1003999.
71. Vasquez-Doorman C and Petersen CP. zic-1 Expression in Planarian neoblasts after injury controls anterior pole regeneration. PLoS Genet. 2014;10 7:e1004452. doi:10.1371/journal.pgen.1004452.
72. Newmark PA and Sanchez Alvarado A. Bromodeoxyuridine specifically labels the regenerative stem cells of planarians. Dev Biol. 2000;220 2:142-53. doi:10.1006/dbio.2000.9645.
73. Adler CE, Seidel CW, McKinney SA and Sanchez Alvarado A. Selective amputation of the pharynx identifies a FoxA-dependent regeneration program in planaria. Elife. 2014;3:e02238. doi:10.7554/eLife.02238.
74. Newmark PA, Reddien PW, Cebria F and Sanchez Alvarado A. Ingestion of bacterially expressed double-stranded RNA inhibits gene expression in planarians. Proc Natl Acad Sci U S A. 2003;100 Suppl 1 Suppl 1:11861-5. doi:10.1073/pnas.1834205100.
75. Pearson BJ, Eisenhoffer GT, Gurley KA, Rink JC, Miller DE and Sanchez Alvarado A. Formaldehyde-based whole-mount in situ hybridization method for planarians. Dev Dyn. 2009;238 2:443-50. doi:10.1002/dvdy.21849.
76. Forsthoefel DJ, Cejda NI, Khan UW and Newmark PA. Cell-type diversity and regionalized gene expression in the planarian intestine. Elife. 2020;9 doi:10.7554/eLife.52613.
77. Emili E, Pérez-Posada A, Vanni V, Salamanca-Díaz D, Ródriguez-Fernández D, Christodoulou MD, et al. Allometry of cell types in planarians by single-cell transcriptomics. Sci Adv. 2025;11 19:eadm7042. doi:10.1126/sciadv.adm7042.
78. Miao Z, Deng K, Wang X and Zhang X. DEsingle for detecting three types of differential expression in single-cell RNA-seq data. Bioinformatics. 2018;34 18:3223-4.

doi:10.1093/bioinformatics/bty332.
79. Qiu X, Zhang Y, Martin-Rufino JD, Weng C, Hosseinzadeh S, Yang D, et al. Mapping transcriptomic vector fields of single cells. Cell. 2022;185 4:690-711 e45. doi:10.1016/j.cell.2021.12.045.
80. Qiu X, Hill A, Packer J, Lin D, Ma YA and Trapnell C. Single-cell mRNA quantification and differential analysis with Census. Nat Methods. 2017;14 3:309-15. doi:10.1038/nmeth.4150.
81. Dann E, Henderson NC, Teichmann SA, Morgan MD and Marioni JC. Differential abundance testing on single-cell data using k-nearest neighbor graphs. Nat Biotechnol. 2022;40 2:245-53. doi:10.1038/s41587-021-01033-z.
82. BGI-Qingdao: 4D-BioReconX. https://github.com/BGI-Qingdao/4D-BioReconX (2025). Accessed 2026-02-24.
83. Lidong G. 4D-BioReconX: a bioinformatic framework for reconstructing 4D spatial transcriptomics atlas and spatiotemporal analyses+. WorkflowHub. 2026; doi:10.48546/workflowhub.workflow.2045.1.

1207 **Figures**

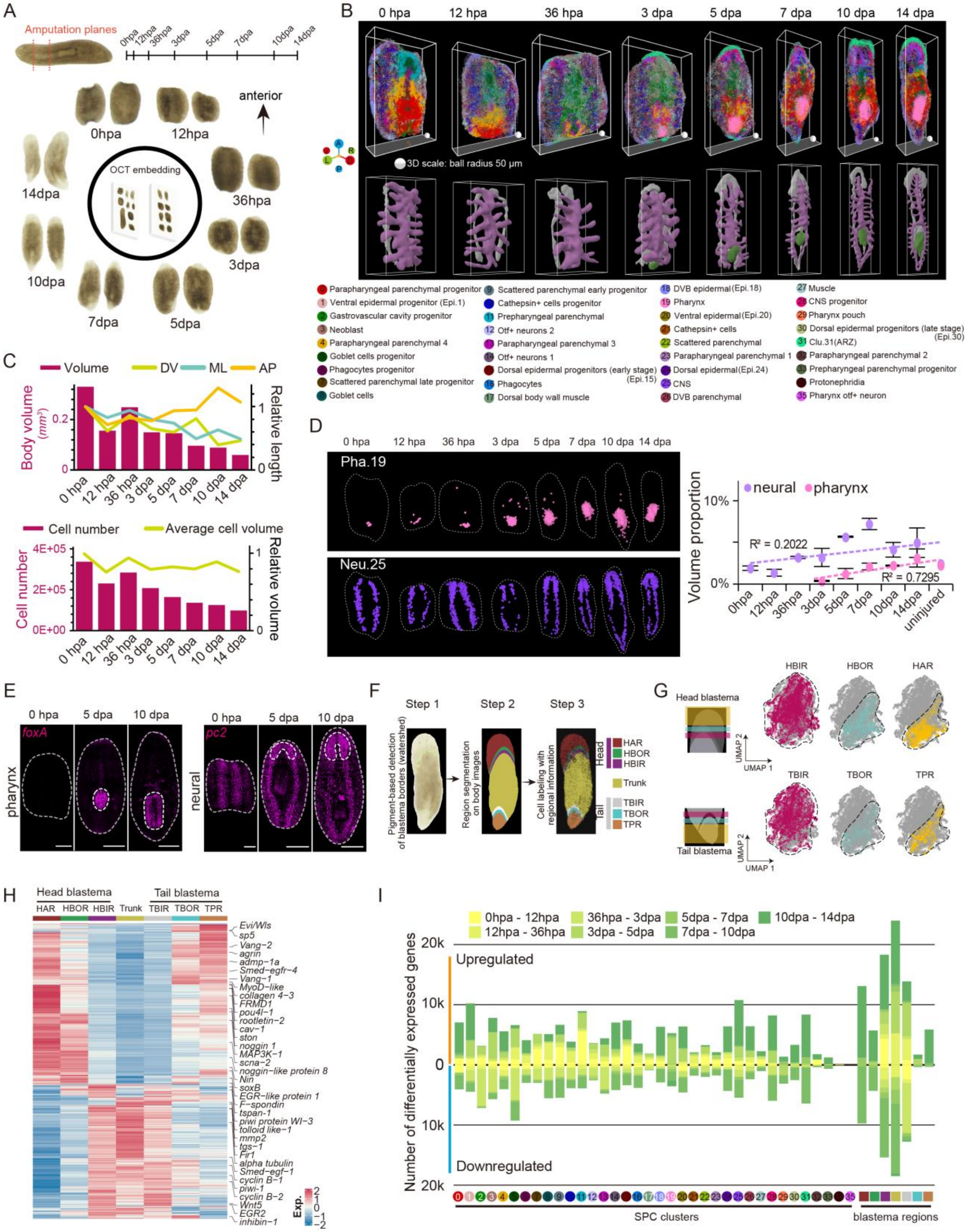

1208

Figure 1: 3D molecular reconstruction of whole-body planarian regeneration using 4D spatial transcriptomics. (A) Schematic representation of the amputation strategy and sampling design for planarian whole-body regeneration (WBR). The dotted line indicates the amputated pre-pharyngeal fragments. The central diagram illustrates the arrangement of 17 embedded tissue samples per block, including one intact sample and two replicates at each of the eight time points post-amputation. **(B)** 3D spatial visualization of 36 SPC clusters (top) and tissue meshes (bottom) in representative animals at eight time points during WBR. SPC clusters are labeled in the bottom panel. Tissue meshes highlight the intestine (purple), pharynx (green), and central nervous system (gray). **(C)** Top: Bar plots showing changes in body volume size; line charts depicting variations in the length of the D/V, M/L, and A/P axes relative to the 0 hpa sample at eight time points of regeneration. Bottom: Bar plots showing SPC cell counts and line charts illustrating the average cell volume across the eight time points of regeneration. **(D)** Left: Spatial patterns of the pharynx (Pha.19) and CNS (Neu.25) clusters at eight regenerative time points. Right: Line plots showing the volume proportions of the reconstructed organs (neural and pharynx) during regeneration. Error bars represent the standard deviation from two replicates. Linear fitting lines show nearly linear time-dependent changes. **(E)** FISH staining showing spatial patterns of pharynx (*foxA*) and neural (*pc2*) markers during regeneration. Scale bars: 500 μm. $n \geq 3$. **(F)** Schematic diagrams illustrating the three-step process for identifying blastema subdomains based on the watershed algorithm for pigment variations. The head blastema region is divided into HAR (head anterior region), HBOR (head border outer region), and HBIR (head border inner region), while the tail blastema region includes TPR (tail posterior region), TBOR (tail border outer region), and TBIR (tail border inner region). **(G)** Left: Conceptual schematic illustrating the spatial definition of blastema sub-regions based on relative distance to the wound. Right: UMAP visualization of

transcriptomic profiles from the corresponding cell populations. Colors indicate the distinct regions as defined in (F). **(H)** Heatmap depicting the relative expression of genes enriched in different blastema regions across all time points. **(I)** Stacked bar plot illustrating the number of differentially expressed genes in each SPC cell type or blastema region under the indicated comparison conditions.

1237

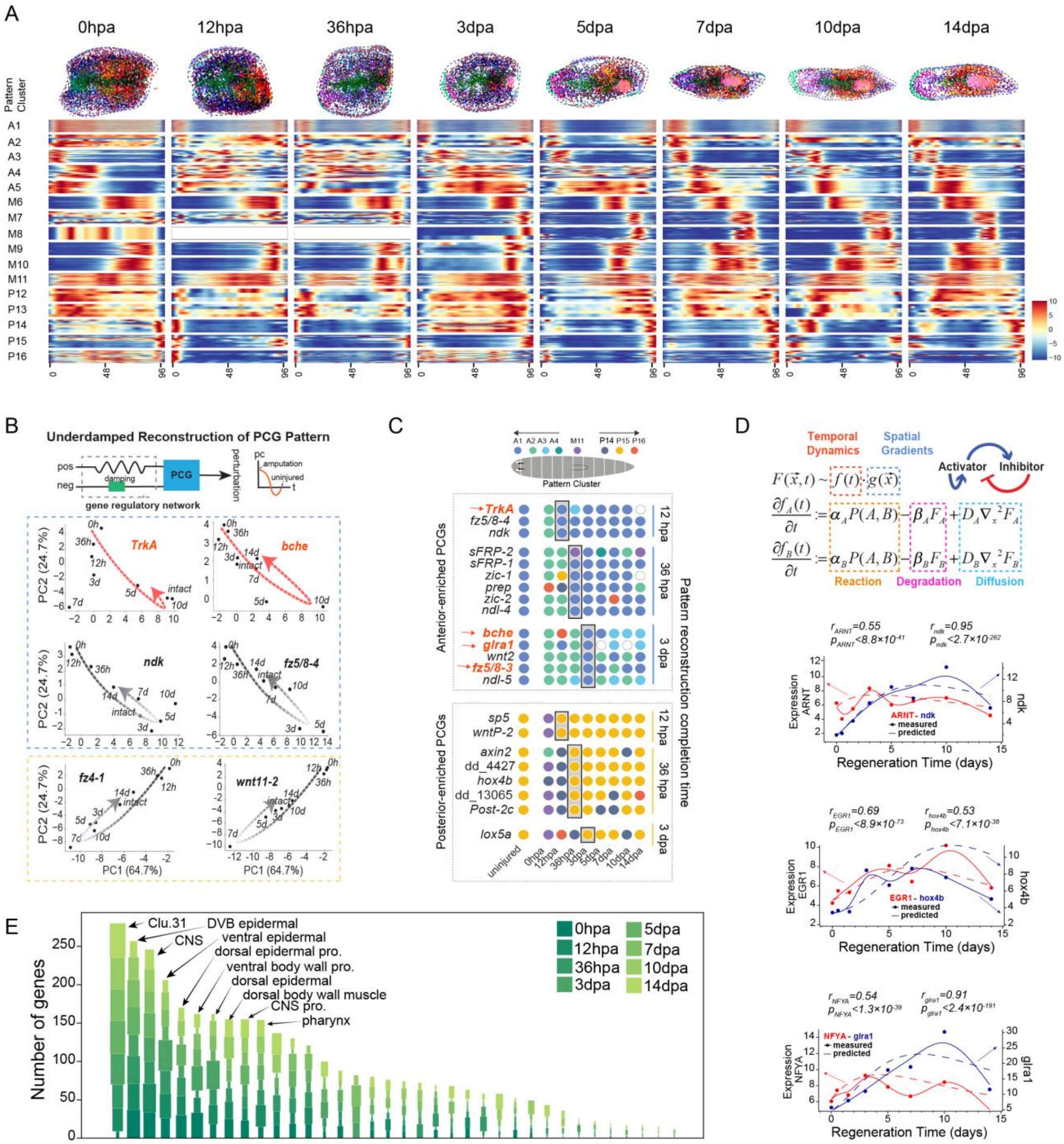
A
0hpa
12hpa
36hpa
3dpa
5dpa
7dpa
10dpa
14dpa
Pattern Cluster
A1
A2
A3
A4
A5
M6
M7
M8
M9
M10
M11
P12
P13
P14
P15
P16
B
Underdamped Reconstruction of PCG Pattern
pos
neg
damping
PCG
gene regulatory network
perturbation
amputation
uninjured
TrkA
bche
ndk
fz5/8-4
fz4-1
wnt11-2
PC2 (24.7%)
PC1 (64.7%)
C
Pattern Cluster
Anterior-enriched PCGs
Posterior-enriched PCGs
Pattern reconstruction completion time
TrkA
fz5/8-4
ndk
sFRP-2
sFRP-1
zic-1
prep
zic-2
ndl-4
bche
glra1
wnt2
fz5/8-3
ndl-5
sp5
wntP-2
axin2
dd_4427
hox4b
dd_13065
Post-2c
lox5a
12 hpa
36 hpa
3 dpa
uninjured
D
Temporal Dynamics
Spatial Gradients
Activator
Inhibitor
Reaction
Degradation
Diffusion
Regeneration Time (days)
ARNT - ndk
EGR1 - hox4b
NFYA - glra1
measured
predicted
E
Number of genes
Clu.31
DVB epidermal
ventral epidermal
CNS
dorsal epidermal pro.
ventral body wall pro.
dorsal epidermal
dorsal body wall muscle
CNS pro.
pharynx

Figure 2: Dynamics of spatially biased genes (SBGs) during whole-body regeneration. (A) Heatmaps showing changes in spatial expression patterns along the A/P axis during regeneration for the 16 A/P pattern cluster genes. These genes exhibit spatially biased expression along the A/P axis in intact animals. Hollow rectangles indicate the absence of specific gene expression clusters at particular time points. Animals are virtually divided into 100 sections along the A/P axis. Left margin annotations indicate cluster numbers. **(B)** PCA representation of pattern reconstruction for known and potential PCG candidates. PC1 corresponds to the head-tail gradient feature, while PC2 captures fluctuations in the pharynx region (convex and concave). Time-resolved trajectories in PCA space reveal universal self-organized dynamics during SBG pattern regeneration, resembling an underdamped mass-spring system (top). **(C)** Schematic diagrams illustrating the hierarchical reconstruction of SBG patterns, colored by cluster ID. SBGs are arranged by the timing of repatterning completion, as shown on the right. Hollow circles indicate the absence of expression at specific time points. Red arrows highlight newly identified SBGs. The color gradient represents the recovery of SBGs at different stages post-amputation. The box highlights the time points when repatterning is complete. **(D)** Spatiotemporal modeling of SBG patterns during regeneration. The interaction between SBGs and their upstream regulators generates Turing patterns in an autoregulatory activator-inhibitor system (top). Gene expression at any time point after amputation can be predicted. The lower line charts compare predicted repatterning (dashed line) with measured data (solid line) for both known and newly identified SBGs. r, Pearson's correlation. **(E)** Bar plot showing the number of SBGs highly enriched in each SPC cluster at different time points during regeneration.

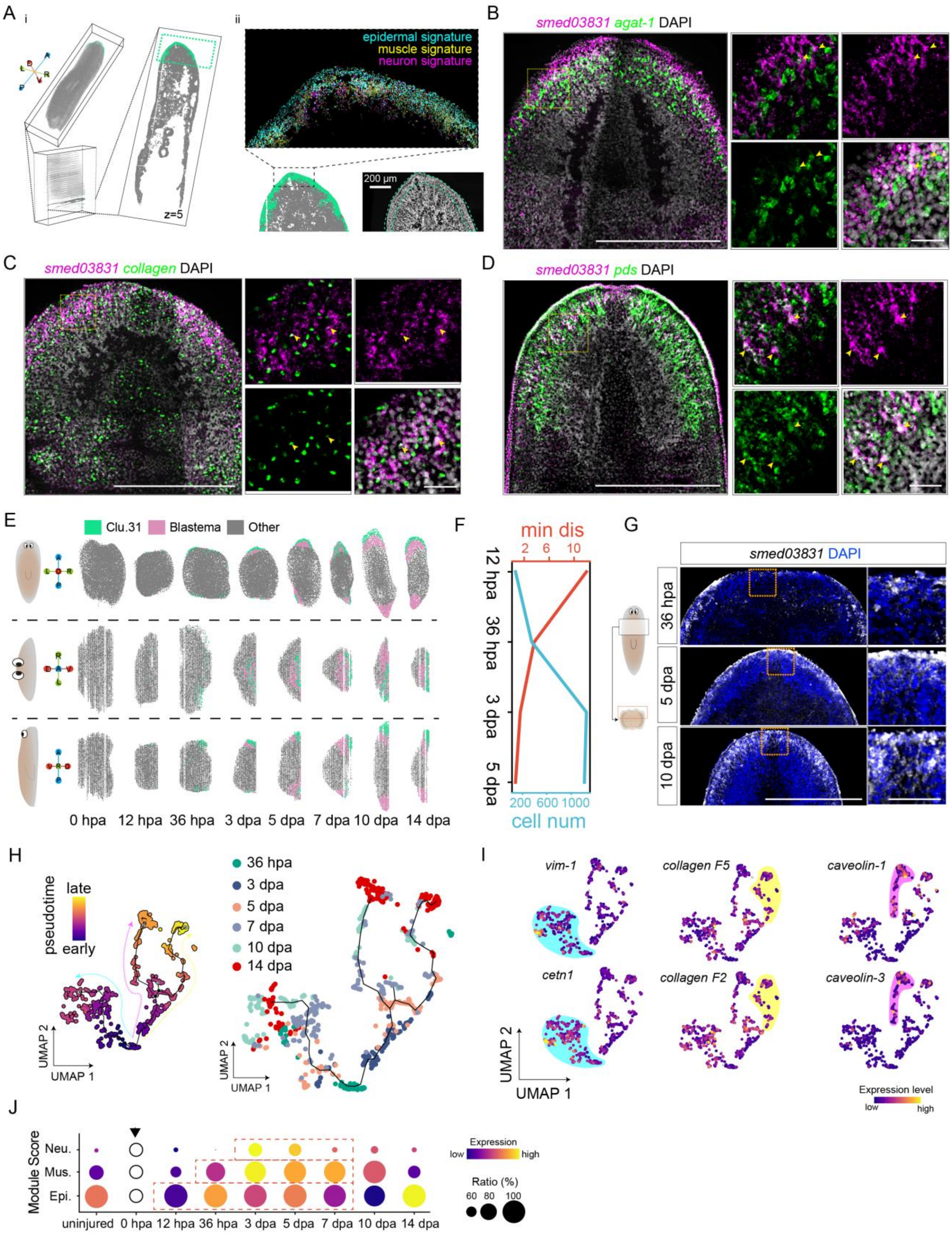
A
i
ii
z=5
epidermal signature
muscle signature
neuron signature
200 μm
B
smed03831 agat-1 DAPI
C
smed03831 collagen DAPI
D
smed03831 pds DAPI
E
Clu.31
Blastema
Other
0 hpa
12 hpa
36 hpa
3 dpa
5 dpa
7 dpa
10 dpa
14 dpa
F
min dis
2
6
10
12 hpa
36 hpa
3 dpa
5 dpa
200
600
1000
cell num
G
smed03831 DAPI
36 hpa
5 dpa
10 dpa
H
pseudotime
late
early
UMAP 2
UMAP 1
36 hpa
3 dpa
5 dpa
7 dpa
10 dpa
14 dpa
I
vim-1
collagen F5
caveolin-1
cetn1
collagen F2
caveolin-3
UMAP 2
UMAP 1
Expression level
low
high
J
Module Score
Neu.
Mus.
Epi.
uninjured
0 hpa
12 hpa
36 hpa
3 dpa
5 dpa
7 dpa
10 dpa
14 dpa
Expression
low
high
Ratio (%)
60
80
100

Figure 3: Identification and characterization of the ARZ (Clu.31) domain in the blastema region. (A) Spatial visualization of the Clu.31 domain in a homeostatic worm. (i) 3D spatial transcriptomics data showing Clu.31 (green) with the right panel displaying the fifth section from ventral to dorsal (z = 5). (ii) Top: Enlarged view of three lineage signatures within Clu.31 in a single slice. Bottom left: Enlarged view of the head region from (i). Bottom right: ssDNA staining highlights the presence of multiple cell layers coexisting in Clu.31, with the green dashed line indicating the location of Clu.31. **(B-D)** FISH staining for the Clu.31 marker (*smed03831*, magenta) with the epidermal marker agat-1 (green) (B), muscle marker collagen (green) (C), and neuron marker pds (green) (D). Co-expressed cells are indicated by yellow arrowheads. Scale bars: 500 µm (left); 50 µm (right). $n \geq 3$. **(E)** Spatial distribution of the Clu.31 domain during regeneration, shown from top, front, and side views. Green dots represent SPC cells within Clu.31, pink dots represent SPC cells in the blastema region, and grey dots represent other SPCs. **(F)** The position and cell number of Clu.31 during regeneration. Top: Line plot (red) showing the decreased minimal distance (min dis) of Clu.31 to the wound surface, accompanied by an increase in the cell number (Cell num) of Clu.31 during wound healing (yellow line). Min dis represents the minimal distance to the wound surface in UV spatial coordinates, while Cell num refers to the number of Clu.31 cells. **(G)** FISH staining showing *smed03831* expression in the head blastema during regeneration. Enlarged areas are shown to the right. $n \geq 3$. Scale bars: 500 µm (left); 50 µm (right). **(H)** Pseudotime trajectory analysis of Clu.31 across six time points of whole-body regeneration (WBR), from 36 hpa to 14 dpa. **(I)** Feature plot showing the expression of representative cell-type marker genes—epidermis (left), muscle (middle), and neuronal (right) lineages—along pseudotime trajectories from (H). **(J)** Bubble plot displaying the gene set module scores of markers for neural (Neu.), muscular (Mus.), and epidermal (Epi.) lineages within Clu.31 during

regeneration.

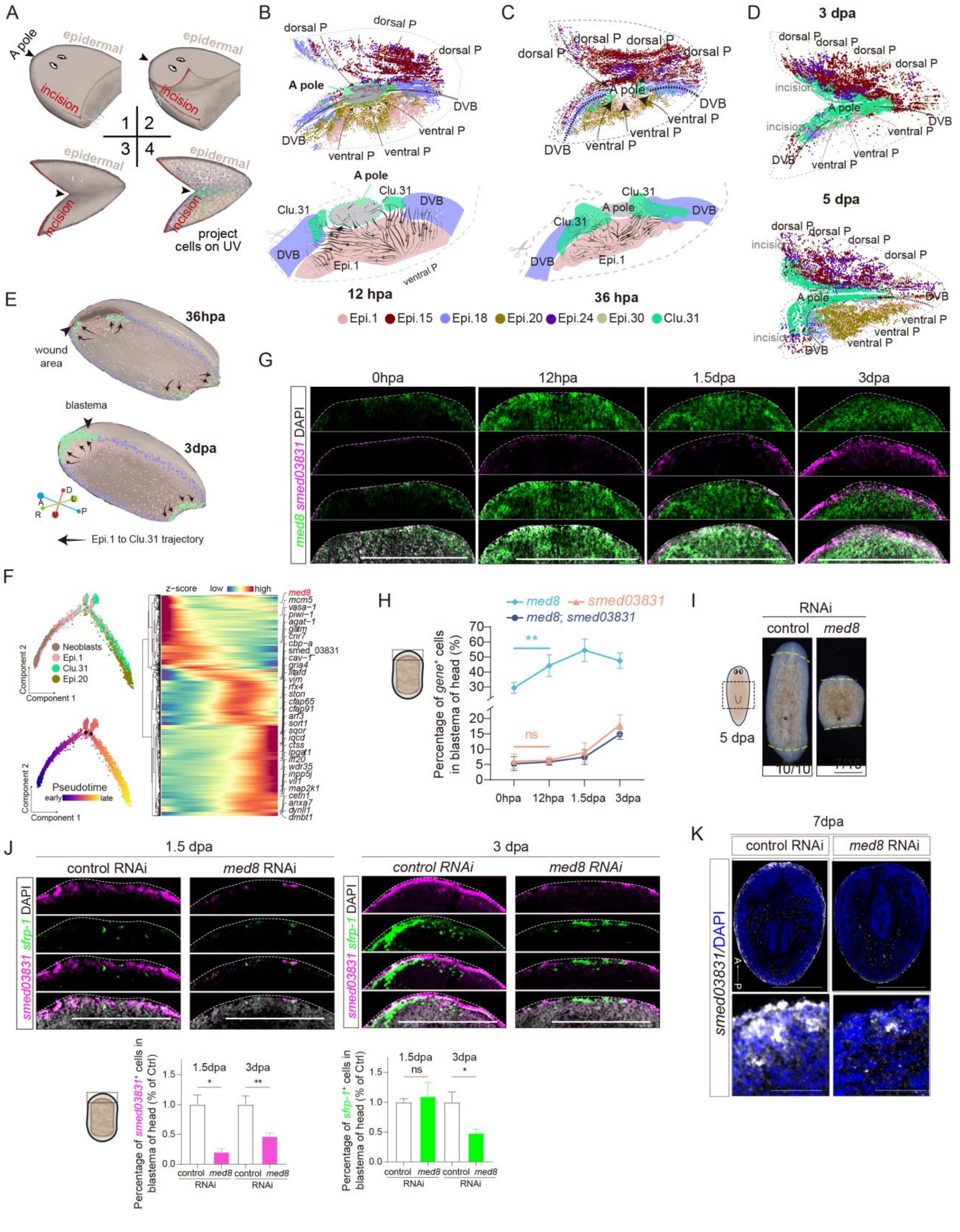
A
A pole
epidermal
incision
1 2
3 4
project
cells on UV
B
dorsal P
A pole
DVB
ventral P
Clu.31
Epi.1
12 hpa
C
36 hpa
D
3 dpa
5 dpa
incision
Epi.1 Epi.15 Epi.18 Epi.20 Epi.24 Epi.30 Clu.31
E
36hpa
wound
area
blastema
3dpa
Epi.1 to Clu.31 trajectory
F
Neoblasts
Epi.1
Clu.31
Epi.20
Component 1
Component 2
Pseudotime
early late
z-score low high
med8
mcm5
vasa-1
piwi-1
agat-1
gatm
cnr7
cbp-a
smed_03831
cav-1
gria4
lltafd
vim
rfx4
ston
cfap65
cfap91
arf3
sort1
sqor
iqcd
ctss
lpgat1
ift20
wdr35
inpp5j
vil1
map2k1
cetn1
anxa7
dynll1
dmbt1
G
0hpa
12hpa
1.5dpa
3dpa
med8 smed03831 DAPI
H
med8
smed03831
med8; smed03831
Percentage of gene+ cells in blastema of head (%)
**
ns
0hpa 12hpa 1.5dpa 3dpa
I
RNAi
control
med8
5 dpa
10/10
7/10
J
1.5 dpa
3 dpa
control RNAi
med8 RNAi
smed03831 sfrp-1 DAPI
Percentage of smed03831+ cells in blastema of head (% of Ctrl)
Percentage of sfrp-1+ cells in blastema of head (% of Ctrl)
1.5dpa
3dpa
*
**
ns
control med8
RNAi
K
7dpa
control RNAi
med8 RNAi
smed03831/DAPI
A—P

Figure 4: Cellular composition and regulation of the ARZ domain. (A) Schematic illustrating the workflow for unwrapping the 3D epidermal surface of the head blastema into 2D spatial coordinates. UV unwrapping of the 3D mesh was performed by manually marking a seam along the D/V boundary (Step 1) and unwrapping the surface by cutting along the seam (Steps 2, 3). Epidermal cells were projected onto the 2D coordinates by minimizing the distance between each 3D cell center and the nearest subdivision vertices (Step 4). See Methods for details. **(B)** UV spatial mapping of the ventral epidermal transition forming the Clu.31 domain at 12 hours post-amputation (hpa). Top: Distribution of Clu.31 (green) and epidermal SPCs on the unwrapped UV map, with dot colors representing SPC types. Labels and dashed lines indicate key positions. Bottom: Predicted cell transition streams of the epidermis as modeled using Dynamo. DVB, Dorsal-Ventral Boundary; A pole, anterior pole; P, posterior. **(C)** UV spatial map showing Clu.31 transition patterns at 36 hpa. Top: Distribution of Clu.31 and epidermal SPCs on the unwrapped UV spatial map. Bottom: Cell transition streams of the epidermis predicted using Dynamo. **(D)** UV spatial map showing Clu.31 transition patterns at 3 days post-amputation (dpa, top) and 5 dpa (bottom). The distribution of Clu.31 (green) and epidermal SPCs on the unwrapped UV spatial map is shown. **(E)** Visualization of Clu.31 movement during wound healing (top, 36 hpa) and blastema formation (bottom, 3 dpa). The white arrow indicates the predicted trajectory of the epidermal transition, delineated based on the spatial dynamo shown in (C) and (D). Arrowheads highlight the location of either the wound surface (top) or blastema (bottom). **(F)** Pseudotime trajectory analysis of ventral epidermis and neoblast cells. Left: Distinct states of SPCs identified by pseudotime analysis, with cells colored by SPC clusters (top left) and pseudotime (bottom left). Right: Heatmap showing significantly altered genes discovered by Monocle 2 along the trajectory. **(G)** Expression and localization of *med8* and *smed03831* in the head blastema of regenerative

fragments at the indicated time points. Scale bars, 500 µm. **(H)** Percentage of *med8*$^{+}$, *smed03831*$^{+}$, or co-expressing cells in the blastema shown in (G). ns, $p > 0.05$; $**p < 0.01$; two-tailed unpaired *t*-test. **(I)** Representative phenotypes following *med8* RNAi at 5 dpa. n = 10 animals for each condition. Scale bars: 500 µm. **(J)** Expression and localization of *smed03831* and *sfrp-1* in the blastema of control and *med8* RNAi animals at 1.5 (top left) and 3 dpa (top right). Scale bars: 500 µm. The ratio of *smed03831*- or *sfrp-1*-expressing cells in the blastema was quantified (bottom). ns, $p > 0.05$; $*p < 0.05$; $**p < 0.01$; two-tailed unpaired *t*-test. **(K)** FISH staining of *smed03831* in control and *med8* RNAi animals. n ≥ 3 animals per condition. Scale bars: 500 µm (top); 50 µm (bottom).

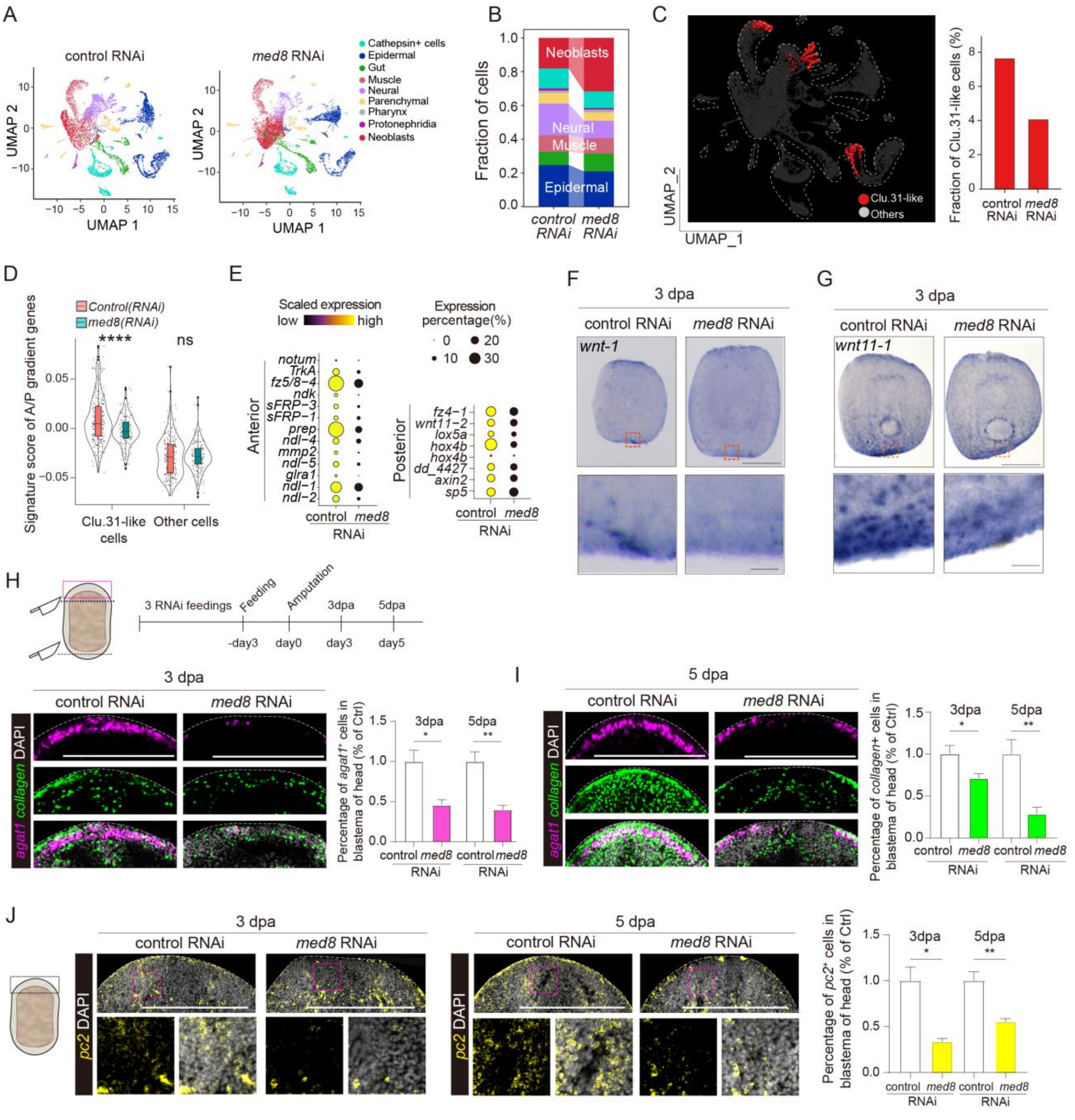
A
control RNAi
med8 RNAi
Cathepsin+ cells
Epidermal
Gut
Muscle
Neural
Parenchymal
Pharynx
Protonephridia
Neoblasts
UMAP 1
UMAP 2
B
Fraction of cells
Neoblasts
Neural
Muscle
Epidermal
control RNAi
med8 RNAi
C
Clu.31-like
Others
UMAP_1
UMAP_2
Fraction of Clu.31-like cells (%)
D
Control(RNAi)
med8(RNAi)
Signature score of A/P gradient genes
Clu.31-like cells
Other cells
E
Scaled expression
low
high
Expression percentage(%)
Anterior
Posterior
F
3 dpa
wnt-1
G
wnt11-1
H
3 RNAi feedings
Feeding
Amputation
3dpa
5dpa
-day3
day0
day3
day5
agat1 collagen DAPI
Percentage of agat1+ cells in blastema of head (% of Ctrl)
I
5 dpa
Percentage of collagen+ cells in blastema of head (% of Ctrl)
J
pc2 DAPI
Percentage of pc2+ cells in blastema of head (% of Ctrl)

1318

Figure 5: *Med8*-dependent regulation of anterior regenerative zone and blastema formation. **(A)** UMAP visualization of scRNA-seq analysis depicting cell lineages from control and *med8* RNAi-treated tail fragments. **(B)** Bar plot showing the changes in cell populations following *med8* RNAi knockdown. **(C)** Left: UMAP visualization of neural, muscular, and epidermal cells highlighting the ARZ signature (Clu.31-like) in scRNA-seq data. Right: Bar plot illustrating the decrease in the fraction of ARZ (Clu.31) cells in *med8* RNAi-treated animals compared to controls. **(D)** Violin plot showing changes in the module score of A/P gradient genes in ARZ (Clu.31) cells. p-values are from the Wilcoxon test: ns, $p > 0.05$; ****, $p < 0.0001$. **(E)** Dot plot illustrating the relative expression of representative PCGs in anterior or posterior regions from scRNA-seq data. **(F-G)** WISH analysis showing the expression and localization of posterior markers *wnt-1* (F) and *wnt11-1* (G) in control and *med8* knockdown animals. Scale bars: 500 µm (top), 50 µm (bottom). n = 6 animals with consistent results. **(H-I)** Expression and localization of muscle marker *collagen* and epidermal marker *agat-1* in the blastema of control and *med8* RNAi animals at 3 (H) and 5 days post-amputation (dpa) (I), respectively. Scale bars: 500 µm. n = 6 animals with consistent results. The percentage of positive cells in the blastema was quantified. ns, $p > 0.05$; *$p < 0.05$; **$p < 0.01$, two-tailed unpaired t-test. **(J)** Expression and localization of the neural marker *PC2* in the blastema of control and *med8* RNAi animals at 3 and 5dpa. Scale bars: 500 µm. The percentage of positive cells in the blastema was quantified (right). *$p < 0.05$; **$p < 0.01$, two-tailed unpaired t-test. All FISH images are maximum-intensity projections. Error bars represent SEM. $n \geq 3$ biologically independent experiments.

Figure 1

Click here to access/download;Figure;fig1-v2_white.png

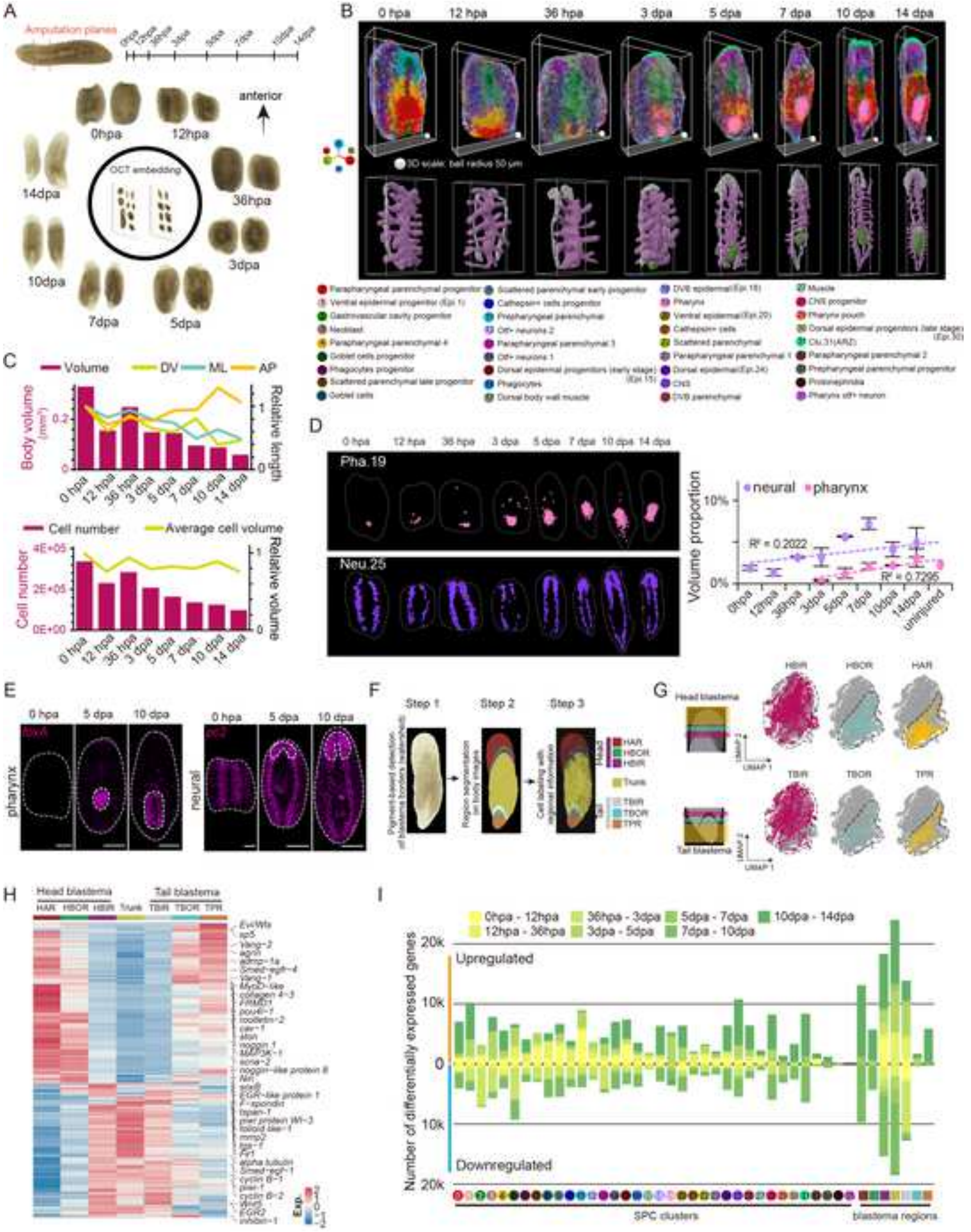

Figure 2

Click here to access/download;Figure;fig2_white.png

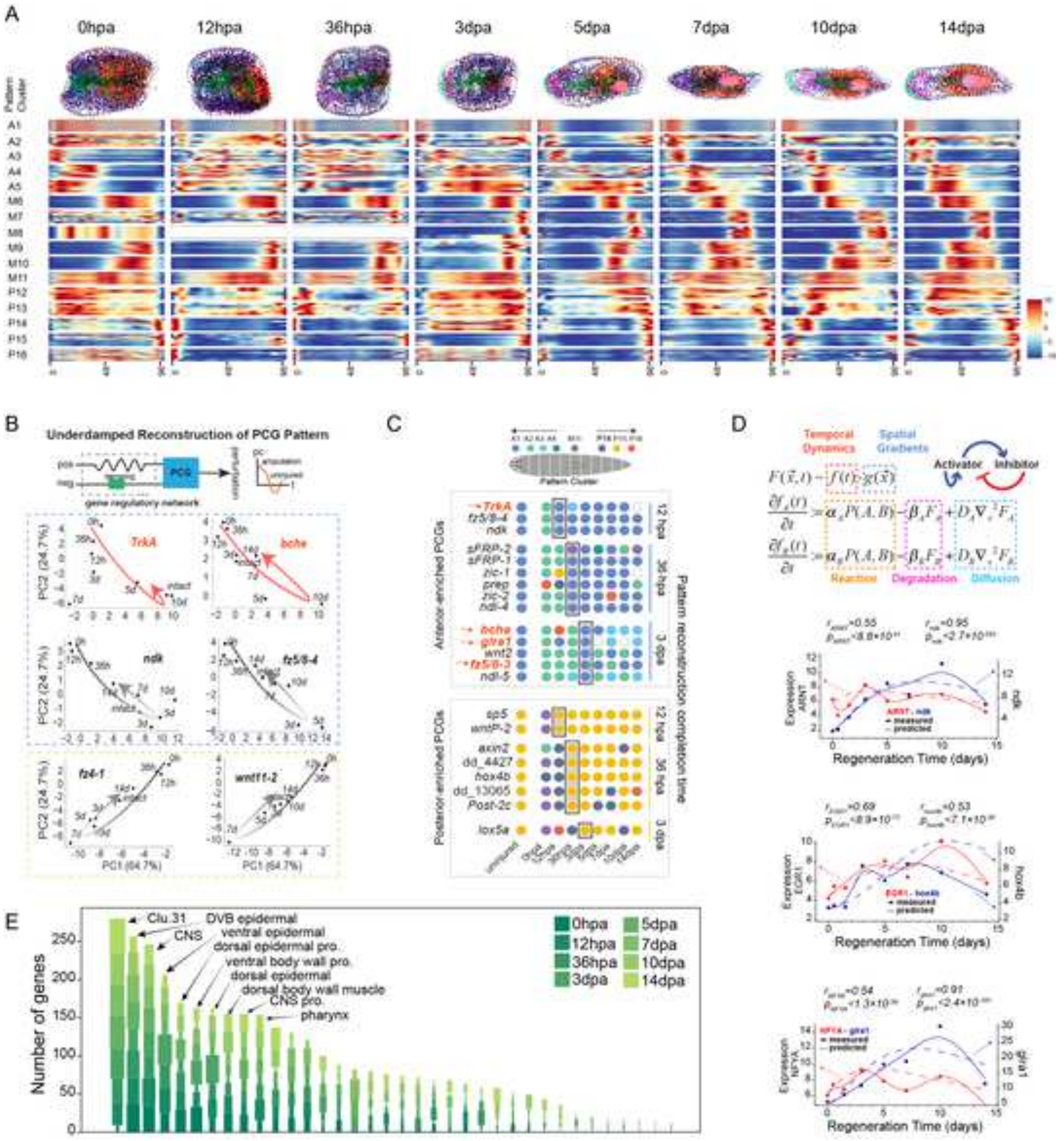

Figure 3

Click here to access/download;Figure;fig3_white.png

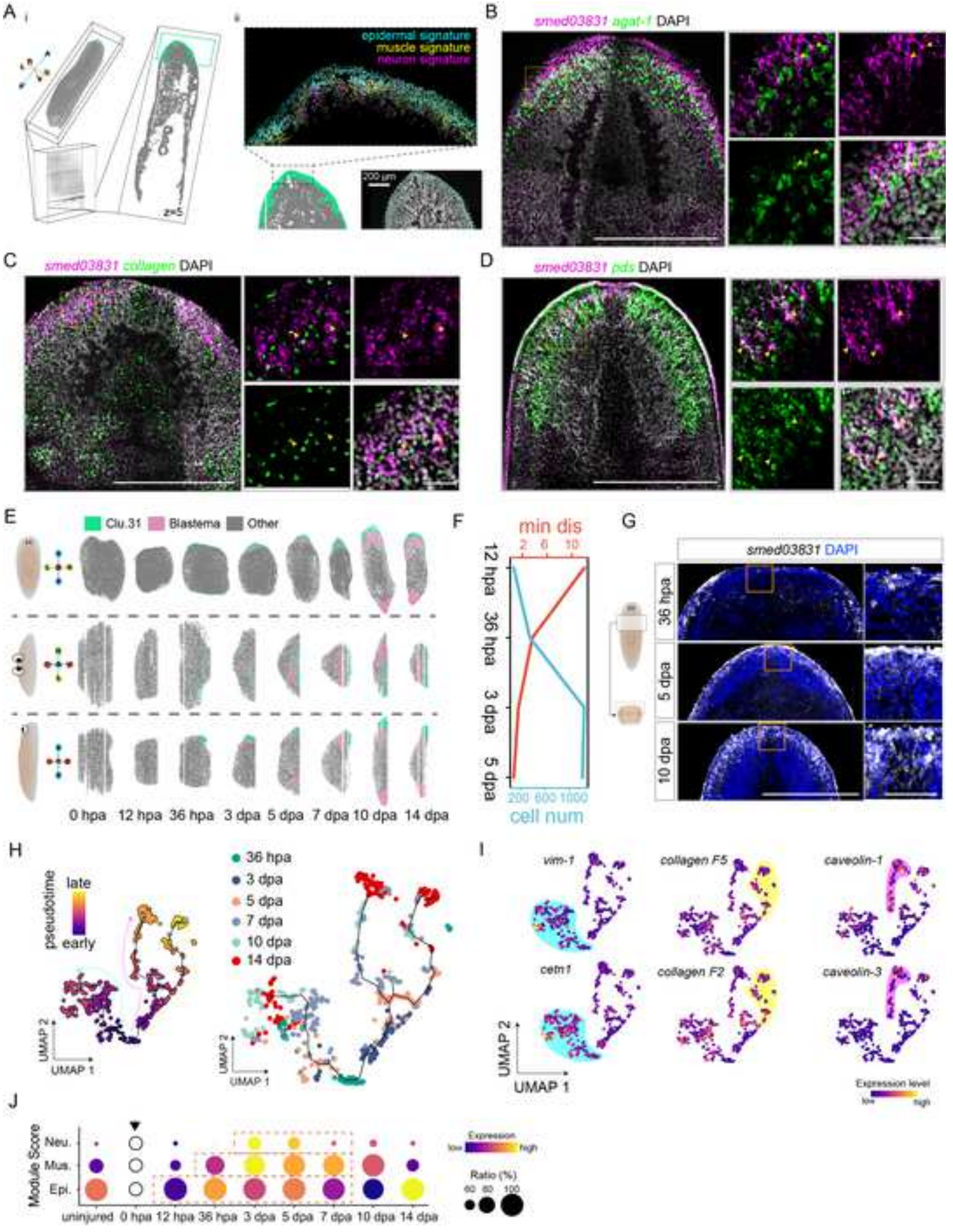

Figure 4

Click here to access/download;Figure;fig4-v2_white.png

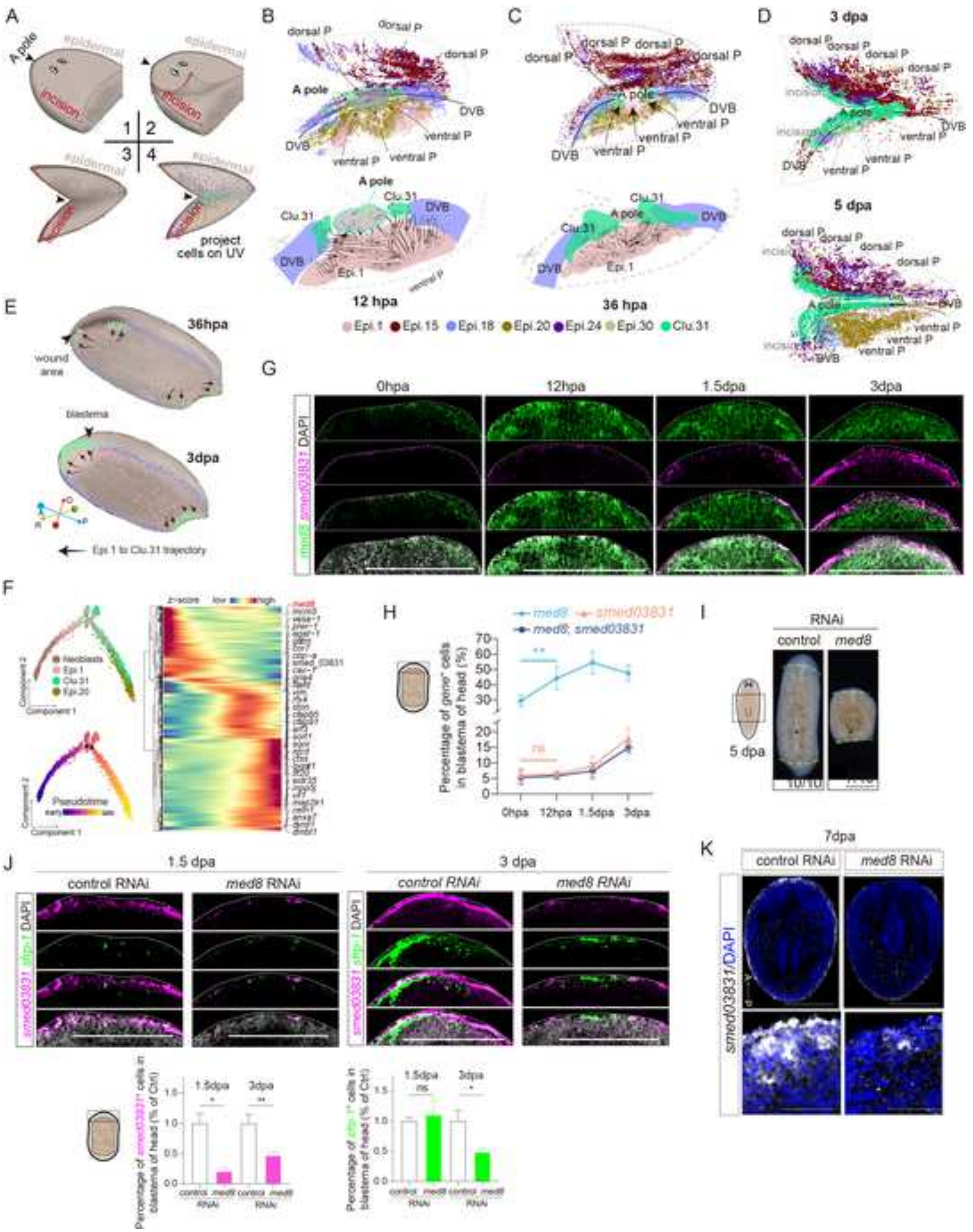

Figure 5

Click here to access/download;Figure;fig5-v2.png

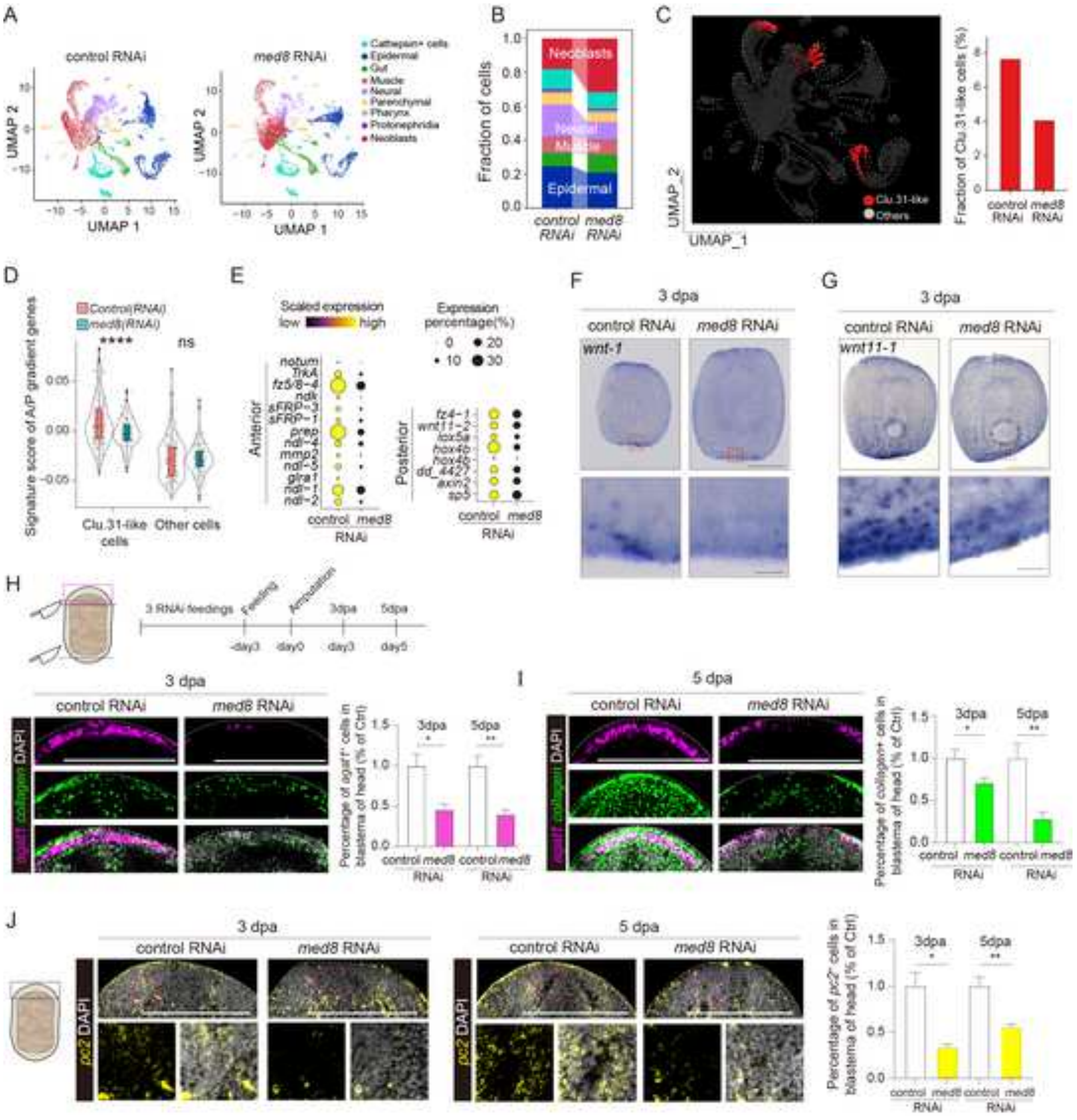

Point by point response with figures

Click here to access/download
Supplementary Material
PointbypointResponse-20260224.docx

Supplementary Figures

Click here to access/download
Supplementary Material
SupplementaryMaterial-20260224.docx

Supplementary Table S1

Click here to access/download
Supplementary Material
data S1.xlsx

Supplementary Table S2

Click here to access/download
Supplementary Material
data S2.xlsx

Supplementary Table S3

Click here to access/download
Supplementary Material
data S3.xlsx

Supplementary Table S4

Click here to access/download
Supplementary Material
data S4.xlsx

Supplementary Table S5

Click here to access/download

Supplementary Material

data S5.xlsx

Supplementary Table S6

Click here to access/download
Supplementary Material
data S6.xlsx

 Click here to access/download;Personal Cover;CoverLetter-20260224.docx

February 24th, 2026

Qingdao, China

Dear Dr. Dongni Ma,

Happy Chinese New Year 2026! We are submitting the revised manuscript titled “4D Single Cell Spatial Transcriptomics Reveals Dynamic Morphogenetic Gradients and Regenerative Domains in Planarians” (GIGA-D-25-00451) for consideration for publication in *GigaScience*. We express our deepest gratitude to the editorial team and the reviewers for their constructive and meticulous evaluation. The feedback has been instrumental in refining our analytical framework and strengthening our biological conclusions.

In accordance with the rigorous transparency standards of *GigaScience*, we have ensured complete compliance with data sharing and software registration policies. The core algorithmic framework previously cited as a submitted manuscript is now publicly accessible as a preprint on the *bioRxiv* server (DOI: 10.64898/2026.02.19.705280) to ensure full methodological transparency. We have registered our computational tools in the required databases, including bio.tools under the identifier 4d-bioreconx and SciCrunch under the identifier SCR_027919. Furthermore, all computational workflows have been deposited in WorkflowHub (DOI: 10.48546/workflowhub.workflow.2045.1). These identifiers are integrated into the Methods and Data Availability sections of the revised manuscript, facilitating tracking reproducibility and reuse by the scientific community.

Our revision strategy focused on four critical scientific enhancements. First, we introduced a formal spatial priming model to characterize the spatiotemporal lag between transcriptional initiation and anatomical patterning. Second, we provided multimodal validation confirming that the Anterior Regenerative Zone represents a biological reality of interwoven lineages rather than a technical artifact. Third, we

conducted extensive benchmarking against existing spatial technologies, highlighting the superior resolution and temporal continuity of our four-dimensional approach. Finally, we clarified the hierarchical role of Mediator 8 as a broad differentiation enabler rather than a direct instructor of polarity. In addition to the above response, we have further refined the language and redrew several figures to improve the overall writing quality and ensure that they effectively support our statements. We have also reformatted the manuscript and supplemental information to meet the requirements of technical information and advice.

We believe these revisions significantly elevate the manuscript and its impact on the field of regenerative biology. We eagerly await your response and are fully prepared to address any additional questions or comments from you or the reviewers.

Thank you once again for your time and favorable consideration.

Sincerely yours,

Mengyang Xu, Ph.D.
BGI Research
E-mail: xumengyang@genomics.cn
