# Supplementary material for: 4D single-cell spatial transcriptomics reveals dynamic morphogenetic gradients and regenerative domains in planarians
Source: Gigascience. 2026 May 22;15:giag064. doi: 10.1093/gigascience/giag064 (PMC13273413; doi:10.1093/gigascience/giag064)
Supplement: giag064_Authors_Response_To_Reviewer_Comments_original_submission [file giag064_Authors_Response_To_Reviewer_Comments_original_submission.pdf]

Author's Response To Reviewer Comments

Please note that all line numbers referenced in our Response to Reviewers correspond to the line numbering in the revised version of the manuscript.
Please also note that due to technical constraints of the online submission system, only text can be submitted, while figure uploads are not supported. Accordingly, all aforementioned figures are included in the Supplementary Materials file titled “Point-by-Point Response-20260224.docx.”

Response to Reviewer #1:
General Comments: The authors employs high-resolution Stereo-seq technology combined with multi-timepoint spatial transcriptomic data to construct a 4D spatiotemporal transcriptomic map of planarian regeneration. This work significantly advances the understanding of spatial gene expression dynamics during planarian regeneration, overcoming the limitations of traditional two-dimensional and planar spatial transcriptomics. Furthermore, the authors identify a novel injury-indced Anterior Regenerative Zone (ARZ) and, through functional validation of Mediator 8 (med8), deepen insights into the mechanisms underlying polarity remodeling in planarians. The study also provides an interactive online database, enriching spatial molecular and cellular data resources for the regenerative biology. The work is notably innovative, and the authors present convincing evidence supporting their conclusions. The manuscript overall is written well and data is presented clearly. The discussion and conclusions has done well to highlight the potential problems in this study. I have a few points that should be addressed before publishingI have a few points that should be addressed before publishing.

Response: We are deeply grateful to the reviewer for the highly positive assessment of our work and for recognizing the significance of our 4D spatiotemporal transcriptomic map in advancing the understanding of planarian regeneration. We particularly appreciate your acknowledgement of the technical innovation behind our high-resolution Stereo-seq approach, as well as the biological insights regarding the injury-induced ARZ and the regulatory role of med8 in polarity remodeling. We are also glad that the interactive online database was found to be a valuable resource for the community.
We sincerely thank you for your constructive comments and the specific points raised regarding the temporal dynamics of PCGs and the regulatory scope of med8. We have taken these suggestions very seriously and have performed additional analyses and revisions to address each point. We believe these changes have significantly strengthened the logical rigor and clarity of our manuscript. Please find our point-by-point responses below.

Comment 1: In lines 233-236, it is reported that the positional control gene (PCG) like ndk restores its spatial expression pattern as early as 12 hpa, whereas its expression level only significantly increases at 36 hpa. Given this pronounced temporal discordance between early recovery of spatial patterning and the later peak in mRNA levels, the authors should analyze and discuss possible molecular mechanisms that could account for this discrepancy, and consider the biological implications of this phenomenon for understanding how spatial information and gene-expression regulation are coordinated during regeneration.

Response: We thank the reviewer for highlighting this critical temporal feature. The observation that spatial patterning (e.g., ndk asymmetry at 12 hpa) is re-established well before the surge in transcriptional abundance (36 hpa) suggests a fundamental “Pattern-then-Amplify” regulatory logic. We have expanded our Discussion to interpret this phenomenon through three synergistic mechanisms, supported by additional literature analysis:
1. Retention of Positional Memory in Existing Tissue
Planarian PCGs are predominantly expressed in body-wall muscle cells, which harbor positional information (Witchley, J. N., et al. (2013). Muscle cells provide instructions for planarian regeneration. Cell Reports, 4(4), 633–641.). Following amputation, the early recovery of spatial patterns at 12 hpa likely represents the rapid repolarization or maintenance of identity in pre-existing muscle cells at the wound boundary, rather than de novo pattern formation in new tissue. As previously demonstrated, planarians utilize landmarks in existing tissue to define the coordinates for regeneration immediately after injury (Oderberg,

I. M., et al. (2017). Landmarks in existing tissue at wounds are utilized to generate pattern in regenerating tissue. Current Biology, 27(5), 733–742.). Thus, the organism re-establishes the map using the surviving tissue scaffold before constructing the blastema.
2. Early Signaling Precedes Transcriptional Bursts
The initial definition of head and tail spatial domains is driven by rapid signaling cascades (e.g., Wnt, MAPK) and post-translational modulators that operate prior to large-scale gene expression changes. For instance, it recently demonstrated that the phosphatase DjPtpn11 modulates early wound response genes and Wnt signaling dynamics shortly after injury, acting as an essential starter for the regenerative program (Pang, Q., et al. (2022). DjPtpn11 is an essential modulator of planarian (Dugesia japonica) regeneration. International Journal of Biological Macromolecules, 209, 1054–1064.). This signaling-primed state allows for the precise spatial restriction of PCG expression pattern at low basal levels before the system ramps up production.
3. Decoupling for Regenerative Fidelity
The lag between pattern establishment (12 hpa) and the transcriptional peak (36 hpa) serves as a fidelity checkpoint. The massive upregulation at 36 hpa coincides with the onset of blastema formation and neoblast proliferation. By decoupling these phases, the organism ensures that the proliferation machinery is only fully engaged after the spatial coordinate system has been correctly verified. This hierarchical strategy prevents maladaptive growth and ensures that the new tissue integrates seamlessly with the old. We have revised the Discussion section to articulate this "Pattern-then-Amplify" model, integrating these citations to provide a robust molecular explanation for the observed spatiotemporal dynamics (Lines 496-508).

Comment 2: In lines 356-360, Med8 knockdown markedly reduces the ARZ cell lineages and the expression of anterior-posterior polarity markers (e.g., sfrp-1, wnt1, wnt11-1), producing a clear effect on regeneration polarity formation.However no gross disruption of the whole-body AP axis was observed. Please further analyze and discuss the possible regulatory scope and mechanisms of Med8. Specifically, do other redundant pathways or compensatory mechanisms exist in planarians that maintain global positional information despite loss of Med8? What is the hierarchical and cell-type specificity of Med8's role in polarity regulation?

Response: We thank the reviewer for identifying this apparent paradox. The observation that med8 RNAi significantly dampens polarity markers without causing a gross axis duplication (e.g., double-head or double-tail) provides critical insight into its hierarchical role. We have expanded our analysis and Discussion to clarify that med8 functions as a differentiation-enabler for polarity-organizing cells, rather than a direct instructor of the body axis gradient itself.
1. Lineage-Dependent Polarity Expression Hypothesis
Our scRNA-seq data demonstrate that med8 is essential for the differentiation of specific ARZ lineages, particularly epidermal, muscle, and neural cells. In planarians, positional information is generated and maintained by these specialized differentiated cells. Therefore, the observed reduction in polarity markers (wnt1, sfrp-1) is a secondary consequence of the failure to generate the signal-producing cells, rather than a direct transcriptional silencing of polarity genes in the pre-existing tissue. Unlike instructional genes (e.g., β-catenin), whose perturbation directly flips the binary switch of axial identity, med8 depletion leads to a loss of voice for the organizer, resulting in regenerative failure rather than ectopic patterning (Lines 395-405).
2. Symmetric Signal Attenuation Preserves the Gradient Profile
Crucially, our data show that med8 knockdown reduces both anterior (sfrp-1) and posterior (wnt1, wnt11-1) markers simultaneously. This symmetric reduction maintains the relative antagonism between Wnt and anti-Wnt signals, preventing the dominance of one pole over the other. In our case, the global dampening of the gradient amplitude halts blastema formation but preserves the gross body plan. (Lines 406-416)
3. Temporal Dynamics
While gross axis disruption was absent in the short regeneration window likely due to the stability of the extracellular matrix and long-lived muscle cells in the stump, we observed that prolonged med8 RNAi leads to head regression in homeostatic animals. This confirms that med8 is eventually required for maintaining global positional information by ensuring the continuous turnover of polarity-specifying cells. (Lines 434-437)
4. Subunit Specificity and Functional Redundancy

The Mediator complex is modular. Previous studies indicate that different subunits govern distinct biological outputs; for instance, Smed-med14 is required for stem cell maintenance, whereas med8 specifically directs differentiation. This suggests that while med8 is critical for the differentiation-coupled polarity maintenance, other Mediator subunits or parallel signaling pathways may compensate to maintain basal transcript levels in surviving tissue, preventing immediate axis collapse. (Lines 532-538)
We have revised the Discussion to incorporate these points, clarifying the hierarchical distinction between med8 (differentiation of the organizer) and canonical PCGs (instruction of the axis) (Lines 544-557).

Response to Reviewer #2:
General Comments: In the manuscript '4D single-cell spatial transcriptomics reveals dynamic morphogenetic gradients and regenerative domains in planarians,' Han and colleagues generate a truly stunning spatial transcriptomics dataset of planarian regeneration from the species Schmidtea mediterranea. The authors' dataset includes whole 3D reconstructions of two regenerating planarian fragments at 8 different timepoints during regeneration, a fantastic accomplishment and resource of broad interest to the regenerative biology community. The authors analysis of the dataset includes characterization of spatially biased genes (SBGs) and exploration of an anterior regenerative zone (ARZ) and the role of the gene med8 in its' regulation. While the authors' dataset is remarkable and their analysis of spatially biased genes and med8 function is interesting, I'm not yet convinced that their conclusions are fully tested by the included experiments. In addition, I think that the authors have not included sufficient quality control metrics for their spatial dataset, which makes determining the limitations or caveats of their analysis and conclusions more difficult. However, my concerns could be addressed by additional analysis and minor experiments, or by softening the conclusions of the authors to include alternative models. I've detailed the areas of analysis/discussion that I believe require improvement below:

Response: We sincerely thank Reviewer #2 for their highly encouraging evaluation of our work and for recognizing our 4D spatial transcriptomics dataset as a stunning and fantastic accomplishment that will serve as a valuable resource for the regenerative biology community. We truly appreciate the time and effort dedicated to reviewing our manuscript.
We find your constructive criticism regarding the interpretation of our conclusions, particularly concerning med8 function and spatially biased genes, and the need for more rigorous quality control metrics to be extremely insightful. We have taken these comments very seriously and have performed additional analyses and text revisions to address each point raised. We believe these improvements have significantly strengthened the manuscript, and we provide a detailed point-by-point response to your specific concerns below.

Major Criticisms:
Comment 1: Stereo-seq resolution and capture efficiency: The authors assert that their spatial approach is high enough resolution to resolve cell types and they claim to have characterized 36 cell types in their abstract. However, the 'cell type' in their dataset that they choose to focus on - Clu.31 - has gene markers expressed in three different cell types that have been shown to be distinct in the literature and prior planarian atlases. The authors should analyze gene expression signatures of other stereo-seq 'cell types' to determine if they also show mixed expression signatures. In addition, I am curious if stereo-seq is more likely to capture highly expressed genes (like those expressed in parenchymal cell types) than more lowly expressed genes (like the transcription factors expressed in stem cells). If it exists, this bias could influence annotation of cell types in highly heterogeneous regions of the worm like the parenchyma or parapharyngeal region. Finally, there is very little QC data in the supplementary materials (Size/volume of segmented cells, UMIs and features per cell, variability in features/UMIs per section, per replicate, and per cell type, etc.) I think this analysis would be highly valuable for the reader to interpret the data and the 36 identified 'cell types'.

Response: We thank the reviewer for this critical assessment of our spatial terminology and quality control metrics. We acknowledge that the term “cell type” was applied too broadly in our initial submission. While our high-resolution Stereo-seq approach (715 nm) is capable of resolving single cells, we agree that the clusters identified via Spatial Proximity Clustering (SPC) technically represent spatial proximity domains, collections of cells with similar gene expression profiles that may include both monophyletic cell types and

heterogeneous cellular neighborhoods. We have revised the manuscript to adopt the term “domains” to more accurately reflect the nature of these spatial units.
Regarding the mixed signature of the Anterior Regenerative Zone (ARZ/Clu.31), we maintain that this is a verified biological feature rather than a technical artifact. The blastema is a region of high cellular density and extensive intercellular communication. Consequently, Clu.31 captures a functional regenerative niche where epidermal, muscle, and neural lineages physically converge. This interpretation is supported by our high-resolution in situ hybridization data (Fig. 3B-D), which confirms that smed03831-positive cells are physically intermingled with these distinct lineages. This multi-lineage convergence zone is reminiscent of the specialized microenvironments observed in other regeneration models.
To address the reviewer’s concern regarding the purity of the other 35 clusters, we performed a comprehensive correlation analysis comparing our Stereo-seq gene signatures with three published single-cell RNA-seq atlases (Emili, E., Pérez-Posada, A., Vanni, V., Salamanca-Díaz, D., & Solana, J. (2025). Allometry of cell types in planarians by single-cell transcriptomics. Science Advances, 11(19), eadm7042. Plass, M., Solana, J., Wolf, F. A., Ayoub, S., Misios, A., Glažar, P., Obermayer, B., Theis, F. J., Kocks, C., & Rajewsky, N. (2018). Cell type atlas and lineage tree of a whole complex animal by single-cell transcriptomics. Science, 360(6391), eaaq1723. Fincher, C. T., Wurtzel, O., de Hoog, T., Kravarik, K. M., & Reddien, P. W. (2018). Cell type transcriptome atlas for the planarian Schmidtea mediterranea. Science, 360(6391), eaaq1736.) and a 10x Genomics Visium spatial dataset (Cui, G., Dong, K., Zhou, J.-Y., Li, S., Wu, Y., Han, Q., Yao, B., Shen, Q., Zhao, Y.-L., Yang, Y., Cai, J., Zhang, S., & Yang, Y.-G. (2023). Spatiotemporal transcriptomic atlas reveals the dynamic characteristics and key regulators of planarian regeneration. Nature Communications, 14(1), 3205.). This analysis, presented in the new Supplementary Fig. S2 (referred to as Fig. R1 here), reveals the cellular composition of our domains (Lines 149-153, 682-687):
1. High-Fidelity Cell Types
Clusters 12 and 14 exhibit high-quality, one-to-one mappings with specific single-cell subtypes, confirming they represent distinct cell populations.
2. Lineage-Restricted Domains
The majority of domains are lineage-specific. For example, clusters 2, 5, 6, 8, 10, 13, 16, and 21 map exclusively to the Gut lineage; clusters 19, 29, and 35 map to the Pharynx; clusters 25 and 28 map to the Neural lineage; and cluster 17 maps to Muscle. These domains show minimal cross-contamination from other germ layers.
3. Stem Cell and Parenchymal Niches
Cluster 3 maps cleanly to the neoblast subtype. However, clusters 7, 9, 11, 22, and to a lesser extent 0, 4, 23, 26, and 33, exhibit signatures of both neoblasts and parenchymal cells. This finding aligns with recent single-cell studies describing the heterogeneity of stem cell microenvironments and the presence of transient post-mitotic states within the parenchyma (Benham-Pyle, B. W., Brewster, C. E., Kent, A. M., Mann, F. G., Chen, S., Scott, A. R., ... & Sánchez Alvarado, A. (2021). Identification of rare, transient post-mitotic cell states that are induced by injury and required for whole-body regeneration in Schmidtea mediterranea. Nature Cell Biology, 23(9), 939–952.).
Figure R1: Correlation analysis of Stereo-seq gene expression signatures with published transcriptomic datasets. (A-C) Heatmaps displaying the Pearson correlation coefficients between the gene expression profiles of Stereo-seq clusters (columns) and annotated cell types from published single-cell RNA-seq datasets (A: Elena Emili et al., Science Advances, 2025; B: Mireya Plass et al., Science, 2018; C: Christopher T. Fincher et al., Science, 2018). (D) Similarity comparison between Stereo-seq clusters and spatial domains defined in previously published 10x Genomics Visium datasets (Cui, G., Dong, K., Zhou, J.-Y., Li, S., Wu, Y., Han, Q., Yao, B., Shen, Q., Zhao, Y.-L., Yang, Y., Cai, J., Zhang, S., & Yang, Y.-G. (2023). Spatiotemporal transcriptomic atlas reveals the dynamic characteristics and key regulators of planarian regeneration. Nature Communications, 14(1), 3205.). The color scale represents the Pearson correlation coefficient, with red indicating higher similarity between the corresponding groups.

4. The Unique ARZ
Consistent with our hypothesis, Clu.31 remains unique in its distinct tri-lineage enrichment (epidermal, neural, muscle), distinguishing it as a complex signaling center.
Regarding capture efficiency and bias, we acknowledge the inherent sparsity of nanoball-based spatial transcriptomics (random sparse capture). However, to test for systematic bias against lowly expressed genes (e.g., transcription factors), we calculated the correlation between our aggregated Stereo-seq “pseudo-bulk” data and published bulk RNA-seq datasets (Roberts-Galbraith, R. H., Brubacher, J. L., &

Newmark, P. A. (2016). A functional genomics screen in planarians reveals regulators of whole-brain regeneration. eLife, 5, e17002.). We observed a high Pearson correlation ($R \geq 0.8$), suggesting that the sparsity is random and does not disproportionately exclude low-abundance regulatory genes essential for defining cell identity.
Finally, we have significantly expanded the Quality Control metrics in the Supplementary Materials as requested. We updated Data S1 to include median Gene counts (150–230), UMI counts (190–370), and segmented cell areas (70–120 pixels) per section and per cluster. These metrics demonstrate high consistency across replicates and sections, ensuring that biological interpretations are not driven by technical variability.

Comment 2: Dynamics of spatially biased genes: The authors analysis on the dynamics of spatially biased genes (SBGs) is very interesting, but the 'oscillations' the authors referred to were not clear to me in the data across all or even most of the pattern clusters in Figure 2A. In general, it seemed more like the pattern cluster was 'noisy' or more broad before stabilizing to its final location. In addition, the PCA analysis in Figure 2B seems to show that Intact and 14dpa transcriptomics is very similar, but 0h, 12h, and 36h timepoints are very distinct from 3, 5, 7, and 10 day fragments. This would suggest that early wound response gene expression is highly distinct (even opposing) the gene expression programs active during late in regeneration. More exploration of this idea, as well as clarified language on exactly what the author means by 'oscillations' and which gene groups follow this pattern would greatly improve this section and better support the author's conclusions.

Response: We thank the reviewer for this insightful observation, particularly regarding the interpretation of the PCA trajectories and the terminology used to describe gene expression dynamics. We agree that the term “oscillation” carries a connotation of periodic sinusoidal behavior, which does not accurately reflect the biological reality of a system returning to homeostasis following severe structural perturbation. To address this, we have revised our terminology from “oscillations” to “underdamped dynamic responses”. We have adopted a systems biology perspective, referencing control theory and physical mechanics to better describe this process (Inman, D. J. (2007). Engineering Vibration (3rd ed.). Pearson Education, Inc. MacArthur, B. D., Ma'ayan, A., & Lemischka, I. R. (2009). Systems biology of stem cell fate and cellular reprogramming. Nature Reviews Molecular Cell Biology, 10(10), 672–681.). In this framework, we model the regenerating planarian as a dynamic system where equilibrium represents the intact, homeostatic state; perturbation represents the amputation event, which displaces the system far from equilibrium; and restoring force represents the GRN that drives the system back toward the homeostatic attractor. Under this model, the noisy broad patterns observed in Fig. 2A represent the system’s transient response, an initial overshoot and subsequent convergence, as it searches for and stabilizes at the new equilibrium.
Furthermore, we agree with your interpretation of the PCA data in Fig. 2B. The distinct separation of time points indeed reflects a phase transition in the biological program:
1. Acute Injury Phase (0h–36h)
The system is in a high-energy, non-equilibrium state dominated by wound healing and immediate injury responses, distinct from both the regenerative and homeostatic states. This aligns with the identification of transient, injury-induced cell states described in recent literature.
2. Patterning & Morphogenesis Phase (3d–10d)
The system begins to converge, dominated by the re-establishment of positional information and tissue differentiation.
3. Homeostatic Return (14dpa)
The system returns to a state statistically similar to the intact control.
We have revised the manuscript to explicitly discuss these distinct phases and have removed the confusing references to “oscillations” in favor of this more rigorous physical systems model (Lines 247-264, 467-473).

Comment 3: The Cellular/Functional identity of Clu.31: The authors state throughout the manuscript that Clu.31 (the ARZ) is an injury-induced anterior state enriched for SBGs and regulating polarity establishment. However, it is also possible that this spatial state represents the anterior peripheral nervous system (numerous sensory neurons and surface epithelial cells that help sense mechanical and chemical cues). SBGs could be enriched because this combination of cell types is only present in the anterior of the

animal. Indeed, the authors show that the ARZ is localized to the anterior in intact animals in the absence of an injury (Figure 3) and enriched genes (S4Aii) strongly indicate that Clu.31 contains gabrg+ mechanosensory neurons. If Clu.31 is regenerating nervous system, this would also explain its ventral bias and expression of tgs-1 and other nb2 genes, since nb2 neoblasts have been suggested to be both an amputation responsive neoblast subset (Zeng et al. Cell) and a neural progenitor state (Raz et al. Cell Stem Cell). Clarifying how the composition of the tri-lineage region changes during regeneration may help distinguish if Clu.31 is truly an injury induced region vs. the regenerating sensory nervous system. For example, it is known that agat-1+ cells transcriptionally responsive and enriched at the wound site a 2-4 days post amputation, but less so at later timepoints (Benham-Pyle et al Nature Cell Biology, Kent et al. Developmental Biology). This shift in composition should be observable in Clu.31 since it contains agat+ epidermal cells. Such a shift in composition or the identification of a regeneration-specific marker expressed in Clu.31 would add support to the author's conclusions. Regardless of the outcome of these experiments/analyses, the discussion and interpretation of the data could be modified to address the hypothesis that Clu.31 represents the cellular neighborhood created when the peripheral nervous system intercalates with the anterior DV boundary epithelium and body wall muscle, which needs to be regenerated in amputated worms. As is, the comparison to the apical epithelial cap considered in the discussion (Line 438) may be pre-mature.

Response: We thank the reviewer for this insightful alternative interpretation. We agree that the cellular composition of Clu.31 rich in sensory neurons and epithelial cells shares significant features with the anterior peripheral nervous system. We appreciate the suggestion to consider this structural identity and have carefully re-analyzed our data to address this point. While Clu.31 contains components of the regenerating nervous system, we respectfully propose that it functions as a dynamic regenerative organizing center rather than solely representing the regenerating anterior structures. This conclusion is supported by three key lines of evidence from our 4D dataset:
1. Transient Appearance in the Tail Blastema (Fig. 3E; Supplementary Fig. S2F)
Crucially, the Clu.31 signature emerges transiently in both anterior and posterior wound sites (head and tail blastemas) at 36 hpa. In tail fragments, which are regenerating a tail and not an anterior brain, this signal appears and then recedes. If Clu.31 were simply the anterior PNS, its specific molecular signature would not be expected to appear in the posterior blastema. This "appear-and-restrict" pattern suggests a role in global polarity assessment.
2. Dynamic Compositional Shifts (Fig. 3H-J)
As suggested, we examined the temporal changes in cell composition. The composition of Clu.31 is not static or linearly increasing (as might be expected for simple tissue growth) but is highly dynamic: initiating with a predominant epidermal signature (12 hpa), recruiting muscle and neural lineages during blastema formation (3 dpa), and eventually reverting to a homeostatic state.
3. Distinct Expression Dynamics of agat-1 (New)
Following the reviewer's specific suggestion regarding agat-1+ cells, we plotted the temporal expression of agat-1 within the Clu.31 cluster (Fig. R2). The results strikingly confirm the reviewer's prediction of a compositional shift:
1) Injury Response (36 hpa – 5 dpa): agat-1 expression is widespread and high, consistent with the recruitment of transient, injury-responsive epidermal states (Benham-Pyle, B. W., Brewster, C. E., Kent, A. M., Mann, F. G., Chen, S., Scott, A. R., ... & Sánchez Alvarado, A. (2021). Identification of rare, transient post-mitotic cell states that are induced by injury and required for whole-body regeneration in Schmidtea mediterranea. Nature Cell Biology, 23(9), 939–952.).
2) Remodeling Dip (7 dpa – 10 dpa): Crucially, we observe a significant reduction in agat-1 expression and cell percentage during the mid-to-late regeneration phase, before recovering in homeostasis. This non-linear fluctuation demonstrates that the ARZ undergoes specific compositional remodeling distinct from monotonic tissue growth.

Figure R2: Temporal expression dynamics of agat-1 in the Clu.31 cluster. Note that the distinct high levels during early regeneration (36hpa-5dpa) followed by a reduction at 7-10dpa, indicating a dynamic state shift.

In light of these findings, we have revised the Discussion section: 1) acknowledge that Clu.31 physically represents a cellular neighborhood comprising mechanosensory neurons, epithelium, and muscle; 2) cite relevant literature on transient cell states (Benham-Pyle, B. W., Brewster, C. E., Kent, A. M., Mann, F. G.,

Chen, S., Scott, A. R., ... & Sánchez Alvarado, A. (2021). Identification of rare, transient post-mitotic cell states that are induced by injury and required for whole-body regeneration in Schmidtea mediterranea. Nature Cell Biology, 23(9), 939–952. Kent, A. M., Guerrero-Hernández, C., Brewster, C., McKinney, S., Morrison, J. A., McKinney, M. C., Ross, E. J., Mann, F. G., Jr, Benham-Pyle, B. W., & Sánchez Alvarado, A. (2026). Metabolites produced by agat+ cells support regeneration in the planarian Schmidtea mediterranea. Developmental Biology, 529, 106–120.) to contextualize the dynamic compositional shifts we observed; and 3) soften the comparison to the vertebrate Apical Epithelial Cap (AEC), describing it as functionally reminiscent rather than implying direct homology (Lines 509-528).

Comment 4: Med8 function: Med8 produces a clear phenotype in the authors' experiments, and their data indicates that it is required for ARZ formation. However, I am not sure that the authors data supports the claim that Med8 is directly regulating blastema and PCG expression, as opposed to regeneration of the nervous system (which is highly interconnected with formation of the anterior pole and the size of the anterior blastema) and stem cell function more broadly. The fact that Med8 RNAi also leads to head degeneration in intact worms (Figure S6F) strongly suggests a more fundamental defect in neural differentiation or stem cell function. The strongest evidence presented by the authors supporting a broader function in polarity establishment is the disruption of posterior Wnt expression, (Figure 5F and G), but these in situs are single representative images with no quantitation and could also be explained by a stem cell defect. Additional data could be provided (e.g. visualization of wound-induced gene expression, quantitation of anterior or posterior stem cell numbers and proliferation rates at 2dpa) to support regulation of PCGs or blastema formation. The authors could also leverage their single cell sequencing to determine if Med8 RNAi impacts neural progenitor abundance more than other progenitor cell types. Together, these experiments would determine if Med8 is important for amputation-induced blastema formation and polarity re-establishment vs. stem cell function and neural differentiation more broadly.

Response: We sincerely thank the reviewer for this insightful comment. We fully agree with the reviewer's interpretation that med8 is likely a fundamental regulator of stem cell function, rather than a direct regulator of polarity signaling. We acknowledge that our original description may have implied a direct regulatory role, which was not our intention. We have revised the manuscript to clarify the hierarchical mechanism: med8 is required for the formation of the ARZ, and the ARZ subsequently acts as the organizer to establish polarity. Thus, the polarity defect is a secondary consequence of the failure to form the ARZ.
The reviewer asked whether the phenotype is driven specifically by a failure in neural regeneration, which then affects the anterior pole. To test this, we followed the reviewer's suggestion to analyze whether med8 RNAi impacts neural progenitor abundance more than other progenitor types. Our scRNA-seq analysis (Fig. R3) reveals that the defect is not neural-specific, but rather a broad suppression of lineage commitment. When we normalized progenitor counts to the size of the neoblast pool (calculating the Differentiation Efficiency), we found a consistent collapse across all major lineages:
1. Neural Differentiation: Efficiency reduced by 66.9%.
2. Muscle Differentiation: Efficiency reduced by 45.5%.
3. Epidermal Differentiation: Efficiency reduced by 45.9%.
The comparable reduction in muscle and epidermal progenitors demonstrates that med8 is not a neural-specific regulator. Since muscle cells are the cellular source of positional information in planarians, the differentiation blockade in the muscle lineage provides a sufficient mechanistic explanation for the polarity defect, independent of the neural phenotype.

Figure R3: Med8 RNAi causes a broad reduction in differentiation efficiency across ARZ lineages.

We also agree with the reviewer that the head regression phenotype points to a fundamental defect in stem cell function. However, our data allows us to be more specific about which function is impaired. In med8 (RNAi) animals, the absolute number of undifferentiated neoblasts (piwi-1+/lineage-negative) actually increased (accumulated) compared to controls, indicating that stem cell maintenance and self-renewal capabilities are preserved. The combination of "neoblast accumulation" and "progenitor depletion" defines the defect as a Differentiation Blockade. Stem cells are maintained but functionally fail to exit the pluripotent state to generate differentiated progeny.

We have rewritten the relevant Results and Discussion sections to adopt this refined model. We no longer claim med8 directly regulates PCGs. Instead, we propose that med8 is required for the broad differentiation output of stem cells, including the muscle and neural lineages that constitute the ARZ. The loss of these signaling centers subsequently leads to the observed polarity collapse.

Minor Criticism/Feedback:
Comment 5: In Figure 1I, the authors show DEGs enriched in each cluster/region. In the blastema regions, I was surprised by the number of DEGs for each time point. It appears that there are ~10K upregulated and 10K downregulated DEGs by the later time points, which suggests that 2/3 of the transcriptome is differentially expressed… The authors should clarify in the text or methods what cutoff they used for the DEGs and how significant the DEGs are in this figure.

Response: We thank the reviewer for this careful observation. We apologize for the omission of specific methodological details regarding the algorithms and cutoffs used for differential expression analysis in the original manuscript. To clarify, the DEGs presented in Fig. 1I were identified using DEsingle, an R package specifically designed to handle the zero-inflation characteristic inherent to single-cell and spatial transcriptomics data. Our analysis strategy involved pairwise comparisons between adjacent time points (e.g., 0 hpa vs. 12 hpa, 12 hpa vs. 36 hpa) for each cluster and blastema region to capture dynamic temporal transitions. We applied a stringent statistical cutoff of False Discovery Rate (FDR) < 0.05 (Benjamini-Hochberg adjusted p-value) to define significance.
The relatively high number of DEGs observed is attributable to two main factors. First, DEsingle is more sensitive than traditional methods; it detects not only changes in mean expression (Type I) but also changes in the proportion of expressing cells (Type II) and composite changes (Type III). This allows us to capture subtle but biologically significant shifts in cell activation states that standard fold-change cutoffs might miss. Second, whole-body regeneration involves profound transcriptomic remodeling, particularly in the blastema, where cells undergo rapid dedifferentiation and re-specification. We have expanded the Methods section to explicitly describe this methodology (Lines 699-709).

Comment 6: For readability, I really think that all figures should be on a white background.
Response: We appreciate the reviewer's suggestion regarding figure readability. We have adjusted the background to white for the majority of the figures to ensure clarity and standard presentation. However, for certain high-density spatial transcriptomic maps (specifically the in situ cell type visualizations), we retained a dark background. This is a standard practice in spatial transcriptomics visualization to ensure sufficient contrast for distinguishing multiple fluorophores or cluster colors, which would otherwise be indiscernible against a white background.

Comment 7: How do gene expression profiles from the stereo-seq compare to bulk rnaseq at similar timepoints?

Response: We thank the reviewer for suggesting this crucial technical validation. To rigorously assess the quantitative accuracy of our Stereo-seq data, we compared our spatial transcriptomic profiles with high-quality published bulk RNA-seq datasets derived from homeostatic planarians (Roberts-Galbraith, R. H., Brubacher, J. L., & Newmark, P. A. (2016). A functional genomics screen in planarians reveals regulators of whole-brain regeneration. eLife, 5, e17002.).
We retrieved raw sequencing data for three biological replicates of intact animals (Accession numbers: SRR3465425, SRR3465432, SRR3465433) from the Sequence Read Archive (SRA). Reads were aligned to the S. mediterranea genome (dd_Smes_G4 assembly) using HISAT2, and gene counts were quantified using featureCounts, ensuring the reference genome and pipeline matched our Stereo-seq analysis. From our Stereo-seq dataset, we aggregated all captured UMIs from the homeostatic individual to construct a pseudo-bulk expression profile. Both datasets were normalized to Counts Per Million (CPM) and log2-transformed to allow for direct comparison. As shown in Fig. R2 (and added to Supplementary Fig. S1C in the revised manuscript), the Stereo-seq pseudo-bulk profile exhibits a high correlation with all three bulk RNA-seq replicates, with Pearson correlation coefficients (R) ranging from 0.80 to 0.81. This strong concordance confirms that our Stereo-seq platform captures the global transcriptome with high sensitivity

and quantitative reliability, comparable to standard bulk sequencing methods.

Figure R4: Correlation analysis between Stereo-seq pseudo-bulk data (Homeostasis) and three independent bulk RNA-seq replicates (Roberts-Galbraith et al., 2016). The Pearson correlation coefficient (R) is indicated for each replicate.

Furthermore, we have included this correlation analysis in Supplementary Fig. S1C and added a description of this validation step in the Methods section under “Data Quality Control and Validation”.

Comment 8: It is very interesting that there are some cell types that appear to contract and then expand during regeneration (Cluster 0, 23) or that aggregate/become more targeted during regeneration (pharynx pouch, cluster 29). Molecular differences between early and late cells within these cell types would be particularly interesting for understanding different phases of regeneration, but this may be beyond the scope of the current study.

Response: We appreciate the reviewer’s interest in the molecular dynamics of these specific cell populations. We fully agree that characterizing the molecular differences between early and late stages within these dynamic clusters (e.g., Clusters 0, 23, and 29) is critical for understanding their regenerative behaviors. In fact, we have already performed this systematic analysis in our original manuscript. As summarized in Fig. 1I, we calculated the DEGs for each cluster including Clusters 0, 23, and 29 and blastema regions between adjacent regeneration timepoints (e.g., 0hpa vs. 12hpa, 12hpa vs. 36hpa, etc.). The detailed results of this analysis are provided in Supplementary Table S2. This supplementary dataset is organized to facilitate exactly the kind of inquiry the reviewer suggests:
1. Each sheet represents a comparison between adjacent time intervals.
2. Columns correspond to specific clusters including the dynamic ones highlighted by the reviewer or regions.
3. Rows list the genes, with values indicating the expression differences.
Thus, the molecular transitions underlying the contraction, expansion, or aggregation of these specific cell types are fully captured and accessible within our provided data. We believe this comprehensive resource allows the community to mine the specific molecular programs driving these fascinating cellular behaviors. We have ensured that the reference to Supplementary Table S2 and Fig. 1I is clear in the Results section (Lines 209-211), emphasizing that time-resolved molecular differences for all clusters are available for detailed exploration.

Comment 9: The authors frequently reference Han et al. submitted, but this manuscript would need to be pre-printed or published in order for this work to reference it.

Response: We have updated the citation. The manuscript “Han et al.” has now been uploaded to bioRxiv (Maoqin Sun, Yuxiaofei Wang, Kai Han, et al. (2026). Cell Type Architecture and Positional Gene Gradients in an Adult Animal at Subcellular Resolution. bioRxiv, 2026.02.19.705280), and the full citation has been added to the reference list.

Comment 10: The Y axis of Figure 2E should be labeled

Response: We have added the missing label to the Y-axis of Fig. 2E as suggested.

Response to Reviewer #3:
General Comments: In the manuscript entitled "4D Single-Cell Spatial Transcriptomics Reveals Dynamic Morphogenetic Gradients and Regenerative Domains in Planarians", Han, Chen, Li et al. use high-resolution Stereo-seq on regenerating planarians to reconstruct a 4D spatiotemporal transcriptomic map of planarian regeneration. In their analysis, they recognize most of the cell types identified in planarians and are able to

recover the gene expression dynamics during regeneration of the body axes at the cellular, tissue, and organismal scales. One of the main findings is the identification of injury-induced spatial domains, specifically the Anterior Regenerative Zone (ARZ). Interestingly, the ARZ is enriched in positional control gene expression in several cell types, not only in muscle. The authors identify Mediator 8 as a gene expressed in the ARZ and required for proper blastema formation. The study also provides an interactive web portal with the corresponding data.
The analysis of the regeneration process in planarians using Stereo-seq provides a new and very useful strategy to understand the dynamics of gene expression integrated with cell and tissue types. Through this strategy, the authors corroborate the expression patterns of several genes already described as essential for planarian regeneration, and they identify new blastema regions comprising different expression patterns, both anterior and posterior. Among them, the study focuses on the ARZ and performs trajectory analysis, which provides a very informative view of the cellular movements and changes that occur at early stages of regeneration. The finding that Med8 is required to initiate regeneration in both wounds validates the utility of the strategy followed.
The publication of an open and interactive web portal with the dataset will be a useful tool for the planarian community and for research on regenerative processes in general.
However, in its present form, the study presents several weaknesses and issues that should be addressed before publication.

Main concerns:

The 3 main concerns are 1) the presentation of the strategy of each analysis performed is not detailed and not clear enough; 2) essential data for the present manuscript is supposedly found it Han et al. submitted, when it should be available in the present manuscript; and 3) the conclusions from the functional analysis of med8 are not accurate.

Response: We extend our sincere gratitude to Reviewer #3 for the comprehensive and insightful evaluation of our manuscript. We appreciate the recognition of our 4D spatiotemporal transcriptomic atlas as a valuable resource for the regeneration community. The reviewer's constructive criticism regarding the definition of spatially biased genes, the interpretation of med8 function, and the clarity of our data integration strategies has been instrumental in refining the logic and precision of this study. We have carefully addressed every point raised, with particular attention to clarifying the distinction between cell fate specification and polarity establishment, as well as correcting the presentation errors in Fig. 2. Below, we provide a detailed response to each concern and outline the corresponding revisions made to the manuscript.

Regarding concern 1:
Comment 1: - The authors explain that they analyzed 16 animals (2 per time point) processed into 10 μm thick sections, producing a total of 353 sections. However, they do not specify whether the same number of sections were analyzed per animal, nor do they indicate the total size (or thickness) and cell number of the animals analyzed.
In this regard, it may be that the number of sections analyzed per animal is shown in Figure S2A (this is not clear from the figure legend or the text). If so, why is there variation in the number of sections per animal? Is it due to differences in animal size? If so, how were these differences addressed in order to integrate the data from different animals?

Response: We apologize for not making the metadata describing individual animals sufficiently prominent in the main text. We clarify that we have indeed recorded detailed metrics for every analyzed animal and employed standard computational strategies to robustly integrate data across individuals despite their biological variations.
1. Animal-Specific Metrics
As listed in Table S1 (Data S1), we provided a comprehensive summary for each of the 16 individuals. This table explicitly documents the exact section number, 3D body dimensions (DV/ML/AP lengths), tissue volume, and total cell counts for every replicate. To ensure clarity, we have now explicitly referenced this table in the Results section (Line 136).
2. Source of Variation

We presume the reviewer intended to refer to Supplementary Fig. S1A (mentioned as Supplementary Fig. S2A in the comment), which illustrates the sections analyzed per animal. The reviewer correctly notes that there is variation in these section counts. This is primarily due to natural biological variation in body size. Although we selected animals of similar size for the experiment, slight differences in body thickness (DV axis) result in different total section numbers, as documented in the physical measurements in Table S1.
3. Data Integration Strategy
To address these size differences and integrate data from different animals, we applied distinct strategies for cellular identity and spatial analysis:
1) For Cell Type Identification: To define cell types consistently across individuals, we employed the standard Seurat integration workflow. This method identifies anchors between datasets to remove batch effects and biological variability inherent to different individuals, allowing us to cluster cells based on their shared transcriptomic profiles rather than their sample of origin.
2) For Spatial Pattern Analysis: We did not perform rigid physical alignment of coordinates between individuals. Instead, to compare spatial distributions across animals of different sizes, we normalized the spatial coordinates (AP, ML, and DV axes) to a relative range (e.g., 0–1 or percentage distance) for each individual. This allowed us to calculate and compare the distribution patterns of cell types and gene expression signals along the canonical body axes, independent of the absolute size of the animal.

Comment 2: - In Figure 1F, the different parts of the blastema are divided according to the pigmented/unpigmented area. What were the criteria used to divide each blastema into three parts (proximal, middle, and distal)? Gene expression? Length of the region? This should be clarified.

Response: We thank the reviewer for this question, as it highlights the need to better explain our segmentation logic. We clarify that the subdivision of the blastema was strictly anatomical and based on pigmentation patterns, not gene expression. This approach was chosen to provide an unbiased spatial reference frame independent of the molecular profiles we intended to study.
The segmentation process involved the following steps:
1. Pigmentation-Based Boundary Definition
We first identified the boundary between the pigmented (old tissue) and unpigmented (new blastema) regions using the Threshold function in ImageJ on microscopy images of the specific animals used for sequencing.
2. Quadratic Regression Refinement
To create a smooth, continuous mathematical boundary from the pixel data, we fitted a quadratic polynomial regression line to the pigmentation threshold.
3. Defining Border Regions (HBOR/HBIR)
A simple hard line often creates noise due to the irregular nature of the regeneration interface. To address this, we defined a buffer zone of 20 μm width on either side of the regression line.
1) HBOR (Head Border Outer Region): The 20 μm strip immediately inside the unpigmented blastema.
2) HBIR (Head Border Inner Region): The 20 μm strip immediately inside the pigmented trunk.
3) HAR (Head Anterior Region): The remaining distal portion of the blastema beyond the HBOR.
4) The same logic applies to the Tail blastema: TBOR, TBIR, and TPR.
This resulted in the distinct regions (HAR, HBOR, HBIR, Trunk, etc.) seen in our analysis. We then aligned the 2D projection of our 3D transcriptomic model with these microscopy images using TrakEM2 to assign every cell to one of these spatial domains based on its physical coordinate.
Following this precise spatial parcellation, the three defined regions at the anterior end (HAR, HBOR, and HBIR) and the three corresponding regions at the posterior end (TPR, TBOR, and TBIR) were collectively designated as the head and tail "blastema regions" in our analyses. In the context of Figure 1F, these three sub-regions structurally correspond to the distal (HAR/TPR), middle (HBOR/TBOR), and proximal (HBIR/TBIR) parts of the regenerating fields, respectively.
We have updated the Methods section under “Blastema region detection in 3D spatial transcriptomics data” to explicitly state that the sub-regions were defined by a fixed physical width (20 μm) relative to the pigmentation boundary, independent of gene expression (Lines 721-733).

Comment 3: - A schematic overview of each strategy used to obtain the results would help the reader understand the procedures.

Response: We thank the reviewer for this highly constructive suggestion. Given that this study integrates high-resolution spatial transcriptomics with a custom multidimensional computational framework encompassing 3D reconstruction, spatial pattern clustering, and polarity modeling, a clear, systematic workflow diagram is indeed essential for readers to grasp the experimental design and technical logic. In response, we have designed and generated a panoramic methodological flowchart (Fig. R5). This diagram explicitly details the pipeline from multimodal data input and preprocessing via the GEM3D toolkit to the core analytical modules (WACCA 3D reconstruction, SPC analysis, Polarity analysis, and other custom modules). We believe this overview significantly enhances the readability of the methodology and clarifies the coupling between different analytical steps. We have explicitly cited and described this flowchart in the Methods section of the manuscript (Lines 677-683).

Figure R5: A schematic overview of the entire computational workflow. This diagram illustrates the sequential processing pipeline, beginning with raw data input (h5ad and imaging files) and preprocessing via the GEM3D toolkit, followed by parallel analytical modules including WACCA for 3D reconstruction, SPC analysis for spatial clustering, and polarity analysis for modeling morphogenetic gradients.

Regarding concern 2:
Comment 4: - Throughout the study, the authors refer to Han et al., submitted for the custom framework used to create the atlas. Since that study is not currently available, this important information is missing from the current manuscript.

Response: We appreciate the reviewer for identifying this critical gap in data accessibility. We agree that access to the detailed algorithmic methodology underlying our custom 4D reconstruction framework is a prerequisite for assessing the technical rigor of this study. To ensure immediate transparency for the reviewers and the broader scientific community, we have deposited the companion manuscript describing the computational framework onto bioRxiv. The manuscript is now publicly accessible (DOI: 10.64898/2026.02.19.705280). We have updated all citations in the text to reflect this accessible version, ensuring that the full mathematical and computational details of the atlas construction are now readily available.

Comment 5: - Similarly, from the section "Spatiotemporal dynamics of positional gradients during whole-body regeneration" onward, the study relies on the so-called SBGs (spatially biased genes), also described in Han et al., submitted, which is not published. This is very important data that, if not published elsewhere, must be included in the current manuscript. A description of how the SBGs were obtained, together with a list and explanation, is required. Otherwise, essential information about the SBGs—on which the subsequent analyses depend—is entirely missing.

Response: We completely agree with the reviewer that the definition and identification of SBGs are fundamental to the subsequent analyses in our study. It is essential that this information be transparent and accessible within the current manuscript. To address this, we have ensured the manuscript is now self-contained regarding this methodology.

As mentioned in the response to the previous comment, the full methodological framework for identifying SBGs is detailed in the companion manuscript (Maoqin Sun, Yuxiaofei Wang, Kai Han, et al. (2026). Cell Type Architecture and Positional Gene Gradients in an Adult Animal at Subcellular Resolution. bioRxiv, 2026.02.19.705280), which is now available as a preprint (DOI: 10.64898/2026.02.19.705280).

To ensure this manuscript is self-contained, we have added a dedicated paragraph in the Methods section under "Identification of Spatially Biased Genes (SBGs)". This section summarizes the core logic of the algorithm used to define SBGs, enabling readers to understand the selection criteria without necessarily consulting the companion paper.

We confirm that the complete list of identified SBGs, along with their spatial domain annotations, is provided in Data S3 (Supplementary Table). We have updated the text to explicitly reference this dataset when SBGs are first introduced.

Comment 6: - According to the authors, PCGs are a subset of SBGs. What is the essential difference between PCGs and SBGs?

Response: This comment addresses a fundamental theoretical contribution of our work: the expansion of the positional information concept beyond the muscle lineage. Historically, the field has focused on PCGs, a specific class of genes, predominantly expressed in the body wall musculature, that encode signaling ligands (Wnt, BMP, FGF) and their antagonists. These genes act as the organism's GPS, constitutively defining the A/P, D/V, and M/L axes. Functional knockdown of PCGs results in catastrophic patterning defects, such as the regeneration of heads in place of tails.
However, our high-resolution 4D Stereo-seq atlas revealed a vast array of genes with highly regionalized expression patterns that do not fit the strict PCG definition. These genes are expressed in epidermis, gut, neural tissue, and parenchyma, and often do not show lethal patterning phenotypes upon knockdown, yet their spatial domains are tightly regulated. We define these as SBGs (Table R1).

Table R1. Differences between PCG and SBG

Feature	Position Control Genes (PCGs)	Spatially Biased Genes (SBGs)
Primary Tissue	Body Wall Muscle (sub-epidermal)	All Lineages (Epidermis, Gut, CNS, Muscle)
Function	Instructional: Define the coordinate system (GPS).	Interpretational: Read out the coordinates to specify local identity.
Molecular Class	Morphogens (Wnt, BMP, FGF), Antagonists (Notum, Noggin).	Diverse: Transcription factors, adhesion molecules, enzymes, structural proteins.
Discovery Method	Functional RNAi screens (phenotype-driven).	Spatial Autocorrelation Statistics (data-driven).
Relationship	A specific subset of SBGs.	The superset containing PCGs and their downstream targets.

To rigorously define SBGs, we moved beyond qualitative observation to statistical quantification. We applied Moran's I spatial autocorrelation statistics to the 3D reconstructed gene expression matrices of Intact animals. An SBG is defined as any gene with a Moran's I > 0.2 and a p-value < 0.01 (adjusted for multiple testing), indicating significant spatial clustering of expression. This analysis identified ~1,200 genes. Of these, only ~80 are canonical muscle PCGs. The remaining >1,100 genes represent the positional readout of the organism, the downstream interpretation of the muscle-derived map by other tissues.

Regarding concern 3:
Comment 7: - Functional analysis of med8. The authors refer to a role of this gene in polarity remodeling. First, this is an unclear concept, because polarity establishment and polarity remodeling are two different processes during regeneration. Second, the RNAi results presented do not support a role for med8 in polarity. The phenotype suggests that med8 is required for cell-fate specification during early stages of regeneration, since neoblasts increase but differentiated cells decrease in general. However, the results do not support a role for med8 in pole formation. The authors report a decrease in sfrp-1, but only at later stages, and no data are shown for notum. The conclusion that med8 regulates blastema growth and positional information is therefore not accurate and does not align with the results presented.

Response: We are grateful to the reviewer for this insightful dissection of the med8 phenotype. We fully agree with the reviewer's interpretation that the primary defect in med8 (RNAi) animals is a blockage in cell-fate specification and differentiation, rather than a direct regulation of polarity signaling or gradient scaling.
We acknowledge that our original use of the term "polarity remodeling" was imprecise. Based on the reviewer's suggestion, and supported by our data showing neoblast accumulation (Fig. 5B) alongside the loss of differentiated lineages (Fig. 5C), we have revised our conclusion to align with the mechanism of differentiation blockage.
We now clarify that the observed failure in polarity establishment (loss of anterior/posterior identity) is a secondary consequence of the failure to differentiate the specific cell populations (e.g., muscle and epidermal progenitors) that harbor positional landmarks.
The reviewer correctly points out the absence of notum data. To address this and further confirm the loss

of anterior identity, we have added the analysis of notum expression in the revised manuscript (added to Fig. 5E). Consistent with the loss of sfrp-1, notum expression is also severely diminished in med8 (RNAi) samples. This data reinforces the conclusion that the anterior pole identity fails to be re-established because the requisite cells are not generated.
We have removed the claim that med8 regulates positional information. Instead, the revised manuscript concludes that med8 is essential for providing the cellular basis for pole formation and maintaining the cell populations that harbor the positional landmarks of the global body axes (med8 part in Results section and Lines 544-557 in Discussion section).

Additional main concerns:
Comment 8: - A spatiotemporal transcriptomic atlas of planarian regeneration was already published in Cui et al. 2023. Although the authors cite it in the introduction, they should also compare their results with those published previously. Do they observe similar cell types and gene expression dynamics at the time points analyzed? This comparison should appear in both the Results and Discussion sections.

Response: We appreciate the reviewer's constructive suggestion to benchmark our findings against the recently published atlas by Cui et al. (Cui, G., Dong, K., Zhou, J.-Y., Li, S., Wu, Y., Han, Q., Yao, B., Shen, Q., Zhao, Y.-L., Yang, Y., Cai, J., Zhang, S., & Yang, Y.-G. (2023). Spatiotemporal transcriptomic atlas reveals the dynamic characteristics and key regulators of planarian regeneration. Nature Communications, 14(1), 3205.). We agree that a direct comparison strengthens the validity of our dataset while highlighting the technological advancements of our approach. We have performed a systematic Pearson correlation analysis between our Stereo-seq clusters and the spatial domains defined in the 10x Genomics Visium dataset from Cui et al..

As shown in the newly generated Fig. R6, we observed a strong concordance between the two datasets, where the spatial domains identified by 10x Genomics Visium map reliably to corresponding clusters in our Stereo-seq atlas. This confirms that our data captures the expected global gene expression dynamics. However, a critical distinction lies in the resolution. The 10x Genomics Visium platform utilizes capture spots with a diameter of 55 μm, which typically encompass 8–10 cells, resulting in a mixed transcriptomic signal. In contrast, our Stereo-seq platform achieves subcellular resolution (715 nm), enabling single-cell distinctness. Consequently, while the global domains align, the limited resolution of the 10x Genomics Visium dataset precludes the identification of the fine-scale cellular heterogeneity and rare transient states (such as the refined subtypes within the ARZ) that we report here.

Figure R6: Similarity comparison between Stereo-seq clusters and spatial domains from the 10x Genomics Visium datasets (Guanshen Cui et al., Nature Communications, 2023). The color scale represents the Pearson correlation coefficient, with red indicating higher similarity between the corresponding groups.

Comment 9: - The authors state that the different clusters of SBGs fluctuate during regeneration and then stabilize. First, which genes belong to each cluster? This information should be included. Second, according to Figure 2A, some clusters fluctuate (e.g., A1 and A2) but others do not (e.g., A5 or M9). A more accurate interpretation is required.
Furthermore, the starting point of the analysis is t0, when head and tail are missing, and the endpoint is 14 days of regeneration. How can one assess whether gene expression stabilizes if the starting and ending states are completely different?
Importantly, in Figure 2A, the cluster labels in the image do not correspond to the bars, and there are 15 bars but 16 clusters. The figure should be corrected. The colormap labels are also missing.

Response: We are deeply grateful for the reviewer's meticulous examination of Fig. 2A and the underlying logic of our SBG analysis. We apologize for the formatting oversights, which occurred during final figure assembly, and for the lack of clarity regarding our analytical framework. We have implemented the following revisions to address these concerns:
1. Gene Cluster Composition
We have compiled the complete lists of genes belonging to each SBG cluster across all regeneration timepoints. These detailed molecular profiles are now included in Data S3 (Supplementary Table), which is

explicitly referenced in the revised Results section to ensure full transparency (Lines 222-226).
2. Clarification of Analysis Logic and Fluctuation
We clarify that our analysis does not track a fixed set of genes over time, but rather the dynamic spatial attribution of gene expression patterns. We normalized the planarian body axis into 100 bins and established 16 standard spatial pattern cluster IDs based on intact animals (e.g., A1 for the anterior tip, A16 for the posterior tip) (Lines 747-769).
1) At T0 (Amputation), the physical removal of the head and tail results in the immediate loss of the spatial domains corresponding to anterior (A1, A2) and posterior (A15, A16) clusters. Consequently, the associated gene expression patterns are disrupted or physically absent, explaining the fluctuations or gaps noted by the reviewer. In contrast, trunk-associated clusters (e.g., A5) remain relatively stable as that tissue is preserved.
2) During regeneration, as the blastema forms and the body axis elongates, the missing spatial bins are reconstructed.
3) At 14 Days (Completion), the stabilization refers to the successful restoration of the gene expression patterns to a state that is highly congruent with the intact reference, signaling the completion of spatial re-patterning.
3. Correction of Fig. 2A:
We have entirely reconstructed Fig. 2A. The new figure strictly aligns the 16 cluster labels with their corresponding heatmap rows and includes a comprehensive colormap legend to facilitate quantitative interpretation.

Comment 10: - The authors find that predictions from the Gierer-Meinhardt model are consistent with the expression of some genes (ARNT, Ndk, EGR1…). First, a description and reference for these genes are required. Second, what about other genes involved in Wnt signaling and AP patterning, previously proposed to follow this model in Werner et al. 2015? Third, how can transcription factors such as Hox4b follow the model if they are not secreted?

Response: We thank the reviewer for this profound critique, which touches upon the conceptual foundation of our modeling approach. We acknowledge that transcription factors such as EGR1 and hox4b are nuclear-localized proteins and cannot function as the primary diffusive activator or inhibitor species within a classic Turing reaction-diffusion system. To address this, we have provided comprehensive descriptions and citations for the genes used in our modeling analysis. For instance, ndk is a conserved FGFR-related gene that restricts brain tissues to the head, while ARNT and EGR1 are transcription factors involved in neurogenesis and injury response. The full list of these genes and their roles in patterning is now summarized in Data S3.
The reviewer correctly notes that previous studies established self-organizing models for the Wnt pathway in planarians (Werner, S., Stuckemann, T., Beirán Amigo, M., Rink, J. C., Jülicher, F., & Friedrich, B. M. (2015). Scaling and regeneration of self-organized patterns. Physical Review Letters, 114(13), 138101.). Our study builds upon this by demonstrating that these global dynamics are reflected across a broader spectrum of the transcriptome. In planarians, the Wnt signaling pathway (Activator) and its secreted antagonist Notum (Inhibitor) form a canonical Turing pair. Our model effectively leverages these established kinetics to predict how downstream genes respond to these primary morphogen gradients (Adell, T., Cebrià, F., & Saló, E. (2010). Gradients in planarian regeneration and homeostasis. Cold Spring Harbor Perspectives in Biology, 2(1), a000505.).
We clarify that our model does not posit that TFs like hox4b are themselves diffusing. Instead, we propose that their spatiotemporal expression profiles serve as a downstream readout or proxy for the underlying, invisible morphogen fields (e.g., Wnt, BMP, and FGF ligands) that do diffuse and follow Gierer-Meinhardt kinetics. Because genes like hox4b are directly regulated by these gradients, their spatial expression patterns act as a shadow of the primary signaling landscape. By fitting these high-resolution data to the GM model, we are effectively quantifying the dynamics of the instructive morphogen gradients that drive whole-body reconstruction (Sureda-Gómez, M., & Adell, T. (2019). Planarian organizers. Seminars in Cell & Developmental Biology, 87, 95–104.) (Lines 273-284, Lines 448-465).

Comment 11: - In general, the manuscript does not refer to specific PCGs known to be critical for regeneration and patterning, such as notum and wnt1. Is this because they could not be identified in the dataset? If so, is it not possible to perform FISH and integrate these results with the Stereo-seq data?

Response: We appreciate the reviewer’s attention to these foundational patterning genes. We would like to clarify the technical context and the performance of our dataset regarding these transcripts:
There are several technical trade-offs of sequencing-based and FISH-based platforms. The primary objective of Stereo-seq is to achieve unbiased, whole-transcriptome capture at subcellular resolution. While FISH-based methods (e.g., MERFISH or seqFISH) offer higher sensitivity for specific low-abundance transcripts through targeted probe hybridization, they lack the discovery breadth of sequencing. Notably, in previously published planarian spatiotemporal atlases using 10x Genomics Visium (e.g., Cui, G., Dong, K., Zhou, J.-Y., Li, S., Wu, Y., Han, Q., Yao, B., Shen, Q., Zhao, Y.-L., Yang, Y., Cai, J., Zhang, S., & Yang, Y.-G. (2023)), notum and wnt1 were frequently undetectable.
Our Stereo-seq platform successfully captured both notum and wnt1. However, due to the biological nature of these signaling ligands—which are characterized by extremely low transcript levels and high spatial sparsity—their raw signal appears "noisier" compared to highly expressed structural or metabolic genes. Despite the sparsity, our data qualitatively recovers the expected dynamics of wnt1: a disorganized pattern at 36 hpa (early injury response) followed by clear enrichment at the posterior blastema at 3 dpa and 5 dpa, aligning with established FISH patterns. Similarly, notum signals are detected in the anterior regenerative regions, though their extreme sparsity makes them less suitable for the complex statistical modeling used for the broader SBG clusters.
To address the reviewer’s concern, we have generated spatial distribution plots for these canonical genes across key regeneration timepoints. These visualizations, which show high concordance with known FISH patterns, are referenced in the response as Fig. R7.
It is important to note that the core conclusions of our study, specifically the identification of the ARZ and the modeling of global morphogenetic gradients, rely on the statistical integration of over 1,000 SBGs. While notum and wnt1 serve as excellent biological benchmarks, the noise inherent in their detection does not undermine the robust coordinate system derived from the wider positional transcriptome.

Figure R7: Spatiotemporal expression of the posterior PCG wnt1 in regenerating planarians. (Left) Relative expression distribution of wnt1 along the A/P axis at 36 hpa, 3 dpa, and 5 dpa. For each time point, biological replicates were segmented into 10 bins along the A/P axis, and the average expression level within each bin was calculated. The peaks at the posterior end (bins 8-10) indicate the correct polarization of wnt1 expression during tail regeneration. (Right) Spatial visualization of wnt1 expression (black dots) within the body outline (black contour) of representative individuals at 3 dpa and 5 dpa. The expression is restricted to the posterior midline and tail tip, consistent with the known role of wnt1 in establishing posterior polarity.

Additional issues to be addressed:
Comment 12: - Lines 161-163: Why do the authors conclude that the increase in dorsal epidermal progenitors, neural progenitors, and pharyngeal lineages indicates an active wound response or the generation of new tissue? This statement is not clear.

Response: We appreciate the reviewer’s scrutiny regarding this interpretation. Our original statement was based on the distinct temporal dynamics and functional annotations of these specific lineages observed in our dataset. Although all three lineages increased, their onset times differed:
1. Dorsal Epidermal Progenitors (Epi.30) showed an early response, which we interpreted as participation in the initial wound response and closure.
2. Pharyngeal Lineages (Pha.19/29) emerged later, representing the de novo reconstruction of the missing pharynx, i.e., the generation of new tissue. Thus, we used the phrasing active wound response or the generation of new tissue to encompass these two distinct biological processes.
However, we fully agree with the reviewer that stating this conclusion based solely on cell number increases, without providing detailed functional context or separating the temporal phases in the immediate text, was potentially confusing and an over-interpretation.
To ensure clarity and precision, we have revised the sentence to focus on the common outcome of these changes: the expansion of lineages necessary for rebuilding the organism (Line 187-190).

Comment 13: - The 4D atlas annotates 36 refined cell types, which is a noteworthy result when compared with published scRNA-seq databases. The authors should compare their results with scRNA-seq in terms of the number and identity of cell types.

Response: We thank the reviewer for recognizing the resolution of our cell type annotation. We fully agree that benchmarking against established datasets is crucial for validating our atlas. We would like to clarify that a comprehensive comparative analysis between our Stereo-seq data and published scRNA-seq databases has been detailed in our companion manuscript (Han et al., now available as a preprint, DOI: 10.64898/2026.02.19.705280).
In that study, we integrated our spatial data with three major published planarian scRNA-seq datasets. In the comparative analysis, we observed a high degree of concordance in cell type identities and gene expression profiles between our spatial atlas and the traditional scRNA-seq datasets, validating the accuracy of our annotations. Crucially, our analysis highlighted that the spatial modality captures spatial heterogeneity and gene co-expression patterns that are often lost or conflated in dissociated single-cell data. This explains why we were able to define refined cell types (e.g., spatially co-expressed domain) that might appear as a single cluster in conventional scRNA-seq.
We have referenced this comparative analysis in the Results section where the cell types are introduced (Line 151-153).

Comment 14: - In Figure 1F, the blastema is divided according to the pigmented/unpigmented region. However, in Figure 1G the dividing lines do not follow this curvature. Should they not follow the pigment curvature as well?
Additionally, why do the blastemas in Figure 1G show such pronounced lateral deviation? Is this because the incisions were not perpendicular to the AP axis? If so, with this variability, it is difficult to understand how the datasets could be integrated. A detailed explanation is required.

Response: We appreciate the reviewer's careful observation. We realize that the visual representation in Fig. 1G may have caused a misunderstanding regarding our experimental standards and data visualization. We would like to clarify that the rectangular diagram on the left side of Fig. 1G is a conceptual schematic designed solely to illustrate the definition of the regions (HAR, HBOR, HBIR) based on their relative proximity to the wound site. It was simplified into straight lines for diagrammatic clarity.
In our actual data analysis, as correctly noted by the reviewer and shown in Fig. 1F, the segmentation boundaries strictly follow the natural curvature of the pigmented/unpigmented interface. The straight lines in the schematic do not represent the actual segmentation masks used.
Regarding the lateral deviation observed in the plots on the right side of Fig. 1G, we must clarify that these are UMAP plots, not physical spatial reconstructions of the tissue sections. UMAP is a dimensionality reduction technique where the axes (UMAP_1 and UMAP_2) represent latent transcriptomic features, not physical spatial coordinates (X and Y). The apparent tilt or deviation of the cell clusters reflects the topological structure of the data in the manifold space or the initialization of the algorithm. It does not correspond to the physical angle of the surgical incision. We assure the reviewer that all amputations were performed under a stereomicroscope with strict adherence to a perpendicular angle relative to the A/P axis.
Since the observed deviation is a computational artifact of the UMAP visualization and not a result of biological or surgical variability, it does not hinder data integration. Our integration strategy relies on robust transcriptomic alignment (Seurat) and relative spatial binning, which are unaffected by the rotational orientation of clusters in UMAP space.
We have updated the figure legend of Fig. 1G to explicitly state that the left panel is a "conceptual schematic" and the right panels are "UMAP visualizations of transcriptomic profiles".

Comment 15: - Lines 202-203: "To test this, we analyzed the spatiotemporal patterns of SBGs by mapping representative samples from each time point onto clusters along the A/P axis using logistic regression (Fig. 2A)." What is meant by "representative samples"? The procedure should be specified more clearly.

Response:
We appreciate the reviewer's scrutiny regarding our sampling and visualization strategy. In whole-organism spatial transcriptomics, biological replicates are exceptionally resource-intensive; our study

utilized two replicates per time point, totaling 16 animals. We clarify that the term “representative sample” was employed strictly in the context of visualization to provide the most biologically accurate single-worm illustration for Fig. 2A.
The selection was not arbitrary but governed by a rigorous statistical distance-to-centroid approach: For each time point, we aggregated the gene expression vectors from both biological replicates to compute a mean vector, or centroid. We then calculated the Pearson correlation of each individual replicate to this centroid. The animal exhibiting the higher correlation, meaning the individual whose transcriptomic state was mathematically closest to the mean, was selected for visualization to ensure the figure accurately reflects the average biological state of that regenerative stage.
Crucially, we emphasize that all quantitative analyses, including spatial clustering, differential expression, and PCA-based trajectory modeling, utilized the integrated data from both replicates to maintain statistical power and biological breadth. We have updated the Methods section to explicitly define this selection procedure (Lines 590-595).

Comment 16: - Data S4 is not mentioned in the text.

Response: We apologize for this oversight. Data S4 contains the full list of known PCGs and potential PCG candidates identified in our atlas, which served as the source pool for selecting the specific candidate genes characterized in this study.
While the comprehensive identification and global analysis of these gene lists are the focus of our companion manuscript (DOI: 10.64898/2026.02.19.705280), Data S4 is essential here to provide transparency regarding the candidate selection process for our computational results regarding the dynamics of spatially biased genes.
We have added citations to Data S4 in the Results section (Lines 225 and 242), explicitly stating that it serves as the list of candidate genes from which specific targets were selected for further analysis.

Comment 17: - smed03831, caveolin3, and smed01640 are the genes enriched in the ARZ domain. However, the authors perform functional analysis only with med8. Is there any specific reason for this?

Response: We thank the reviewer for this question regarding our candidate selection strategy. The rationale for focusing on med8 over other highly enriched genes such as smed03831 or caveolin3 lies in our distinction between structural markers and upstream regulators.
Genes such as smed03831 and caveolin3 are highly enriched in the ARZ and serve as excellent differentiation markers for this domain. In fact, we utilized smed03831 extensively in our study as a phenotypic readout (Fig. 4G-J) to assess whether the ARZ was successfully formed. However, based on their predicted functions (e.g., structural roles), they are less likely to be the master regulators driving the formation of the domain itself. Our goal was to identify the drivers controlling ARZ specification. Through our pseudotime trajectory analysis (Fig. 4F), med8 emerged as an early-expressed gene that precedes the peak expression of differentiation markers like smed03831. Furthermore, as a subunit of the Mediator complex, med8 plays a fundamental role in transcriptional regulation (Brower, C. S., Sato, S., Tomomori-Sato, C., Kamura, T., Pause, A., Stearman, R., Klausner, R. D., Malik, S., Lane, W. S., Sorokina, I., Roeder, R. G., Conaway, J. W., & Conaway, R. C. (2002). Mammalian mediator subunit mMED8 is an Elongin BC-interacting protein that can assemble with Cul2 and Rbx1 to reconstitute a ubiquitin ligase. Proceedings of the National Academy of Sciences, 99(16), 10353–10358.), making it a strong candidate for controlling cell fate decisions and lineage specification. Therefore, we prioritized med8 for functional analysis to uncover the molecular mechanism governing the establishment of the ARZ, rather than its maintenance or structural composition.
We have clarified this selection logic in the Results section (Lines 362-363), explicitly stating that we prioritized candidate genes based on their early temporal expression and potential regulatory functions to identify drivers of ARZ formation.

Cookie Preference Center

We use cookies which are necessary to make our site work. We may also use additional cookies to analyze, improve and personalize our content and your digital experience. For more information, see our Cookie Policy.

You may choose not to allow some types of cookies. However, blocking some types may impact your experience of our site and the services we are able to offer. See the different category headings below to find out more or change your settings.

Allow all

Manage Consent Preferences

Strictly Necessary Cookies

Always active

These cookies are necessary for the website to function and cannot be switched off in our systems. They are usually only set in response to actions made by you which amount to a request for services, such as setting your privacy preferences, logging in or filling in forms. You can set your browser to block or alert you about these cookies, but some parts of the site will not then work.

Functional Cookies

These cookies enable the website to provide enhanced functionality and personalisation. They may be set by us or by third party providers whose services we have added to our pages. If you do not allow these cookies then some or all of these services may not function properly.

Performance Cookies

These cookies allow us to count visits and traffic sources so we can measure and improve the performance of our site. They help us to know which pages are the most and least popular and see how visitors move around the site.

Targeting Cookies

These cookies may be set through our site by our advertising partners. They may be used by those companies to build a profile of your interests and show you relevant adverts on other sites. If you do not allow these cookies, you will experience less targeted advertising.

Cookie List

Clear

Apply Cancel
Consent Leg.Interest

Confirm my choices

Power
