# Supplementary material for: 4D single-cell spatial transcriptomics reveals dynamic morphogenetic gradients and regenerative domains in planarians
Source: Gigascience. 2026 May 22;15:giag064. doi: 10.1093/gigascience/giag064 (PMC13273413; doi:10.1093/gigascience/giag064)
Supplement: giag064_Authors_Response_To_Reviewer_Comments_revision_1 [file giag064_Authors_Response_To_Reviewer_Comments_revision_1.pdf]

Author's Response To Reviewer Comments

Reviewer #2: In the revised manuscript '4D single-cell spatial transcriptomics reveals dynamic morphogenetic gradients and regenerative domains in planarians,' Han and colleagues improved their data analysis and data interpretation from the previous submission. Overall, my concerns were sufficiently addressed by additional analysis and minor experiments, or by softening the conclusions of the authors, for me to recommend publication after minor revisions. I've detailed my remaining minor comments/criticisms below:

Response to Major Criticisms:
1. Stereo-seq resolution and capture efficiency: The authors have made significant efforts to reframe their stereo-seq clusters as 'domains' rather than 'cell types.' However, there are few areas where the authors refer to cells when I believe they mean tissues or domains. Similarly, when discussing the med8 RNAi scRNAseq data, cell types rather than domains is the correct description of datapoints, so they need to be described as such. A careful edit through the document will resolve this issue. The authors' added QC statistics for their dataset are also sufficient, but the segmented cell areas should be reported in microns, rather than pixels.
Response: We thank the reviewer for pointing this out. We have carefully reviewed the manuscript and corrected the remaining instances where "cells" was inadvertently used instead of "spatial domains" to describe Stereo-seq spatial clusters. The terms "cells" and "cell types" are now strictly reserved for the med8 RNAi scRNA-seq data. Furthermore, we have updated Data S1, converting the segmented cell areas from pixels to square microns (μm^2) based on our physical resolution (1 pixel = 0.715 μm, and therefore 1 $pixel^2 \approx 0.511 \mu m^2$).

2. Dynamics of spatially biased genes: The authors' improved modeling that describes the SBG dynamics as a disruption and then return to equilibrium is much improved from the previous version and more consistently fits with the observed data. As such, I feel that the authors sufficiently addressed this prior criticism.
Response: We appreciate the positive feedback. As suggested by the reviewer, the improved modeling approach indeed provides a more robust fit for the observed biological dynamics.

3. The Cellular/Functional identity of Clu.31: The authors conclude that the ARZ is a 'co-regulated, regeneration-responsive regions' (Line 370) containing three primary tissue lineages - epidermis, muscle, and neurons. I found the description of the ARZ and the authors conclusions much more consistent with the data. I also found the discussion of the potential similarity between the ARZ and the apical cap in the discussion much improved. However, I still believe that the ARZ (given its existence at homeostasis) may not be exclusive to regeneration, but rather a cellular neighborhood that is resident to the anterior CNS and whose cellular components have significantly altered transcription during regeneration (even to the point of being transiently induced in posterior wounded CNS). But this difference in interpretation may be more rhetorical than substantive.
Response: We agree with the reviewer's perspective. Given its presence during homeostasis, it is highly probable that the ARZ represents a resident cellular neighborhood within the anterior tissue rather than an entirely de novo structure exclusive to regeneration. This conceptual framework aligns well with our spatial data and elegantly explains the hyper-responsive transcriptional alterations observed upon injury, including its transient ectopic induction in the posterior wound.
Accordingly, we have updated the Discussion section (Lines 516-523) to explicitly integrate this interpretation. We now describe the ARZ as a "specialized, resident cellular neighborhood within the uninjured anterior tissue", emphasizing its baseline existence, plasticity (including transient ectopic induction in the posterior wound), and responsive transcriptional activation during regeneration.

4. Med8 function: The authors interpretation of their data related to Med8 function is significantly improved and I believe their conclusions and discussion points are now supported by the data.
Response: We thank the reviewer for the positive assessment of our revised analysis and conclusions regarding Med8.

Minor Remaining Criticism/Feedback:
1. Previously, I expressed surprise at the number of DEGs that the authors report for each time point. It appears that there are ~10K upregulated and 10K downregulated DEGs by the later time points, which suggests that 2/3 of the transcriptome is differentially expressed… While the authors did clarify the cutoff they used for the DEGs, they still did not address the BIOLOGICAL significance of the DEGs in this figure. scRNAseq and spatial transcriptomics datasets often have artificially reduced P values due to the extreme number of 'cells/domains' captured. However, these genes do not have significant fold changes and are often not able to be detected by independent means in vivo. The authors should address this concern when reported DEGs and in their methods.
Response: We agree with the reviewer that relying solely on adjusted P-values in high-throughput spatial transcriptomics can lead to P-value inflation, capturing genes with statistically significant but biologically marginal expression changes.
To ensure the biological relevance of our DEGs, we have now applied a strict fold-change threshold (|log2(Fold Change)| > 0.5) in addition to the standard P-adj < 0.05 cutoff. Implementing this stringency effectively filtered out background noise, refining the maximum number of DEGs at the later regenerative stages to a more biologically meaningful ~3,000 genes. We have updated Figure 1I to reflect this refined dataset and have detailed these filtering criteria in the Methods and Results sections.

Reviewer #3: In the revised version of the manuscript, the authors have adequately addressed the main concerns. I recommend it for publication.
Response: We thank the reviewer for their time, their constructive feedback throughout the review process, and their recommendation for publication.

Editorial Manager®

Cookie Preference Center

We use cookies which are necessary to make our site work. We may also use additional cookies to analyze, improve and personalize our content and your digital experience. For more information, see our Cookie Policy.

You may choose not to allow some types of cookies. However, blocking some types may impact your experience of our site and the services we are able to offer. See the different category headings below to find out more or change your settings.

Allow all

Manage Consent Preferences

Strictly Necessary Cookies

Always active

These cookies are necessary for the website to function and cannot be switched off in our systems. They are usually only set in response to actions made by you which amount to a request for services, such as setting your privacy preferences, logging in or filling in forms. You can set your browser to block or alert you about these cookies, but some parts of the site will not then work.

Functional Cookies

These cookies enable the website to provide enhanced functionality and personalisation. They may be set by us or by third party providers whose services we have added to our pages. If you do not allow these cookies then some or all of these services may not function properly.

Performance Cookies

These cookies allow us to count visits and traffic sources so we can measure and improve the performance of our site. They help us to know which pages are the most and least popular and see how visitors move around the site.

Targeting Cookies

These cookies may be set through our site by our advertising partners. They may be used by those companies to build a profile of your interests and show you relevant adverts on other sites. If you do not allow these cookies, you will experience less targeted advertising.

Cookie List

Clear

Apply Cancel

Consent Leg.Interest

Confirm my choices

Powe
