# Supplementary material for: 4D single-cell spatial transcriptomics reveals dynamic morphogenetic gradients and regenerative domains in planarians
Source: Gigascience. 2026 May 22;15:giag064. doi: 10.1093/gigascience/giag064 (PMC13273413; doi:10.1093/gigascience/giag064)
Supplement: giag064_Reviewer_1_Report_original_submission — Reviewer 1 -- 11/20/2025 [file giag064_Reviewer_1_Report_original_submission.pdf]

Reviewer Report

Title: 4D Single-Cell Spatial Transcriptomics Reveals Dynamic Morphogenetic Gradients and Regenerative Domains in Planarians

Version: Original Submission **Date:** 11/20/2025

Reviewer name: Reviewer 1

Reviewer Comments to Author:

The authors employs high-resolution Stereo-seq technology combined with multi-timepoint spatial transcriptomic data to construct a 4D spatiotemporal transcriptomic map of planarian regeneration. This work significantly advances the understanding of spatial gene expression dynamics during planarian regeneration, overcoming the limitations of traditional two-dimensional and planar spatial transcriptomics. Furthermore, the authors identify a novel injury-indced Anterior Regenerative Zone (ARZ) and, through functional validation of Mediator 8 (Med8), deepen insights into the mechanisms underlying polarity remodeling in planarians. The study also provides an interactive online database, enriching spatial molecular and cellular data resources for the regenerative biology. The work is notably innovative, and the authors present convincing evidence supporting their conclusions. The manuscript overall is written well and data is presented clearly. The discussion and conclusions has done well to highlight the potential problems in this study. I have a few points that should be addressed before publishingI have a few points that should be addressed before publishing.
1.In lines 233-236, it is reported that the positional control gene (PCG) like ndk restores its spatial expression pattern as early as 12 hpa, whereas its expression level only significantly increases at 36 hpa. Given this pronounced temporal discordance between early recovery of spatial patterning and the later peak in mRNA levels, the authors should analyze and discuss possible molecular mechanisms that could account for this discrepancy, and consider the biological implications of this phenomenon for understanding how spatial information and gene-expression regulation are coordinated during regeneration.
2.In lines 356-360, Med8 knockdown markedly reduces the ARZ cell lineages and the expression of anterior-posterior polarity markers (e.g., sfrp-1, wnt1, wnt11-1), producing a clear effect on regeneration polarity formation.However no gross disruption of the whole-body AP axis was observed. Please further analyze and discuss the possible regulatory scope and mechanisms of Med8. Specifically, do other redundant pathways or compensatory mechanisms exist in planarians that maintain global positional information despite loss of Med8? What is the hierarchical and cell-type specificity of Med8's role in polarity regulation?

Methods

Are the methods appropriate to the aims of the study, are they well described, and are necessary controls included? Choose an item.

Conclusions

Are the conclusions adequately supported by the data shown? Choose an item.

Reporting Standards

Does the manuscript adhere to the journal's guidelines on [minimum standards of reporting?] Choose an item.

Choose an item.

Statistics

Are you able to assess all statistics in the manuscript, including the appropriateness of statistical tests used? Choose an item.

Quality of Written English

Please indicate the quality of language in the manuscript: Choose an item.

Declaration of Competing Interests

Please complete a declaration of competing interests, considering the following questions:

- Have you in the past five years received reimbursements, fees, funding, or salary from an organisation that may in any way gain or lose financially from the publication of this manuscript, either now or in the future?
- Do you hold any stocks or shares in an organisation that may in any way gain or lose financially from the publication of this manuscript, either now or in the future?
- Do you hold or are you currently applying for any patents relating to the content of the manuscript?
- Have you received reimbursements, fees, funding, or salary from an organization that holds or has applied for patents relating to the content of the manuscript?
- Do you have any other financial competing interests?
- Do you have any non-financial competing interests in relation to this paper?

If you can answer no to all of the above, write 'I declare that I have no competing interests' below. If your reply is yes to any, please give details below.

I declare that I have no competing interests' below.

I agree to the open peer review policy of the journal. I understand that my name will be included on my report to the authors and, if the manuscript is accepted for publication, my named report including any attachments I upload will be posted on the website along with the authors' responses. I agree for my report to be made available under an Open Access Creative Commons CC-BY license (http://creativecommons.org/licenses/by/4.0/). I understand that any comments which I do not wish to

be included in my named report can be included as confidential comments to the editors, which will not be published.

Choose an item.

To further support our reviewers, we have joined with Publons, where you can gain additional credit to further highlight your hard work (see: https://publons.com/journal/530/gigascience). On publication of this paper, your review will be automatically added to Publons, you can then choose whether or not to claim your Publons credit. I understand this statement.

Yes Choose an item.
