# Supplementary material for: 4D single-cell spatial transcriptomics reveals dynamic morphogenetic gradients and regenerative domains in planarians
Source: Gigascience. 2026 May 22;15:giag064. doi: 10.1093/gigascience/giag064 (PMC13273413; doi:10.1093/gigascience/giag064)
Supplement: giag064_Reviewer_2_Report_original_submission — Reviewer 2 -- 11/26/2025 [file giag064_Reviewer_2_Report_original_submission.pdf]

Reviewer Report

Title: 4D Single-Cell Spatial Transcriptomics Reveals Dynamic Morphogenetic Gradients and Regenerative Domains in Planarians

Version: Original Submission **Date:** 11/26/2025

Reviewer name: Reviewer 2

Reviewer Comments to Author:

In the manuscript '4D single-cell spatial transcriptomics reveals dynamic morphogenetic gradients and regenerative domains in planarians,' Han and colleagues generate a truly stunning spatial transcriptomics dataset of planarian regeneration from the species Schmidtea mediterranea. The authors' dataset includes whole 3D reconstructions of two regenerating planarian fragments at 8 different timepoints during regeneration, a fantastic accomplishment and resource of broad interest to the regenerative biology community. The authors analysis of the dataset includes characterization of spatially biased genes (SBGs) and exploration of an anterior regenerative zone (ARZ) and the role of the gene med8 in its' regulation. While the authors' dataset is remarkable and their analysis of spatially biased genes and med8 function is interesting, I'm not yet convinced that their conclusions are fully tested by the included experiments. In addition, I think that the authors have not included sufficient quality control metrics for their spatial dataset, which makes determining the limitations or caveats of their analysis and conclusions more difficult. However, my concerns could be addressed by additional analysis and minor experiments, or by softening the conclusions of the authors to include alternative models. I've detailed the areas of analysis/discussion that I believe require improvement below:

Major Criticisms:

1. Stereo-seq resolution and capture efficiency: The authors assert that their spatial approach is high enough resolution to resolve cell types and they claim to have characterized 36 cell types in their abstract. However, the 'cell type' in their dataset that they choose to focus on - Clu.31 - has gene markers expressed in three different cell types that have been shown to be distinct in the literature and prior planarian atlases. The authors should analyze gene expression signatures of other stereo-seq 'cell types' to determine if they also show mixed expression signatures. In addition, I am curious if stereo-seq is more likely to capture highly expressed genes (like those expressed in parenchymal cell types) than more lowly expressed genes (like the transcription factors expressed in stem cells). If it exists, this bias could influence annotation of cell types in highly heterogeneous regions of the worm like the parenchyma or parapharyngeal region. Finally, there is very little QC data in the supplementary materials (Size/volume of segmented cells, UMIs and features per cell, variability in features/UMIs per section, per replicate, and per cell type, etc.) I think this analysis would be highly valuable for the reader to interpret the data and the 36 identified 'cell types'.

2. Dynamics of spatially biased genes: The authors analysis on the dynamics of spatially biased genes (SBGs) is very interesting, but the 'oscillations' the authors referred to were not clear to me in the data across all or even most of the pattern clusters in Figure 2A. In general, it seemed more like the pattern cluster was 'noisy' or more broad before stabilizing to its final location. In addition, the PCA analysis in

Figure 2B seems to show that Intact and 14dpa transcriptomics is very similar, but 0h, 12h, and 36h timepoints are very distinct from 3, 5, 7, and 10 day fragments. This would suggest that early wound response gene expression is highly distinct (even opposing) the gene expression programs active during late in regeneration. More exploration of this idea, as well as clarified language on exactly what the author means by 'oscillations' and which gene groups follow this pattern would greatly improve this section and better support the author's conclusions.

3. The Cellular/Functional identity of Clu.31: The authors state throughout the manuscript that Clu.31 (the ARZ) is an injury-induced anterior state enriched for SBGs and regulating polarity establishment. However, it is also possible that this spatial state represents the anterior peripheral nervous system (numerous sensory neurons and surface epithelial cells that help sense mechanical and chemical cues). SBGs could be enriched because this combination of cell types is only present in the anterior of the animal. Indeed, the authors show that the ARZ is localized to the anterior in intact animals in the absence of an injury (Figure 3) and enriched genes (S4Aii) strongly indicate that Clu.31 contains gabrg+ mechanosensory neurons. If Clu.31 is regenerating nervous system, this would also explain its ventral bias and expression of tgs-1 and other nb2 genes, since nb2 neoblasts have been suggested to be both an amputation responsive neoblast subset (Zeng et al. Cell) and a neural progenitor state (Raz et al. Cell Stem Cell). Clarifying how the composition of the tri-lineage region changes during regeneration may help distinguish if Clu.31 is truly an injury induced region vs. the regenerating sensory nervous system. For example, it is known that agat-1+ cells transcriptionally responsive and enriched at the wound site a 2-4 days post amputation, but less so at later timepoints (Benham-Pyle et al Nature Cell Biology, Kent et al. Developmental Biology). This shift in composition should be observable in Clu.31 since it contains agat+ epidermal cells. Such a shift in composition or the identification of a regeneration-specific marker expressed in Clu.31 would add support to the author's conclusions. Regardless of the outcome of these experiments/analyses, the discussion and interpretation of the data could be modified to address the hypothesis that Clu.31 represents the cellular neighborhood created when the peripheral nervous system intercalates with the anterior DV boundary epithelium and body wall muscle, which needs to be regenerated in amputated worms. As is, the comparison to the apical epithelial cap considered in the discussion (Line 438) may be pre-mature.

4. Med8 function: Med8 produces a clear phenotype in the authors' experiments, and their data indicates that it is required for ARZ formation. However, I am not sure that the authors data supports the claim that Med8 is directly regulating blastema and PCG expression, as opposed to regeneration of the nervous system (which is highly interconnected with formation of the anterior pole and the size of the anterior blastema) and stem cell function more broadly. The fact that Med8 RNAi also leads to head degeneration in intact worms (Figure S6F) strongly suggests a more fundamental defect in neural differentiation or stem cell function. The strongest evidence presented by the authors supporting a broader function in polarity establishment is the disruption of posterior Wnt expression, (Figure 5F and G), but these in situs are single representative images with no quantitation and could also be explained by a stem cell defect. Additional data could be provided (e.g. visualization of wound-induced gene expression, quantitation of anterior or posterior stem cell numbers and proliferation rates at 2dpa) to support regulation of PCGs or blastema formation. The authors could also leverage their single cell sequencing to determine if Med8 RNAi impacts neural progenitor abundance more than other progenitor cell types. Together, these experiments would determine if Med8 is important for

amputation-induced blastema formation and polarity re-establishment vs. stem cell function and neural differentiation more broadly.

Minor Criticism/Feedback:

1. In Figure 1I, the authors show DEGs enriched in each cluster/region. In the blastema regions, I was surprised by the number of DEGs for each time point. It appears that there are ~10K upregulated and 10K downregulated DEGs by the later time points, which suggests that 2/3 of the transcriptome is differentially expressed… The authors should clarify in the text or methods what cutoff they used for the DEGs and how significant the DEGs are in this figure.
2. For readability, I really think that all figures should be on a white background.
3. How do gene expression profiles from the stereo-seq compare to bulk rnaseq at similar timepoints?
4. It is very interesting that there are some cell types that appear to contract and then expand during regeneration (Cluster 0, 23) or that aggregate/become more targeted during regeneration (pharynx pouch, cluster 29). Molecular differences between early and late cells within these cell types would be particularly interesting for understanding different phases of regeneration, but this may be beyond the scope of the current study.
5. The authors frequently reference Han et al. submitted, but this manuscript would need to be pre-printed or published in order for this work to reference it.
6. The Y axis of Figure 2E should be labeled

Methods

Are the methods appropriate to the aims of the study, are they well described, and are necessary controls included? Choose an item.

Conclusions

Are the conclusions adequately supported by the data shown? Choose an item.

Reporting Standards

Does the manuscript adhere to the journal's guidelines on minimum standards of reporting? Choose an item.

Choose an item.

Statistics

Are you able to assess all statistics in the manuscript, including the appropriateness of statistical tests used? Choose an item.

Quality of Written English

Please indicate the quality of language in the manuscript: Choose an item.

Declaration of Competing Interests

Please complete a declaration of competing interests, considering the following questions:

- Have you in the past five years received reimbursements, fees, funding, or salary from an organisation that may in any way gain or lose financially from the publication of this manuscript, either now or in the future?
- Do you hold any stocks or shares in an organisation that may in any way gain or lose financially from the publication of this manuscript, either now or in the future?
- Do you hold or are you currently applying for any patents relating to the content of the manuscript?
- Have you received reimbursements, fees, funding, or salary from an organization that holds or has applied for patents relating to the content of the manuscript?
- Do you have any other financial competing interests?
- Do you have any non-financial competing interests in relation to this paper?

If you can answer no to all of the above, write 'I declare that I have no competing interests' below. If your reply is yes to any, please give details below.

I declare that I have no competing interests

I agree to the open peer review policy of the journal. I understand that my name will be included on my report to the authors and, if the manuscript is accepted for publication, my named report including any attachments I upload will be posted on the website along with the authors' responses. I agree for my report to be made available under an Open Access Creative Commons CC-BY license (http://creativecommons.org/licenses/by/4.0/). I understand that any comments which I do not wish to be included in my named report can be included as confidential comments to the editors, which will not be published.

Choose an item.

To further support our reviewers, we have joined with Publons, where you can gain additional credit to further highlight your hard work (see: https://publons.com/journal/530/gigascience). On publication of this paper, your review will be automatically added to Publons, you can then choose whether or not to claim your Publons credit. I understand this statement.

Yes Choose an item.
