# Supplementary material for: 4D single-cell spatial transcriptomics reveals dynamic morphogenetic gradients and regenerative domains in planarians
Source: Gigascience. 2026 May 22;15:giag064. doi: 10.1093/gigascience/giag064 (PMC13273413; doi:10.1093/gigascience/giag064)
Supplement: giag064_Reviewer_2_Report_revision_1 — Reviewer 2 -- 3/23/2026 [file giag064_Reviewer_2_Report_revision_1.pdf]

Reviewer Report

Title: 4D Single-Cell Spatial Transcriptomics Reveals Dynamic Morphogenetic Gradients and Regenerative Domains in Planarians

Version: Revision 1 **Date:** 3/23/2026

Reviewer name: Reviewer 2

Reviewer Comments to Author:

In the revised manuscript '4D single-cell spatial transcriptomics reveals dynamic morphogenetic gradients and regenerative domains in planarians,' Han and colleagues improved their data analysis and data interpretation from the previous submission. Overall, my concerns were sufficiently addressed by additional analysis and minor experiments, or by softening the conclusions of the authors, for me to recommend publication after minor revisions. I've detailed my remaining minor comments/criticisms below:

Response to Major Criticisms:

1. Stereo-seq resolution and capture efficiency: The authors have made significant efforts to reframe their stereo-seq clusters as 'domains' rather than 'cell types.' However, there are few areas where the authors refer to cells when I believe they mean tissues or domains. Similarly, when discussing the med8 RNAi scRNAseq data, cell types rather than domains is the correct description of datapoints, so they need to be described as such. A careful edit through the document will resolve this issue. The authors' added QC statistics for their dataset are also sufficient, but the segmented cell areas should be reported in microns, rather than pixels.

2. Dynamics of spatially biased genes: The authors' improved modeling that describes the SBG dynamics as a disruption and then return to equilibrium is much improved from the previous version and more consistently fits with the observed data. As such, I feel that the authors sufficiently addressed this prior criticism.

3. The Cellular/Functional identity of Clu.31: The authors conclude that the ARZ is a 'co-regulated, regeneration-responsive regions' (Line 370) containing three primary tissue lineages - epidermis, muscle, and neurons. I found the description of the ARZ and the authors conclusions much more consistent with the data. I also found the discussion of the potential similarity between the ARZ and the apical cap in the discussion much improved. However, I still believe that the ARZ (given its existence at homeostasis) may not be exclusive to regeneration, but rather a cellular neighborhood that is resident to the anterior CNS and whose cellular components have significantly altered transcription during regeneration (even to the point of being transiently induced in posterior wounded CNS). But this difference in interpretation may be more rhetorical than substantive.

4. Med8 function: The authors interpretation of their data related to Med8 function is significantly improved and I believe their conclusions and discussion points are now supported by the data.

Minor Remaining Criticism/Feedback:

1. Previously, I expressed surprise at the number of DEGs that the authors report for each time point. It appears that there are ~10K upregulated and 10K downregulated DEGs by the later time points, which

suggests that 2/3 of the transcriptome is differentially expressed… While the authors did clarify the cutoff they used for the DEGs, they still did not address the BIOLOGICAL significance of the DEGs in this figure. scRNAseq and spatial transcriptomics datasets often have artificially reduced P values due to the extreme number of 'cells/domains' captured. However, these genes do not have significant fold changes and are often not able to be detected by independent means in vivo. The authors should address this concern when reported DEGs and in their methods.

Methods

Are the methods appropriate to the aims of the study, are they well described, and are necessary controls included? Choose an item.

Conclusions

Are the conclusions adequately supported by the data shown? Choose an item.

Reporting Standards

Does the manuscript adhere to the journal's guidelines on minimum standards of reporting? Choose an item.

Choose an item.

Statistics

Are you able to assess all statistics in the manuscript, including the appropriateness of statistical tests used? Choose an item.

Quality of Written English

Please indicate the quality of language in the manuscript: Choose an item.

Declaration of Competing Interests

Please complete a declaration of competing interests, considering the following questions:

- Have you in the past five years received reimbursements, fees, funding, or salary from an organisation that may in any way gain or lose financially from the publication of this manuscript, either now or in the future?
- Do you hold any stocks or shares in an organisation that may in any way gain or lose financially from the publication of this manuscript, either now or in the future?
- Do you hold or are you currently applying for any patents relating to the content of the manuscript?

- Have you received reimbursements, fees, funding, or salary from an organization that holds or has applied for patents relating to the content of the manuscript?
- Do you have any other financial competing interests?
- Do you have any non-financial competing interests in relation to this paper?

If you can answer no to all of the above, write 'I declare that I have no competing interests' below. If your reply is yes to any, please give details below.

I declare that I have no competing interests

I agree to the open peer review policy of the journal. I understand that my name will be included on my report to the authors and, if the manuscript is accepted for publication, my named report including any attachments I upload will be posted on the website along with the authors' responses. I agree for my report to be made available under an Open Access Creative Commons CC-BY license (http://creativecommons.org/licenses/by/4.0/). I understand that any comments which I do not wish to be included in my named report can be included as confidential comments to the editors, which will not be published.

I agree to the open peer review policy of the journal

To further support our reviewers, we have joined with Publons, where you can gain additional credit to further highlight your hard work (see: https://publons.com/journal/530/gigascience). On publication of this paper, your review will be automatically added to Publons, you can then choose whether or not to claim your Publons credit. I understand this statement.

Yes Choose an item.
