# Supplementary material for: 4D single-cell spatial transcriptomics reveals dynamic morphogenetic gradients and regenerative domains in planarians
Source: Gigascience. 2026 May 22;15:giag064. doi: 10.1093/gigascience/giag064 (PMC13273413; doi:10.1093/gigascience/giag064)
Supplement: giag064_Reviewer_3_Report_original_submission — Reviewer 3 -- 11/30/2025 [file giag064_Reviewer_3_Report_original_submission.pdf]

Reviewer Report

Title: 4D Single-Cell Spatial Transcriptomics Reveals Dynamic Morphogenetic Gradients and Regenerative Domains in Planarians

Version: Original Submission **Date:** 11/30/2025

Reviewer name: Reviewer 3

Reviewer Comments to Author:

In the manuscript entitled "4D Single-Cell Spatial Transcriptomics Reveals Dynamic Morphogenetic Gradients and Regenerative Domains in Planarians", Han, Chen, Li et al. use high-resolution Stereo-seq on regenerating planarians to reconstruct a 4D spatiotemporal transcriptomic map of planarian regeneration. In their analysis, they recognize most of the cell types identified in planarians and are able to recover the gene expression dynamics during regeneration of the body axes at the cellular, tissue, and organismal scales. One of the main findings is the identification of injury-induced spatial domains, specifically the Anterior Regenerative Zone (ARZ). Interestingly, the ARZ is enriched in positional control gene expression in several cell types, not only in muscle. The authors identify Mediator 8 as a gene expressed in the ARZ and required for proper blastema formation. The study also provides an interactive web portal with the corresponding data.

The analysis of the regeneration process in planarians using Stereo-seq provides a new and very useful strategy to understand the dynamics of gene expression integrated with cell and tissue types. Through this strategy, the authors corroborate the expression patterns of several genes already described as essential for planarian regeneration, and they identify new blastema regions comprising different expression patterns, both anterior and posterior. Among them, the study focuses on the ARZ and performs trajectory analysis, which provides a very informative view of the cellular movements and changes that occur at early stages of regeneration. The finding that Med8 is required to initiate regeneration in both wounds validates the utility of the strategy followed.

The publication of an open and interactive web portal with the dataset will be a useful tool for the planarian community and for research on regenerative processes in general.

However, in its present form, the study presents several weaknesses and issues that should be addressed before publication.

Main concerns:

The 3 main concerns are 1) the presentation of the strategy of each analysis performed is not detailed and not clear enough; 2) essential data for the present manuscript is supposedly found it Han et al. submitted, when it should be available in the present manuscript; and 3) the conclusions from the functional analysis of med8 are not accurate.

Regarding concern 1:

- The authors explain that they analyzed 16 animals (2 per time point) processed into 10 Î¼m thick sections, producing a total of 353 sections. However, they do not specify whether the same number of sections were analyzed per animal, nor do they indicate the total size (or thickness) and cell number of the animals analyzed.

In this regard, it may be that the number of sections analyzed per animal is shown in Figure S2A (this is not clear from the figure legend or the text). If so, why is there variation in the number of sections per animal? Is it due to differences in animal size? If so, how were these differences addressed in order to integrate the data from different animals?
- In Figure 1F, the different parts of the blastema are divided according to the pigmented/unpigmented area. What were the criteria used to divide each blastema into three parts (proximal, middle, and distal)? Gene expression? Length of the region? This should be clarified.
- A schematic overview of each strategy used to obtain the results would help the reader understand the procedures.
Regarding concern 2:
- Throughout the study, the authors refer to Han et al., submitted for the custom framework used to create the atlas. Since that study is not currently available, this important information is missing from the current manuscript.
- Similarly, from the section "Spatiotemporal dynamics of positional gradients during whole-body regeneration" onward, the study relies on the so-called SBGs (spatially biased genes), also described in Han et al., submitted, which is not published. This is very important data that, if not published elsewhere, must be included in the current manuscript. A description of how the SBGs were obtained, together with a list and explanation, is required. Otherwise, essential information about the SBGsâ€”on which the subsequent analyses dependâ€”is entirely missing.
- According to the authors, PCGs are a subset of SBGs. What is the essential difference between PCGs and SBGs?
Regarding concern 3:
- Functional analysis of med8. The authors refer to a role of this gene in polarity remodeling. First, this is an unclear concept, because polarity establishment and polarity remodeling are two different processes during regeneration. Second, the RNAi results presented do not support a role for med8 in polarity. The phenotype suggests that med8 is required for cell-fate specification during early stages of regeneration, since neoblasts increase but differentiated cells decrease in general. However, the results do not support a role for med8 in pole formation. The authors report a decrease in sfrp-1, but only at later stages, and no data are shown for notum. The conclusion that med8 regulates blastema growth and positional information is therefore not accurate and does not align with the results presented.
Additional main concerns:
- A spatiotemporal transcriptomic atlas of planarian regeneration was already published in Cui et al. 2023. Although the authors cite it in the introduction, they should also compare their results with those published previously. Do they observe similar cell types and gene expression dynamics at the time points analyzed? This comparison should appear in both the Results and Discussion sections.
- The authors state that the different clusters of SBGs fluctuate during regeneration and then stabilize. First, which genes belong to each cluster? This information should be included. Second, according to Figure 2A, some clusters fluctuate (e.g., A1 and A2) but others do not (e.g., A5 or M9). A more accurate interpretation is required.
Furthermore, the starting point of the analysis is t0, when head and tail are missing, and the endpoint is 14 days of regeneration. How can one assess whether gene expression stabilizes if the starting and ending states are completely different?

Importantly, in Figure 2A, the cluster labels in the image do not correspond to the bars, and there are 15 bars but 16 clusters. The figure should be corrected. The colormap labels are also missing.
- The authors find that predictions from the Gierer-Meinhardt model are consistent with the expression of some genes (ARNT, Ndk, EGR1…). First, a description and reference for these genes are required. Second, what about other genes involved in Wnt signaling and AP patterning, previously proposed to follow this model in Werner et al. 2015? Third, how can transcription factors such as Hox4b follow the model if they are not secreted?
- In general, the manuscript does not refer to specific PCGs known to be critical for regeneration and patterning, such as notum and wnt1. Is this because they could not be identified in the dataset? If so, is it not possible to perform FISH and integrate these results with the Stereo-seq data?
Additional issues to be addressed:
- Lines 161-163: Why do the authors conclude that the increase in dorsal epidermal progenitors, neural progenitors, and pharyngeal lineages indicates an active wound response or the generation of new tissue? This statement is not clear.
- The 4D atlas annotates 36 refined cell types, which is a noteworthy result when compared with published scRNA-seq databases. The authors should compare their results with scRNA-seq in terms of the number and identity of cell types.
- In Figure 1F, the blastema is divided according to the pigmented/unpigmented region. However, in Figure 1G the dividing lines do not follow this curvature. Should they not follow the pigment curvature as well?
Additionally, why do the blastemas in Figure 1G show such pronounced lateral deviation? Is this because the incisions were not perpendicular to the AP axis? If so, with this variability, it is difficult to understand how the datasets could be integrated. A detailed explanation is required.
- Lines 202-203: "To test this, we analyzed the spatiotemporal patterns of SBGs by mapping representative samples from each time point onto clusters along the A/P axis using logistic regression (Fig. 2A)." What is meant by "representative samples"? The procedure should be specified more clearly.
- Data S4 is not mentioned in the text.
- smed03831, caveolin3, and smed01640 are the genes enriched in the ARZ domain. However, the authors perform functional analysis only with med8. Is there any specific reason for this?

Methods

Are the methods appropriate to the aims of the study, are they well described, and are necessary controls included? Choose an item.

Conclusions

Are the conclusions adequately supported by the data shown? Choose an item.

Reporting Standards

Does the manuscript adhere to the journal's guidelines on [minimum standards of reporting?] Choose an item.

Choose an item.

Statistics

Are you able to assess all statistics in the manuscript, including the appropriateness of statistical tests used? Choose an item.

Quality of Written English

Please indicate the quality of language in the manuscript: Choose an item.

Declaration of Competing Interests

Please complete a declaration of competing interests, considering the following questions:

- Have you in the past five years received reimbursements, fees, funding, or salary from an organisation that may in any way gain or lose financially from the publication of this manuscript, either now or in the future?
- Do you hold any stocks or shares in an organisation that may in any way gain or lose financially from the publication of this manuscript, either now or in the future?
- Do you hold or are you currently applying for any patents relating to the content of the manuscript?
- Have you received reimbursements, fees, funding, or salary from an organization that holds or has applied for patents relating to the content of the manuscript?
- Do you have any other financial competing interests?
- Do you have any non-financial competing interests in relation to this paper?

If you can answer no to all of the above, write 'I declare that I have no competing interests' below. If your reply is yes to any, please give details below.

I declare that I have no competing interests.

I agree to the open peer review policy of the journal. I understand that my name will be included on my report to the authors and, if the manuscript is accepted for publication, my named report including any attachments I upload will be posted on the website along with the authors' responses. I agree for my report to be made available under an Open Access Creative Commons CC-BY license (http://creativecommons.org/licenses/by/4.0/). I understand that any comments which I do not wish to be included in my named report can be included as confidential comments to the editors, which will not be published.

Choose an item.

To further support our reviewers, we have joined with Publons, where you can gain additional credit to further highlight your hard work (see: https://publons.com/journal/530/gigascience). On publication of this paper, your review will be automatically added to Publons, you can then choose whether or not to claim your Publons credit. I understand this statement.

Yes Choose an item.
